# In silico analysis of bacterial translation factors reveal distinct translation event specific pI values

**DOI:** 10.1186/s12864-021-07472-x

**Published:** 2021-03-29

**Authors:** Soma Jana, Partha P. Datta

**Affiliations:** Department of Biological Sciences, Indian Institute of Science Education and Research Kolkata, Mohanpur, WB PIN 741246 India

**Keywords:** Ribosome, Translation, Translation factors, Isoelectric point, Molecular weight, Phylogeny

## Abstract

**Background:**

Protein synthesis is a cellular process that takes place through the successive translation events within the ribosome by the event-specific protein factors, namely, initiation, elongation, release, and recycling factors. In this regard, we asked the question about how similar are those translation factors to each other from a wide variety of bacteria? Hence, we did a thorough in silico study of the translation factors from 495 bacterial sp., and 4262 amino acid sequences by theoretically measuring their pI and MW values that are two determining factors for distinguishing individual proteins in 2D gel electrophoresis in experimental procedures. Then we analyzed the output from various angles.

**Results:**

Our study revealed the fact that it’s not all same, or all random, but there are distinct orders and the pI values of translation factors are translation event specific. We found that the translation initiation factors are mainly basic, whereas, elongation and release factors that interact with the inter-subunit space of the intact 70S ribosome during translation are strictly acidic across bacterial sp. These acidic elongation factors and release factors contain higher frequencies of glutamic acids. However, among all the translation factors, the translation initiation factor 2 (IF2) and ribosome recycling factor (RRF) showed variable pI values that are linked to the order of phylogeny.

**Conclusions:**

From the results of our study, we conclude that among all the bacterial translation factors, elongation and release factors are more conserved in terms of their pI values in comparison to initiation and recycling factors. Acidic properties of these factors are independent of habitat, nature, and phylogeny of the bacterial species. Furthermore, irrespective of the different shapes, sizes, and functions of the elongation and release factors, possession of the strictly acidic pI values of these translation factors all over the domain Bacteria indicates that the acidic nature of these factors is a necessary criterion, perhaps to interact into the partially enclosed rRNA rich inter-subunit space of the translating 70S ribosome.

**Supplementary Information:**

The online version contains supplementary material available at 10.1186/s12864-021-07472-x.

## Background

The translation is a complex universal biological process that takes place in a large macromolecular machine called ribosome in all living organisms. It is an energy-exhaustive cellular process. In *Escherichia coli*, 40% of the total cellular energy is utilized by the translation system [1]. With the help of specific protein factors and aminoacyl tRNAs, ribosomes carry out protein synthesis following the decoding of the genetic information from mRNA in successive events, namely, initiation, elongation, and termination (release and recycling). The protein factors that are involved in the successive events are initiation factors (IF), elongation factors (EF), release factors (RF), and ribosome recycling factors (RRF). Here, the accurate coordination of every participant protein factor is necessary to perform the process successfully. Based on several years of biochemical and structural biological studies worldwide, fairly detailed knowledge of the mechanisms of cellular protein synthesis is now known [2–4]. However, in the broad aspect, which characteristics of the translation factors i.e., IF, EF and RF are necessary to be conserved for the accuracy of the universal process of protein synthesis among the different kinds of organisms need to be investigated.

In this study, we focused on the charge distribution (in terms of acidic and basic properties) of the translation factors throughout the domain Bacteria to comprehend the importance of the influence of the charge distribution of these factors on their accommodation on the ribosome and thus in their functions during this process of translation. For this, we made use of the principle of the 2D gel electrophoresis [5], whereby, we computed the pI values using the “Compute pI/Mw tool – ExPASy” (https://web.expasy.org/compute_pi/) online webserver. This web server calculates the pI values of proteins using pK values of amino acids as defined in [6–8], which were determined by examining polypeptide migration in an immobilized pH gradient (between pH 4.5 to 7.3) gel environment with 9.2 M and 9.8 M urea at 15 °C or 25 °C. In that study, the authors determined the focusing positions of 29 polypeptides of known amino acid sequence within a narrow range of immobilized pH gradients i.e., between pH 4.5 to 7.3 under denaturing conditions with 9.2 M and 9.8 M urea at 15 °C or 25 °C, respectively. They separately calculated the pI values of those proteins from their amino acid sequences. The comparison of isoelectric points of the proteins calculated from their amino acid sequences showed reliably good accuracy with the experimentally determined pl values. The reliability of the tool is broad, except for the study of highly basic proteins and small proteins. As the translational factors are not highly basic and also not too small, we believed our study was within the scope of the above mentioned web-based method. Our study revealed that the bacterial translational elongation and release factors have similar pI value distribution, and that was strictly acidic throughout the domain Bacteria. Irrespective of the habitat, nature, or the phylogeny of the bacterial species as well as irrespective of the different shapes, sizes, and functions of the elongation and release factors, these factors had strictly acidic pI values. We believe, our study indicates that the charge distribution of these factors might play important roles in the fidelity of the process of translation.

## Results

We studied 495 bacterial species throughout the domain of Bacteria. The habitats of these bacteria are very different from each other. The nature of these bacteria in terms of cell shape (coccus or bacillus), intracellular metabolic reactions (aerobic or anaerobic), and even the way they respond to the external environments (mesophilic or thermophilic or psychrophilic) are distinct [9]. Here, we studied the following bacterial phyla, such as Deinococcus-Thermus, Chlorobi, Actinobacteria, Firmicutes, Chlamydiae, Fusobacteria, Spirochaetes, Chloroflexi, Tenericutes, Cyanobacteria, Bacteroidetes, Thermotogae, Acidobacteria, Aquificae, Caldiserica, Chrysiogenetes, Deferribacteres, Elusimicrobia, Fibrobacteres, Gemmatimonadetes, Lentisphaerae, Nitrospirae, Planctomycetes, Thermodesulfobacteria, Verrucomicrobia, and Proteobacteria [10].

### pI and molecular weight value distribution of translation protein factors

**Fig. 1:**
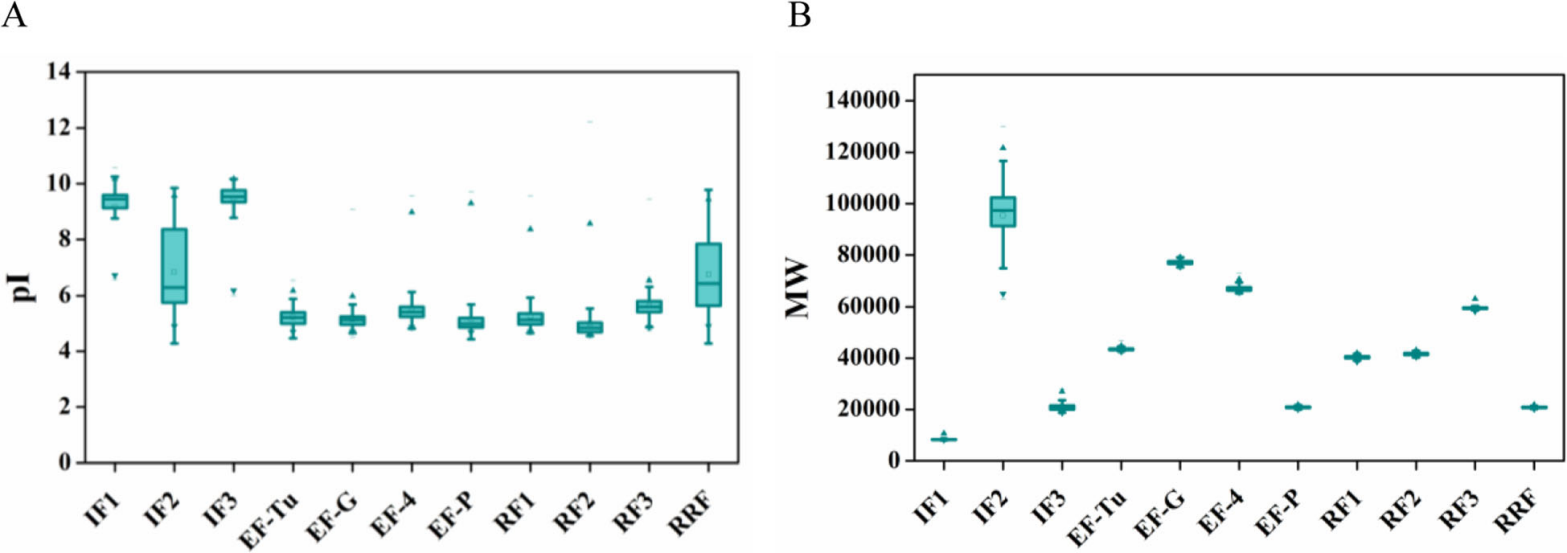
Box plot diagram of pI values and MW values of the translation factors. **a** pI value distribution of translation factors. **b** MW value distribution of translation factors. In both the cases, **a** and **b**, of the box plot diagrams, the lower hinge showed the first quartile (25%), whereas the upper hinge represented the third quartile (75%). The sign (−) above and below the box diagrams represented the maximum and minimum values respectively. The upper and lower solid triangles represented 99 and 1% values of the data set respectively. The horizontal line and the box inside the box plot represented the median and mean values of samples respectively

In the process of translation, we found a unique pattern of pI value distribution as depicted in Fig. 1a, (see Additional file 1; Table S1). The initiation factors, IF1, and IF3 were strictly basic except IF2. Conversely, the elongation and release factors were strictly acidic. On the other hand, like IF2, RRF also showed a broad range of pI value distribution ranging from acidic to basic. All the four quartiles of initiation factor 1 (IF1) and initiation factor 3 (IF3) were above pI 7. The elongation factor Tu (EF-Tu), elongation factor G (EF-G), elongation factor 4 (EF-4), & elongation factor P (EF-P) and the release factor 1 (RF1), release factor 2 (RF2), & release factor 3 (RF3) had all the four quartiles in the acidic range. For the comprehensive in silico study, along with the pI values, we also studied the molecular weight (MW) value distribution of these translation protein factors (Fig. 1b), (see Additional file 1; Table S1). Like pI value distribution, the protein IF2 showed a wide range of variations in MW value distribution as well (Fig. 1b). All the other proteins showed precise MW value distribution. A surprising observation is to be noted here that although RRF proteins showed a highly variable pI value distribution, their MW value distribution was quite narrow.

### Statistical analysis of pI values of translation factors

We further performed asymptotic tests [11] for 5% quantile and 95% quantile (Table 1) of these translation factors. We found that the *p* values corresponding to the null hypotheses (H0: q05 ≥ 7, and H0: q95 ≤ 9.95) for the 5 and 95% quantiles, respectively, for both the initiation factors, IF1 and IF3 to be more than 0.05, from which we inferred that 90% data lied in basic pI values, i.e., between 7 to 9.95. On the contrary, in the case of elongation (EF-Tu, EF-G, EF-4, and EF-P) and release factors (RF1, RF2, and RF3), 90% of data lied in completely acidic pI values i.e., between 4.635 and 6.225 (*p* values corresponding to H0: q05 ≥ 4.635 and H0: q95 ≤ 6.225 turned out to be more than 0.05, respectively). But we found a different scenario in the case of initiation factor, IF2, and ribosome recycling factor, RRF. In both these cases, 90% of data stretched in between acidic 5.1 to basic 9.25 (*p* values are more than 0.05 for H0: q05 ≥ 5.1 and H0: q95 ≤ 9.25, respectively).

**Table 1:** Asymptotic tests for 5% quantile and 95% quantile for translation factors

Translation Factors (IF1 & IF3)	5% sample quantiles	*p*-values (H_0_: q_05_ ≥ 7)	95% sample quantiles	*p*-values (H_0_: q_95_ ≤ 9.95)
IF1	6.82	0.2839	9.98	0.1684
IF3	8.87	0.5	9.94	0.6444
Translational Factors (IF2)	5% sample quantiles	*p*-values (H_0_: q_05_ ≥ 5.1)	95% sample quantiles	*p*-values (H_0_: q_95_ ≤ 9.25)
IF2	5.09	0.4173	9.31	0.1198
Translational Factors (Elongation and Release Factors)	5% sample quantiles	*p*-values (H_0_: q_05_ ≥ 4.635)	95% sample quantiles	*p*-values (H_0_: q_95_ ≤ 6.225)
EF-Tu	4.81	0.9861	5.77	0.7839
EF-G	4.78	0.9573	5.54	0.7272
EF-4	4.99	0.5337	6.36	0.0909
EF-P	4.77	0.9978	5.75	0.5473
RF1	4.81	0.9746	6.06	0.8042
RF2	4.62	0.1005	5.47	0.5
RF3	5.03	0.6555	6.14	0.8347
Translational Factors (RRF)	5% sample quantiles	*p*-values (H_0_: q_05_ ≥ 5.1)	95% sample quantiles	*p*-values (H_0_: q_95_ ≤ 9.25)
RRF	5.063	0.1999	9.028	0.9905

### Amino acid frequency distribution of elongation and release factors

**Fig. 2:**
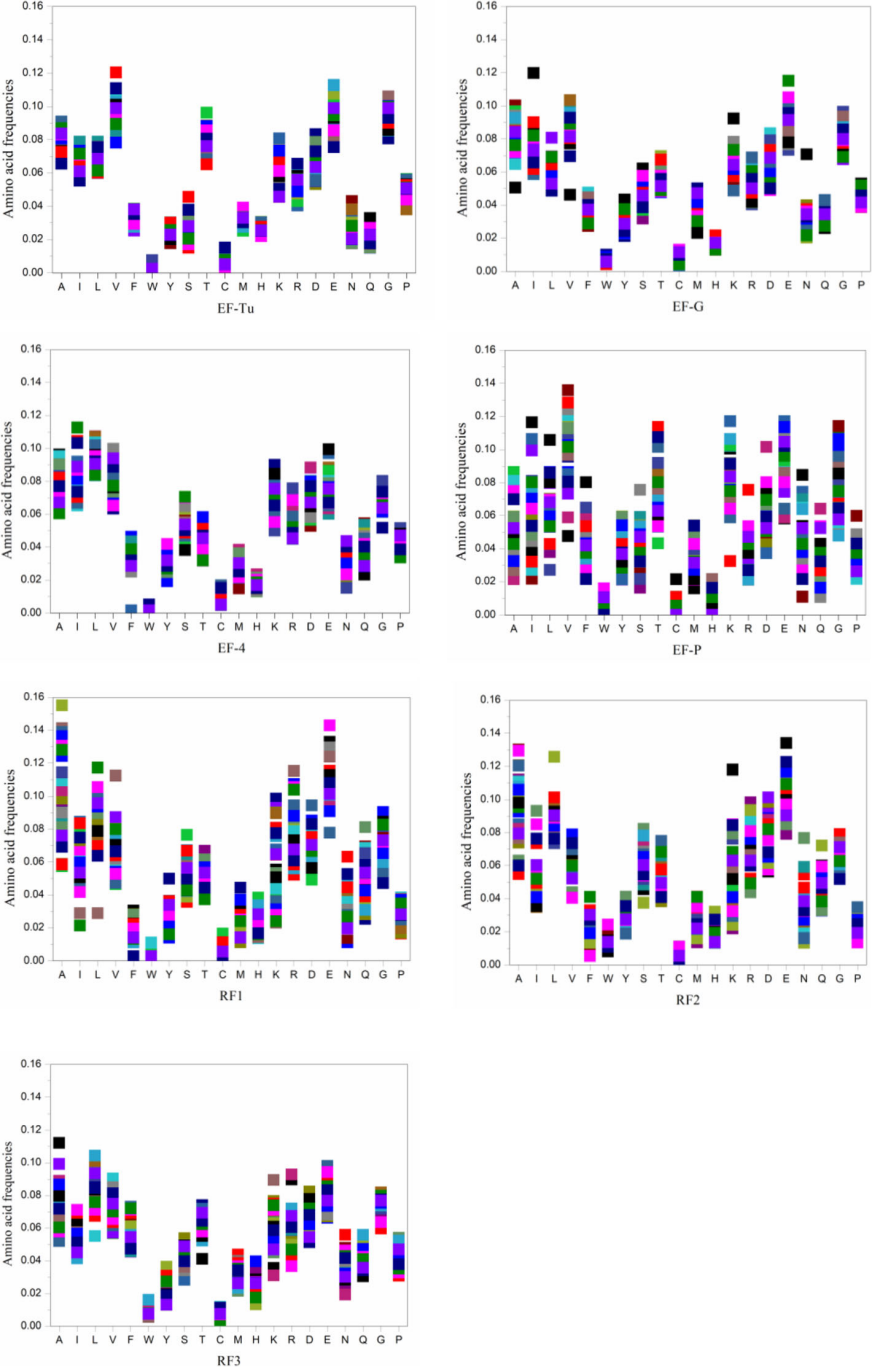
Amino acid frequency distribution of elongation and release factors. In each case of the elongation (EF-F, EF-G, EF-4, and EF-P) and release factors (RF1, RF2, and RF3), we selected 60 amino acid sequences that correspond to 60 bacterial species to study the amino acid frequency distribution. Each colour represented each randomly selected bacterial species

Interestingly, when we randomly chose 60 amino acid sequences (representing 60 bacterial species) of each of the elongation and release factors and calculated their amino acid frequencies, we found the occurrence of a high frequency of glutamic acid in all of those factors, (Fig. 2). In 2001, Schwartz et al. [12] also observed that the cytosolic acidic proteins were also found to have a high frequency of glutamic acid.

### Surface charge distribution of the elongation and release factors

**Fig. 3:**
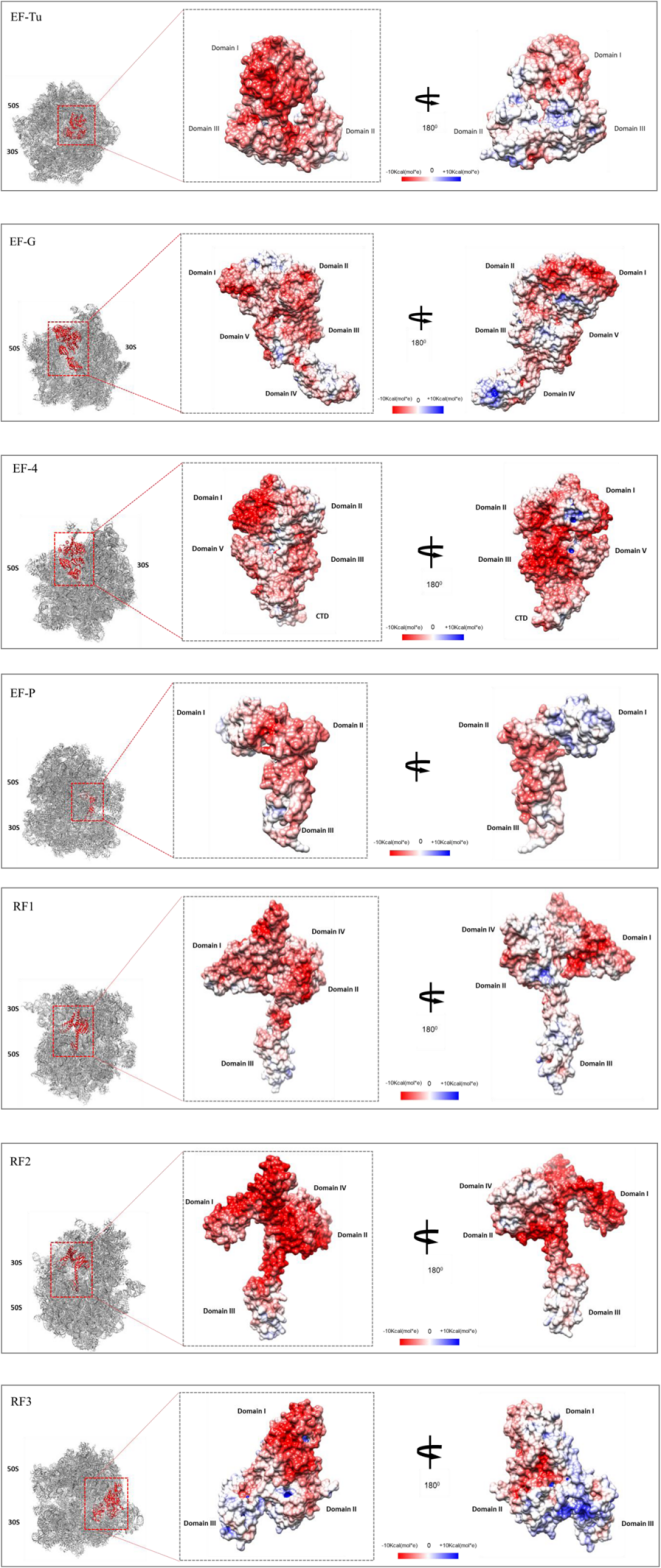
Surface charge distribution of the elongation and release factors. On the left of every panel, the 70S ribosome (grey ribbons) bound translation factor (inset, red ribbon) had been used as a thumbnail to reveal the corresponding orientation of translation factors shown next to it on the middle. The surface charges of the translation factors are shown in the middle. At the right of every panel surface charges of the translation factors had been displayed in 180 degree rotated state along the horizontal plane: The red dotted box (inset) indicated the location of the translation factors bound with 70S ribosome; EF-Tu – 70S ribosome (PDB ID: 5AFI) [47], EF-G – 70S ribosome (PDB ID: 3JA1) [48], EF-4 – 70S ribosome (PDB ID: 5J8B) [49], EF-P – 70S ribosome (PDB ID: 6ENJ) [50], RF1 – 70S ribosome (PDB ID: 6DNC) [51], RF2 – 70S ribosome (PDB ID: 5MDV) [52], and RF3 – 70S ribosome (PDB ID: 6GXM) [53]. The gray dotted boxes showed the surface charge distribution of the elongation and release factors [13–19]. All the domains of these factors were marked on the right side and the left side of their structures. The calculated electrostatic net charge of EF-Tu (PDB ID: 2FX3) was − 1.40e +01e, EF-G (PDB ID: 3J0E) was − 1.50e +01e, EF-4 (PDB ID: 3DEG) was − 2.00e +01e, EF-P (PDB ID: 3OYY) was − 8.00e +00e, RF1 (PDB ID: 4V7P) was − 1.40e +01e, RF2 (PDB ID: 5MGP) was − 2.60e +01e, RF3 (PDB ID: 4 V85) was − 7.00e +00e**.** Red and blue colour indicated negative charge and positive charge respectively whereas white colour indicates neutral charge

To further understand our observation, in the viewpoint of physiological context, we focused on the surface charge distribution of the atomic coordinates of these elongation and release factors; EF-Tu (PDB ID: 2FX3) [13], EF-G (PDB ID: 3J0E) [14], EF-4 (PDB ID: 3DEG) [15], EF-P (PDB ID: 3OYY) [16], RF1 (PDB ID: 4V7P) [17], RF2 (PDB ID: 5MGP) [18], and RF3 (PDB ID: 4 V85) [19]. We used online APBS-PDB2PQR software [20, 21], which employs Poisson-Boltzmann electrostatics calculations to analyze the surface charge of the translation protein factors mentioned above. We found out that though there are some patches of positive charges (blue) on the surface, the overall charge of all these factors (Fig. 3) is negative (red). We provided all the PDB IDs, studied here, in Table 2.

**Table 2:** PDB IDs of the translation factors, and translation factor and ribosome complex

Translation Factor	PDB ID	Translation Factor - Ribosome complex	PDB ID
EF-Tu	2FX3	EF-Tu – 70S ribosome	5AFI
EF-G	3J0E	EF-G – 70S ribosome	3JA1
EF-4	3DEG	EF-4 – 70S ribosome	5J8B
EF-P	3OYY	EF-P – 70S ribosome	6ENJ
RF1	4V7P	RF1 – 70S ribosome	6DNC
RF2	5MGP	RF2 – 70S ribosome	5MDV
RF3	4V85	RF3 – 70S ribosome	6GXM

### Relation of pI values of IF2 and RRF proteins with phylogeny

**Fig. 4:**
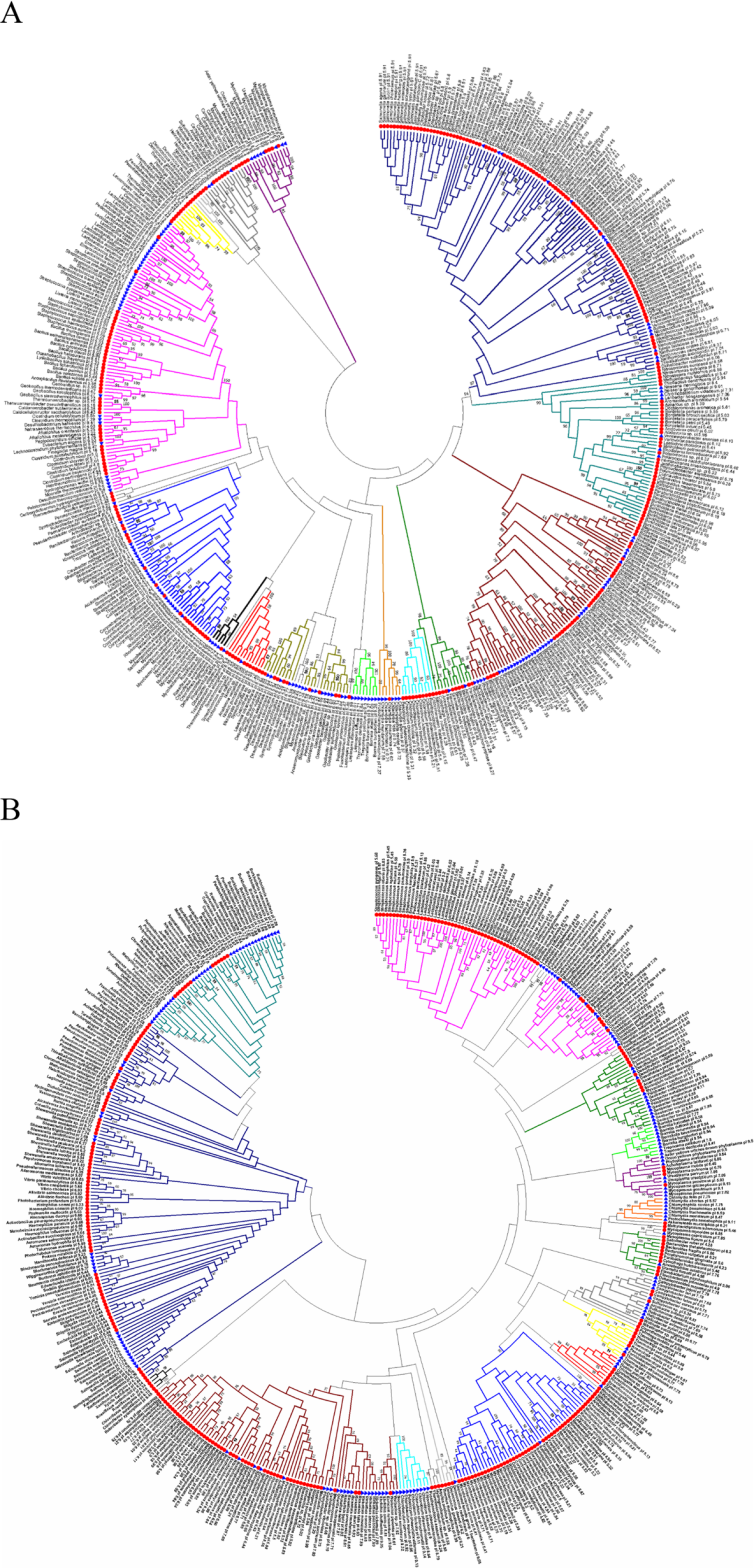
Phylogenetic tree constructed using primary amino acid sequences of IF2 and RRF proteins. **a** Phylogenetic analysis of IF2 protein. **b** Phylogenetic analysis of RRF protein. In both cases, **a** and **b**, we analyzed the evolutionary history using the Maximum Likelihood method based on the JTT matrix-based model. We took 500 bootstrap replicates to build the phylogenetic tree. Numbers near the branches refer to the bootstrap percentages (greater than 50% bootstrap replicates only shown here). The tree had been drawn to scale after eliminating all positions containing gaps and missing data. The branch lengths were measured by the number of substitutions per site. Blue triangles and red circles refer to the basic and acidic pI values respectively

Since IF2 had a wide range of pI value distribution from acidic to basic, we performed phylogenetic analysis (Fig. 4a) of the IF2 proteins (Additional file 1; Table S1) to investigate the relation of its pI value distribution with the phylogeny. In the case of the phylum Proteobacteria, we found that the class of Gammaproteobacteria (blue) and Betaproteobacteria (verdigris) were acidic (with only a few exceptions). Whereas the class Alphaproteobacteria (brown) had few genera as acidic (i.e., *Ehrlichia* spp.) and some genera as basic (i.e., *Brucella* spp. and *Bartonella* spp.), and others had both acidic and basic (i.e., *Rickettsia* spp.) pI values. In the case of other phyla, Chlorobi (cyan), Cyanobacteria (red), Thermotogae (yellow), and Deinococcus-Thermus (light grey), they had mostly acidic pI values, whereas the Chlamydiae (saffron) and Spirochaetes (light green) had basic pI values. The pI values of the IF2 protein in phyla Firmicutes (pink) and Actinobacteria (light blue) and Tenericutes (purple) had both the acidic and basic pI values.

The phylogenetic analysis of RRF (which had a wide range of pI value distribution) showed that the pI value distribution of RRF (Fig. 4b), (Additional file 1; Table S1) like IF2 (Fig. 4a) also linked to the phylogeny. We found that different classes of Proteobacteria had different pI value distribution. The Gammaproteobacteria (blue), and Alphaproteobacteria (brown) (with a few exceptions e.g., Genus; *Salmonella* spp. of Gammaproteobacteria and Genus; *Rickettsia* spp. and *Ehrlichia* spp. of Alphaproteobacteria) had acidic pI values. However, Betaproteobacteria (verdigris) (i.e., *Bordetella* spp. - acidic, *Burkholderia* spp. - basic) and Deltaproteobacteria (apple green) (i.e., *Desulfococcus* spp. – acidic, *Geobacter* spp. – basic,) had acidic and basic pI values as well. In the case of other phyla, Chlamydiae (safron), Chlorobi (cyan), and Spirochaetes (light green), they had basic pI values. In contrast, the phylum, Actinobacteria (light blue), and the phylum Firmicutes (pink) had both the acidic and basic pI values.

## Discussion

Our study revealed that irrespective of external environments or bacterial phylum, all the translation factors (except IF2 and RRF) are conserved throughout the domain Bacteria in terms of isoelectric point value distribution. Along with the translation process, we did additional studies on the pI value distribution of the two other universal processes of central dogma i.e., replication and transcription processes in domain Bacteria. We studied 529 number of bacterial sp., and 1707 number of amino acid sequences for replication (Additional file 2; Table S2) and 488 number of bacterial sp., and 1998 number of amino acid sequences for transcription (Additional file 3; Table S3). In the case of replication and transcription, some of the proteins showed a narrow range and others showed a wide range of pI value (Additional file 2; Fig. S1 and Additional file 3; Fig. S3 respectively) and molecular weight value (Additional file 2; Fig. S2 and Additional file 3; Fig. S4 respectively) distribution. Unlike translation factors, we found no specific pattern of pI value distribution of the proteins involved in the individual steps of the initiation, elongation, and release in those two processes. So, in conclusion, the observation of our study of the precise pI value distribution of the translation factors throughout the domain Bacteria indicates that the overall acidity or basicity of translation factors is an essential feature in the process of translation. The proteins involved in the initiation event of the process of translation i.e., initiation factors, were basic, whereas in the cases of the elongation and release events, i.e., elongation and release factors were strictly acidic due to the high frequency of negatively charged amino acids i.e., glutamic acids (Fig. 2). If we focus on the mode of interaction of these factors with the ribosome, we can categorize the facts i.e., initiation factors, IF1, IF2, and IF3 are involved in the formation of the 30S initiation complex, which is an open complex. On the other hand, the elongation and release factors interact with the ribosome when the 50S ribosomal subunit binds to the 30S initiation complex and all these three initiation factors eject from the initiation complex. Both the elongation and release factors irrespective of these proteins' different shapes, sizes, and functions interact with the A site of the semi-enclosed inter-subunit space of the translating 70S ribosome. Another important fact needs to be noted that the process of initiation of translation takes some seconds [22–24] to assemble the ribosome on the mRNA with the accordance of initiation factors but the elongation process happens at a faster rate than initiation. Several amino acids are incorporated within a second [22–24] and it continues until the whole mRNA gets read and the stop codon appears.

Based on our observation, if we focus our discussion on the molecular details of the individual steps of the process of translation, the importance of the charge distribution of the factors for the proper electrostatic interaction during this process will help to understand the process in a more comprehensive depiction. In case of initiation, a detailed biochemical and mutagenesis study on the interaction on IF1 and 30S ribosomal subunit showed that IF1 interacts with the 530 loop and helix 44 of 16S rRNA [25], which contains a highly negative charge. Thus the part of that surface region of IF1 is responsible for the interaction, which has the positive surface potential [25]. In the case of IF3, studies showed that site-directed mutagenesis of positively charged eight arginine residues, which are present in the IF3C domain, play an important role in the interaction with the 30S ribosomal subunit [25, 26].

In the case of elongation and release factors, in 2004, Trylska et al. [27], measured the electrostatic potential of the ribosomal A-site. They found a positive potential area in the A-site of the 70S ribosome complex that was mainly contributed by S12, L11, and S19 proteins. Biochemical and structural studies have shown that elongation factors; EF-Tu [28–30], EF-G [31–33], EF-4 [34], EF-P [35] interact with L11 protein, which is found to have the positive potential [27]. This positive potential contributed by these proteins of the A-site may be necessary for the interaction as it has been found that mutant lacking L-11 is extremely compromised in *E. coli* [36]. EF-G interacts with the S12 and S19 proteins as well [37]. This kind of interaction of the complementary electrostatic potential of the translation factors and the proteins of the A-site may help in the proper accommodation of these factors in the A-site. In this direction, a recent study [38], sheds light on the role of electrostatic interactions on the accommodation of cognate aa-tRNA in the A site, as well. In the next step, the rotation of the 30S ribosomal subunit with respect to the ratchet-like motion of the 50S ribosomal subunit causes the rearrangement of the electrostatic potential of the A-site i.e., a reduction of the positive potentials around the A-site. Thus it promotes the process of translocation [27] of tRNA from A-site to P-site and then from P-site to E-site. In the case of release factors, the positive potential of L11 causes the proper accommodation of the negative potential containing release factors, RF1 and RF2. After the RF3-induced ribosome rearrangements, the interactions between RF1/RF2 and the L11 region break, which causes the release of RF1/RF2 [39, 40]. On the other hand, the wide range of pI value distribution of IF2 and RRF reveals that the conservedness with respect to the acidic and basic properties of this translation factor may not be as important as the other translation factors in bacteria.

In this study, we took into account a wide range of bacterial species that belong to the entire domain of Bacteria on earth. For the sake of survival, bacteria evolve numerous mechanisms to adapt to that environment. The habitat of these bacteria vary in a wide range from the soil, water, food, industrial waste, deep ocean, acidic hot springs, in symbiotic and parasitic relationships with animals and plants, and radioactive waste also [41]. The nature of these bacteria are also different (i.e., acidophiles, alkaliphiles, aerobic, anaerobic, phototrophs, chemotrophs, nitrogen-fixing Bacteria, nitrifying and denitrifying bacteria, bioluminescent bacteria, free-living bacteria, enteric bacteria, and obligate intracellular parasites) [41]. Irrespective of the wide range of phylogeny, habitat, and nature of these bacteria, our statistical test showed that except IF2 and RRF, all the initiation, elongation, and release factors are conserved in terms of pI values all over the domain Bacteria.

Besides the elongation factors, the highly conserved basic pI value distribution of the initiation factors, IF1 and IF3, indicated that the pI values of these two translation factors are also not affected by phylogeny, nature, or habitat of the bacteria. The wide range of pI value distribution of IF2 and RRF (Fig. 4a and Fig. 4b respectively) unveiled that different phyla of bacteria had different traits of pI value distribution.

## Conclusions

**Fig. 5:**
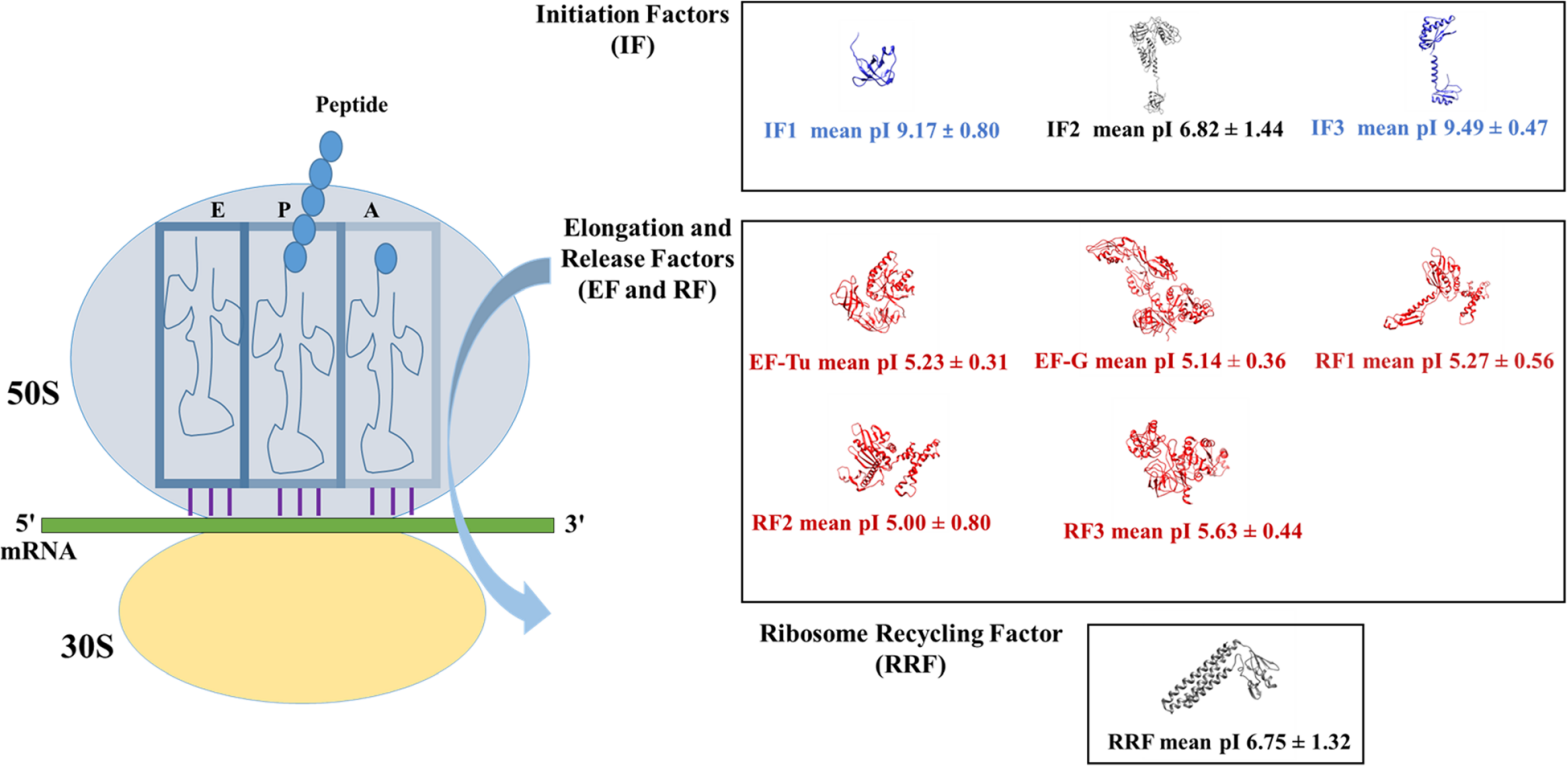
Summary of the study as a schematic representation. This figure showed translating intact 70S ribosome, initiation, elongation, release, and recycling factors along with the mean pI values and standard deviation values. Red and blue colour referred to the acidic and basic mean pI values, respectively. The dark grey colour of IF2 and RRF represented the mean pI values close to neutral with high standard deviation values

We concluded our study with a pictorial description of our findings in Fig. 5, where we depicted the mean pI value distribution along with the standard deviation values of all the translation factors in bacteria that showed distinct translation event specificity.

## Methods

### Data collection

We studied the following translation factors viz., IF1, IF2, IF3, EF-Tu, EF-G, EF-4, EF-P, RF1, RF2, RF3, and RRF from bacteria that directly interact with ribosome. Between the reviewed and unreviewed categories of the protein sequences of the UniProt [42] database, we collected the reviewed only for the accuracy of sample data. We removed all the incomplete fragments and repeated sequences as well to circumvent erroneous assumptions.

We calculated pI and MW values from 4262 reviewed amino acid sequences (Additional file 1; Table S1) of the bacterial translation factors, and those pI values, and MW values, and the corresponding accession numbers had been provided with the Additional file 1; Table S1.

### Method of pI value and MW value calculation

We used the “Compute pI/MW tool” (http://web.expasy.org/compute_pi/) of the ExPaSy-Bioinformatic resource portal to calculate the pI value and MW value. We chose this “Compute pI/MW tool” webserver because it shows reasonable good agreements of the calculated pI values with the experimentally determined pI values [6–8].

### Statistical test

We performed the asymptotic test [11] for the translation proteins for 5% quantile and 95% quantile. We calculated the *p* values corresponding to the null hypotheses for 5% and 95% quantiles for the translation proteins in MATLAB (R2019b) software (https://in.mathworks.com/products/new_products/release2019b.html). We generated all the graphs of this study in OriginPro 8.5 software (Origin (Pro), “Version 2019b”) [43].

### Electrostatic potential calculation

We downloaded the following atomic coordinates, viz., 2FX3 of EF-Tu, 3J0E of EF-G, 3DEG of EF-4, 3OYY of EF-P, 4V7P of RF1, 5MGP of RF2, and 4V85 of RF3 from the Protein Data Bank (PDB) database (www.rcsb.org). We deleted all the ions, and solvents, and other chemical modifications using Chimera software [44] (https://www.rbvi.ucsf.edu/chimera/). We calculated charges of these factors in APBS-PDB2PQR software, (https://server.poissonboltzmann.org/), that uses the Poisson Boltzmann equation to calculate the charge of a molecule. We used the output file to visualize the surface charge of these factors in Chimera software.

### Phylogenetic analysis

We used MEGA7 software [45, 46] to investigate the distribution of the pI values of IF2 and RRF protein in the bacterial taxonomy. We used primary amino acid sequences to construct the phylogenetic trees in both the cases of IF2 and RRF protein. We used 500 bootstrap replicates to analyze the phylogenetic tree, and we presented here the tree having the highest log-likelihood.

## Supplementary Information

**Additional file 1: **Proteins of the process of translation. **Table S1.** Accession numbers, pI values and MW values of the proteins of translation factors.

**Additional file 2: **Proteins of the process of replication. **Fig. S1** and **S2.** Box plot diagram of pI value and molecular weight value distribution respectively of the proteins of the process of replication. **Table S2.** Accession numbers, pI values and MW values of the proteins of replication factors.

**Additional file 3: **Proteins of the process of transcription. **Fig. S3** and **S4.** Box plot diagram of pI values, and molecular weight value distribution of the proteins of the process of transcription. **Table S3.** Accession numbers, pI values and MW values of the proteins of transcription factors.

## Data Availability

We obtained amino acid sequences of all the translation factors (used in this study) for pI value and molecular weight value calculation from the Uniprot database (www.uniprot.org). We provided the pI values and the molecular weight values (calculated by using “Compute pI/Mw tool – ExPASy” (web.expasy.org/compute_pi)) along with the accession numbers of these translation factors in the Additional file 1; Table S1. We obtained the identification code (ID) of the three-dimensional structures of the elongation and release factors from the Protein Data Bank (PDB) database (www.rcsb.org). Here, we provided all the PDB IDs used in this study in Table 2.

## Abbreviations

IF: Initiation factors
EF: Elongation factors
RF: Release factors
IF1: Initiation factor 1
IF2: Initiation factor 2
IF3: Initiation factor 3
EF-Tu: Elongation factor Tu
EF-G: Elongation factor G
EF-4: Elongation factor 4
EF-P: Elongation factor P
RF1: Release factor 1
RF2: Release factor 2
RF3: Release factor 3
RRF: Ribosome recycling factor
MW: Molecular weight
pI: Isoelectric point

## References

[1] Wilson DN, Nierhaus KH (2007). The weird and wonderful world of bacterial ribosome regulation. Crit Rev Biochem Mol Biol.

[2] Ramakrishnan V (2002). Ribosome structure and the mechanism of translation. Cell.

[3] Noller HF, Lancaster L, Mohan S, Zhou J (2017). Ribosome structural dynamics in translocation: yet another functional role for ribosomal RNA. Q Rev Biophys.

[4] Noeske J, Cate JH (2012). Structural basis for protein synthesis: snapshots of the ribosome in motion. Curr Opin Struct Biol.

[5] O’Farrell PH (1975). High resolution two-dimensional electrophoresis of proteins. J Biol Chem.

[6] Bjellqvist B, Hughe GJ, Pasquali C, Paquet N, Ravier F, Sanchez J-C, Frutiger S, Hochstrasser DF (1993). The focusing positions of polypeptides in immobilized pH gradients can be predicted from their amino acid sequences. Electrophoresis.

[7] Bjellqvist B, Basse B, Olsen EJ, Celis E (1994). Reference points for comparisons of two-dimensional maps of proteins from different human cell types defined in a pH scale where isoelectric points correlate with polypeptide compositions. Electrophoresis.

[8] Gasteiger E, Hoogland C, Gattiker A, Duvaud S, Wilkins MR, Appel RD, Bairoch A. Protein Identification and Analysis Tools on the ExPASy Server. In: Walker JM, editor. The Proteomics Protocols Handbook. Totowa: Humana Press; 2005.

[9] Prescott LM, Harley GP, Klein DE. Microbiology, W. Brown publishers, Dubuque, Iowa, USA, 2nd edition, 1993.

[10] Madigan MT, Brock TD. Brock biology of microorganisms. 12th ed. San Francisco: Pearson/Benjamin Cummings; 2009.

[11] Serfling RJ. Approximation theorems of mathematical statistics. 1980.

[12] Schwartz R, Ting CS, King J (2001). Whole proteome pI values correlate with subcellular localizations of proteins for organisms within the three domains of life. Genome Res.

[13] Heffron SE, Moeller R, Jurnak F (2006). Solving the structure of *Escherichia coli* elongation factor Tu using a twinned data set. Acta Crystallogr D Biol Crystallogr.

[14] Yokoyama T, Shaikh TR, Iwakura N, Kaji H, Kaji A, Agrawal RK (2012). Structural insights into initial and intermediate steps of the ribosome-recycling process. EMBO J.

[15] Connell SR, Topf M, Qin Y, Wilson DN, Mielke T, Fucini P, Nierhaus KH, Spahn CMT (2008). A new tRNA intermediate revealed on the ribosome during EF4-mediated back-translocation. Nat Struct Mol Biol.

[16] Choi S, Choe J (2011). Crystal structure of elongation factor P from *Pseudomonas aeruginosa* at 1.75 angstrom resolution. Proteins.

[17] Korostelev A, Zhu J, Asahara H, Noller HF (2010). Recognition of the amber UAG stop codon by release factor RF1. EMBO J.

[18] Huter P, Muller C, Beckert B, Arenz S, Berninghausen O, Beckmann R, Wilson DN (2017). Structural basis for ArfA-RF2-mediated translation termination on mRNAs lacking stop codons. Nature.

[19] Zhou J, Lancaster L, Trakhanov S, Noller HF (2012). Crystal structure of release factor RF3 trapped in the GTP state on a rotated conformation of the ribosome. RNA.

[20] Baker NA, Sept D, Joseph S, Holst MJ, McCammon JA. Electrostatics of nanosystems: application to microtubules and the ribosome. Proc Natl Acad Sci. 2001:10037–41. 10.1073/pnas.181342398 PMC56910 11517324

[21] Dolinsky TJ, Nielsen JE, McCammon JA, Baker NA (2004). PDB2PQR: an automated pipeline for the setup, execution, and analysis of Poisson-Boltzmann electrostatics calculations. Nucleic Acids Res.

[22] Wintermeyer W, Peske F, Beringer M, Gromadski KB, Savelsbergh A, Rodnina MV (2004). Mechanisms of elongation on the ribosome: dynamics of a macromolecular machine. Biochem Soc Trans.

[23] Lovmar M, Ehrenberg M (2006). Rate, accuracy and cost of ribosomes in bacterial cells. Biochemie.

[24] Rodnina MV, Beringer M, Wintermeyer W (2007). How ribosomes make peptide bonds. Trends Biochem Sci.

[25] Laursen BS, Sorensen HP, Mortensen KK, Sperling-Petersen HU (2005). Initiation of protein synthesis in bacteria. Microbiol Mol Biol Rev.

[26] Petrelli D (2003). C Garofalo, M Lammi, R Spurio, C L Pon, CO Gualerzi and ALa Teana. Mapping the active sites of bacterial translation initiation factor IF3. J Mol Biol.

[27] Trylska J, Konecny R, Tama F, Brooks CL, McCammon JA (2004). Ribosome motions modulate electrostatic properties. Biopolymers.

[28] Schmeing TM, Voorhees RM, Kelley AC, Gao YG, Murphy FV, Weir JR, Ramakrishnan V (2009). The crystal structure of the ribosome bound to EF-Tu and aminoacyl-tRNA. Science.

[29] Morse JC, Girodat D, Burnett BJ, Holm M, Altman RB, Sanbonmatsu KY, Wieden HJ, Blanchard SC (2020). Elongation factor-Tu can repetitively engage aminoacyl-tRNA within the ribosome during the proofreading stage of tRNA selection. PNAS.

[30] Loveland AB, Demo G, Korostelev AA (2020). Cryo-EM of elongating ribosome with EF-Tu•GTP elucidates tRNA proofreading. Nature.

[31] Bowen WS, Van Dyke N, Murgola EJ, Lodmell JS, Hill WE (2005). Interaction of thiostrepton and elongation factor-G with the ribosomal protein L11-binding domain. J Biol Chem.

[32] Wang Y, Qin H, Kudaravalli RD, Kirillov SV, Dempsey GT, Pan D, Cooperman BS, Goldman YE (2007). Single-molecule structural dynamics of EF-G-ribosome interaction during translocation. Biochemistry.

[33] Lin J, Gagnon MG, Bulkley D, Steitz TA (2015). Conformational changes of elongation factor G on the ribosome during tRNA translocation. Cell.

[34] Kumar V, Ero R, Ahmed T, Goh KJ, Zhan Y, Bhushan S, Gao YG (2016). Structure of the GTP form of elongation factor 4 (EF4) bound to the ribosome. J Biol Chem.

[35] Aoki H, Xu J, Emili A, Chosay JG, Golshani A, Ganoza MC. Interactions of elongation factor EF-P with the *Escherichia coli* ribosome. FEBS J. 2008:275–671. 10.1111/j.1742-4658.2007.06228.x 18201202

[36] Stoffler G, Cundliffe E, Stofflermeilicke M, Dabbs ER (1980). Mutants of *Escherichia coli* lacking ribosomal protein L11. J Biol Chem.

[37] Skold SE (1982). Chemical crosslinking of elongation factor G to both subunits of the 70S ribosome from *E. coli*. Eur J Biochem.

[38] Noel JK, Whitford PC (2016). How EF-Tu can contribute to efficient proofreading of aa-tRNA by the ribosome. Nat Commun.

[39] Gao H, Zhou Z, Rawat U, Huang C, Bouakaz L, Wang C, Cheng Z, Liu Y, Zavialov A, Gursky R, Sanyal S, Ehrenberg M, Frank J, Song H (2007). RF3 induces ribosomal conformational changes responsible for dissociation of class I release factors. Cell.

[40] Adio S, Sharma H, Senyushkina T, Karki P, Maracci C, Wohlgemuth I, Holtkamp W, Peske F, Rodnina MV (2018). Dynamics of ribosomes and release factors during translation termination *in E coli*. eLife.

[41] Gupta A, Gupta R, Singh RL: Microbes and environment. In Principles and Applications of Environmental Biotechnology for a Sustainable Future 2017;43–84.

[42] Pichler K, Warner K, Magrane M (2018). UniProt consortium, SPIN: submitting sequences determined at protein level to UniProt. Curr Protoc Bioinformatics.

[43] Origin (Pro), "Version 2019b". OriginLab Corporation, Northampton. https://www.originlab.com/.

[44] Pettersen EF, Goddard TD, Huang CC, Couch GS, Greenblatt DM, Meng EC, Ferrin TE (2004). UCSF chimera - a visualization system for exploratory research and analysis. J Comput Chem.

[45] Jones DT, Taylor WR, Thornton JM (1992). The rapid generation of mutation data matrices from protein sequences. Comput Appl Biosci.

[46] Tamura K, Stecher G, Peterson D, Filipski A, Kumar S (2013). MEGA6: molecular evolutionary genetics analysis version 6.0. Mol Biol Evol.

[47] Fischer N, Neumann P, Konevega AL, Bock LV, Ficner R, Rodnina MV, Stark H (2015). Structure of the *E. coli* ribosome-EF-Tu complex at <3 angstrom resolution by Cs-corrected cryo-EM. Nature.

[48] Li W, Liu Z, Koripella RK, Langlois R, Sanyal S, Frank J (2015). Activation of GTP hydrolysis in mRNA-tRNA translocation by elongation factor G. Sci Adv.

[49] Gagnon MG, Lin J, Steitz TA (2016). Crystal structure of elongation factor 4 (EF-4/LepA) in complex with GDPCP bound to the *Thermus thermophilus* 70S ribosome. Proc Natl Acad Sci.

[50] Huter P, Arenz S, Bock LV, Graf M, Frister JO, Heuer A, Peil L, Starosta AL, Wohlgemuth I, Peske F, Novacek J, Berninghausen O, Grubmuller H, Tenson T, Beckmann R, Rodnina MV, Vaiana AC, Wilson DN (2017). Structural basis for Polyproline-mediated ribosome stalling and rescue by the translation elongation factor EF-P. Mol Cell.

[51] Svidritskiy E, Demo G, Korostelev AA (2018). Mechanism of premature translation termination on a sense codon. J Biol Chem.

[52] James NR, Brown A, Gordiyenko Y, Ramakrishnan V (2016). Translational termination without a stop codon. Science.

[53] Graf M, Huter P, Maracci C, Peterek M, Rodnina MV, Wilson DN (2018). Visualization of translation termination intermediates trapped by the Apidaecin 137 peptide during RF3-mediated recycling of RF1. Nat Commun.

