# Supplementary material for: In silico analysis of bacterial translation factors reveal distinct translation event specific pI values
Source: BMC Genomics. 2021 Mar 29;22:220. doi: 10.1186/s12864-021-07472-x (PMC8008671; doi:10.1186/s12864-021-07472-x)
Supplement: Supplementary file 1 — Additional file 1: Proteins of the process of translation. Table S1. Accession numbers, pI values and MW values of the proteins of translation factors. [file 12864_2021_7472_MOESM1_ESM.docx]

**Additional file 1 of Accession numbers, pI values and MW values of the proteins of translation factors.**

Additional file 1: Table S1. Accession numbers, pI values and MW values of the proteins of translation factors.

| IF1 | Accession number | pI | MW |
| --- | --- | --- | --- |
| IF1_ECOLI_1 | P69222 | 9.23 | 8118.38 |
| IF1_BACSU_1 | P20458 | 7.07 | 8082.37 |
| IF1_LEPBL_1 | Q055C2 | 9.4 | 8193.64 |
| IF1_LACLA_1 | P0A3K4 | 8.29 | 8064.31 |
| IF1_MYCTU_1 | P9WKK3 | 9.45 | 8488.93 |
| IF1_THET8_1 | Q5SHR1 | 9.69 | 8233.65 |
| IF11_DESVH_1 | P61686 | 9.45 | 8328.77 |
| IF12_BURMA_1 | Q62GM6 | 9.6 | 8251.69 |
| IF12_CUPNH_1 | Q0K640 | 9.6 | 8224.66 |
| IF1_HAEIN_1 | P44322 | 8.95 | 8153.5 |
| IF1_BACHD_1 | O50630 | 8.33 | 8161.52 |
| IF1_LEPIN_1 | Q9XD14 | 9.4 | 8062.45 |
| IF1_STRR6_1 | P65122 | 8.06 | 8202.52 |
| IF1_SYNY3_1 | P73301 | 9.75 | 8681.2 |
| IF1_DEIRA_1 | Q9RSK1 | 9.39 | 9300.67 |
| IF1_FUSNN_1 | Q8R5W2 | 9.4 | 8214.66 |
| IF1_BACCR_1 | Q814C3 | 6.72 | 8185.56 |
| IF1_VIBCH_1 | P65128 | 9.22 | 8222.61 |
| IF11_BURCA_1 | Q1BRW9 | 9.6 | 8217.67 |
| IF11_POLAQ_1 | A4SUY2 | 9.6 | 8286.81 |
| IF1_AERS4_1 | A4SNL6 | 9.6 | 8190.59 |
| IF11_RALSO_1 | Q8XV33 | 9.6 | 8232.64 |
| IF1_CARHZ_1 | Q3A9T9 | 8.16 | 8243.6 |
| IF1_CHLAB_1 | Q5L5H5 | 9.8 | 8368.03 |
| IF1_BACFN_1 | Q5L8D1 | 9.1 | 8234.62 |
| IF1_CHLFF_1 | Q255F4 | 9.65 | 8367.98 |
| IF1_CHLPD_1 | A1BJ12 | 9.3 | 8183.65 |
| IF1_CHLTA_1 | Q3KM39 | 9.42 | 8412.98 |
| IF1_HELPY_1 | P65108 | 9.46 | 8216.66 |
| IF1_MYCGI_1 | A4TEI3 | 9.45 | 8502.96 |
| IF1_DEHM1_1 | Q3Z958 | 9.59 | 8247.62 |
| IF1_DESHY_1 | Q250K9 | 9.22 | 8209.67 |
| IF1_MYCPE_1 | Q8EUD5 | 9.75 | 8033.5 |
| IF1_BIFAA_1 | A1A091 | 9.45 | 8377.84 |
| IF1_MYCSK_1 | A1UBY0 | 9.45 | 8488.93 |
| IF1_PSEPG_1 | B0KLX5 | 9.15 | 8302.55 |
| IF1_PSEU2_1 | Q4ZRK8 | 9.15 | 8302.55 |
| IF1_LACP7_1 | A9KJH1 | 8.08 | 8178.61 |
| IF1_BRUC2_1 | A9M7T6 | 9.52 | 8337.86 |
| IF1_BRUO2_1 | A5VNK0 | 9.52 | 8371.88 |
| IF1_SHESM_1 | Q0HJP2 | 9.6 | 8272.7 |
| IF1_RHOBA_1 | Q7UEY5 | 9.86 | 8416.74 |
| IF1_NITWN_1 | Q3SVW2 | 9.98 | 10618.25 |
| IF1_GEOKA_1 | Q5L3R6 | 6.83 | 8269.59 |
| IF1_NOCSJ_1 | A1SNJ3 | 9.45 | 8447.92 |
| IF1_SULNB_1 | A6QCR9 | 8.79 | 8211.7 |
| IF1_SOLUE_1 | Q01QC0 | 10.13 | 8244.56 |
| IF1_RHOS4_1 | Q3J085 | 9.45 | 8249.66 |
| IF1_XANOR_1 | Q5GZR5 | 9.45 | 8375.65 |
| IF1_PARD8_1 | A6LEG9 | 9.4 | 8206.61 |
| IF1_GRAFK_1 | A0M577 | 9.05 | 8094.48 |
| IF1_HAEDU_1 | Q7VNW5 | 8.93 | 8266.73 |
| IF1_STAAC_1 | Q5HDY0 | 6.72 | 8279.64 |
| IF1_RICM5_1 | A8F2V3 | 9.98 | 8469.06 |
| IF1_MYCGA_1 | Q9R6P8 | 9.57 | 8059.56 |
| IF1_STAES_1 | Q8CRI1 | 6.72 | 8265.61 |
| IF1_VESOH_1 | A5CW05 | 9.17 | 8375.82 |
| IF1_VIBVU_1 | P65130 | 9.22 | 8222.61 |
| IF1_CLOTH_1 | A3DJJ6 | 6.73 | 8176.55 |
| IF1_CORDI_1 | P61685 | 9.15 | 8388.81 |
| IF1_COREF_1 | Q8FS36 | 9.15 | 8360.76 |
| IF11_AROAE_1 | Q5P6C0 | 10.11 | 9727.34 |
| IF1_TRIEI_1 | Q110C7 | 9.82 | 8565.02 |
| IF1_COLP3_1 | Q480P2 | 9.22 | 8247.6 |
| IF1_CAMJR_1 | Q5HSK1 | 8.79 | 8300.81 |
| IF1_CHLPM_1 | A4SCT1 | 9 | 8196.65 |
| IF12_BURVG_1 | A4JNX5 | 9.76 | 9801.23 |
| IF1_PSEA6_1 | Q15T99 | 7.8 | 8261.63 |
| IF1_SALAR_1 | A9MI00 | 9.22 | 8249.58 |
| IF1_PSEE4_1 | Q1IBD7 | 9.15 | 8302.55 |
| IF1_SALRD_1 | Q2S3P3 | 9 | 8422.83 |
| IF1_BLOFL_1 | Q7VR35 | 9.77 | 8336.77 |
| IF1_SHEB9_1 | A9L4G2 | 9.6 | 8272.7 |
| IF1_STRP8_1 | P65125 | 8.06 | 8272.65 |
| IF1_PSEU5_1 | A4VLV5 | 9.15 | 8302.55 |
| IF1_PSYA2_1 | Q4FRV1 | 9 | 8458.85 |
| IF1_PSYIN_1 | A1SSY1 | 9.6 | 8219.59 |
| IF1_NEIMA_1 | P65112 | 9.22 | 8294.71 |
| IF1_FRATH_1 | Q2A2Y6 | 9.25 | 8253.75 |
| IF1_NITHX_1 | Q1QR57 | 9.98 | 10592.21 |
| IF1_BUCAP_1 | Q8K9M8 | 9.52 | 8404.86 |
| IF1_NOCFA_1 | Q5Z1L4 | 9.45 | 8488.93 |
| IF1_LEUMM_1 | Q03ZM4 | 9.22 | 8090.46 |
| IF1_GEOSL_1 | P61688 | 8.05 | 8186.58 |
| IF1_SYNAS_1 | Q2LQB5 | 9.22 | 8327.82 |
| IF1_PAEAT_1 | A1R8R9 | 9.45 | 8463.96 |
| IF1_XYLFA_1 | Q9PDD4 | 9.25 | 8393.72 |
| IF1_PELCD_1 | Q3A4P9 | 9.45 | 8298.72 |
| IF1_PETMO_1 | A9BFZ5 | 9.85 | 8662.23 |
| IF11_ACIAC_1 | A1TL44 | 10.33 | 9696.16 |
| IF11_ACISJ_1 | A1W3R6 | 10.59 | 10149.72 |
| IF12_NOVAD_1 | Q2G552 | 10.18 | 9532.14 |
| IF11_BURP1_1 | Q3JUP8 | 9.96 | 10078.48 |
| IF1_ANADE_1 | Q2IQC5 | 9.34 | 8153.47 |
| IF1_CALS8_1 | A4XLQ7 | 9.39 | 8186.59 |
| IF1_ANAVT_1 | Q3MFA1 | 9.82 | 8565.02 |
| IF1_CHLMU_1 | P65107 | 9.42 | 8412.98 |
| IF12_BURL3_1 | Q39KE6 | 9.6 | 8217.67 |
| IF1_SACD2_1 | Q21K31 | 9.4 | 8296.57 |
| IF1_KLEP7_1 | A6T6Y1 | 9.22 | 8249.58 |
| IF1_LACBA_1 | Q03PX9 | 8.1 | 8274.63 |
| IF1_BORBU_1 | O51191 | 9.65 | 8475.06 |
| IF1_NITMU_1 | Q2YAX6 | 9.6 | 8250.7 |
| IF1_SHIFL_1 | P69227 | 9.23 | 8118.38 |
| IF1_RHOP2_1 | Q2IZ60 | 9.98 | 10693.32 |
| IF1_SULDN_1 | Q30TU3 | 9.13 | 8255.8 |
| IF1_GEOUR_1 | A5G3G6 | 9 | 8229.69 |
| IF1_OENOB_1 | Q04G64 | 9.45 | 8087.35 |
| IF1_GLUDA_1 | A9HFM1 | 9.86 | 8372.81 |
| IF1_METCA_1 | Q607H0 | 9.77 | 8404.77 |
| IF1_HAES1_1 | Q0I2S8 | 8.93 | 8284.7 |
| IF1_MYCA1_1 | A0QKV0 | 9.45 | 8488.93 |
| IF1_THEEB_1 | Q8DML3 | 9.82 | 8504.91 |
| IF1_RICTY_1 | Q68VT5 | 9.98 | 8210.62 |
| IF1_MYCGE_1 | P47419 | 9.52 | 8113.64 |
| IF11_BURCM_1 | Q0BJ25 | 9.6 | 8217.67 |
| IF12_THIDA_1 | Q3SIV9 | 9.89 | 10056.59 |
| IF1_CAMC5_1 | A7H0Z3 | 8.76 | 8302.78 |
| IF1_CAMHC_1 | A7HZX5 | 8.76 | 8332.81 |
| IF1_CHLCV_1 | Q822I3 | 9.65 | 8367.98 |
| IF1_CHLPN_1 | Q9Z9A8 | 9.36 | 8354.94 |
| IF1_BACP2_1 | A8F9A8 | 6.83 | 8171.49 |
| IF1_MYCH2_1 | Q601J2 | 9.52 | 8666.31 |
| IF1_POLNA_1 | A1VKX0 | 9.86 | 9535.98 |
| IF1_MYCLE_1 | P0A5H7 | 9.45 | 8357.74 |
| IF1_MYCPA_1 | P61691 | 9.45 | 8488.93 |
| IF1_PSEAB_1 | Q02NB1 | 9.15 | 8302.55 |
| IF1_MYCPN_1 | Q50298 | 9.89 | 9024.7 |
| IF1_KORVE_1 | Q1IS99 | 9.45 | 8311.82 |
| IF1_MYCS2_1 | A0QSL3 | 9.45 | 8502.96 |
| IF1_LACGA_1 | Q046A4 | 8.05 | 8287.73 |
| IF1_SHEFN_1 | Q081G6 | 9.6 | 8258.67 |
| IF1_BRUME_1 | P62923 | 9.52 | 8371.88 |
| IF1_SHESH_1 | A8FUH5 | 9.6 | 8274.67 |
| IF1_LEIXX_1 | Q6AD17 | 9.45 | 8435.91 |
| IF1_RHOFT_1 | Q21QP4 | 9.6 | 8219.64 |
| IF1_ONYPE_1 | P61692 | 9.79 | 9061.59 |
| IF1_GRABC_1 | Q0BTC4 | 9.86 | 8372.81 |
| IF1_PARDP_1 | A1B2Z3 | 9.45 | 8376.83 |
| IF1_YERP3_1 | A7FJZ1 | 9.22 | 8235.55 |
| IF1_METPP_1 | A2SLD6 | 9.6 | 8247.7 |
| IF1_MOOTA_1 | Q2RFS1 | 9.59 | 8291.74 |
| IF1_THEFY_1 | Q47LL7 | 9.1 | 8426.87 |
| IF1_THEMA_1 | P56866 | 9.86 | 8589.34 |
| IF1_RUTMC_1 | A1AXB0 | 9.57 | 8374.85 |
| IF1_CITK8_1 | A8AIJ9 | 9.22 | 8249.58 |
| IF1_CLOB1_1 | A7FZ49 | 6.72 | 8183.56 |
| IF1_DINSH_1 | A8LS17 | 9.45 | 8311.72 |
| IF1_PSEHT_1 | Q3IH34 | 9.22 | 8220.64 |
| IF1_BIFLO_1 | Q8G3Z7 | 9.45 | 8377.84 |
| IF1_LACH4_1 | A8YXM7 | 8.05 | 8273.71 |
| IF1_LACJO_1 | P61689 | 8.05 | 8287.73 |
| IF1_BRUA2_1 | Q2YPC1 | 9.52 | 8371.88 |
| IF1_LACSS_1 | Q38UT3 | 9 | 8120.5 |
| IF1_FRACC_1 | Q2JFF3 | 9.77 | 8542 |
| IF1_SHIBS_1 | Q323L9 | 9.22 | 8249.58 |
| IF1_RICPR_1 | Q9ZCE3 | 9.98 | 8224.65 |
| IF1_MYCBO_1 | P0A5H6 | 9.45 | 8357.74 |
| IF1_MYCCT_1 | Q2SRH5 | 9.63 | 8417.94 |
| IF1_PHOLL_1 | P65114 | 9.22 | 8235.55 |
| IF1_CLOPE_1 | Q8XHU6 | 8.14 | 8153.57 |
| IF11_BORA1_1 | Q2L254 | 9.6 | 8263.69 |
| IF1_DEIGD_1 | Q1IX94 | 9.39 | 9312.73 |
| IF1_MYCMO_1 | Q6KI33 | 9.05 | 8124.37 |
| IF1_DICNV_1 | A5EXE7 | 9.16 | 8400.74 |
| IF1_LACAC_1 | Q5FM69 | 8.05 | 8273.71 |
| IF1_ACICJ_1 | A5G201 | 9.86 | 8324.75 |
| IF1_SHELP_1 | A3QD86 | 9.6 | 8274.67 |
| IF1_FERNB_1 | A7HM29 | 6.58 | 10453.19 |
| IF1_PSYCK_1 | Q1QAF7 | 9 | 8458.85 |
| IF1_PELPD_1 | A1APV4 | 9.05 | 8229.65 |
| IF1_THEM4_1 | A6LLN6 | 9.75 | 8484.09 |
| IF1_CLOK5_1 | A5N4S0 | 6.72 | 8167.56 |
| IF1_COXBU_1 | Q83CD1 | 9.1 | 9325.69 |
| IF11_BORBR_1 | Q7WRA2 | 9.6 | 8263.69 |
| IF12_PARXL_1 | Q13M01 | 9.85 | 10157.53 |
| IF11_DECAR_1 | Q47J82 | 9.45 | 8330.82 |
| IF1_AERHH_1 | A0KJD8 | 9.6 | 8190.59 |
| IF1_ALIF1_1 | Q5E3Y7 | 9.22 | 8176.52 |
| IF1_ALKEH_1 | Q0A8P2 | 9.69 | 8246.62 |
| IF1_AZOC5_1 | A8I655 | 9.85 | 10563.99 |
| IF1_BACCN_1 | A7GK43 | 6.72 | 8185.56 |
| IF1_CYTH3_1 | Q11QD4 | 9.82 | 8287.75 |
| IF1_STRCO_1 | P60515 | 9.45 | 8410.87 |
| IF1_ACIBT_1 | A3M1T2 | 9.15 | 8491.86 |
| IF1_PSEMY_1 | A4XUY2 | 9.15 | 8302.55 |
| IF1_BLOPB_1 | Q492S0 | 9.77 | 8288.68 |
| IF1_SHEAM_1 | A1S7A4 | 9.6 | 8245.67 |
| IF1_RUBXD_1 | Q1AU52 | 8.11 | 8420.63 |
| IF1_CLOAB_1 | Q97EK1 | 6.56 | 8188.57 |
| IF1_CORGB_1 | A4QBQ3 | 9.15 | 8374.78 |
| IF12_STRAW_1 | Q82QK2 | 9.51 | 8673.22 |
| IF11_DESAG_1 | Q30V89 | 9.39 | 8143.49 |
| IF1_ACTPJ_1 | B0BQG0 | 8.93 | 8282.72 |
| IF11_SYMTH_1 | Q67QQ3 | 9.39 | 8736.04 |
| IF1_BACAH_1 | A0R8K3 | 6.72 | 8185.56 |
| IF1_CHLAA_1 | A9WH88 | 9.63 | 8538.03 |
| IF1_CHLCH_1 | Q3APJ5 | 9.3 | 8211.66 |
| IF1_PSE14_1 | Q48H67 | 9.15 | 8302.55 |
| IF1_STRMU_1 | Q8DS34 | 8.03 | 8244.64 |
| IF1_BORAP_1 | Q0SNZ5 | 9.48 | 8476.05 |
| IF1_ENT38_1 | A4W8Q8 | 9.22 | 8235.55 |
| IF1_BRADU_1 | Q89UH1 | 9.98 | 10838.44 |
| IF1_ERYLH_1 | Q2NCK2 | 8.93 | 9504.11 |
| IF1_SHEPA_1 | A8H5L4 | 9.6 | 8262.62 |
| IF1_FLAPJ_1 | A6GZ78 | 9.05 | 8092.51 |
| IF1_BRUSI_1 | B0CJM7 | 9.52 | 8371.88 |
| IF1_STRSV_1 | A3CK86 | 8.06 | 8202.52 |
| IF1_SODGM_1 | Q2NTZ7 | 9.22 | 8235.55 |
| IF1_GLUOX_1 | Q5FS72 | 9.69 | 8341.74 |
| IF1_RICBR_1 | Q1RK89 | 10.26 | 8285.73 |
| IF1_MESFL_1 | Q6F1X1 | 9 | 8888.47 |
| IF1_PASMU_1 | P57898 | 8.95 | 8194.56 |
| IF1_PECAS_1 | Q6D3U1 | 9.22 | 8235.55 |
| IF1_YERPE_1 | P65115 | 9.22 | 8235.55 |
| IF1_HAHCH_1 | Q2SJL1 | 9.69 | 8236.64 |
| IF1_ZYMMO_1 | Q5NNS2 | 9.22 | 8438.93 |
| IF1_CLAM3_1 | A5CU84 | 9.45 | 8377.83 |
| IF1_CLOB8_1 | A6LPT5 | 8.14 | 8182.57 |
| IF1_VIBPA_1 | P65129 | 9.22 | 8222.61 |
| IF12_METFK_1 | Q1H3X8 | 9.99 | 10053.75 |
| IF11_POLSJ_1 | Q12G82 | 9.6 | 8219.64 |
| IF1_BURTA_1 | Q2SU48 | 9.6 | 8217.67 |
| IF1_CALS4_1 | Q8R7X7 | 9.3 | 8142.54 |
| IF1_ANADF_1 | A7HDD6 | 9.34 | 8169.47 |
| IF1_CUTAK_1 | Q6A6Q6 | 9.98 | 8358.83 |
| IF1_BACVZ_1 | A7Z0R1 | 6.83 | 8213.57 |
| IF1_BARQU_1 | Q6FYU2 | 9.45 | 8425.9 |
| IF1_BAUCH_1 | Q1LTE4 | 9.52 | 8215.57 |
| IF1_LACDB_1 | Q04BZ3 | 8.05 | 8273.71 |
| IF1_ENTFA_1 | Q839E2 | 6.72 | 8236.62 |
| IF1_MYCVP_1 | A1T515 | 9.45 | 8488.93 |
| IF1_LACPL_1 | Q88XW4 | 8.27 | 8104.48 |
| IF1_LACS1_1 | Q1WSB2 | 6.83 | 8288.69 |
| IF1_LEGPA_1 | Q5X4E2 | 9.16 | 8341.72 |
| IF1_RHIEC_1 | Q2KCN5 | 9.52 | 8381.86 |
| IF1_NITOC_1 | Q3J7Z5 | 9.16 | 8231.56 |
| IF1_LISMO_1 | P65110 | 6.72 | 8217.56 |
| IF1_RICAH_1 | A8GQ12 | 9.98 | 8182.57 |
| IF1_MANSM_1 | Q65UU9 | 8.93 | 8325.75 |
| IF1_MARHV_1 | A1U1H0 | 9.69 | 8162.58 |
| IF1_YERE8_1 | A1JMD0 | 9.22 | 8235.55 |
| IF1_PHOPR_1 | Q6LT12 | 9.22 | 8219.55 |
| IF1_CHRSD_1 | Q1QUS2 | 9.15 | 8298.65 |
| IF1_TROW8_1 | Q83I58 | 9.52 | 8375.75 |
| IF1_VEREI_1 | A1WSA8 | 9.86 | 9233.62 |
| IF1_WOLSU_1 | Q7M8F3 | 9.21 | 8352.87 |
| IF1_ACTSZ_1 | A6VQL8 | 9.22 | 8280.69 |
| IF1_ARCB4_1 | A8ESW4 | 8.82 | 8233.72 |
| IF1_ARTS2_1 | A0JZ53 | 9.45 | 8477.99 |
| IF1_CHESB_1 | Q11LY3 | 9.52 | 8413.92 |
| IF1_PORGI_1 | Q7MTN5 | 9.57 | 8193.66 |
| IF1_KINRD_1 | A6W5W1 | 9.45 | 8433.89 |
| IF1_ACAM1_1 | B0C1F3 | 9.82 | 8506.94 |
| IF1_MYCPU_1 | Q98Q04 | 9.7 | 8344.89 |
| IF1_ACIAD_1 | Q6FEV4 | 9.15 | 8504.85 |
| IF1_PSELT_1 | A8F4T4 | 9.81 | 9851.39 |
| IF1_LACP3_1 | Q035A5 | 9.05 | 8147.55 |
| IF1_SHEON_1 | Q8EDW6 | 9.6 | 8272.7 |
| IF1_FLAJ1_1 | A5FN00 | 9.05 | 8108.51 |
| IF1_RENSM_1 | A9WSR5 | 8.07 | 8853.38 |
| IF1_NITEC_1 | Q0AIH5 | 9.6 | 8271.71 |
| IF1_RHILO_1 | Q989F0 | 9.52 | 8427.94 |
| IF1_SHIDS_1 | Q32E03 | 9.22 | 8249.58 |
| IF1_NITSB_1 | A6Q1J9 | 8.79 | 8241.79 |
| IF1_STRTD_1 | Q03IH3 | 8.06 | 8272.65 |
| IF1_PARL1_1 | A7HQG6 | 9.45 | 8343.8 |
| IF1_PEPD6_1 | Q18CI2 | 9.25 | 8281.75 |
| IF1_STAHJ_1 | Q4L892 | 6.72 | 8265.61 |
| IF1_TREPA_1 | O83135 | 9.59 | 8287.62 |
| IF1_UREPA_1 | Q9PQN8 | 9.22 | 8383.71 |
| IF1_AYWBP_1 | Q2NIX7 | 9.74 | 9081.62 |
| IF1_CAUVC_1 | Q9A5V4 | 9.45 | 8301.72 |
| IF1_DESPS_1 | Q6AP48 | 9.82 | 8932.53 |
| IF1_SALTI_1 | P69225 | 9.23 | 8118.38 |
| IF1_MYCSP_1 | P38037 | 9.58 | 10559.41 |
| IF1_STRS2_1 | A4VYR5 | 8.06 | 8226.56 |
| IF1_RHOJR_1 | Q0S3F2 | 9.45 | 8488.93 |
| IF1_SINMW_1 | A6U5Z0 | 9.52 | 8341.79 |
| IF1_XANC8_1 | Q4UUK1 | 9.39 | 8401.68 |
| IF1_GEOTN_1 | A4IJL1 | 6.83 | 8241.58 |
| IF1_RHORT_1 | Q2RWM9 | 9.98 | 10741.47 |
| IF1_SPHWW_1 | A5VDT7 | 9.15 | 8410.91 |
| IF1_MAGMM_1 | A0L5J6 | 9.3 | 9871.47 |
| IF1_MARMM_1 | Q0AQL4 | 9.1 | 8357.79 |
| IF1_HALHL_1 | A1WWV4 | 9.52 | 8341.72 |
| IF1_PELUB_1 | Q4FNE1 | 10.11 | 8042.44 |
| IF1_AGRFC_1 | Q8UHX4 | 9.52 | 8385.84 |
| IF12_CHRVO_1 | Q7NQH3 | 9.52 | 8192.66 |
| IF1_JANMA_1 | A6T3I3 | 9.6 | 8238.68 |
| IF1_SALCH_1 | Q57R48 | 9.22 | 8249.58 |
| IF1_SALPB_1 | A9N7Y7 | 9.22 | 8249.58 |
| IF1_ACIC1_1 | A0LRP3 | 9.77 | 8430.95 |
| IF1_BORBP_1 | Q662J5 | 9.65 | 8475.06 |
| IF1_MYCUA_1 | A0PMB2 | 9.45 | 8488.93 |
| IF1_PELTS_1 | A5D5E3 | 9.59 | 8299.77 |
| IF1_ROSDO_1 | Q169N4 | 9.45 | 8285.64 |
| IF1_THEP1_1 | A5IMA6 | 9.86 | 8535.06 |
| IF11_CUPMC_1 | Q1LLC3 | 9.15 | 8393.65 |
| IF1_BARHE_1 | Q6G252 | 9.45 | 8437.95 |
| IF1_PSEPF_1 | Q3KA77 | 9.15 | 8302.55 |
| IF1_SHEDO_1 | Q12N57 | 9.6 | 8258.67 |
| IF1_RHIL3_1 | Q1MLP3 | 9.52 | 8381.86 |
| IF1_NITEU_1 | Q820Q7 | 9.6 | 8271.71 |
| IF1_LISIN_1 | P65111 | 6.72 | 8217.56 |
| IF1_OCEIH_1 | Q8ETW2 | 6.82 | 8187.49 |
| IF1_GLOVI_1 | Q7NNJ8 | 9.69 | 8462.93 |
| IF1_SYNWW_1 | Q0AUK4 | 8.11 | 8314.8 |
| IF1_RICRS_1 | A8GTV5 | 9.98 | 8240.71 |
| IF1_HELAH_1 | Q17ZB8 | 9.51 | 8375.87 |
| IF1_STRA5_1 | P65127 | 8.06 | 8272.65 |
| IF1_CAMC1_1 | A7ZFZ1 | 8.76 | 8316.81 |
| IF1_AQUAE_1 | O66488 | 9.91 | 9195.88 |
| IF1_CAMFF_1 | A0RM32 | 8.76 | 8316.81 |
| IF1_BACAN_1 | Q81VQ7 | 6.72 | 8185.56 |
| IF12_AZOSB_1 | A1KB06 | 9.52 | 8253.71 |
| IF1_BACLD_1 | Q65P83 | 6.83 | 8199.54 |
| IF1_BACSK_1 | Q5WLN9 | 8.08 | 8269.68 |
| IF1_HERAR_1 | A4G9R7 | 9.6 | 8238.68 |
| IF1_MYCS5_1 | Q4A5I6 | 9.64 | 8237.68 |
| IF1_NEIG1_1 | Q5F5U8 | 9.22 | 8294.71 |
| IF1_PSYWF_1 | A5WGT1 | 9.3 | 8574.02 |
| IF1_RHIME_1 | Q92S23 | 9.52 | 8341.79 |
| IF1_ALKMQ_1 | A6TWF8 | 8.13 | 8210.59 |
| IF12_BORPA_1 | Q7WB25 | 9.97 | 10066.53 |
| IF1_CHLL7_1 | Q3B6E0 | 9.3 | 8239.72 |
| IF1_HYDCU_1 | Q31GL5 | 9.4 | 8334.71 |
| IF1_BACTN_1 | Q8A498 | 9.1 | 8234.62 |
| IF1_SALAI_1 | A8M505 | 9.69 | 8484.98 |
| IF1_JANSC_1 | Q28NC3 | 9.45 | 8329.75 |
| IF1_ACHLI_1 | A9NEF6 | 9.45 | 8221.6 |
| IF1_SERP5_1 | A8GCD9 | 9.22 | 8235.55 |
| IF1_SHISS_1 | Q3Z3N6 | 9.22 | 8249.58 |
| IF1_NOSS1_1 | Q8YPJ9 | 9.82 | 8565.02 |
| IF1_SPHAL_1 | Q1GRU4 | 9.15 | 8410.91 |
| IF1_SPIKU_1 | P61694 | 9.6 | 8166.7 |
| IF1_PARUW_1 | Q6MDN1 | 9.43 | 8240.78 |
| IF1_RICFE_1 | Q4UJZ6 | 9.86 | 8236.65 |
| IF1_SYNJB_1 | Q2JIK6 | 9.57 | 8492.91 |
| IF1_ROSCS_1 | A7NR41 | 9.81 | 8580.11 |
| IF1_RUEPO_1 | Q5LLF0 | 9.45 | 8285.64 |
| IF1_RUEST_1 | Q1GLE0 | 9.52 | 8313.65 |
| IF1_CLONN_1 | A0PXX0 | 8.16 | 8101.45 |
| IF1_WIGBR_1 | Q8D2W3 | 9.45 | 8360.85 |
| IF1_ALKOO_1 | A8MLG4 | 9.22 | 8175.61 |
| IF12_BORPE_1 | Q7VTB0 | 9.6 | 8263.69 |
| IF1_HYPNA_1 | Q0C2H2 | 9.1 | 8409.83 |
| IF1_BARBK_1 | A1URE4 | 9.45 | 8423.93 |
| IF1_BART1_1 | A9IXQ8 | 9.45 | 8411.87 |
| IF1_MAGSA_1 | Q2W1Y2 | 9.52 | 8338.68 |
| IF1_RICCK_1 | A8F030 | 9.98 | 8237.64 |
| IF1_RICCN_1 | Q92G58 | 9.98 | 8240.71 |
| IF1_PEDPA_1 | Q03ED8 | 8.02 | 8249.67 |
| IF1_CHLTE_1 | Q8KAJ3 | 9 | 8269.66 |
| IF1_STAS1_1 | Q49ZE6 | 6.72 | 8279.64 |
| IF1_TREDE_1 | P61696 | 9.69 | 8230.73 |
| IF1_VIBCB_1 | A7N1L7 | 9.22 | 8222.61 |
| IF1_ALCBS_1 | Q0VQ14 | 9.69 | 8367.9 |
| IF1_CROS8_1 | A7MEQ6 | 9.22 | 8249.58 |
| IF1_IDILO_1 | Q5R0C5 | 9.3 | 8201.53 |
| IF1_DESRM_1 | A4J134 | 9.86 | 8292.76 |
| IF1_STRGC_1 | A8AZK3 | 8.06 | 8216.54 |
| IF1_MYXXD_1 | Q1D753 | 9.52 | 8219.7 |
| IF1_LACRD_1 | A5VLI3 | 9.05 | 8161.64 |
| IF1_SHEPC_1 | A4Y7L4 | 9.6 | 8272.7 |
| IF1_FRAAA_1 | Q0RRP8 | 9.77 | 8542 |
| IF1_GEOMG_1 | Q39UK8 | 8.05 | 8172.56 |
| IF1_OCHA4_1 | A6WVQ0 | 9.52 | 8371.88 |
| IF1_LISW6_1 | A0ALU6 | 6.72 | 8217.56 |
| IF1_SYNFM_1 | A0LIL3 | 9.3 | 8306.68 |
| IF1_HELHP_1 | Q7VGC3 | 8.82 | 8231.75 |
| IF1_ROSS1_1 | A5USG7 | 9.81 | 8580.11 |
| IF1_XANAC_1 | Q8PL04 | 9.45 | 8375.65 |
| IF1_CORJK_1 | Q4JTB8 | 9.15 | 8374.78 |
| IF2 | Accession number | pI | MW |
| IF2_ECOLI_1 | P0A705 | 5.8 | 97349.9 |
| IF2_BACSU_1 | P17889 | 5.4 | 78621.6 |
| IF2_SALTY_1 | Q9ZF31 | 5.91 | 97402.21 |
| IF2_KLEOX_1 | Q9ZF28 | 5.81 | 98094.91 |
| IF2_ENTCL_1 | Q9ZF25 | 5.67 | 97892.61 |
| IF2_CHLPN_1 | Q9Z8M1 | 6.62 | 97105.07 |
| IF2_ENTFC_1 | P18311 | 7.23 | 86301.59 |
| IF2_PROVU_1 | Q9ZF22 | 5.87 | 101295.7 |
| IF2_STRA3_1 | P0A3K6 | 9.04 | 102401.2 |
| IF2_MYCTU_1 | P9WKK1 | 6.01 | 94041 |
| IF2_LISIN_1 | Q92C29 | 6.12 | 84977.28 |
| IF2_WIGBR_1 | Q8D2X6 | 9.76 | 94759.04 |
| IF2_LACLC_1 | Q9X764 | 7.73 | 104712.3 |
| IF2_GEOSE_1 | P04766 | 6.73 | 82061.46 |
| IF2_PSEAE_1 | Q9HV55 | 5.83 | 90912.47 |
| IF2_BLOFL_1 | Q7VQM3 | 8.27 | 100775 |
| IF2_BORA1_1 | Q2KXY7 | 5.45 | 109419.7 |
| IF2_HELPY_1 | P55972 | 7.03 | 105194.7 |
| IF2_CHLAD_1 | B8GAE2 | 8.74 | 80878.81 |
| IF2_ARTS2_1 | A0JUU0 | 6.55 | 99772.06 |
| IF2_FINM2_1 | B0S1E5 | 6.47 | 85138.53 |
| IF2_CYAP7_1 | B7KIU2 | 5.48 | 121652 |
| IF2_BRUAB_1 | Q57AA0 | 9.06 | 104140.8 |
| IF2_KLEP3_1 | B5XSX4 | 5.75 | 98073.69 |
| IF2_BACAA_1 | C3P5L5 | 5.03 | 75752.34 |
| IF2_CHLSY_1 | B9LBJ2 | 8.74 | 79371.21 |
| IF2_FRATH_1 | Q2A1G8 | 6.32 | 92422.06 |
| IF2_MAGMM_1 | A0LE19 | 6.07 | 103803 |
| IF2_BURCA_1 | Q1BWS7 | 5.95 | 104278.2 |
| IF2_BACCR_1 | Q812X7 | 5.03 | 75752.34 |
| IF2_CLOB6_1 | C3L0B6 | 4.9 | 75727.25 |
| IF2_PSEE4_1 | Q1IF43 | 6.42 | 91915.24 |
| IF2_RICPR_1 | Q9ZCZ8 | 8.03 | 91275 |
| IF2_RICRS_1 | A8GSP4 | 6.52 | 91073.54 |
| IF2_BURP1_1 | Q3JSY9 | 6.04 | 104764.7 |
| IF2_MYCA1_1 | A0QIY2 | 6.18 | 95466.4 |
| IF2_PSEP1_1 | A5W987 | 6.42 | 91504.91 |
| IF2_BURVG_1 | A4JDX1 | 5.95 | 104459.4 |
| IF2_LEGPA_1 | Q5X1C3 | 5.33 | 94699.24 |
| IF2_RUEST_1 | Q1GCH2 | 5.65 | 89511.92 |
| IF2_CAMC1_1 | A7ZC69 | 6.51 | 96941.25 |
| IF2_SACEN_1 | A4FM34 | 9.32 | 106676.5 |
| IF2_PSYIN_1 | A1ST45 | 7.21 | 95350.92 |
| IF2_RALSO_1 | Q8XZV6 | 6.07 | 103313.3 |
| IF2_CLOTE_1 | Q895J8 | 5.01 | 75657.3 |
| IF2_PELPD_1 | A1AMM1 | 8.47 | 98624.76 |
| IF2_CORA7_1 | C3PH19 | 6.76 | 94791.54 |
| IF2_ACICJ_1 | A5FV21 | 6.5 | 95356.5 |
| IF2_RHOBA_1 | Q7URR0 | 8.5 | 110114.7 |
| IF2_PERMH_1 | C0QTL9 | 5.46 | 98875.09 |
| IF2_RHOOB_1 | C1B313 | 8.66 | 100062.6 |
| IF2_STRS2_1 | A4W3R7 | 8.53 | 104075.3 |
| IF2_RHOP5_1 | Q07V78 | 8.92 | 95637.64 |
| IF2_VIBPA_1 | Q87M02 | 5.51 | 99396.14 |
| IF2_VIBTL_1 | B7VJH7 | 5.61 | 97524.98 |
| IF2_POLAQ_1 | A4SY78 | 5.75 | 98347.69 |
| IF2_SALTO_1 | A4X4N7 | 9.4 | 102374.3 |
| IF2_RHOS5_1 | A4WW80 | 6.31 | 90964 |
| IF2_STRZT_1 | C1CQ48 | 8.71 | 102907.3 |
| IF2_POLSJ_1 | Q12AU7 | 6.32 | 103487.1 |
| IF2_XANCB_1 | B0RRB4 | 8.37 | 97592.29 |
| IF2_BORPE_1 | Q7VYR2 | 5.55 | 106159.3 |
| IF2_AZOC5_1 | A8IG20 | 9.29 | 111349.7 |
| IF2_KINRD_1 | A6W7Z2 | 8.9 | 107925 |
| IF2_DECAR_1 | Q47D94 | 5.61 | 98102.98 |
| IF2_DESAH_1 | C0QHM2 | 8.92 | 111558.1 |
| IF2_LACDA_1 | Q1G9P9 | 9.23 | 90866.22 |
| IF2_BACCN_1 | A7GRE3 | 5.03 | 76010.68 |
| IF2_NITSB_1 | A6Q226 | 5.76 | 93414.64 |
| IF2_CLAM3_1 | A5CSZ4 | 5.63 | 98306.51 |
| IF2_DINSH_1 | A8LQ56 | 5.57 | 89918.33 |
| IF2_OCHA4_1 | A6WWW5 | 9.15 | 104399.1 |
| IF2_OENOB_1 | Q04GN0 | 9.42 | 90524.11 |
| IF2_MYCBT_1 | C1AFV3 | 6.01 | 94041 |
| IF2_CAMJE_1 | Q9PIZ1 | 5.57 | 96125.98 |
| IF2_CLOPE_1 | Q8XJR8 | 4.94 | 74160.55 |
| IF2_MYCPU_1 | Q98R05 | 8.5 | 67855.01 |
| IF2_LISMH_1 | B8DG02 | 6.09 | 84810.07 |
| IF2_PHEZH_1 | B4RC55 | 9.15 | 105814.5 |
| IF2_PHOLL_1 | Q7MYY7 | 5.84 | 99902.82 |
| IF2_SALPC_1 | C0PZ54 | 5.91 | 97402.21 |
| IF2_STAA1_1 | A7X1Q1 | 5.09 | 77856.69 |
| IF2_MYCVP_1 | A1T7H8 | 6.18 | 96071.87 |
| IF2_VIBVU_1 | Q8DBW0 | 5.43 | 99590.38 |
| IF2_SYNJA_1 | Q2JSB7 | 6.55 | 111749.1 |
| IF2_GEOTN_1 | A4IMD7 | 6.47 | 81495.85 |
| IF2_GLOVI_1 | Q7NH85 | 5.27 | 98909.54 |
| IF2_GLUOX_1 | Q5FQM3 | 8.36 | 98526.4 |
| IF2_SHESA_1 | A0KTZ6 | 5.82 | 96508.58 |
| IF2_YERPA_1 | Q1CC07 | 5.86 | 96653.59 |
| IF2_NOCSJ_1 | A1SLK7 | 5.42 | 98290.87 |
| IF2_BACHD_1 | Q9KA77 | 5.51 | 79993.77 |
| IF2_BURM9_1 | A2S2L1 | 6.04 | 104750.6 |
| IF2_BACP2_1 | A8FDD1 | 5.35 | 77573.42 |
| IF2_PSEFS_1 | C3K259 | 6.61 | 90598.93 |
| IF2_CAMFF_1 | A0RQZ4 | 5.48 | 91823.79 |
| IF2_MYCLE_1 | Q9Z5I9 | 5.84 | 96650.79 |
| IF2_CARHZ_1 | Q3AB98 | 8.97 | 92574.18 |
| IF2_RHIL3_1 | Q1MN39 | 7.3 | 99536.58 |
| IF2_STRT1_1 | Q5M1B9 | 9.14 | 103827 |
| IF2_SALSV_1 | B4TWD8 | 5.91 | 97414.26 |
| IF2_SALTI_1 | Q8Z3H7 | 5.91 | 97444.29 |
| IF2_POLNA_1 | A1VNU2 | 8.48 | 105593.9 |
| IF2_SERP5_1 | A8G907 | 5.85 | 97586.48 |
| IF2_XANAC_1 | Q8PJ55 | 7.01 | 96180.67 |
| IF2_CHESB_1 | Q11BC8 | 6.3 | 94135.64 |
| IF2_COXBU_1 | Q83BS1 | 6.48 | 88485.57 |
| IF2_ANAVT_1 | Q3MBZ7 | 6.06 | 111596.9 |
| IF2_HERAR_1 | A4G7S7 | 6.44 | 101593.3 |
| IF2_BORBU_1 | O51741 | 9.26 | 97795.66 |
| IF2_PROMT_1 | Q46J13 | 9.85 | 128121 |
| IF2_LACF3_1 | B2GBN7 | 8.69 | 84640.7 |
| IF2_PSEA6_1 | Q15V72 | 5.45 | 94942.66 |
| IF2_MARMM_1 | Q0AK69 | 6.37 | 93250.22 |
| IF2_DESVV_1 | A1VG83 | 8.03 | 115420.3 |
| IF2_METFK_1 | Q1GXD6 | 5.47 | 98031.74 |
| IF2_METPB_1 | B1ZDQ8 | 8.47 | 106294.3 |
| IF2_ROSCS_1 | A7NID1 | 8.39 | 78189.65 |
| IF2_AMOA5_1 | B3EUG6 | 5.61 | 100503.1 |
| IF2_BORBP_1 | Q65ZX2 | 9.27 | 98162.06 |
| IF2_CHLCH_1 | Q3AQK7 | 5.31 | 111718.7 |
| IF2_HYDCU_1 | Q31GK5 | 5.81 | 89602.61 |
| IF2_AZOPC_1 | B6YQ44 | 9.27 | 103019.8 |
| IF2_AZOVD_1 | C1DFK9 | 5.95 | 90863.29 |
| IF2_CHLPM_1 | A4SGG2 | 5.21 | 98878.58 |
| IF2_FRASN_1 | A8L6F4 | 9.62 | 106406.7 |
| IF2_LACBA_1 | Q03QT5 | 6.36 | 85857.51 |
| IF2_CHLTR_1 | O84098 | 8.21 | 97009.74 |
| IF2_CHRVO_1 | Q7NY13 | 7.31 | 103104.5 |
| IF2_BACFN_1 | Q5LIN1 | 6.46 | 112630.5 |
| IF2_BURCM_1 | Q0BFY3 | 6.06 | 104324.3 |
| IF2_BACSK_1 | Q5WFU2 | 5.96 | 82736.05 |
| IF2_MICLC_1 | C5C9T1 | 6.55 | 97153.34 |
| IF2_BARBK_1 | A1UU50 | 7.35 | 93749.74 |
| IF2_PSEU5_1 | A4VPP0 | 6 | 90460.05 |
| IF2_LEPBP_1 | B0SQH4 | 8.71 | 97207.83 |
| IF2_PELPB_1 | B4SCE7 | 5.35 | 109121.3 |
| IF2_BIFLD_1 | B3DQF0 | 9.11 | 104024.4 |
| IF2_MYCMS_1 | Q6MTQ0 | 6.09 | 68802.36 |
| IF2_ANAMM_1 | Q5PAJ5 | 6.59 | 88755.05 |
| IF2_ENT38_1 | A4WEY3 | 5.79 | 97984.71 |
| IF2_AROAE_1 | Q5NZS1 | 5.54 | 102381.4 |
| IF2_CHLMU_1 | Q9PKU0 | 7.59 | 97498.04 |
| IF2_DEHMB_1 | A5FQR6 | 5.84 | 64079.92 |
| IF2_CHLT3_1 | B3QUN2 | 5.15 | 122673.4 |
| IF2_LACCB_1 | B3WER5 | 9.46 | 103151.1 |
| IF2_BUCAT_1 | B8D7R4 | 9.38 | 97504.38 |
| IF2_NITOC_1 | Q3J9B6 | 7.3 | 92578.42 |
| IF2_LACH4_1 | A8YVQ7 | 9.57 | 97279.04 |
| IF2_LACP7_1 | A9KNW4 | 8.37 | 123782.3 |
| IF2_ACIAC_1 | A1TSK3 | 6.02 | 101154.5 |
| IF2_PARXL_1 | Q140U6 | 6.12 | 106048.2 |
| IF2_PASMU_1 | P57873 | 6.32 | 90862.42 |
| IF2_BDEBA_1 | Q6MMS6 | 8.42 | 103244.7 |
| IF2_PECCP_1 | C6DKK3 | 5.83 | 98571.44 |
| IF2_PSYCK_1 | Q1QEP5 | 6.04 | 99947.35 |
| IF2_BIFAA_1 | A1A0A2 | 9.12 | 99003.38 |
| IF2_MYCMO_1 | Q6KID8 | 6.05 | 66505.4 |
| IF2_PELUB_1 | Q4FNM9 | 9.01 | 79862.41 |
| IF2_SINMW_1 | A6UF29 | 6.57 | 96135.66 |
| IF2_SODGM_1 | Q2NW23 | 5.87 | 97841.27 |
| IF2_AERS4_1 | A4SJR5 | 5.65 | 98376.4 |
| IF2_ALIFM_1 | B5FA79 | 5.6 | 97335.94 |
| IF2_NEIM0_1 | A9M1D5 | 8.4 | 103138.5 |
| IF2_ALKEH_1 | Q0A797 | 5.27 | 96385.64 |
| IF2_ALKMQ_1 | A6TRK7 | 5.22 | 76792.77 |
| IF2_PROA2_1 | B4S4S6 | 5.6 | 104102.6 |
| IF2_SYNFM_1 | A0LHL8 | 5.41 | 106191 |
| IF2_XANOP_1 | B2SVK3 | 6.81 | 96197.54 |
| IF2_XYLFA_1 | Q9PGR3 | 7.12 | 97010.04 |
| IF2_PROMH_1 | B4F2B9 | 5.89 | 101072.4 |
| IF2_YERPB_1 | B2K2Q5 | 5.75 | 97567.49 |
| IF2_SYNWW_1 | Q0AYI8 | 8.66 | 97143.36 |
| IF2_HAEIE_1 | A5UBT6 | 6.03 | 91631.26 |
| IF2_SHEWM_1 | B1KRR0 | 5.64 | 95998.64 |
| IF2_THET2_1 | Q72KE8 | 5.27 | 63178.41 |
| IF2_THISH_1 | B8GP02 | 5.71 | 91668.15 |
| IF2_TRIEI_1 | Q10XM3 | 6.4 | 116623.5 |
| IF2_STRPC_1 | Q1JKH1 | 9.19 | 105536.4 |
| IF2_UREU1_1 | B5ZBC9 | 5.69 | 67760.09 |
| IF2_FERNB_1 | A7HN01 | 5.44 | 77128.1 |
| IF2_AYWBP_1 | Q2NIQ6 | 8.9 | 69163.03 |
| IF2_BRASB_1 | A5E866 | 9.17 | 98696.87 |
| IF2_FLAPJ_1 | A6GWV8 | 7.26 | 105716.9 |
| IF2_CHLPB_1 | B3ELN8 | 5.31 | 101322.3 |
| IF2_BRUME_1 | Q8YEB3 | 9 | 104121.8 |
| IF2_KOSOT_1 | C5CDZ4 | 5.61 | 77427.2 |
| IF2_MARMS_1 | A6VU29 | 5.81 | 93292.17 |
| IF2_MESFL_1 | Q6F1H1 | 5.4 | 68990.07 |
| IF2_NOVAD_1 | Q2G5E7 | 6.52 | 93569.93 |
| IF2_BURM1_1 | A9ABD5 | 6.03 | 103899.9 |
| IF2_PSELT_1 | A8F5A0 | 5.48 | 75905.73 |
| IF2_LACS1_1 | Q1WUF4 | 7.11 | 82194.39 |
| IF2_ROSDO_1 | Q16D38 | 5.69 | 88505.97 |
| IF2_CALS4_1 | Q8RA37 | 5.36 | 78856.9 |
| IF2_BAUCH_1 | Q1LSK8 | 9.02 | 97834.11 |
| IF2_MYCH7_1 | Q4A7E2 | 8.77 | 66776.17 |
| IF2_LEPIC_1 | Q72NX3 | 9.01 | 92993.75 |
| IF2_LEUCK_1 | B1MZH4 | 9.18 | 91144.88 |
| IF2_MYCPA_1 | Q73VV4 | 6.22 | 96525.54 |
| IF2_CORDI_1 | Q6NGN2 | 9.24 | 99597.43 |
| IF2_MYCS5_1 | Q4A578 | 7.19 | 67225.03 |
| IF2_RHOJR_1 | Q0S219 | 8.66 | 100669.4 |
| IF2_VIBC3_1 | A5F926 | 5.59 | 98705.51 |
| IF2_AERHH_1 | A0KNE3 | 5.65 | 98170.22 |
| IF2_STAS1_1 | Q49X54 | 5.01 | 77470.44 |
| IF2_THEFY_1 | Q47RV1 | 9.34 | 100541.5 |
| IF2_HALOH_1 | B8CW72 | 5.77 | 75987.18 |
| IF2_SHIF8_1 | Q0T0B3 | 5.9 | 96436 |
| IF2_TREPA_1 | O83861 | 9.3 | 90924.99 |
| IF2_ANAPZ_1 | Q2GKQ2 | 6.29 | 88766.93 |
| IF2_BORBR_1 | Q7WHG2 | 5.63 | 106165.4 |
| IF2_CUPNH_1 | Q0K9B9 | 5.84 | 103710.4 |
| IF2_CHLL2_1 | B3EFB1 | 5.32 | 105919.7 |
| IF2_JANMA_1 | A6T0Y8 | 6.32 | 100981.3 |
| IF2_BRUO2_1 | A5VTB2 | 8.96 | 104099.8 |
| IF2_MACCJ_1 | B9EBE9 | 5.22 | 79856.82 |
| IF2_DESHY_1 | Q24UI6 | 9.41 | 106887.6 |
| IF2_DESRM_1 | A4J5X2 | 9.2 | 107321.1 |
| IF2_PSEMY_1 | A4XYE0 | 5.97 | 89825.49 |
| IF2_PARDP_1 | A1B587 | 6.04 | 91294.13 |
| IF2_LARHH_1 | C1D8X2 | 7.06 | 102235.5 |
| IF2_MYCCT_1 | Q2SSE6 | 6.19 | 68659.18 |
| IF2_CAMHC_1 | A7I3V0 | 8.95 | 100601.1 |
| IF2_CLOK5_1 | A5N842 | 5.17 | 76852.81 |
| IF2_ORITB_1 | A5CEN6 | 8.78 | 93392.58 |
| IF2_LEPBL_1 | Q04ZJ5 | 8.99 | 92440.22 |
| IF2_BEUC1_1 | C5BWS3 | 5.54 | 98103.74 |
| IF2_ACIB5_1 | B7I3R9 | 6.51 | 97314.55 |
| IF2_EDWI9_1 | C5BFB7 | 5.71 | 98215.95 |
| IF2_STRSV_1 | A3CQ18 | 8.9 | 102360.4 |
| IF2_RHORT_1 | Q2RMS0 | 6.43 | 93793.84 |
| IF2_NEIG1_1 | Q5F797 | 9.01 | 101018.4 |
| IF2_WOLPM_1 | Q73FL0 | 6.78 | 86182 |
| IF2_WOLTR_1 | Q5GS99 | 7.93 | 86324.29 |
| IF2_RICB8_1 | A8GW31 | 6.24 | 90860.9 |
| IF2_GEOKA_1 | Q5L0I8 | 8.27 | 78241.31 |
| IF2_SHEDO_1 | Q12QI1 | 6.01 | 95784.1 |
| IF2_SHELP_1 | A3QGU5 | 5.81 | 95158.95 |
| IF2_SHEPC_1 | A4Y9C0 | 5.74 | 95515.5 |
| IF2_YERE8_1 | A1JIW9 | 5.84 | 97336.26 |
| IF2_SYNY3_1 | P72689 | 5.73 | 108118.8 |
| IF2_THEP3_1 | B0K9Q0 | 5.16 | 76822.63 |
| IF2_UREP2_1 | B1AIV8 | 5.77 | 68048.52 |
| IF2_ANADE_1 | Q2IPZ7 | 7.28 | 100890.5 |
| IF2_CHLAB_1 | Q5L627 | 8.37 | 95618.5 |
| IF2_BORPD_1 | A9ITX6 | 5.49 | 104344.2 |
| IF2_CHLL7_1 | Q3B1Z8 | 5.38 | 99922.36 |
| IF2_FLAJ1_1 | A5FJF9 | 6.35 | 104573.2 |
| IF2_FRAAA_1 | Q0RDS4 | 9.56 | 103534.6 |
| IF2_DEIGD_1 | Q1IZ02 | 5.08 | 64583.36 |
| IF2_BRUSU_1 | Q8FXT2 | 9 | 104198.9 |
| IF2_DESAG_1 | Q30WJ0 | 8.64 | 105451.6 |
| IF2_CHLTE_1 | Q8KFT1 | 5.56 | 100647.3 |
| IF2_OCEIH_1 | Q8EQU1 | 5.23 | 76147.3 |
| IF2_ONYPE_1 | Q6YR66 | 8.59 | 68496.04 |
| IF2_PSEHT_1 | Q3IJ53 | 6.09 | 96673.72 |
| IF2_SACD2_1 | Q21H61 | 5.15 | 97952.32 |
| IF2_PSEU2_1 | Q4ZNR2 | 6.48 | 90538.96 |
| IF2_PECAS_1 | Q6D9A5 | 5.88 | 98924.87 |
| IF2_CLONN_1 | A0Q0Q7 | 5 | 75059.78 |
| IF2_LEPCP_1 | B1XY67 | 6.41 | 104938.4 |
| IF2_BORAP_1 | Q0SM50 | 9.09 | 86405.03 |
| IF2_BORPA_1 | Q7W9A5 | 5.79 | 103544.5 |
| IF2_HYPNA_1 | Q0C5Z5 | 6.35 | 91692.49 |
| IF2_BRADU_1 | Q89WA9 | 8.93 | 96916.95 |
| IF2_JANSC_1 | Q28WF7 | 5.37 | 89054.25 |
| IF2_CYTH3_1 | Q11PK5 | 6.02 | 108860.2 |
| IF2_AZOSB_1 | A1K7B9 | 5.39 | 100828.7 |
| IF2_NITEU_1 | Q82WD0 | 6.58 | 96986.55 |
| IF2_NITHX_1 | Q1QS64 | 8.72 | 93501.4 |
| IF2_FUSNN_1 | Q8R5Z1 | 5.46 | 81757.78 |
| IF2_NOCFA_1 | Q5YSC6 | 9.02 | 100739.3 |
| IF2_RUEPO_1 | Q5LWL4 | 5.58 | 90199.55 |
| IF2_CALS8_1 | A4XL70 | 6.43 | 96947.34 |
| IF2_PARUW_1 | Q6MD64 | 7.27 | 101783.2 |
| IF2_MYCGA_1 | Q7NBZ4 | 6.6 | 68086.17 |
| IF2_BART1_1 | A9IMT5 | 7.34 | 93356.38 |
| IF2_BEII9_1 | B2IIJ7 | 9.21 | 111002.8 |
| IF2_PEDPA_1 | Q03FS3 | 9.27 | 101974 |
| IF2_SALAR_1 | A9MP36 | 5.91 | 97417.18 |
| IF2_RENSM_1 | A9WPV8 | 6.41 | 98745.97 |
| IF2_CLOTH_1 | A3DE44 | 7.29 | 114955.6 |
| IF2_EHRCJ_1 | Q3YS01 | 5.67 | 93816.04 |
| IF2_PELTS_1 | A5D2S0 | 9.2 | 107814.2 |
| IF2_COREF_1 | Q8FPA7 | 8.85 | 100081.2 |
| IF2_RHOE4_1 | C0ZYA5 | 8.95 | 100446.2 |
| IF2_ACTP7_1 | B3H163 | 5.95 | 91495.17 |
| IF2_SALHS_1 | B4TJ06 | 5.91 | 97402.21 |
| IF2_MYCSS_1 | Q1BA94 | 6.43 | 95438.51 |
| IF2_VIBCB_1 | A7MZI5 | 5.61 | 97855.55 |
| IF2_STRS7_1 | C0MDY5 | 9.26 | 105256.7 |
| IF2_NATTJ_1 | B2A397 | 4.68 | 74857.26 |
| IF2_SHEAM_1 | A1S462 | 5.93 | 95960.19 |
| IF2_PORG3_1 | B2RHM9 | 6.16 | 107544.3 |
| IF2_SHEB8_1 | A6WRG8 | 5.77 | 95542.55 |
| IF2_SYNE7_1 | Q31LL9 | 6.26 | 110640.9 |
| IF2_GEOSL_1 | Q74CT3 | 6.14 | 95693.18 |
| IF2_STACT_1 | B9DPF5 | 5.02 | 78174.09 |
| IF2_SHEHH_1 | B0TQA2 | 5.77 | 95663.42 |
| IF2_SHEON_1 | Q8EHL5 | 5.93 | 96113.34 |
| IF2_STRCO_1 | Q8CJQ8 | 9.47 | 105657.7 |
| IF2_HELHP_1 | Q7VHF6 | 6.25 | 97443.01 |
| IF2_THIDA_1 | Q3SKX1 | 5.94 | 97800.99 |
| IF2_TOLAT_1 | C4L8X4 | 5.87 | 100014 |
| IF2_ANADF_1 | A7H9F3 | 6.35 | 102676.4 |
| IF2_ENTFA_1 | Q835U8 | 6.8 | 88147.03 |
| IF2_ANOFW_1 | B7GG75 | 5.98 | 80796.17 |
| IF2_HISS2_1 | B0UU13 | 6.46 | 91334.02 |
| IF2_CUPTR_1 | B3R1E4 | 5.9 | 103804.3 |
| IF2_CHLP8_1 | B3QQI2 | 5.45 | 103258.2 |
| IF2_FRACC_1 | Q2J728 | 9.69 | 107742.3 |
| IF2_NITMU_1 | Q2Y7W2 | 6.56 | 95075.42 |
| IF2_MAGSA_1 | Q2VZV0 | 5.77 | 93829.76 |
| IF2_PSE14_1 | Q48E77 | 6.48 | 90796.3 |
| IF2_MARHV_1 | A1U600 | 5.21 | 92212.8 |
| IF2_LACJO_1 | Q74IS8 | 9.3 | 98662.39 |
| IF2_CLOAB_1 | Q97I51 | 5.16 | 76337.27 |
| IF2_MOOTA_1 | Q2RJM5 | 8.88 | 98207.71 |
| IF2_CAMC5_1 | A7GZZ3 | 6.85 | 99188.78 |
| IF2_SALAI_1 | A8M746 | 9.41 | 102114.2 |
| IF2_RHILO_1 | Q98BI8 | 6.35 | 93870.9 |
| IF2_ACISJ_1 | A1W8Z4 | 5.96 | 101832.2 |
| IF2_CORJK_1 | Q4JV51 | 8.99 | 96527.5 |
| IF2_STRU0_1 | B9DVB7 | 9.04 | 105493.4 |
| IF2_WOLSU_1 | Q7M7X5 | 4.96 | 102670.6 |
| IF2_POLNS_1 | B1XV89 | 6.28 | 97961.92 |
| IF2_SYMTH_1 | Q67P86 | 9.27 | 110586 |
| IF2_SYNAS_1 | Q2LWU6 | 8.77 | 101214.1 |
| IF2_SHEFN_1 | Q086H2 | 6.07 | 95938.24 |
| IF2_GRAFK_1 | A0LXQ1 | 5.85 | 104312.2 |
| IF2_SHIBS_1 | Q31W47 | 5.9 | 96407.94 |
| IF2_THEMA_1 | Q9WZN3 | 5.44 | 77773.84 |
| IF2_THEPX_1 | B0K1D6 | 5.15 | 77133.06 |
| IF2_STRMU_1 | Q8DVP9 | 8.97 | 101538.5 |
| IF2_EHRRW_1 | Q5HB61 | 5.83 | 94986.45 |
| IF2_MICAN_1 | B0JU67 | 5.65 | 109509.9 |
| IF2_RICTY_1 | Q68WI4 | 7.17 | 91281.96 |
| IF2_PARD8_1 | A6LHS1 | 6.28 | 108317.5 |
| IF2_MYCA9_1 | B1MD87 | 6.02 | 94772.79 |
| IF2_PARPJ_1 | B2T381 | 6.18 | 105633.8 |
| IF2_RALPJ_1 | B2UAA3 | 5.92 | 103458.3 |
| IF2_CAUSK_1 | B0T167 | 9.3 | 110522 |
| IF2_ACIC1_1 | A0LV27 | 6.64 | 92534.22 |
| IF2_ACIC5_1 | C1F697 | 9.45 | 111261.2 |
| IF2_RHIME_1 | Q92SW4 | 6.99 | 96655.13 |
| IF2_CORGL_1 | Q8NP40 | 9.1 | 103412.6 |
| IF2_RHOFT_1 | Q21WJ5 | 7.69 | 105183.2 |
| IF2_SALNS_1 | B4T6Z8 | 5.91 | 97402.21 |
| IF2_SALRD_1 | Q2S1N7 | 4.28 | 112063.4 |
| IF2_MYXXD_1 | Q1DAM6 | 9.22 | 111900.2 |
| IF2_SULDN_1 | Q30SS6 | 5.59 | 95934.78 |
| IF2_ALKOO_1 | A8MFA8 | 5.25 | 78336.39 |
| IF2_STAHJ_1 | Q4L5X1 | 5.07 | 78730.57 |
| IF2_STIAU_1 | P55875 | 9.12 | 111323.6 |
| IF2_SHEPW_1 | B8CKH3 | 5.83 | 96531.07 |
| IF2_SHESH_1 | A8FYS0 | 5.9 | 96154.94 |
| IF2_STRAW_1 | Q82K53 | 9.42 | 106883.1 |
| IF2_STRGC_1 | A8AVQ2 | 9 | 105035.2 |
| IF2_HALHL_1 | A1WXV1 | 5.03 | 97161.63 |
| IF2_THEP1_1 | A5IJ09 | 5.34 | 78265.44 |
| IF2_THESQ_1 | B1L7T1 | 5.37 | 77893.98 |
| IF2_THEYD_1 | B5YHT8 | 6.41 | 83755 |
| IF2_TROW8_1 | Q83HG7 | 6.53 | 86452.86 |
| IF2_VARPS_1 | C5CLW3 | 6.12 | 104802.9 |
| IF2_BLOPB_1 | Q493T7 | 9.13 | 99368.08 |
| IF2_CUPMC_1 | Q1LLR6 | 5.73 | 104941.4 |
| IF2_ARCB4_1 | A8EWD7 | 5.27 | 97262.08 |
| IF2_CUTAK_1 | Q6A7M5 | 8.71 | 101393.7 |
| IF2_DEIRA_1 | Q9RTG5 | 4.87 | 64023.53 |
| IF2_LISW6_1 | A0AIC6 | 6.09 | 84718.96 |
| IF2_DESOH_1 | A8ZZ65 | 5.34 | 95548.6 |
| IF2_DESPS_1 | Q6AJY4 | 6.29 | 101215 |
| IF2_LACGA_1 | Q044B7 | 9.41 | 98853.82 |
| IF2_METCA_1 | Q609C0 | 6.05 | 94854.29 |
| IF2_METPP_1 | A2SH40 | 5.92 | 101248.3 |
| IF2_METSB_1 | B8EIA7 | 7.76 | 95766.92 |
| IF2_BACV8_1 | A6L030 | 6.43 | 111108.6 |
| IF2_BURTA_1 | Q2SVG8 | 5.98 | 104993.8 |
| IF2_PARP8_1 | B2JKT4 | 6.18 | 103848.8 |
| IF2_ACIAD_1 | Q6FF40 | 6.84 | 98115.5 |
| IF2_LEIXX_1 | Q6AG49 | 5.67 | 95932.85 |
| IF2_SALEP_1 | B5QZV8 | 5.91 | 97402.21 |
| IF2_PETMO_1 | A9BJ54 | 5.39 | 78578.52 |
| IF2_SPHAL_1 | Q1GVI9 | 6.51 | 91055.6 |
| IF2_MYCUA_1 | A0PQC4 | 6.51 | 97995.23 |
| IF2_ALISL_1 | B6ENE2 | 5.91 | 97549.38 |
| IF2_RICCK_1 | A8EYF4 | 8.6 | 91534.45 |
| IF2_RICFE_1 | Q4UL51 | 6.35 | 90737.86 |
| IF2_GEOSM_1 | C6E2Q0 | 8.02 | 104584.1 |
| IF2_STAEQ_1 | Q5HPS2 | 5.09 | 79343.46 |
| IF2_SHEPA_1 | A8H740 | 5.71 | 96150.84 |
| IF2_HAEDU_1 | Q7VLI2 | 7.15 | 91847.01 |
| IF2_THEAB_1 | B7IF03 | 5.22 | 78609.58 |
| IF2_HAES1_1 | Q0I3P5 | 6.46 | 91336 |
| IF2_THEM4_1 | A6LP48 | 5.2 | 78185.19 |
| IF2_HELAH_1 | Q17WQ8 | 7.26 | 105210 |
| IF2_SHISS_1 | Q3YX73 | 5.78 | 97330.85 |
| IF2_STRMK_1 | B2FN90 | 6.71 | 94768.21 |
| IF2_VESOH_1 | A5CXX6 | 8.09 | 89128.81 |
| IF2_CELJU_1 | B3PI96 | 5.48 | 100714.7 |
| IF2_BRUC2_1 | A9M9Z4 | 9.12 | 104109.8 |
| IF2_FRAP2_1 | B0TWR3 | 7.96 | 91762.16 |
| IF2_DEIDV_1 | C1CUQ9 | 5.03 | 65073.97 |
| IF2_KORVE_1 | Q1IIT3 | 9.36 | 106829.6 |
| IF2_DESAA_1 | B8FCY5 | 5.51 | 111921.6 |
| IF2_MANSM_1 | Q65SK9 | 5.98 | 89537.95 |
| IF2_NOSS1_1 | Q8YQJ1 | 6.06 | 111595.8 |
| IF2_BACHK_1 | Q6HF02 | 5.03 | 75724.29 |
| IF2_LACP3_1 | Q038M5 | 9.46 | 103123.1 |
| IF2_LACSS_1 | Q38W81 | 9.51 | 103215.5 |
| IF2_ROSS1_1 | A5UZQ2 | 8.66 | 77422.89 |
| IF2_RUBXD_1 | Q1AW55 | 5.08 | 74403.9 |
| IF2_SALA4_1 | B5F6T8 | 5.91 | 97402.21 |
| IF2_PSYA2_1 | Q4FVL5 | 6 | 99875.19 |
| IF2_PSYWF_1 | A5WBS5 | 6.01 | 99297.84 |
| IF2_MYCMM_1 | B2HKS2 | 6.41 | 98161.49 |
| IF2_SALDC_1 | B5FI13 | 5.91 | 97402.21 |
| IF2_MYCPE_1 | Q8EWU0 | 7.6 | 68954.4 |
| IF2_PEPD6_1 | Q18BH4 | 4.76 | 69710.86 |
| IF2_PHOPR_1 | Q6LUJ2 | 5.99 | 98470.39 |
| IF2_ALTMD_1 | B4RXT8 | 5.4 | 95580.15 |
| IF2_CROS8_1 | A7MQE1 | 5.84 | 98677.43 |
| IF2_CHLFF_1 | Q254H4 | 8.74 | 96155.43 |
| IF2_NITWN_1 | Q3SWP9 | 9.06 | 93664.62 |
| IF2_GEOBB_1 | B5EI57 | 8.44 | 105095.6 |
| IF2_AQUAE_1 | O67825 | 5.32 | 91600.45 |
| IF2_ESCF3_1 | B7LR37 | 5.8 | 97312.85 |
| IF2_IDILO_1 | Q5QTY8 | 5.45 | 99177.89 |
| IF2_KOCRD_1 | B2GKR5 | 7.73 | 100648 |
| IF2_DICNV_1 | A5EWY9 | 6.08 | 97945.97 |
| IF2_PSECP_1 | B8HG54 | 6.41 | 98634.85 |
| IF2_BACLD_1 | Q65JI1 | 5.43 | 78577.6 |
| IF2_LACRD_1 | A5VJE0 | 7.8 | 83537.56 |
| IF2_PARL1_1 | A7HZ93 | 6.52 | 95736.69 |
| IF2_BACWK_1 | A9VT50 | 5.05 | 75718.37 |
| IF2_BARQU_1 | Q6G0P2 | 8.53 | 93497.63 |
| IF2_CLOCE_1 | B8I6E7 | 8.85 | 130124.9 |
| IF2_RHIEC_1 | Q2KDZ5 | 7.75 | 99078.12 |
| IF2_LEUMM_1 | Q03WH4 | 9.13 | 90378.93 |
| IF2_ACTSZ_1 | A6VNE0 | 6.32 | 91539.96 |
| IF2_SPHWW_1 | A5VCZ5 | 6 | 91944.14 |
| IF2_AGRVS_1 | B9JYK6 | 7.31 | 100385.6 |
| IF2_RICAH_1 | A8GNW1 | 6.71 | 90893.25 |
| IF2_GEODF_1 | B9M1G0 | 8.64 | 98704.61 |
| IF2_GEOLS_1 | B3EAE7 | 8.04 | 101298.1 |
| IF2_GEOUR_1 | A5GF86 | 8.09 | 96150.2 |
| IF2_TERTT_1 | C5BPV9 | 4.96 | 101496.4 |
| IF2_THEEB_1 | Q8DK04 | 6.55 | 104247.2 |
| IF2_HAHCH_1 | Q2SML3 | 5.19 | 93752.08 |
| IF2_ZYMMO_1 | Q5NQ27 | 9.37 | 106620.5 |
| IF2_SHIDS_1 | Q32BG5 | 5.8 | 97379.92 |
| IF2_HAMD5_1 | C4K3F0 | 8.8 | 97372.32 |
| IF2_TREDE_1 | Q73NP6 | 8.64 | 98140.59 |
| IF2_VEREI_1 | A1WLI3 | 8.13 | 105143.5 |
| IF2_CAUVC_1 | Q9AC25 | 9.35 | 107459.6 |
| IF2_ERYLH_1 | Q2NC10 | 5.82 | 90666.82 |
| IF2_CHLCV_1 | Q823F2 | 8.72 | 96938.3 |
| IF2_EUBE2_1 | C4Z5N7 | 6.53 | 103788.5 |
| IF2_LACAC_1 | Q5FJN6 | 9.47 | 98133.81 |
| IF2_LYSSC_1 | B1HR05 | 6.13 | 83263.57 |
| IF2_DESDA_1 | B8J1Y4 | 6.96 | 106103 |
| IF2_CHRSD_1 | Q1QSZ0 | 5.54 | 91352.06 |
| IF2_NOSP7_1 | B2J955 | 6.09 | 114029.4 |
| IF2_BURL3_1 | Q39H30 | 5.94 | 104295.2 |
| IF2_CLOB8_1 | A6LSQ4 | 5 | 76128.05 |
| IF2_LACPL_1 | Q88VK7 | 8.92 | 93661.88 |
| IF2_PAEAT_1 | A1R516 | 6.7 | 98838.3 |
| IF2_BACTN_1 | Q8A2A1 | 6.47 | 114866.2 |
| IF2_BACVZ_1 | A7Z4T4 | 5.44 | 78595.65 |
| IF2_LAWIP_1 | Q1MQY8 | 6.37 | 106236 |
| IF2_BARHE_1 | Q6G4W7 | 7.09 | 93648.54 |
| IF2_RUTMC_1 | A1AV99 | 8.96 | 88783.09 |
| IF2_MYCGE_1 | P47388 | 9.02 | 67887.53 |
| IF2_MYCGI_1 | A4TCF8 | 6.29 | 95978.8 |
| IF2_BIFA0_1 | B8DW43 | 9.3 | 100016.5 |
| IF2_PELCD_1 | Q3A4A7 | 5.22 | 102913.3 |
| IF2_CAMLR_1 | B9KEV0 | 5.92 | 96556.42 |
| IF2_SALCH_1 | Q57JH9 | 5.91 | 97448.29 |
| IF2_COLP3_1 | Q482T9 | 5.54 | 95679.8 |
| IF2_EHRCR_1 | Q2GGQ8 | 6.01 | 93190.52 |
| IF2_MYCPN_1 | P75590 | 8 | 67897.24 |
| IF2_SALG2_1 | B5REN6 | 5.91 | 97414.26 |
| IF2_CORU7_1 | B1VGC2 | 8.89 | 97716.49 |
| IF2_SOLUE_1 | Q01W31 | 8.38 | 107507.1 |
| IF2_AGRFC_1 | Q8UJ51 | 7.31 | 98821.75 |
| IF2_ALCBS_1 | Q0VSS1 | 5.75 | 98532.51 |
| IF2_SULNB_1 | A6QBQ5 | 5.23 | 98543.4 |
| IF2_NITEC_1 | Q0AFJ3 | 6.71 | 96696.7 |
| IF2_GEOMG_1 | Q39VA6 | 6.93 | 96111.85 |
| IF2_RICM5_1 | A8F223 | 6.52 | 91239.75 |
| IF2_GEOSW_1 | C5D9C9 | 5.99 | 81945.4 |
| IF2_GLUDA_1 | A9HF18 | 7.24 | 97864.76 |
| IF2_GRABC_1 | Q0BPG2 | 8.89 | 98695.77 |
| IF2_STRGG_1 | B1VYN5 | 9.35 | 106008.1 |
| IF3 | Accession number | pI | MW |
| IF3_ECOLI_1 | P0A707 | 9.54 | 20563.92 |
| IF3_GEOSE_1 | P03000 | 9.89 | 19677.89 |
| IF3_MYXXA_1 | P48516 | 9.68 | 27013.85 |
| IF3_THET8_1 | Q5SKU2 | 9.57 | 19866.28 |
| IF3_SALTY_1 | P33321 | 9.54 | 20593.95 |
| IF3_HAEIN_1 | P43814 | 9.1 | 19630.76 |
| IF3_STRPN_1 | P65144 | 9.84 | 21164.79 |
| IF3_RHOS4_1 | O33567 | 9.27 | 20540.92 |
| IF3_BACSU_1 | P55872 | 9.85 | 19600.78 |
| IF3_MYCTU_1 | P9WKJ9 | 9.79 | 22333.48 |
| IF3_YERPE_1 | Q8ZDW6 | 9.49 | 20936.28 |
| IF3_THEMA_1 | Q9X1S6 | 9.78 | 20046.73 |
| IF3_COXBU_1 | Q83C11 | 10.17 | 21389.93 |
| IF3_XANCP_1 | Q8P7Z3 | 9.02 | 20992.31 |
| IF3_PSEAE_1 | Q9I0A0 | 9.6 | 20882.41 |
| IF3_BUCAP_1 | P46243 | 9.8 | 20659.27 |
| IF3_VIBCH_1 | O68844 | 9.59 | 20745.02 |
| IF3_LISMO_1 | P0A3L1 | 9.58 | 19647.02 |
| IF3_BACTN_1 | Q8AAP1 | 9.68 | 21840.5 |
| IF3_CLOAB_1 | Q97GK5 | 9.59 | 19857.27 |
| IF3_BACFR_1 | Q64VP1 | 9.3 | 23306.93 |
| IF3_ANASK_1 | B4UAN5 | 10.17 | 25359.55 |
| IF3_CLOPE_1 | Q8XJ67 | 9.39 | 19989.42 |
| IF3_LEGPA_1 | Q5X1H4 | 9.74 | 20374.62 |
| IF3_BLOFL_1 | Q7VR70 | 9.84 | 20391.99 |
| IF3_BORAP_1 | Q0SNX3 | 9.85 | 21594.17 |
| IF3_BORDL_1 | B5RL16 | 9.82 | 21640.19 |
| IF3_ANADE_1 | Q2IJB8 | 10.1 | 25472.66 |
| IF3_TROW8_1 | Q83HH0 | 9.92 | 20110.33 |
| IF3_AZOPC_1 | B6YQ94 | 9.5 | 21608.31 |
| IF3_CYAP8_1 | B7JYL4 | 9.71 | 20497.85 |
| IF3_DEIDV_1 | C1D0V9 | 9.18 | 23567.15 |
| IF3_DEIGD_1 | Q1IY80 | 9.44 | 23089.54 |
| IF3_LEPBJ_1 | Q04U56 | 9.54 | 20855.3 |
| IF3_LEPIC_1 | Q72PK8 | 9.55 | 20776.16 |
| IF3_BORBR_1 | Q7WKF6 | 9.35 | 18748.8 |
| IF3_METPB_1 | B1ZGB8 | 9.21 | 20010.11 |
| IF3_MYCPN_1 | P78024 | 9.91 | 23145.06 |
| IF3_HAMD5_1 | C4K777 | 9.77 | 20959.49 |
| IF3_CAMC5_1 | A7GVZ3 | 9.15 | 19685.02 |
| IF3_PROMT_1 | Q46IH3 | 9.73 | 23549.34 |
| IF3_CAMJ8_1 | A8FK08 | 8.9 | 19781.08 |
| IF3_NEIG1_1 | Q5F9U3 | 9.34 | 19778.97 |
| IF3_NEIMA_1 | P65137 | 9.34 | 19778.97 |
| IF3_CAUSK_1 | B0SY03 | 8.87 | 20011.42 |
| IF3_ACIBS_1 | B0VV99 | 9.5 | 20639.98 |
| IF3_PARL1_1 | A7HPJ7 | 8.54 | 19887.3 |
| IF3_RHIME_1 | Q92ST3 | 9.32 | 20218.78 |
| IF3_STRE4_1 | C0M8S9 | 9.83 | 20037.62 |
| IF3_STRMU_1 | Q8DV22 | 9.93 | 20102.65 |
| IF3_STRP6_1 | Q5XCU2 | 9.83 | 20053.62 |
| IF3_SHEB2_1 | B8EES7 | 9.34 | 20348.58 |
| IF3_YERP3_1 | A7FHG2 | 9.49 | 20936.28 |
| IF3_SHEWM_1 | B1KG60 | 9.47 | 20361.69 |
| IF3_SHIFL_1 | Q83L38 | 8.83 | 23374.05 |
| IF3_SOLUE_1 | Q02D92 | 9.93 | 24117.75 |
| IF3_CAUVN_1 | B8H312 | 8.87 | 19976.26 |
| IF3_CHLMU_1 | Q9PL86 | 9.5 | 21068.49 |
| IF3_CHLPM_1 | A4SCK3 | 9.46 | 24465.07 |
| IF3_CLOTE_1 | Q891T1 | 9.54 | 19763.34 |
| IF3_BARQU_1 | Q6G1I3 | 9.17 | 19467.83 |
| IF3_MESFL_1 | Q6F1S8 | 9.6 | 20846.23 |
| IF3_METRJ_1 | B1M6P9 | 9.21 | 19949.02 |
| IF3_METS4_1 | B0UP34 | 9.18 | 19912.95 |
| IF3_PROMH_1 | B4ETK7 | 9.44 | 20522.81 |
| IF3_PSEE4_1 | Q1IC14 | 9.4 | 20954.36 |
| IF3_CAMLR_1 | B9KEB2 | 8.86 | 19846.23 |
| IF3_STAAB_1 | Q2YTA9 | 9.72 | 20213.71 |
| IF3_STRTD_1 | Q03KI9 | 9.9 | 20161.76 |
| IF3_STRU0_1 | B9DU40 | 9.86 | 20019.64 |
| IF3_SYNPX_1 | Q7UA08 | 9.8 | 24906.97 |
| IF3_TREPA_1 | O83822 | 9.97 | 20358.8 |
| IF3_CHRVO_1 | Q7NYC5 | 9.25 | 19966.14 |
| IF3_CORGB_1 | A4QDY1 | 10.03 | 21690.13 |
| IF3_LEPBA_1 | B0SCH4 | 9.52 | 21516.94 |
| IF3_DESVH_1 | Q728R6 | 9.64 | 20496.71 |
| IF3_BORPE_1 | Q7VY63 | 9.35 | 18748.8 |
| IF3_METI4_1 | B3DYI1 | 9.8 | 24827.68 |
| IF3_BORT9_1 | A1QYY5 | 9.8 | 21573.09 |
| IF3_ELUMP_1 | B2KB83 | 9.54 | 20485.96 |
| IF3_ENTFA_1 | Q837C9 | 9.92 | 19517.76 |
| IF3_BRUME_1 | P0A3K8 | 9.4 | 20444.79 |
| IF3_MYCBO_1 | P65136 | 9.79 | 22333.48 |
| IF3_MYCGE_1 | P47438 | 9.94 | 21276.06 |
| IF3_GLOVI_1 | Q7NJS6 | 9.73 | 23130.9 |
| IF3_CAMC1_1 | A7ZAZ8 | 8.89 | 19723.94 |
| IF3_CAMHC_1 | A7I108 | 9.19 | 19855.28 |
| IF3_NAUPA_1 | B9L5U8 | 9.45 | 20605.19 |
| IF3_PSEFS_1 | C3JZN6 | 9.54 | 20850.36 |
| IF3_LACLA_1 | Q9CEJ7 | 10.13 | 20558.17 |
| IF3_PSEP1_1 | A5W5E0 | 9.4 | 20879.33 |
| IF3_RHILO_1 | Q98CP5 | 9.72 | 20224.66 |
| IF3_RHILW_1 | B5ZXR7 | 9.42 | 20185.71 |
| IF3_RHOBA_1 | Q7UJ17 | 9.69 | 20179.31 |
| IF3_PHEZH_1 | B4R9C1 | 8.87 | 20040.42 |
| IF3_PORGI_1 | Q7MVQ6 | 8.98 | 23148.5 |
| IF3_RICFE_1 | Q4ULG2 | 9.91 | 21540.61 |
| IF3_PROHU_1 | P33319 | 9.36 | 20534.76 |
| IF3_RUEPO_1 | Q5LQ58 | 9.47 | 20911.28 |
| IF3_SHEON_1 | Q8EER8 | 9.34 | 20394.65 |
| IF3_WIGBR_1 | Q8D3B9 | 10.17 | 22878.27 |
| IF3_WOLSU_1 | Q7M9L9 | 9.14 | 20372.76 |
| IF3_BORBU_1 | O51208 | 9.85 | 21534.12 |
| IF3_EHRCR_1 | Q2GI91 | 9.65 | 20017.63 |
| IF3_FUSNN_1 | Q8R5Y3 | 9.4 | 21838.44 |
| IF3_BRUSU_1 | P0A3K9 | 9.4 | 20444.79 |
| IF3_MYCFE_1 | Q05426 | 9.47 | 24486.22 |
| IF3_MYCLE_1 | Q9CC22 | 9.16 | 22517.49 |
| IF3_HAEDU_1 | Q7VKS0 | 9.41 | 21867.26 |
| IF3_KLEPN_1 | P33318 | 9.54 | 20504.9 |
| IF3_STAS1_1 | Q49YB2 | 9.74 | 20301.8 |
| IF3_STRA1_1 | Q3K0C8 | 9.77 | 20091.66 |
| IF3_UNCTG_1 | B1GZU2 | 9.81 | 21071.9 |
| IF3_XANAC_1 | Q8PJE2 | 9.02 | 20964.26 |
| IF3_AQUAE_1 | O67653 | 9.5 | 20819.51 |
| IF3_AROAE_1 | Q5P7X6 | 8.81 | 19926.11 |
| IF3_CHLTE_1 | Q8KAM9 | 9.42 | 24810.55 |
| IF3_BACCZ_1 | Q633M1 | 9.81 | 19080.22 |
| IF3_BACSK_1 | Q5WEI6 | 9.86 | 19093.28 |
| IF3_BACV8_1 | A6L7J6 | 9.26 | 22642.15 |
| IF3_BART1_1 | A9ILC8 | 9.25 | 19478.79 |
| IF3_GEOMG_1 | Q39VS8 | 9.76 | 19619.14 |
| IF3_MYCCT_1 | Q2SSS1 | 9.76 | 20997.26 |
| IF3_CHLCV_1 | Q822B2 | 9.53 | 21067.47 |
| IF3_CHLL2_1 | B3EEC0 | 9.66 | 24701.66 |
| IF3_AZOVI_1 | Q8RQ01 | 9.54 | 20781.22 |
| IF3_CHLPN_1 | Q9Z6R9 | 9.6 | 20867.28 |
| IF3_BORRA_1 | B5RR09 | 9.82 | 21640.19 |
| IF3_BRADU_1 | Q89WH7 | 9.42 | 20414.61 |
| IF3_MICAN_1 | B0JKQ1 | 9.44 | 20438.66 |
| IF3_GEOSL_1 | Q74D03 | 9.79 | 19592.01 |
| IF3_MYCPU_1 | Q98QV2 | 9.65 | 23283.02 |
| IF3_HYPNA_1 | Q0C535 | 9.39 | 20428.76 |
| IF3_IDILO_1 | Q5QYN5 | 9.25 | 20540.59 |
| IF3_LACJO_1 | Q74IC4 | 10.12 | 19526.97 |
| IF3_LACPL_1 | Q88WU8 | 9.96 | 19647.89 |
| IF3_NOSP7_1 | B2IY66 | 9.28 | 20762.2 |
| IF3_PSESM_1 | P0A132 | 9.54 | 20806.31 |
| IF3_ALISL_1 | B6EN33 | 9.65 | 20385.68 |
| IF3_SORC5_1 | A9F8E5 | 5.98 | 27939.8 |
| IF3_STACT_1 | B9DNC6 | 9.85 | 20317.88 |
| IF3_CHLL7_1 | Q3B6L9 | 9.54 | 23994.63 |
| IF3_CHLP8_1 | B3QRM3 | 9.63 | 24753.53 |
| IF3_CHLPD_1 | A1BJB3 | 9.52 | 24396.26 |
| IF3_BARBK_1 | A1UUD5 | 9.3 | 19667 |
| IF3_BARHE_1 | Q6G570 | 9.49 | 19431.79 |
| IF3_LISIN_1 | P0A3L2 | 9.58 | 19647.02 |
| IF3_BORHD_1 | B2RZQ1 | 9.82 | 21498.09 |
| IF3_BORPA_1 | Q7W911 | 9.35 | 18748.8 |
| IF3_BRUAB_1 | P0A3L0 | 9.4 | 20444.79 |
| IF3_MYCMS_1 | Q6MU22 | 9.78 | 21025.27 |
| IF3_CALS4_1 | Q8R9C2 | 9.69 | 21060.68 |
| IF3_HELPY_1 | P55973 | 9.57 | 23341.97 |
| IF3_NOSS1_1 | Q8YNE3 | 9.28 | 20750.13 |
| IF3_ACAM1_1 | B0CD77 | 9.77 | 20559.86 |
| IF3_OCHA4_1 | A6WX21 | 9.4 | 20411.79 |
| IF3_RICCN_1 | Q92HK6 | 9.81 | 21664.71 |
| IF3_PROA2_1 | B4S3D1 | 9.67 | 23264.93 |
| IF3_RICTY_1 | Q68WK5 | 9.68 | 21675.73 |
| IF3_SALPA_1 | Q5PH88 | 9.54 | 20623.98 |
| IF3_XYLFA_1 | Q9PFE1 | 9.46 | 20831.11 |
| IF3_YERE8_1 | A1JMK2 | 9.54 | 20906.24 |
| IF3_SULDN_1 | Q30UI3 | 8.78 | 19917.16 |
| IF3_ANAVT_1 | Q3MBH6 | 9.28 | 20750.13 |
| IF3_AZOC5_1 | A8HWL0 | 9.27 | 20007.33 |
| IF3_BACHD_1 | Q9K867 | 9.84 | 19291.49 |
| IF3_COREF_1 | Q8FTQ2 | 9.94 | 23193.78 |
| IF3_EHRRG_1 | Q5FGP0 | 9.47 | 20053.53 |
| IF3_NITSB_1 | A6Q167 | 9.35 | 20343.97 |
| IF3_OCEIH_1 | Q8EPF5 | 9.83 | 19857.07 |
| IF3_ACIAD_1 | Q6F861 | 9.48 | 20792.13 |
| IF3_PARD8_1 | A6L9S3 | 9.54 | 23603.45 |
| IF3_PASMU_1 | Q9CN42 | 9.34 | 20405.71 |
| IF3_PECAS_1 | Q6D4G9 | 9.56 | 20540.83 |
| IF3_PECCP_1 | C6DFY7 | 9.56 | 20577.9 |
| IF3_STRAW_1 | Q828D2 | 6.18 | 26779.48 |
| IF3_RICPR_1 | Q9ZD19 | 9.62 | 21604.69 |
| IF3_SALTI_1 | Q8Z6I3 | 9.63 | 20592.97 |
| IF3_XANP2_1 | A7IGN8 | 9.14 | 19968.2 |
| IF3_ZYMMO_1 | Q5NL80 | 9.49 | 20635.89 |
| IF3_STAEQ_1 | Q5HNM3 | 9.72 | 20268.79 |
| IF3_SYNY3_1 | P72874 | 9.36 | 20559.72 |
| IF3_THEEB_1 | Q8DIG8 | 9.32 | 20986.24 |
| IF3_CHLCH_1 | Q3APW0 | 8.91 | 26276.13 |
| IF3_BACAN_1 | Q81L15 | 9.81 | 19080.22 |
| IF3_CHLTR_1 | O84840 | 9.65 | 19770.15 |
| IF3_DEIRA_1 | Q9RSN7 | 9.18 | 23617.16 |
| IF3_BORBP_1 | Q662H4 | 9.79 | 21678.29 |
| IF3_MACCJ_1 | B9E784 | 9.73 | 20605.09 |
| IF3_METC4_1 | B7KV98 | 9.21 | 20038.16 |
| IF3_HELHP_1 | Q7VJ08 | 9.11 | 19899.3 |
| IF3_CAMFF_1 | A0RRI8 | 9.15 | 19758.05 |
| IF3_PSEHT_1 | Q3IL80 | 9.23 | 20598.87 |
| IF3_PSEPH_1 | Q9X6E7 | 9.54 | 20835.31 |
| IF3_RALSO_1 | Q8XZ28 | 9.47 | 20800.25 |
| IF3_STRCO_1 | O88060 | 8.82 | 24167.66 |
| IF3_SERMA_1 | P33320 | 8.45 | 20877.18 |
| IF3_UREPA_1 | Q9PQR4 | 9.67 | 20812.15 |
| EF-Tu | Accession number | pI | MW |
| EFTU1_THET8_1 | Q5SHN6 | 5.42 | 44650.98 |
| EFTU_LACJO_1 | Q74JU6 | 4.75 | 43664.22 |
| EFTU_MYCTU_1 | P9WNN1 | 5.28 | 43593.56 |
| EFTU_EHRRW_1 | Q5HAS0 | 5.23 | 43281.51 |
| EFTU_BACSU_1 | P33166 | 4.91 | 43593.27 |
| EFTU1_STRCU_1 | Q53871 | 5 | 43879.01 |
| EFTU_COXBU_1 | Q83ES6 | 5.31 | 43525.82 |
| EFTU_PSEAB_1 | Q02T82 | 5.22 | 43369.64 |
| EFTU_ANAPZ_1 | Q2GJ61 | 5.3 | 42858.09 |
| EFTU_AERHH_1 | A0KQ95 | 5.24 | 43381.69 |
| EFTU_ALCBS_1 | Q0VSL7 | 4.84 | 43222.01 |
| EFTU_RUEPO_1 | Q5LMR5 | 4.96 | 42723.48 |
| EFTU_MYCHP_1 | P22679 | 5.75 | 43595.89 |
| EFTU_MYCPN_1 | P23568 | 6.05 | 43149.3 |
| EFTU3_STRCO_1 | P40175 | 5.23 | 41676.24 |
| EFTU_RHOPA_1 | Q6N4Q4 | 5.52 | 43336.69 |
| EFTU_ACIAD_1 | Q6FF97 | 5.2 | 42954.91 |
| EFTU_ROSDO_1 | Q160Y4 | 4.89 | 42693.44 |
| EFTU_BUCAI_1 | O31297 | 5.57 | 43464.83 |
| EFTU_SHIDS_1 | Q32B27 | 5.3 | 43283.55 |
| EFTU_SALCH_1 | Q57H76 | 5.3 | 43283.55 |
| EFTU1_VIBVU_1 | Q8DD27 | 4.84 | 43162.15 |
| EFTU1_XANCB_1 | B0RU84 | 5.51 | 43208.49 |
| EFTU1_YERPS_1 | Q66FQ9 | 5.11 | 43170.26 |
| EFTU2_CAUSK_1 | B0T2B5 | 5.2 | 42960.23 |
| EFTU2_MYXXD_1 | Q1D776 | 5.68 | 43415.95 |
| EFTU_BACAN_1 | Q81VT2 | 4.91 | 42938.87 |
| EFTU_BACC1_1 | Q73F98 | 4.91 | 42924.84 |
| EFTU_BACFR_1 | P33165 | 5.22 | 43580.08 |
| EFTU_BACHD_1 | Q9Z9L6 | 4.8 | 43383.13 |
| EFTU_BACWK_1 | A9VP75 | 4.89 | 43154.12 |
| EFTU_BEUC1_1 | C5C0J3 | 5.02 | 43634.68 |
| EFTU_BORDL_1 | B5RM34 | 5.44 | 43461.94 |
| EFTU_BORPE_1 | Q7TT91 | 5.34 | 42916 |
| EFTU_BORT9_1 | A1QZR2 | 5.43 | 43291.75 |
| EFTU_BRAHW_1 | C0QVZ4 | 5.83 | 44461.84 |
| EFTU_BRASB_1 | A5ELM9 | 5.69 | 43482.94 |
| EFTU_BREBN_1 | C0ZIH6 | 4.97 | 43257.12 |
| EFTU_BRUC2_1 | A9M5Q2 | 5.27 | 42604.72 |
| EFTU_DESAH_1 | C0Q9Y7 | 5.1 | 43599.88 |
| EFTU_DESVV_1 | A1VAK4 | 5.18 | 43406.63 |
| EFTU2_YERPN_1 | Q1CCT9 | 5.17 | 43160.37 |
| EFTU_ACIAC_1 | A1TJ05 | 5.34 | 43128.26 |
| EFTU_ACIBT_1 | A3M1F6 | 5.21 | 42891.94 |
| EFTU_ACIC1_1 | A0LRL8 | 5.53 | 44053.49 |
| EFTU_ACICJ_1 | A5FZW7 | 5.33 | 42628.74 |
| EFTU_ALKEH_1 | Q0ABH7 | 4.88 | 43203.12 |
| EFTU_AMOA5_1 | B3ETZ7 | 5.41 | 43513.76 |
| EFTU_AROAE_1 | Q5P334 | 5.42 | 43204.44 |
| EFTU_AYWBP_1 | Q2NJ20 | 5.74 | 43407.52 |
| EFTU_AZOC5_1 | A8HTW6 | 5.5 | 43314.73 |
| EFTU_BAUCH_1 | Q1LSY4 | 5.64 | 43428.92 |
| EFTU_BIFAA_1 | A1A0T1 | 4.93 | 44105.69 |
| EFTU_BIFLS_1 | B7GU46 | 4.92 | 43879.46 |
| EFTU_BLOFL_1 | Q7VRP0 | 5.71 | 43474.82 |
| EFTU_BORBP_1 | Q661E5 | 5.6 | 43369.83 |
| EFTU_BORBR_1 | Q79G84 | 5.34 | 42916 |
| EFTU_BRELN_1 | P42471 | 5.02 | 43785.61 |
| EFTU_CYAP7_1 | B7K834 | 5.15 | 44856.29 |
| EFTU_DEHMB_1 | A5FQQ5 | 5.83 | 43971.7 |
| EFTU_DEIGD_1 | Q1IX70 | 5.27 | 44214.52 |
| EFTU_DEISP_1 | P33168 | 5.28 | 44340.64 |
| EFTU_EXIS2_1 | B1YGU8 | 4.91 | 43624.54 |
| EFTU_FRAAA_1 | Q0RRS3 | 5.41 | 43975.45 |
| EFTU_FRACC_1 | Q2JFH8 | 5.29 | 43946.36 |
| EFTU_FRAP2_1 | B0TX03 | 5.1 | 43322.52 |
| EFTU_RICPA_1 | Q8KTA6 | 5.5 | 42884.07 |
| EFTU1_HYPNA_1 | Q0C1F4 | 5.23 | 43164.46 |
| EFTU1_ORITB_1 | A5CCA0 | 5.56 | 42902.42 |
| EFTU1_PHOLL_1 | Q7N9B1 | 5.18 | 43174.29 |
| EFTU1_PHOPR_1 | Q6LVC0 | 4.79 | 42975.01 |
| EFTU1_PSYWF_1 | A5WGK9 | 5.01 | 43209.06 |
| EFTU1_RHOFT_1 | Q21SF0 | 5.67 | 42991.33 |
| EFTU1_RUTMC_1 | A1AVJ8 | 4.87 | 43452.35 |
| EFTU2_ECOLC_1 | B1IVA7 | 5.3 | 43313.58 |
| EFTU1_ROSCS_1 | A7NR65 | 5.53 | 43672.34 |
| EFTU1_SERP5_1 | A8G8E0 | 5.23 | 43188.38 |
| EFTU1_SHEB5_1 | A3DA74 | 5.12 | 43335.46 |
| EFTU1_SHEON_1 | Q8EK81 | 5.13 | 43347.45 |
| EFTU1_SYNWW_1 | Q0AUH8 | 5.18 | 44179.65 |
| EFTU1_WOLTR_1 | Q5GSU2 | 5.66 | 43712.28 |
| EFTU2_ACISJ_1 | A1WCN6 | 5.41 | 43243.34 |
| EFTU2_BARQU_1 | Q6FZL2 | 5.28 | 42874.06 |
| EFTU2_DESPS_1 | Q6AP73 | 4.83 | 43300.29 |
| EFTU2_RHIEC_1 | Q2K9L8 | 5.22 | 42780.88 |
| EFTU_AKKM8_1 | B2UQY9 | 5.04 | 43430.6 |
| EFTU_ANADE_1 | Q2II78 | 5.61 | 43330.77 |
| EFTU_ANAMM_1 | Q5PBH1 | 5.37 | 42914.04 |
| EFTU_ARTPT_1 | P13552 | 5.16 | 44757.94 |
| EFTU_BACCN_1 | A7GK18 | 4.81 | 42893.7 |
| EFTU_BACSK_1 | Q5WLR4 | 4.85 | 43433.02 |
| EFTU_BACV8_1 | A6KYK9 | 5.26 | 43563.01 |
| EFTU_BDEBA_1 | Q6MJ00 | 5.89 | 43265.81 |
| EFTU_BIFA0_1 | B8DTV7 | 4.87 | 44069.61 |
| EFTU_ENTFA_1 | Q839G8 | 4.73 | 43387.91 |
| EFTU_FLAPJ_1 | A6GYU7 | 5.13 | 43204.4 |
| EFTU_FRASN_1 | A8LC58 | 5.23 | 43958.42 |
| EFTU_FRAT1_1 | Q14JU2 | 5.11 | 43404.64 |
| EFTU_PSEFS_1 | C3K2X8 | 5.27 | 43563.96 |
| EFTU_PSEP1_1 | A5VXN3 | 5.21 | 43495.75 |
| EFTU_PSESM_1 | Q889X3 | 5.34 | 43364.58 |
| EFTU_PSYCK_1 | Q1Q8P2 | 5.04 | 43075.84 |
| EFTU_RICBR_1 | Q1RHL9 | 5.65 | 43161.5 |
| EFTU_RICCN_1 | Q92GW4 | 5.42 | 42868.02 |
| EFTU_RICMO_1 | Q8KTA1 | 5.5 | 42902.1 |
| EFTU_RICRI_1 | P0A3A9 | 5.5 | 42933.13 |
| EFTU1_METFK_1 | Q1H4Q1 | 5.34 | 42922.05 |
| EFTU1_PELCD_1 | Q3A6R2 | 4.9 | 43586.75 |
| EFTU1_ROSS1_1 | A5USJ1 | 5.59 | 43856.46 |
| EFTU1_SHELP_1 | A3Q968 | 4.9 | 43283.29 |
| EFTU1_SHESM_1 | Q0HNV1 | 5.09 | 43416.51 |
| EFTU1_SHISS_1 | Q3YWT3 | 5.3 | 43283.55 |
| EFTU1_STRAW_1 | Q82DQ0 | 5 | 43825.94 |
| EFTU2_ALKMQ_1 | A6TWJ8 | 5.02 | 43722.03 |
| EFTU2_HALHL_1 | A1WVD6 | 5.05 | 43125.02 |
| EFTU2_PELTS_1 | A5D5I8 | 5.6 | 44156.8 |
| EFTU2_SHEFN_1 | Q089Q6 | 5.07 | 43346.6 |
| EFTU2_STRRA_1 | P29543 | 5.53 | 44268.61 |
| EFTU2_YERE8_1 | A1JS52 | 5.23 | 43223.49 |
| EFTU_ACAM1_1 | B0CCD0 | 4.98 | 44773.95 |
| EFTU_ACHLI_1 | A9NEN4 | 5.2 | 42850.78 |
| EFTU_ACTP2_1 | A3N246 | 5.28 | 43466.53 |
| EFTU_ANADF_1 | A7HBL7 | 5.52 | 43397.76 |
| EFTU_ANAVT_1 | Q3MDM5 | 5.24 | 44797.27 |
| EFTU_ARCB4_1 | A8EW02 | 4.96 | 43951.04 |
| EFTU_AZOPC_1 | B6YQ04 | 5.47 | 43602.97 |
| EFTU_AZOSB_1 | A1KB29 | 5.4 | 43116.36 |
| EFTU_BACAH_1 | A0R8H8 | 4.91 | 42938.87 |
| EFTU_BACTN_1 | Q8A463 | 5.16 | 43634.99 |
| EFTU_BACVZ_1 | A7Z0N5 | 4.83 | 43413.06 |
| EFTU_BLOPB_1 | Q492B2 | 5.76 | 43841.25 |
| EFTU_BORAP_1 | Q0SN31 | 5.6 | 43353.83 |
| EFTU_BORBZ_1 | B7J241 | 5.52 | 43398.87 |
| EFTU_BORPA_1 | Q79GC6 | 5.34 | 42916 |
| EFTU_BORRA_1 | B5RPI0 | 5.44 | 43461.94 |
| EFTU_CYTH3_1 | Q11Q98 | 5.49 | 43021.38 |
| EFTU_DESAD_1 | C6C171 | 4.74 | 43114.96 |
| EFTU_DESAG_1 | Q30X13 | 4.99 | 43434.67 |
| EFTU_DESDA_1 | B8J1A0 | 5.14 | 43441.66 |
| EFTU_DESHD_1 | B8G1W4 | 5.27 | 43694.99 |
| EFTU_DESRM_1 | A4J0Z5 | 5.18 | 43951.01 |
| EFTU_DINSH_1 | A8LLG2 | 4.91 | 42776.63 |
| EFTU_EHRCJ_1 | Q3YRK7 | 5.31 | 43480.73 |
| EFTU_ENT38_1 | A4W5A0 | 5.24 | 43244.43 |
| EFTU_EXISA_1 | C4KZP9 | 4.86 | 43482.31 |
| EFTU_FERNB_1 | A7HM54 | 5.36 | 44338.14 |
| EFTU_FLAJ1_1 | A5FIJ9 | 5.24 | 43051.24 |
| EFTU_FLESI_1 | P26184 | 5.01 | 43900.11 |
| EFTU_PSYA2_1 | Q4FQG6 | 5.09 | 43098.87 |
| EFTU_PSYIN_1 | A1T056 | 5.17 | 43262.55 |
| EFTU_RENSM_1 | A9WSW5 | 5.11 | 43707.66 |
| EFTU_RICPR_1 | P48865 | 5.77 | 42921.18 |
| EFTU_RICSI_1 | P0A3B0 | 5.5 | 42933.13 |
| EFTU_BRUA2_1 | Q2YM08 | 5.27 | 42604.72 |
| EFTU_ERYLH_1 | Q2N9A8 | 4.8 | 43063.68 |
| EFTU_FERIS_1 | O50340 | 5.31 | 44145 |
| EFTU_FIBSS_1 | P42475 | 5.43 | 43286.53 |
| EFTU_PSEE4_1 | Q1IFW8 | 5.21 | 43478.73 |
| EFTU_PSELT_1 | A8F4Q9 | 5.3 | 43916.35 |
| EFTU_RALSO_1 | Q8XGZ0 | 5.41 | 43167.44 |
| EFTU_RICAH_1 | A8GPF2 | 5.83 | 42890.15 |
| EFTU_RICCK_1 | A8EZL8 | 5.5 | 42864.08 |
| EFTU_SYNPX_1 | Q7U4D1 | 4.9 | 43638.83 |
| EFTU_CHLPN_1 | Q9Z9A7 | 5.43 | 43005.09 |
| EFTU_MICLU_1 | P09953 | 5.12 | 43820.69 |
| EFTU_GEOSL_1 | Q748X8 | 5.14 | 43218.32 |
| EFTU_MYCA9_1 | B1MGH7 | 5.06 | 43527.33 |
| EFTU_MYCAP_1 | A5IYA9 | 5.89 | 43550.86 |
| EFTU_MYCBO_1 | P0A559 | 5.28 | 43593.56 |
| EFTU_CORGL_1 | P42439 | 4.92 | 43851.86 |
| EFTU_CHLAA_1 | A9WFP3 | 5.87 | 44018.85 |
| EFTU_MYCGE_1 | P13927 | 6.17 | 42990.22 |
| EFTU_LACSS_1 | Q38WR7 | 4.71 | 43277.73 |
| EFTU_CHLCV_1 | Q822I4 | 5.35 | 43095.23 |
| EFTU_PAEAT_1 | A1R8U9 | 5.04 | 43563.41 |
| EFTU_PARD8_1 | A6LE88 | 5.34 | 43609.99 |
| EFTU_LEGPL_1 | Q5WZL4 | 5.22 | 43193.33 |
| EFTU_MYCMM_1 | B2HSL3 | 5.22 | 43805.77 |
| EFTU_MYCMO_1 | Q6KI66 | 5.5 | 43968.05 |
| EFTU_MYCMS_1 | Q6MU81 | 5.11 | 43304.2 |
| EFTU_HAEIE_1 | A5U9R1 | 5.26 | 43354.36 |
| EFTU_PEDPA_1 | Q03F25 | 4.73 | 43311.83 |
| EFTU_MYCS2_1 | A0QS98 | 5.18 | 43735.72 |
| EFTU_HAES1_1 | Q0I1U9 | 5.28 | 43386.41 |
| EFTU_PELUB_1 | Q4FLK5 | 5.51 | 43195.45 |
| EFTU_LISMC_1 | C1KZK6 | 4.81 | 43342.06 |
| EFTU_PHYMT_1 | B3QZH5 | 6.42 | 43729.27 |
| EFTU_LISW6_1 | A0ALY8 | 4.81 | 43342.06 |
| EFTU_LYSSC_1 | B1HMZ0 | 4.89 | 43263.14 |
| EFTU_MACCJ_1 | B9E8Q0 | 4.78 | 43386.02 |
| EFTU_NEIG1_1 | Q5F5Q8 | 5.17 | 42938.01 |
| EFTU_POLAQ_1 | A4SUU7 | 5.41 | 43013.36 |
| EFTU_MARHV_1 | A1TYJ5 | 5.03 | 43662.74 |
| EFTU_NEIMF_1 | A1KRF9 | 5.07 | 42908.92 |
| EFTU_HISS2_1 | B0UV21 | 5.28 | 43386.41 |
| EFTU_MARMS_1 | A6W394 | 4.9 | 44330.37 |
| EFTU_PROM1_1 | A2C4U5 | 4.91 | 43581.69 |
| EFTU_METI4_1 | B3E156 | 5.67 | 43854.58 |
| EFTU_NITOC_1 | Q3J8Q0 | 5.03 | 43673.81 |
| EFTU_MICAN_1 | B0JSE0 | 5.29 | 44918.28 |
| EFTU_KITAU_1 | O33594 | 5.06 | 43509.67 |
| EFTU_SACD2_1 | Q21M86 | 4.82 | 44287.28 |
| EFTU_STREM_1 | B4U3U1 | 4.88 | 43868.59 |
| EFTU_PSECP_1 | B8HD11 | 5.15 | 43591.55 |
| EFTU_SALAR_1 | A9MHG0 | 5.3 | 43283.55 |
| EFTU_THEMA_1 | P13537 | 5.3 | 44466.98 |
| EFTU_THENN_1 | B9K884 | 5.16 | 44555.04 |
| EFTU_STRP3_1 | P0DA82 | 4.91 | 43825.56 |
| EFTU_VIBCB_1 | A7MXE4 | 4.8 | 43137.03 |
| EFTU_STRU0_1 | B9DRL9 | 4.93 | 43913.67 |
| EFTU_SYNFM_1 | A0LIH6 | 5.49 | 44025.49 |
| EFTU_STAAS_1 | Q6GBT9 | 4.74 | 43103.64 |
| EFTU_STRA5_1 | Q8E0H1 | 4.83 | 44009.72 |
| EFTU_TRIEI_1 | Q118Z2 | 5.11 | 44586.82 |
| EFTU_TROWT_1 | Q83GW1 | 5.35 | 43520.58 |
| EFTU_SHIBS_1 | Q31VV0 | 5.3 | 43283.55 |
| EFTU_STRR6_1 | P64031 | 4.86 | 43970.74 |
| EFTU_SOLUE_1 | Q01SX2 | 5.84 | 43550.13 |
| EFTU_SYNE7_1 | P33171 | 5.21 | 44190.42 |
| EFTU_STAEQ_1 | Q5HRK4 | 4.7 | 43157.71 |
| EFTU_STAS1_1 | Q49V58 | 4.63 | 43221.66 |
| EFTU_STIAU_1 | P42479 | 5.77 | 43366.91 |
| EFTU_BRUO2_1 | A5VR08 | 5.27 | 42604.72 |
| EFTU_BRUSI_1 | B0CH34 | 5.27 | 42604.72 |
| EFTU_UREP2_1 | B1AJG3 | 5.83 | 42902.1 |
| EFTU_VEREI_1 | A1WHC3 | 5.53 | 43183.41 |
| EFTU_STRT1_1 | Q5M101 | 4.89 | 43866.66 |
| EFTU_VIBC3_1 | A5F3K0 | 5.04 | 43126.32 |
| EFTU_ZYMMO_1 | Q5NQ65 | 5.11 | 43095.19 |
| EFTU_CHLPD_1 | A1BJ36 | 5.37 | 43288.68 |
| EFTU_CHLT2_1 | B0B7N8 | 5.44 | 43177.47 |
| EFTU_CHLTE_1 | Q8KAH0 | 5.29 | 42899.21 |
| EFTU_CHRSD_1 | Q1R0H7 | 4.89 | 43345.26 |
| EFTU_BURM9_1 | A2S7F9 | 5.34 | 42991.2 |
| EFTU1_BARBK_1 | A1USC1 | 5.21 | 42802.87 |
| EFTU1_CARHZ_1 | Q3A9R3 | 5.36 | 44193.8 |
| EFTU_CALS8_1 | A4XI37 | 5.43 | 44017.55 |
| EFTU_CAMC5_1 | A7GZK6 | 5.15 | 43745.92 |
| EFTU_CAMJE_1 | O69303 | 5.11 | 43593.62 |
| EFTU_BURCE_1 | P33167 | 5.4 | 42876.01 |
| EFTU_BURCM_1 | Q0BJ48 | 5.4 | 42934.15 |
| EFTU_BURL3_1 | Q39KI2 | 5.4 | 42934.15 |
| EFTU_CHRVO_1 | Q7M7F1 | 5.11 | 43072.05 |
| EFTU_CLAMS_1 | B0RB36 | 5.22 | 43428.43 |
| EFTU_CLOB1_1 | A7FZ71 | 5.1 | 43483.41 |
| EFTU_CLONN_1 | A0PXT1 | 4.94 | 43095.92 |
| EFTU_LACF3_1 | B2GBC2 | 4.84 | 43474.15 |
| EFTU_GEOKA_1 | Q5L3Z9 | 4.86 | 43306.24 |
| EFTU_MYCA5_1 | B3PMU1 | 5.78 | 43699.97 |
| EFTU_OCHA4_1 | A6X0A2 | 5.21 | 42573.66 |
| EFTU_COREF_1 | Q8FS84 | 4.88 | 43743.76 |
| EFTU_GEOUR_1 | A5GAW4 | 5.24 | 42998.25 |
| EFTU_LACP7_1 | A9KRZ4 | 5.27 | 43856.11 |
| EFTU_LACRD_1 | A5VJ92 | 4.92 | 43432.18 |
| EFTU_GLUOX_1 | Q5FTY1 | 5.15 | 42980.96 |
| EFTU_LACS1_1 | Q1WU83 | 4.78 | 43273.93 |
| EFTU_ONYPE_1 | Q6YQV8 | 5.56 | 43406.45 |
| EFTU_CHLCH_1 | Q3APH1 | 5.24 | 43102.39 |
| EFTU_CROS8_1 | A7MKI5 | 5.35 | 43204.41 |
| EFTU_MYCHJ_1 | Q4A9G1 | 5.61 | 44122.49 |
| EFTU_STRMU_1 | P72483 | 4.84 | 43918.61 |
| EFTU_STROR_1 | P33170 | 4.87 | 44012.78 |
| EFTU_THEM4_1 | A6LLL1 | 4.98 | 44405.79 |
| EFTU_THESQ_1 | B1LBP2 | 5.36 | 44466 |
| EFTU_SORC5_1 | A9ETD1 | 6.44 | 43373.96 |
| EFTU_SPHWW_1 | A5V604 | 5.17 | 42943.94 |
| EFTU_CHLT3_1 | B3QY22 | 5.08 | 42990.15 |
| EFTU_CLOCE_1 | B8I5N8 | 5.21 | 43815.06 |
| EFTU_CLOK5_1 | A5N4N1 | 5.2 | 43674.97 |
| EFTU_LACCB_1 | B3WE38 | 4.85 | 43573.5 |
| EFTU_CLOPS_1 | Q0SQC8 | 4.92 | 43557.49 |
| EFTU_MOOTA_1 | Q2RFP5 | 5.21 | 44124.62 |
| EFTU_CLOTH_1 | A3DJ00 | 5.35 | 44188.47 |
| EFTU_CAMLR_1 | B9KFF9 | 5.02 | 43672.58 |
| EFTU_CARRP_1 | Q05FI3 | 5.79 | 44125.04 |
| EFTU_OCEIH_1 | Q8ETY4 | 4.81 | 43227.77 |
| EFTU_CORDI_1 | Q6NJD5 | 4.89 | 43943.95 |
| EFTU_LACPL_1 | Q88VE0 | 4.94 | 43377.13 |
| EFTU_GLUDA_1 | A9H3R7 | 5.33 | 43009.03 |
| EFTU_CHLAB_1 | Q5L5H6 | 5.35 | 43213.42 |
| EFTU_GRABC_1 | Q0BUQ2 | 5.34 | 43008.16 |
| EFTU_GRAFK_1 | A0M3Z6 | 5 | 43048.92 |
| EFTU_CHLFF_1 | Q255F3 | 5.35 | 43191.38 |
| EFTU_CHLL7_1 | Q3B6G3 | 5.25 | 43072.31 |
| EFTU_MYCLB_1 | B8ZSC1 | 5.28 | 43667.65 |
| EFTU_CUTAK_1 | Q6A6L7 | 5.32 | 44140.3 |
| EFTU_PARL1_1 | A7HWP7 | 5.28 | 43194.46 |
| EFTU_LEPBA_1 | B0SAF6 | 5.73 | 43925.38 |
| EFTU_LEPBL_1 | Q055E6 | 5.66 | 43595.99 |
| EFTU_THEAB_1 | B7IHU4 | 5 | 44393.82 |
| EFTU_THEAQ_1 | Q01698 | 5.43 | 44683.1 |
| EFTU_THEEB_1 | Q8DI42 | 5.28 | 44945.41 |
| EFTU_THEP1_1 | A5IM81 | 5.36 | 44466 |
| EFTU_THIDA_1 | Q3SLQ1 | 5.35 | 43079.23 |
| EFTU_THIDL_1 | P42481 | 5.57 | 43026.36 |
| EFTU_WIGBR_1 | Q8D240 | 6.05 | 43710.46 |
| EFTU_SULDN_1 | Q30TQ5 | 4.97 | 43606.69 |
| EFTU_CHLMU_1 | Q9PK73 | 5.36 | 43215.7 |
| EFTU_CHLPM_1 | A4SCQ7 | 5.18 | 43015.21 |
| EFTU_BURCA_1 | Q1BRT3 | 5.4 | 42934.15 |
| EFTU_CAMC1_1 | A7ZCN0 | 5.13 | 43613.71 |
| EFTU_CAMFF_1 | A0RQJ3 | 4.98 | 43680.63 |
| EFTU_CAMHC_1 | A7I3U7 | 5.24 | 43652.76 |
| EFTU_LACH4_1 | A8YUS2 | 4.94 | 43566.4 |
| EFTU_COLP3_1 | Q47UU9 | 4.86 | 43217.24 |
| EFTU_CORA7_1 | C3PKP2 | 4.87 | 44100.28 |
| EFTU_SHEAM_1 | A1S204 | 5.14 | 43367.56 |
| EFTU_VESOH_1 | A5CW32 | 4.91 | 43408.37 |
| EFTU_SPHAL_1 | Q1GP97 | 5.11 | 42953.06 |
| EFTU_STACT_1 | B9DKV8 | 4.67 | 43244.78 |
| EFTU_LACGA_1 | Q042T5 | 4.84 | 43677.35 |
| EFTU_LACLA_1 | Q9CEI0 | 4.89 | 43211.91 |
| EFTU_GEOSW_1 | C5D3R5 | 4.89 | 43391.31 |
| EFTU_OENOB_1 | Q04FQ4 | 5.01 | 43624.56 |
| EFTU_CHLL2_1 | B3EH93 | 5.37 | 43140.56 |
| EFTU_CUPNH_1 | Q0K5Z9 | 5.48 | 43046.39 |
| EFTU_MYCPA_1 | Q73SD1 | 5.17 | 43770.74 |
| EFTU_LEPIC_1 | Q72NF9 | 5.73 | 43574.05 |
| EFTU_LEUCK_1 | B1MY04 | 4.82 | 43457.22 |
| EFTU_MYCSK_1 | A1UBL1 | 5.17 | 43763.6 |
| EFTU_HELAH_1 | Q17VM8 | 5.2 | 43539.73 |
| EFTU_MYCUA_1 | A0PM42 | 5.22 | 43805.77 |
| EFTU_HELPJ_1 | Q9ZK19 | 5.16 | 43729.89 |
| EFTU_MAGMM_1 | A0L5V8 | 5.02 | 43194.32 |
| EFTU_MANSM_1 | Q65QG6 | 5.23 | 43390.43 |
| EFTU_PORG3_1 | B2RL52 | 5.31 | 43668.96 |
| EFTU_NITMU_1 | Q2YAZ9 | 5.49 | 42925.17 |
| EFTU_METSB_1 | B8ELG5 | 5.48 | 43134.54 |
| EFTU_SALPA_1 | Q5PIW4 | 5.3 | 43283.55 |
| EFTU_SALTO_1 | A4XBP8 | 5.31 | 43999.49 |
| EFTU_SHEDO_1 | Q12SW1 | 5.08 | 43367.53 |
| EFTU_TREDE_1 | Q73PN3 | 5.42 | 43788.02 |
| EFTU_SHEPA_1 | A8GYW2 | 4.89 | 43159.23 |
| EFTU_SHEPC_1 | A4YBY5 | 5.08 | 43289.44 |
| EFTU_STRSV_1 | A3CP09 | 4.85 | 43937.79 |
| EFTU_SULMW_1 | A8Z5T8 | 6.44 | 43862.64 |
| EFTU_SYNY3_1 | P74227 | 5.16 | 43733.03 |
| EFTU_TERFE_1 | P42476 | 5.37 | 43166.38 |
| EFTU_GEOMG_1 | Q39Y08 | 5.18 | 43207.29 |
| EFTU_MYCA1_1 | A0QL35 | 5.17 | 43770.74 |
| EFTU_CORJK_1 | Q4JT41 | 4.91 | 43914.86 |
| EFTU_MYCCT_1 | Q2SSW8 | 5.11 | 43304.2 |
| EFTU_CORU7_1 | B1VET1 | 4.89 | 43819.71 |
| EFTU_OLICO_1 | B6JET1 | 5.72 | 43443.9 |
| EFTU_MYCGI_1 | A4T1R2 | 5.17 | 43697.73 |
| EFTU_OPITP_1 | B1ZPC5 | 6.1 | 43021.31 |
| EFTU_LAWIP_1 | Q1MPT8 | 5.29 | 43589.98 |
| EFTU_PARDP_1 | A1B002 | 5.14 | 42971.92 |
| EFTU_LEIXX_1 | Q6ACZ0 | 5.14 | 43394.24 |
| EFTU_PELPD_1 | A1ALS6 | 5.18 | 43079.2 |
| EFTU_LISIN_1 | Q927I6 | 4.81 | 43353.08 |
| EFTU_HAMD5_1 | C4K4F8 | 5.49 | 43486.85 |
| EFTU_PETMO_1 | A9BHA7 | 5.12 | 44023.38 |
| EFTU_HELHP_1 | Q7VJ74 | 5.1 | 43699.76 |
| EFTU_MYCVP_1 | A1T4L6 | 5.08 | 43802.77 |
| EFTU_PLARO_1 | P72231 | 5.35 | 43911.28 |
| EFTU_POLNA_1 | A1VIP8 | 5.38 | 42946.23 |
| EFTU_POLSJ_1 | Q123F6 | 5.61 | 42940.24 |
| EFTU_HERAU_1 | P42477 | 5.35 | 43738.22 |
| EFTU_NEOSM_1 | Q2GD83 | 5.32 | 46705.26 |
| EFTU_PROA2_1 | B4S5M9 | 4.96 | 43099.34 |
| EFTU_NITEU_1 | Q81ZS3 | 5.21 | 42928.19 |
| EFTU_HYDCU_1 | Q31IY4 | 4.87 | 43247.92 |
| EFTU_METPP_1 | A2SLF9 | 5.54 | 43088.3 |
| EFTU_JANMA_1 | A6T3K6 | 5.48 | 43109.27 |
| EFTU_JANSC_1 | Q28UW7 | 4.85 | 42738.41 |
| EFTU_KINRD_1 | A6W5T5 | 5.11 | 43714.94 |
| EFTU_KOCRD_1 | B2GIL2 | 4.97 | 43549.43 |
| EFTU_KORVE_1 | Q1IHG6 | 5.66 | 43030.35 |
| EFTU_NOCSJ_1 | A1SNN5 | 5.05 | 43852.99 |
| EFTU_LACAC_1 | Q5FKR8 | 4.96 | 43579.5 |
| EFTU_LACBA_1 | Q03QN5 | 4.72 | 43639.11 |
| EFTU_STRCJ_1 | P95724 | 5.09 | 43891.11 |
| EFTU_STRGC_1 | A8AWA0 | 4.86 | 44010.85 |
| EFTU_SALRD_1 | Q2S1P8 | 4.47 | 43729.17 |
| EFTU_SHEHH_1 | B0TM14 | 4.89 | 43111.13 |
| EFTU_TREPA_1 | O83217 | 5.56 | 43260.49 |
| EFTU_SHESH_1 | A8G1F0 | 4.83 | 43163.13 |
| EFTU_XANOM_1 | Q2NZX1 | 5.45 | 43080.29 |
| EFTU_SYMTH_1 | Q67JU1 | 5.44 | 43578.86 |
| EFTU_TERTT_1 | C5BQ44 | 4.79 | 44279.28 |
| EFTU_BURP0_1 | A3P0B5 | 5.34 | 42991.2 |
| EFTU_CLOAB_1 | Q97EH5 | 5.02 | 43452.55 |
| EFTU_CLOB8_1 | A6LPP6 | 5.01 | 43623.74 |
| EFTU_BURTA_1 | Q2SU25 | 5.34 | 42991.2 |
| EFTU_BURVG_1 | A4JAM5 | 5.33 | 43033.24 |
| EFTU_CALBD_1 | B9MQH1 | 5.43 | 44007.51 |
| EFTU_LACDA_1 | Q1GAQ0 | 4.84 | 43280.96 |
| EFTU_FUSNN_1 | Q8R603 | 5.18 | 43457.55 |
| EFTU_GEMAT_1 | C1A6Q3 | 5.66 | 44213.63 |
| EFTU_NOVAD_1 | Q2G8Y2 | 5.06 | 42976.87 |
| EFTU_GEOSE_1 | O50306 | 4.9 | 43290.28 |
| EFTU_CELLY_1 | P42474 | 5.03 | 42972.88 |
| EFTU_GEOTN_1 | A4IJI7 | 4.86 | 43323.19 |
| EFTU_LACP3_1 | Q039K9 | 4.85 | 43573.5 |
| EFTU_CHESB_1 | Q11HA6 | 5.17 | 42793.82 |
| EFTU_CORK4_1 | C4LL63 | 4.92 | 43854.69 |
| EFTU_MYCGA_1 | P18906 | 5.57 | 43099.15 |
| EFTU_CUPMC_1 | Q1LI13 | 5.41 | 43088.38 |
| EFTU_PAROE_1 | P42480 | 5.25 | 43038.2 |
| EFTU_PARUW_1 | Q6MDN0 | 5.89 | 42988.42 |
| EFTU_PARXL_1 | Q13TF5 | 5.4 | 43058.25 |
| EFTU_MYCPE_1 | Q8EX18 | 6.05 | 43074.27 |
| EFTU_PECAS_1 | Q6CZW6 | 5.24 | 43274.58 |
| EFTU_MYCPU_1 | Q98QG1 | 5.45 | 43227.35 |
| EFTU_PELPB_1 | B4SBU5 | 5.43 | 42943.29 |
| EFTU_MYCS5_1 | Q4A597 | 5.61 | 43230.46 |
| EFTU_LEUMM_1 | Q03YI2 | 4.77 | 43371.05 |
| EFTU_HAHCH_1 | Q2S8Z8 | 5.08 | 43243.42 |
| EFTU_PEPD6_1 | Q18CE4 | 4.93 | 44026.06 |
| EFTU_HELMI_1 | B0TC54 | 5.38 | 44013.47 |
| EFTU_NAUPA_1 | B9L7I8 | 4.88 | 43883.92 |
| EFTU_MAGSA_1 | Q2W2H3 | 5.34 | 43053.36 |
| EFTU_HERAR_1 | A4G9U0 | 5.47 | 42997.2 |
| EFTU_MARMM_1 | Q0ANN1 | 4.95 | 43126.09 |
| EFTU_MESFL_1 | Q6F0J5 | 4.98 | 42904.79 |
| EFTU_NITHX_1 | Q1QN32 | 5.67 | 43181.61 |
| EFTU_METCA_1 | Q605B0 | 5.28 | 43046.32 |
| EFTU_IDILO_1 | Q5QWA3 | 4.92 | 43254.25 |
| EFTU_NITSB_1 | A6Q1L5 | 4.94 | 43933.03 |
| EFTU_NITWN_1 | Q3SSW8 | 5.55 | 43179.59 |
| EFTU_NOCFA_1 | Q5YPG4 | 5.14 | 43768.9 |
| EFTU_KOSOT_1 | C5CGR6 | 5.14 | 44098.65 |
| EFTU_NOSP7_1 | B2J5B1 | 5.46 | 44711.28 |
| EFTU_NOSS1_1 | Q8YP63 | 5.25 | 44811.3 |
| EFTU_RUEST_1 | Q1GDV0 | 4.87 | 42704.52 |
| EFTU_PSE14_1 | Q48D34 | 5.34 | 43445.73 |
| EFTU_PSEA6_1 | Q15NP2 | 4.85 | 43026.91 |
| EFTU_SACEN_1 | A4FPM7 | 5.09 | 44041.24 |
| EFTU_SALAI_1 | A8M531 | 5.36 | 43989.46 |
| EFTU_UREU1_1 | B5ZC31 | 5.83 | 42918.1 |
| EFTU_SINMW_1 | A6U842 | 5.28 | 42748.88 |
| EFTU_STRS2_1 | A4VZZ3 | 4.86 | 44046.75 |
| EFTU_WOLSU_1 | P42482 | 5.11 | 43602.71 |
| EFTU_STAHJ_1 | Q4L3K9 | 4.7 | 43091.63 |
| EFTU_CHLP8_1 | P42473 | 5.12 | 42698.83 |
| EFTU_SODGM_1 | Q2NQL7 | 5.22 | 43106.24 |
| EFTU_SULNB_1 | A6Q6H4 | 4.66 | 44070.98 |
| EFTU_SYNAS_1 | Q2LQA3 | 5.29 | 43912.28 |
| EFTU1_NITEC_1 | Q0AIJ7 | 5.49 | 42938.29 |
| EFTU1_PSEHT_1 | Q3ILP4 | 4.98 | 43306.4 |
| EFTU1_PSEU5_1 | A4VHL6 | 5.36 | 43445.78 |
| EFTU1_RHORT_1 | Q2RQV8 | 5.1 | 43142.21 |
| EFTU1_SHIF8_1 | Q0SZX8 | 5.36 | 43282.57 |
| EFTU2_WOLPM_1 | Q73H85 | 5.84 | 42654.16 |
| EFTU_ACTSZ_1 | A6VKH7 | 5.17 | 43308.29 |
| EFTU_AERS4_1 | A4SHU2 | 5.17 | 43299.61 |
| EFTU_AGARV_1 | C4ZB99 | 5.03 | 43410.38 |
| EFTU_ALKOO_1 | A8MLC4 | 5.21 | 43898.03 |
| EFTU_ALTMD_1 | B4RYQ8 | 4.94 | 43224.25 |
| EFTU_ANOFW_1 | B7GJ65 | 4.92 | 43329.33 |
| EFTU_APPPP_1 | Q9ZEU3 | 6.55 | 43699.28 |
| EFTU_BARHE_1 | Q8KHX9 | 5.28 | 42866.01 |
| EFTU_BART1_1 | A9ISD9 | 5.28 | 42823.93 |
| EFTU_BRADU_1 | Q89J82 | 5.77 | 43424.88 |
| EFTU_DECAR_1 | Q47JA5 | 5.32 | 43161.37 |
| EFTU_EUBE2_1 | C4Z2R9 | 5.06 | 43997.02 |
| EFTU_PSEMY_1 | A4XZ92 | 5.36 | 43587.98 |
| EFTU_RHIL3_1 | Q1MIE3 | 5.34 | 42593.81 |
| EFTU_RHOE4_1 | C0ZVT7 | 5.02 | 43312.31 |
| EFTU_RHOOB_1 | C1AYS3 | 5.09 | 43536.57 |
| EFTU_RHOS1_1 | A3PGI1 | 5.28 | 42873.94 |
| EFTU_RICAE_1 | C3PPA9 | 5.5 | 42878.11 |
| EFTU_RICFE_1 | Q8KT97 | 5.59 | 42894.19 |
| EFTU_RICTY_1 | Q8KT95 | 5.77 | 42981.24 |
| EFTU_RUBXD_1 | Q1AU14 | 4.97 | 44073.16 |
| EFTU_AQUPY_1 | O50293 | 5.14 | 44672.2 |
| EFTU_ARTS2_1 | A0JZ88 | 5.16 | 43591.55 |
| EFTU_BACLD_1 | Q65PA9 | 4.9 | 43653.41 |
| EFTU_BACP2_1 | A8F982 | 4.89 | 43497.1 |
| EFTU_DICNV_1 | A5EX84 | 5.12 | 43165.28 |
| EFTU_EHRCR_1 | Q2GFN6 | 5.37 | 43386.65 |
| EFTU_RHOBA_1 | Q7UMZ0 | 5.4 | 43185.04 |
| EFTU_RHOJR_1 | Q0SFF4 | 5.09 | 43536.57 |
| EFTU_RICHE_1 | Q8KT99 | 5.59 | 42942.19 |
| EFTU_RICM5_1 | A8F2E9 | 5.35 | 42850.99 |
| EFTU_RICPU_1 | C4K2I2 | 5.5 | 42878.11 |
| EFTU_RICRH_1 | Q8KTA3 | 5.28 | 42905.09 |
| EF-G | Accession number | pI | MW |
| EFG_THETH_1 | P13551 | 5.3 | 76879.22 |
| EFG_STAAU_1 | P68790 | 4.79 | 76481.21 |
| EFG_BACSU_1 | P80868 | 4.79 | 76485.41 |
| EFG_ACHLI_1 | A9NEN3 | 5.29 | 76334.8 |
| EFG_AERS4_1 | A4SHV8 | 5.3 | 77497.38 |
| EFG_ALCBS_1 | Q0VSL8 | 4.86 | 77313.5 |
| EFG_ACIB3_1 | B7GYM8 | 5.04 | 78805.89 |
| EFG_ACTSZ_1 | A6VL11 | 5.04 | 77410.73 |
| EFG_ACIAC_1 | A1TJ04 | 5.26 | 77381.56 |
| EFG_ACIAD_1 | Q6FDS6 | 5.04 | 78796.75 |
| EFG_ACIC5_1 | C1F645 | 5.49 | 76546.58 |
| EFG_ACIF2_1 | B7J464 | 5.13 | 76555.26 |
| EFG_AGRRK_1 | B9JDS6 | 5.13 | 77747.58 |
| EFG_CLOK5_1 | A5N4P4 | 5.03 | 76490.96 |
| EFG_MANSM_1 | Q65W89 | 5.01 | 77226.54 |
| EFG_MARMM_1 | Q0ANP7 | 4.99 | 76150.08 |
| EFG_STAIN_1 | Q5U8S9 | 4.79 | 76599.31 |
| EFG_PSECP_1 | B8HD12 | 4.98 | 77661.14 |
| EFG_NEIM0_1 | A9M3X0 | 5.08 | 77215.81 |
| EFG_BACHD_1 | Q9Z9L7 | 4.89 | 76888.86 |
| EFG_EHRRG_1 | Q5FFE7 | 5.2 | 76041.59 |
| EFG_BACSK_1 | Q5WLR5 | 4.82 | 76401.33 |
| EFG_THEP3_1 | B0KCJ7 | 5.1 | 76672.74 |
| EFG_RHIL3_1 | Q1MIE4 | 5.16 | 77775.7 |
| EFG_SALRD_1 | Q2S3R7 | 4.5 | 79104.62 |
| EFG_CHLP8_1 | B3QR64 | 5.19 | 77958.89 |
| EFG_RHIRD_1 | P70782 | 5.13 | 78044.87 |
| EFG_SALTY_1 | P0A1H3 | 5.17 | 77468.02 |
| EFG_FRATH_1 | Q2A5H2 | 4.97 | 77730.37 |
| EFG_UREU1_1 | B5ZC32 | 5.41 | 76374.82 |
| EFG_STRPQ_1 | P0DA85 | 4.82 | 76397.53 |
| EFG_VIBVU_1 | Q8DCQ8 | 4.88 | 77412.86 |
| EFG_GEOLS_1 | B3E7T2 | 5.04 | 76625.49 |
| EFG_ERYLH_1 | Q2N9A7 | 4.8 | 78690.02 |
| EFG_STRP7_1 | C1CB46 | 4.86 | 76831 |
| EFG_NITSB_1 | A6Q1M7 | 5.08 | 77104.9 |
| EFG_RHOPB_1 | Q211E5 | 5.24 | 75857.77 |
| EFG_OCHA4_1 | A6X0B5 | 5.06 | 76498.2 |
| EFG_BAUCH_1 | Q1LSY5 | 5.54 | 78844.47 |
| EFG_STRS2_1 | A4VYX6 | 4.88 | 76710.79 |
| EFG_PHOLL_1 | Q7N9B2 | 5.04 | 77698.42 |
| EFG_HERAR_1 | A4G9U1 | 5.4 | 77453.59 |
| EFG_BACFN_1 | Q5L8A7 | 5.09 | 77508.19 |
| EFG_SACEN_1 | A4FPM8 | 4.97 | 77308.83 |
| EFG_COREF_1 | Q8FS85 | 4.8 | 77480.82 |
| EFG_EHRCR_1 | Q2GFN5 | 5.25 | 76242.88 |
| EFG_PSEU2_1 | Q4ZMP1 | 5.28 | 77141.92 |
| EFG_KOCRD_1 | B2GIL1 | 4.82 | 77514.7 |
| EFG_SALPA_1 | Q5PIW3 | 5.17 | 77468.02 |
| EFG_LACCB_1 | B3WAM2 | 4.76 | 76860.52 |
| EFG_CHLFF_1 | Q253F1 | 5.25 | 76647.71 |
| EFG_THEPX_1 | B0K5P0 | 5.1 | 76672.74 |
| EFG_MYCGE_1 | P47335 | 5.7 | 76538.54 |
| EFG_THIDA_1 | Q3SLQ2 | 5.35 | 76918.07 |
| EFG_SHIBS_1 | Q31VU9 | 5.24 | 77581.31 |
| EFG_CHLSY_1 | B9LJC8 | 5.28 | 78312.65 |
| EFG_SHIFL_1 | Q83JC3 | 5.24 | 77418.05 |
| EFG_GEMAT_1 | C1A6Q2 | 5.55 | 78556.76 |
| EFG_STRS7_1 | C0MF25 | 4.82 | 76601.82 |
| EFG_BIFLD_1 | B3DT30 | 4.82 | 78136.58 |
| EFG_SODGM_1 | Q2NQL6 | 5.22 | 77710.41 |
| EFG_LACP3_1 | Q034X8 | 4.76 | 76819.47 |
| EFG_TOLAT_1 | C4LBU4 | 5.28 | 77261.97 |
| EFG_NOCSJ_1 | A1SNN6 | 4.95 | 77517.83 |
| EFG_CHLTB_1 | B0BC74 | 5.21 | 76493.52 |
| EFG_XANAC_1 | Q8PNS6 | 5.19 | 77635.33 |
| EFG_PERMH_1 | C0QQM0 | 4.97 | 76817.06 |
| EFG_BRUSI_1 | B0CH35 | 5.08 | 76245.99 |
| EFG_HAEIN_1 | P43925 | 5.04 | 77132.45 |
| EFG_BUCAI_1 | P57593 | 5.84 | 78182.61 |
| EFG_CLOBJ_1 | C1FMV4 | 5.02 | 76312.2 |
| EFG_CAMC1_1 | A8Z6I6 | 5.08 | 77101.87 |
| EFG_MESFL_1 | Q6F0J4 | 5.08 | 76129.66 |
| EFG_RUBXD_1 | Q1AU26 | 5.08 | 79416.1 |
| EFG_HISS2_1 | B0UWC4 | 5.15 | 77147.8 |
| EFG_CAMJJ_1 | A1VYJ8 | 5.07 | 76745.33 |
| EFG_STRA3_1 | Q8E3E7 | 4.78 | 76598.65 |
| EFG_CORDI_1 | Q6NJD6 | 4.85 | 77325.78 |
| EFG_EHRCJ_1 | Q3YSU3 | 5.21 | 76168.61 |
| EFG_YERPS_1 | Q664R6 | 5.29 | 77537.35 |
| EFG_METRJ_1 | B1LWS3 | 5.22 | 76117.58 |
| EFG_NEOSM_1 | Q2GD82 | 5.21 | 76136.08 |
| EFG_MICLU_1 | P09952 | 5.06 | 77424.96 |
| EFG_MYCA9_1 | B1MGH8 | 4.89 | 77043.38 |
| EFG_RHIE6_1 | B3PWR8 | 5.13 | 77793.71 |
| EFG_CUTAK_1 | Q6A6L5 | 4.91 | 76604.89 |
| EFG_MYCBT_1 | C1AL17 | 4.96 | 77202.53 |
| EFG_CHLPB_1 | B3EP64 | 5.29 | 76886.68 |
| EFG_FRAP2_1 | B0U0Z1 | 4.94 | 77681.37 |
| EFG_MYCHJ_1 | Q4AAQ6 | 5.31 | 77408.1 |
| EFG_NOVAD_1 | Q2G8Y3 | 5.03 | 76180.53 |
| EFG_GEOKA_1 | Q5L400 | 5.09 | 77022.54 |
| EFG_GEOSW_1 | C5D3R4 | 5.09 | 77210.75 |
| EFG_PARL1_1 | A7HWQ8 | 5.11 | 76848.67 |
| EFG_STRT1_1 | Q5LY21 | 4.87 | 76610.77 |
| EFG_PETMO_1 | A9BHA8 | 4.99 | 77662.62 |
| EFG_CITK8_1 | A8AQM8 | 5.21 | 77460.15 |
| EFG_HAES1_1 | Q0I537 | 5.15 | 77147.8 |
| EFG_XYLF2_1 | B2IA64 | 5.55 | 78087.54 |
| EFG_PROM1_1 | A2C4U6 | 4.93 | 75598.26 |
| EFG_BACAC_1 | C3LJ79 | 4.9 | 76307.53 |
| EFG_RICPU_1 | C4K1P6 | 5.22 | 77722.42 |
| EFG_YERPA_1 | Q1C2U0 | 5.29 | 77537.35 |
| EFG_BACC2_1 | B7IT16 | 4.9 | 76326.53 |
| EFG_THEMA_1 | P38525 | 5.17 | 77846.47 |
| EFG_BACP2_1 | A8F981 | 4.81 | 76470.45 |
| EFG_NITMU_1 | Q2YB00 | 5.35 | 77186.04 |
| EFG_LACH4_1 | A8YXK3 | 4.93 | 76821.83 |
| EFG_DEIRA_1 | Q9RXK5 | 5 | 76798.14 |
| EFG_DESVH_1 | Q72CI3 | 5.24 | 76009.98 |
| EFG_OCEIH_1 | Q8ETY5 | 4.84 | 76793.77 |
| EFG_LAWIP_1 | Q1MPS9 | 5.2 | 76401.3 |
| EFG_ONYPE_1 | Q6YQV9 | 5.35 | 76257.91 |
| EFG1_PSEAE_1 | Q9HWD2 | 5.06 | 77784.42 |
| EFG_GLUOX_1 | Q5FUP6 | 5.15 | 77090.82 |
| EFG_DICNV_1 | A5EX85 | 5.11 | 77503.4 |
| EFG_SPHWW_1 | A5V605 | 5.03 | 76188.66 |
| EFG_BRUO2_1 | A5VR09 | 5.05 | 76251 |
| EFG_MYCTA_1 | A5U070 | 4.96 | 77202.53 |
| EFG_BURM9_1 | A2S7H3 | 5.33 | 77446.38 |
| EFG_LYSSC_1 | B1HMZ1 | 5 | 76432.49 |
| EFG_SYNR3_1 | A5GW13 | 4.87 | 75271.91 |
| EFG_CLOTH_1 | A3DIZ9 | 5.17 | 77635.69 |
| EFG_THEAB_1 | B7IHU3 | 5.23 | 77819.46 |
| EFG_CAUVN_1 | B8H414 | 5.2 | 76344.16 |
| EFG_PSYA2_1 | Q4FQG5 | 4.93 | 79141.07 |
| EFG_MOOTA_1 | Q2RFP4 | 5.05 | 76842.56 |
| EFG_PSYWF_1 | A5WGL0 | 4.97 | 79388.28 |
| EFG_MYCAP_1 | A5IZ33 | 5.47 | 77376.39 |
| EFG_LACDB_1 | Q04C17 | 4.81 | 76554.59 |
| EFG_RHIME_1 | Q92QH2 | 5.23 | 77807.83 |
| EFG_BACVZ_1 | A7Z0N4 | 4.79 | 76534.61 |
| EFG_SHIDS_1 | Q32B26 | 5.24 | 77581.31 |
| EFG_LACPL_1 | Q88XY8 | 4.81 | 77012.7 |
| EFG_TROWT_1 | Q83FP1 | 5.19 | 77310.84 |
| EFG_BARQU_1 | Q6FZB9 | 5.12 | 76594.43 |
| EFG_PEDPA_1 | Q03EB4 | 4.72 | 76752.59 |
| EFG_SPHAL_1 | Q1GP96 | 5.02 | 76700.15 |
| EFG_DEHM1_1 | Q3Z983 | 5.3 | 76420.85 |
| EFG_FRACC_1 | Q2JFH9 | 5 | 76734.94 |
| EFG_NOCFA_1 | Q5YPG3 | 4.92 | 77019.57 |
| EFG_LACRD_1 | A5VLK8 | 4.77 | 76770.79 |
| EFG_PARD8_1 | A6LEJ3 | 5.08 | 78377.42 |
| EFG_WOLTR_1 | Q5GSU1 | 5.23 | 76013.56 |
| EFG_PECAS_1 | Q6CZW5 | 5.23 | 77480.1 |
| EFG_BORPE_1 | Q7VTD5 | 5.15 | 77115.72 |
| EFG2_SHESR_1 | Q0HRE9 | 4.89 | 76806.57 |
| EFG1_PSEHT_1 | Q3ILP5 | 4.89 | 77485.26 |
| EFG1_RALSO_1 | Q8XV10 | 5.31 | 77510.61 |
| EFG2_SYNY3_1 | P74228 | 4.94 | 75431.16 |
| EFG1_STRCO_1 | P40173 | 4.97 | 77657.2 |
| EFG1_BDEBA_1 | Q6MJ13 | 5.44 | 77090.44 |
| EFG1_BORBR_1 | Q7WRC7 | 5.15 | 77087.67 |
| EFG1_TRIEI_1 | Q118Z3 | 4.96 | 75988.95 |
| EFG1_BORPA_1 | Q7W2F8 | 5.12 | 77113.7 |
| EFG1_BURPS_1 | Q63WJ7 | 5.29 | 77807.61 |
| EFG2_ANADE_1 | Q2IK81 | 5.65 | 76971.97 |
| EFG2_GEOMG_1 | Q39SN2 | 5.22 | 74959.67 |
| EFG2_GEOSL_1 | Q748Y8 | 5.05 | 76201.06 |
| EFG_CHLTE_1 | Q8KAG9 | 5.16 | 77951.87 |
| EFG_ANAMF_1 | B9KHV3 | 5.15 | 75536.69 |
| EFG_PHYAS_1 | B1VAM2 | 5.56 | 76234.08 |
| EFG_GRABC_1 | Q0BSG5 | 5.37 | 76534.05 |
| EFG_ANOFW_1 | B7GJ64 | 5.04 | 77141.66 |
| EFG_AQUAE_1 | O66428 | 5.32 | 78398.11 |
| EFG_CLAM3_1 | A5CUB7 | 5.02 | 77471.13 |
| EFG_ARCB4_1 | A8EW86 | 5.11 | 77599.7 |
| EFG_CLOAB_1 | Q97EH4 | 4.96 | 76403.08 |
| EFG_RICRH_1 | Q8KTB7 | 5.15 | 77307.82 |
| EFG_CALS8_1 | A4XI36 | 5.15 | 77244.59 |
| EFG_STACT_1 | B9DKV7 | 4.73 | 76835.68 |
| EFG_CLOP1_1 | Q0TMP3 | 4.85 | 76003.35 |
| EFG_HYDCU_1 | Q31IY5 | 4.83 | 77458.87 |
| EFG_IDILO_1 | Q5QWB4 | 4.96 | 78014.84 |
| EFG_PSYCK_1 | Q1Q8P1 | 4.93 | 79155.1 |
| EFG_CHLAD_1 | B8G6S9 | 5.37 | 78414.83 |
| EFG_RENSM_1 | A9WSW6 | 4.98 | 77576.22 |
| EFG_DECAR_1 | Q47JA6 | 5.3 | 77112.19 |
| EFG_MYCCT_1 | Q2SSW9 | 5.37 | 76264.98 |
| EFG_LACLA_1 | Q9CDG1 | 4.74 | 77956.04 |
| EFG_DESAP_1 | B1I1I5 | 5.68 | 76484.55 |
| EFG_SINMW_1 | A6U856 | 5.14 | 77889.73 |
| EFG_ANAVT_1 | Q3MDM4 | 5.13 | 76052.61 |
| EFG_POLAQ_1 | A4SUV8 | 5.33 | 77552.97 |
| EFG_MYCSK_1 | A1UBL0 | 4.95 | 77367.75 |
| EFG_LEPIC_1 | Q72VM5 | 5.54 | 79001.24 |
| EFG_PORGI_1 | Q7MTL1 | 5 | 78262.08 |
| EFG_HAEPS_1 | B8F7Z4 | 5.06 | 77071.35 |
| EFG_ECO5E_1 | B5YTP7 | 5.24 | 77581.31 |
| EFG_LISIN_1 | Q927I5 | 4.84 | 76821.73 |
| EFG_SYMTH_1 | Q67JU0 | 5.2 | 76871.8 |
| EFG_MACCJ_1 | B9E8Q1 | 4.84 | 76428.38 |
| EFG_CAMLR_1 | B9KFH1 | 5.1 | 76775.36 |
| EFG_STRAW_1 | Q82DQ1 | 4.98 | 77793.48 |
| EFG_CARRP_1 | Q05FI2 | 9.07 | 77204.97 |
| EFG_COXB1_1 | B6J5C9 | 5.07 | 77747.22 |
| EFG_STRMK_1 | B2FQ42 | 5.22 | 77854.94 |
| EFG_THENN_1 | B9K883 | 5.2 | 78033.69 |
| EFG_ENTFA_1 | Q839G9 | 4.8 | 76678.72 |
| EFG_CHLL7_1 | Q3B6G4 | 5.28 | 78074.94 |
| EFG_LACGA_1 | Q046C7 | 4.91 | 76947.94 |
| EFG_BACTN_1 | Q8A474 | 5.1 | 77587.39 |
| EFG_BARHE_1 | Q8KQB3 | 5.12 | 76333.11 |
| EFG_SINFN_1 | C3MAX7 | 5.17 | 77786.72 |
| EFG_LACSS_1 | Q38UQ9 | 4.71 | 76923.69 |
| EFG_LARHH_1 | C1DAR4 | 5.1 | 77047.79 |
| EFG_BART1_1 | A9IW31 | 5.19 | 76483.48 |
| EFG_ORITI_1 | B3CTE7 | 5.5 | 78202.56 |
| EFG_PARUW_1 | Q6MER8 | 5.51 | 77085.34 |
| EFG_BLOPB_1 | Q492B1 | 5.8 | 78490.71 |
| EFG_MYCS5_1 | Q4A703 | 5.28 | 77259.96 |
| EFG_LEPBL_1 | Q04Y01 | 5.54 | 78956.13 |
| EFG_BRUME_1 | Q8YHP3 | 5.05 | 76295.06 |
| EFG_XANOM_1 | Q2NZY2 | 5.16 | 77608.3 |
| EFG_LEUCK_1 | B1MW21 | 4.66 | 77332.3 |
| EFG_SULDN_1 | Q30TP3 | 5.03 | 76943.4 |
| EFG_HAMD5_1 | C4K4F9 | 5.51 | 78039.18 |
| EFG_RICTY_1 | Q8KTB2 | 5.15 | 77652.17 |
| EFG_CAMFF_1 | A0RQI0 | 5.07 | 76771.67 |
| EFG_STAHJ_1 | Q4L3K8 | 4.78 | 76772.47 |
| EFG_ROSS1_1 | A5USJ2 | 5.28 | 77928.13 |
| EFG_METC4_1 | B7L0Q8 | 5.2 | 76171.72 |
| EFG_NITEC_1 | Q0AIJ8 | 5.38 | 76925.71 |
| EFG_MYCA1_1 | A0QL36 | 4.98 | 77232.61 |
| EFG_NITEU_1 | Q82T70 | 5.29 | 77098.03 |
| EFG_RHOOB_1 | C1AYS4 | 4.9 | 76871.41 |
| EFG_DESHD_1 | B8G1W3 | 5.07 | 76241.79 |
| EFG_MYCMM_1 | B2HSL2 | 4.96 | 76965.37 |
| EFG_MYCMO_1 | Q6KHS5 | 5.71 | 76915.86 |
| EFG_VARPS_1 | C5CP58 | 5.36 | 77231.27 |
| EFG_OLICO_1 | B6JET0 | 5.37 | 75687.44 |
| EFG_ARTPT_1 | P13550 | 5.02 | 76775.45 |
| EFG_PROA2_1 | B4S5N0 | 5.36 | 77726.76 |
| EFG_RICPR_1 | P41084 | 5.21 | 77750.35 |
| EFG_HELHP_1 | Q7VJ85 | 5.18 | 76907.92 |
| EFG_LISW6_1 | A0ALY9 | 4.84 | 76835.71 |
| EFG_RICRS_1 | A8GQV7 | 5.19 | 77682.27 |
| EFG_STAEQ_1 | Q5HRK5 | 4.8 | 76745.44 |
| EFG_METNO_1 | B8IS82 | 5.25 | 76171.84 |
| EFG_METPB_1 | B1ZLK1 | 5.2 | 76146.65 |
| EFG_SALA4_1 | B5F8F8 | 5.17 | 77599.22 |
| EFG_PSEPF_1 | Q3K5Y5 | 5.25 | 77252.97 |
| EFG_STRGC_1 | A8AUR6 | 4.88 | 76775.95 |
| EFG_THEFY_1 | Q47LJ0 | 4.84 | 77360.68 |
| EFG_CHESB_1 | Q11HP9 | 5.12 | 77267.51 |
| EFG_CHLL2_1 | B3EH94 | 5.48 | 78030.36 |
| EFG_EXIS2_1 | B1YGU7 | 4.91 | 76364.57 |
| EFG_LACP7_1 | A9KRZ3 | 5.08 | 78188.93 |
| EFG_RHORT_1 | Q2RQV7 | 5.18 | 76157.12 |
| EFG_BEII9_1 | B2IK59 | 5.24 | 76325.15 |
| EFG_PAEAT_1 | A1R8V0 | 4.97 | 77335.92 |
| EFG_WOLPP_1 | B3CLA3 | 5.27 | 76474.1 |
| EFG_PASMU_1 | P57938 | 5.07 | 77186.49 |
| EFG_CHRVO_1 | Q7NQF0 | 5.23 | 77007.91 |
| EFG_XANC8_1 | Q4URD6 | 5.19 | 77621.3 |
| EFG_LISMC_1 | C1KZK7 | 4.84 | 76849.74 |
| EFG_SULSY_1 | B2V7L6 | 5.33 | 77365.69 |
| EFG_SYNE7_1 | Q31PV4 | 4.93 | 75909.65 |
| EFG_HELPH_1 | Q1CS71 | 5.28 | 77052.11 |
| EFG_MAGSA_1 | Q2W2I8 | 5.1 | 76624.23 |
| EFG_ROSCS_1 | A7NR66 | 5.28 | 77688.84 |
| EFG_HERA2_1 | A9B746 | 5.14 | 77921.02 |
| EFG_CLOTE_1 | Q890N8 | 5.09 | 77154.42 |
| EFG_RICB8_1 | A8GV17 | 5.32 | 77489.9 |
| EFG_AROAE_1 | Q5P335 | 5.39 | 77541.08 |
| EFG_XANP2_1 | A7IFX8 | 5.21 | 75964.79 |
| EFG_RICHE_1 | Q8KTB4 | 5.33 | 77682.38 |
| EFG_MYCUA_1 | A0PM41 | 4.96 | 76938.34 |
| EFG_CALS4_1 | Q8R7V1 | 5.11 | 76674.75 |
| EFG_MAGMM_1 | A0L5X0 | 4.96 | 76616.41 |
| EFG_RICS2_1 | Q8KTB8 | 5.26 | 77651.3 |
| EFG_BACCN_1 | A7GK17 | 4.88 | 76482.61 |
| EFG_RUTMC_1 | A1AVJ7 | 4.96 | 77656.53 |
| EFG_TERTT_1 | C5BQ43 | 4.86 | 77473.04 |
| EFG_PSEU5_1 | A4VHM7 | 5.03 | 77850.35 |
| EFG_SALNS_1 | B4SUU6 | 5.17 | 77599.22 |
| EFG_THESQ_1 | B1LBP3 | 5.14 | 78242.87 |
| EFG_SALTI_1 | P0A1H4 | 5.17 | 77468.02 |
| EFG_LACJO_1 | Q74L90 | 4.91 | 76916.88 |
| EFG_FINM2_1 | B0S0I4 | 4.78 | 76591.78 |
| EFG_THIDL_1 | O50565 | 5.45 | 78146.87 |
| EFG_NITHX_1 | Q1QN33 | 5.42 | 75693.54 |
| EFG_FRAAA_1 | Q0RRS4 | 4.95 | 76774.76 |
| EFG_MYCLE_1 | P30767 | 5.14 | 77160.67 |
| EFG_DESRM_1 | A4J108 | 5.18 | 76378.95 |
| EFG_BIFA0_1 | B8DTV6 | 4.74 | 78550.92 |
| EFG_PEPD6_1 | Q18CF4 | 4.91 | 75896.47 |
| EFG1_SHEDO_1 | Q12SW2 | 4.94 | 76994.36 |
| EFG1_SHEFN_1 | Q089Q7 | 5 | 76927.39 |
| EFG2_SYNWW_1 | Q0AUH7 | 5.25 | 77316.22 |
| EFG2_VIBCH_1 | Q9KPM5 | 5.02 | 76482.18 |
| EFG2_VIBPA_1 | Q87M30 | 4.81 | 76367.45 |
| EFG3_MYXXD_1 | Q1D513 | 5.9 | 75745.6 |
| EFG2_COLP3_1 | Q47UW3 | 4.73 | 76936.31 |
| EFG1_METCA_1 | Q60BD3 | 5.27 | 76999.64 |
| EFG1_PARXL_1 | Q13UU8 | 5.38 | 77747.57 |
| EFG1_POLSJ_1 | Q12GX4 | 5.36 | 77440.86 |
| EFG2_SACD2_1 | Q21M87 | 5.02 | 76678.3 |
| EFG2_SHEON_1 | Q8EIJ7 | 4.91 | 76662.48 |
| EFG_ALKOO_1 | A8MLD7 | 5.07 | 76370.09 |
| EFG_BRASB_1 | A5ELN0 | 5.34 | 75415.11 |
| EFG_AMOA5_1 | B3EUF3 | 5.29 | 78521.87 |
| EFG_GLOVI_1 | Q7NEF2 | 5.08 | 77642.59 |
| EFG_MYCPN_1 | P75544 | 5.53 | 76499.55 |
| EFG_CHRSD_1 | Q1R0H8 | 4.85 | 77994.42 |
| EFG_BRUAB_1 | Q57CQ5 | 5.05 | 76235.01 |
| EFG_PHYMT_1 | B3QZH4 | 5.98 | 77025.56 |
| EFG_DINSH_1 | A8LM45 | 4.85 | 77845.33 |
| EFG_CLOB8_1 | A6LPQ8 | 5 | 75866.41 |
| EFG_AZOSB_1 | A1KB30 | 5.31 | 77394.76 |
| EFG_CLOCE_1 | B8I5N7 | 5.07 | 76441.18 |
| EFG_CAMC5_1 | A7GZJ4 | 5.15 | 77279.03 |
| EFG_NEIG2_1 | B4RQX2 | 5.08 | 77172.78 |
| EFG_KINRD_1 | A6W5T4 | 5.02 | 76935.65 |
| EFG_CORU7_1 | B1VET0 | 4.83 | 77932.52 |
| EFG_THEM4_1 | A6LLL0 | 5.04 | 77598.05 |
| EFG_SALHS_1 | B4TKM1 | 5.17 | 77599.22 |
| EFG_LACAC_1 | Q5FM92 | 4.93 | 76853.87 |
| EFG_CYTH3_1 | Q11QB0 | 5.31 | 77864.92 |
| EFG_EXISA_1 | C4KZQ0 | 4.88 | 76518.66 |
| EFG_BACWK_1 | A9VP74 | 4.92 | 76326.57 |
| EFG_SHISS_1 | Q3YWT2 | 5.24 | 77581.31 |
| EFG_NOSS1_1 | Q8YP62 | 5.08 | 76144.81 |
| EFG_UREP2_1 | B1AJG4 | 5.46 | 76429.94 |
| EFG_OENOB_1 | Q04ED6 | 4.82 | 77965.01 |
| EFG_BEUC1_1 | C5C0J4 | 4.97 | 77613.43 |
| EFG_PHEZH_1 | B4R8L3 | 5.19 | 76214.97 |
| EFG_PSE14_1 | Q48D33 | 5.21 | 77228.95 |
| EFG_JANMA_1 | A6T3K7 | 5.33 | 77379.54 |
| EFG_SALAR_1 | A9MN39 | 5.14 | 77600.2 |
| EFG_CORJK_1 | Q4JT40 | 4.84 | 77953.47 |
| EFG_SALDC_1 | B5FJM0 | 5.17 | 77599.22 |
| EFG_ERWT9_1 | B2VK36 | 5.25 | 77230.96 |
| EFG_CHLMU_1 | Q9PJV6 | 5.18 | 76521.65 |
| EFG_NITOC_1 | Q3J8R1 | 5.35 | 77197.99 |
| EFG_BACV8_1 | A6KYJ7 | 5.17 | 77456.19 |
| EFG_RHOBA_1 | Q7URV2 | 4.97 | 77788.99 |
| EFG_CHLPN_1 | Q9Z802 | 5.23 | 76587.57 |
| EFG_MYCPA_1 | Q73SD2 | 4.98 | 77232.61 |
| EFG_MYCPE_1 | Q8EX19 | 5.46 | 75995.78 |
| EFG_FUSNN_1 | Q8R602 | 5.12 | 77132.18 |
| EFG_RHOS4_1 | Q3J5S5 | 5.02 | 77719.74 |
| EFG_BIFAA_1 | A1A0T0 | 4.68 | 78465.66 |
| EFG_BLOFL_1 | Q7VRN9 | 5.83 | 79449.01 |
| EFG2_SYNAS_1 | Q2LUL6 | 5.42 | 74958.01 |
| EFG1_BORA1_1 | Q2L2H1 | 5.19 | 77127.88 |
| EFG1_BORAP_1 | Q0SMX0 | 5.56 | 77340.71 |
| EFG1_BORBP_1 | Q660Y4 | 5.56 | 77332.7 |
| EFG2_ALIF1_1 | Q5E7H2 | 4.77 | 76048.14 |
| EFG1_CUPNJ_1 | Q46WE0 | 5.24 | 77370.42 |
| EFG1_PELCD_1 | Q3A834 | 5.56 | 76453.3 |
| EFG_LEGPH_1 | Q5ZYP6 | 5.35 | 77026.07 |
| EFG_MYCPU_1 | Q98QD8 | 5.6 | 77009.81 |
| EFG_RICAE_1 | C3PMH0 | 5.22 | 77720.45 |
| EFG_BRUC2_1 | A9M5Q3 | 5.05 | 76265.03 |
| EFG_LEUMM_1 | Q03ZQ2 | 4.65 | 77569.54 |
| EFG_RICM5_1 | A8F0P0 | 5.22 | 77400.96 |
| EFG_RICMO_1 | Q8KTB6 | 5.26 | 77706.47 |
| EFG_SULNB_1 | A6Q6I6 | 4.89 | 77449.97 |
| EFG_HELMI_1 | B0TC53 | 5.26 | 76492.28 |
| EFG_RUEPO_1 | Q5LMR4 | 4.93 | 77798.63 |
| EFG_HYDS0_1 | B4U741 | 5.23 | 76904.28 |
| EFG_CORGB_1 | A4QBG9 | 4.81 | 77849.38 |
| EFG_BACLD_1 | Q65PB0 | 4.83 | 76315.14 |
| EFG_LACBA_1 | Q03PV4 | 4.73 | 77127.83 |
| EFG_CROS8_1 | A7MKJ6 | 5.24 | 77680.34 |
| EFG_LACF3_1 | B2GDX1 | 4.72 | 76352.21 |
| EFG_RHOJR_1 | Q0SFF3 | 4.89 | 76885.44 |
| EFG_NOSP7_1 | B2J5B0 | 5.18 | 76041.71 |
| EFG_UNCTG_1 | B1GZ80 | 5.37 | 76921.62 |
| EFG_PARDP_1 | A1B023 | 4.93 | 78389.24 |
| EFG_PELUB_1 | Q4FLL6 | 5.21 | 76606.34 |
| EFG2_TREPA_1 | O83464 | 5.85 | 75801.95 |
| EFG1_TREDE_1 | Q73R08 | 5.17 | 75414.07 |
| EFG1_BORBU_1 | O30913 | 5.46 | 77572.85 |
| EFG1_BURCA_1 | Q1BU86 | 5.31 | 77633.49 |
| EFG1_BURL3_1 | Q39KH0 | 5.39 | 77420.51 |
| EFG1_BURTA_1 | Q2T0I7 | 5.36 | 77922.71 |
| EFG1_DESPS_1 | Q6ASC7 | 4.92 | 76905.27 |
| EFG_BREBN_1 | C0ZIH5 | 5.03 | 76320.05 |
| EFG_LEIXX_1 | Q6ACY9 | 5.05 | 77063.61 |
| EFG_GRAFK_1 | A0M5A0 | 4.91 | 77673.2 |
| EFG_APPPP_1 | Q9ZEU4 | 6.17 | 77226.77 |
| EFG_AQUPY_1 | P46211 | 5.22 | 78157.71 |
| EFG_RICPA_1 | Q8KTB9 | 5.26 | 77819.56 |
| EFG_HELAH_1 | Q17VN9 | 5.25 | 77106.16 |
| EFG_PROMH_1 | B4EYV7 | 5.07 | 77884.63 |
| EFG_MARHV_1 | A1TYJ4 | 4.94 | 77803.37 |
| EFG_ROSDO_1 | Q160Y3 | 4.82 | 77838.51 |
| EFG_PSELT_1 | A8F4Q8 | 5.22 | 77364.29 |
| EFG_CARHZ_1 | Q3A9R2 | 5.26 | 76970.19 |
| EFG_SALAI_1 | A8M532 | 5 | 76483.04 |
| EFG_SALCH_1 | Q57J26 | 5.17 | 77599.22 |
| EFG_STRGG_1 | B1W417 | 4.96 | 77574.07 |
| EFG_KORVE_1 | Q1ISC5 | 5.55 | 77037.12 |
| EFG_CHLCV_1 | Q824G0 | 5.31 | 76859.96 |
| EFG_FERNB_1 | A7HM55 | 5.07 | 77003.47 |
| EFG_LACS1_1 | Q1WVA0 | 4.71 | 76860.58 |
| EFG_MYCMS_1 | Q6MU82 | 5.29 | 76184.86 |
| EFG_WOLSU_1 | Q7MA53 | 5.18 | 76900.81 |
| EFG_PECCP_1 | C6DG80 | 5.16 | 77450.07 |
| EFG_SOLUE_1 | Q01W89 | 5.46 | 77340.55 |
| EFG_RICCK_1 | A8EXK1 | 5.21 | 76588.12 |
| EFG_AYWBP_1 | Q2NJ19 | 5.45 | 76273.88 |
| EFG_MYCVP_1 | A1T4L5 | 4.93 | 77013.29 |
| EFG_CALBD_1 | B9MQH0 | 5.23 | 77220.47 |
| EFG_CLONN_1 | A0PXU3 | 5.02 | 76158.8 |
| EFG_RUEST_1 | Q1GK42 | 4.81 | 77936.57 |
| EFG_CORA7_1 | C3PKP1 | 4.76 | 78161.4 |
| EFG_EDWI9_1 | C5BGM8 | 5.2 | 77472.04 |
| EFG_BACHK_1 | Q6HPR1 | 4.9 | 76335.58 |
| EFG_CAUSK_1 | B0SUQ6 | 5.19 | 76171.1 |
| EFG_SALG2_1 | B5RH09 | 5.17 | 77599.22 |
| EFG_ZYMMO_1 | Q5NQ66 | 4.99 | 76553.2 |
| EFG_MYCA5_1 | B3PME9 | 5.33 | 77770.69 |
| EFG_CYAP4_1 | B8HVR8 | 5.04 | 77471.58 |
| EFG_RHILO_1 | Q98N59 | 5.28 | 77208.28 |
| EFG_SALSV_1 | B4TXE8 | 5.17 | 77599.22 |
| EFG_SALTO_1 | A4XBP9 | 4.98 | 76567.26 |
| EFG_MYCGI_1 | A4T1R3 | 4.97 | 77254.7 |
| EFG_DEIGD_1 | Q1IX68 | 5.05 | 76528.04 |
| EFG_NITWN_1 | Q3SSW9 | 5.47 | 75752.73 |
| EFG_FRASN_1 | A8LC59 | 4.94 | 76675.65 |
| EFG_CHLT3_1 | B3QY21 | 5.56 | 78167.52 |
| EFG_GEOBB_1 | B5EFP7 | 5.11 | 76047.88 |
| EFG_WIGBR_1 | Q8D3H2 | 8.39 | 79351.01 |
| EFG_PELPB_1 | B4SBU4 | 5.42 | 77987.15 |
| EFG_RICAH_1 | A8GMA0 | 5.23 | 77568.32 |
| EFG_HAEDU_1 | Q7VNA2 | 5.04 | 77079.48 |
| EFG_POLNS_1 | B1XSP8 | 5.28 | 77262.62 |
| EFG_AZOC5_1 | A8IAT3 | 5.19 | 75960.53 |
| EFG_HALOH_1 | B8D0C1 | 4.73 | 76771.12 |
| EFG_NAUPA_1 | B9L7K0 | 5.09 | 77727.67 |
| EFG_HYPNA_1 | Q0BYB1 | 5.13 | 77822.76 |
| EFG_PSEMY_1 | A4XZ93 | 5.08 | 78892.56 |
| EFG_METS4_1 | B0UHX2 | 5.25 | 76342.98 |
| EFG_SALEP_1 | B5R297 | 5.17 | 77599.22 |
| EFG_DEIDV_1 | C1CXH0 | 5 | 76634.21 |
| EFG_DESAG_1 | Q30Z38 | 5.12 | 76328.12 |
| EFG1_PSEPK_1 | Q88QN8 | 5.13 | 78817.52 |
| EFG1_CUPMC_1 | Q1LI29 | 5.24 | 77520.61 |
| EFG1_HAHCH_1 | Q2S909 | 5 | 77031.66 |
| EFG1_PSEA6_1 | Q15YP4 | 4.78 | 76519.68 |
| EFG2_PHOPR_1 | Q6LST1 | 4.83 | 75816.35 |
| EFG_ALKMQ_1 | A6TWI5 | 4.93 | 76078.71 |
| EFG_BRADU_1 | Q89J81 | 5.32 | 75829.66 |
| EFG_ALTMD_1 | B4RSU5 | 4.87 | 76289.59 |
| EFG_GEOTN_1 | A4IJI6 | 5.04 | 77199.7 |
| EFG_ANAPZ_1 | Q2GJ60 | 5.07 | 75768.99 |
| EFG_LEPCP_1 | B1Y7G9 | 5.33 | 77183.43 |
| EFG_STRU0_1 | B9DVS2 | 4.87 | 76559.75 |
| EFG_RICCN_1 | Q92J93 | 5.22 | 77708.39 |
| EFG_RICFE_1 | Q8KTA8 | 5.27 | 77671.3 |
| EFG_NATTJ_1 | B2A4D6 | 4.8 | 77425.82 |
| EFG_METPP_1 | A2SLG0 | 5.43 | 77494.81 |
| EFG_JANSC_1 | Q28UW8 | 4.83 | 78293.98 |
| EFG_METSB_1 | B8ELG6 | 5.25 | 76289.06 |
| EFG_STRMU_1 | Q8DVV4 | 4.81 | 76663.94 |
| EFG_CHLCH_1 | Q3APH0 | 5.47 | 78050.21 |
| EFG_MYCGA_1 | Q7NAV3 | 5.54 | 76695.4 |
| EFG_CHLPM_1 | A4SCQ6 | 5.16 | 78044.71 |
| EFG_RHOE4_1 | C0ZVT6 | 4.92 | 76830.56 |
| EFG_VEREI_1 | A1WHC2 | 5.53 | 77283.5 |
| EFG_HALHL_1 | A1WVC5 | 4.91 | 77631.66 |
| EFG_SULMW_1 | A8Z666 | 8.34 | 78214.75 |
| EFG_STAS1_1 | Q49V57 | 4.76 | 76976.64 |
| EFG_METFK_1 | Q1H4P0 | 5.19 | 77264.19 |
| EFG_CHLAB_1 | Q5L6S5 | 5.22 | 76891.01 |
| EFG_THEP1_1 | A5IM80 | 5.15 | 78256.9 |
| EFG_RHOFT_1 | Q21RV5 | 5.39 | 77684.14 |
| EFG_VESOH_1 | A5CXN7 | 4.94 | 77728.62 |
| EFG_STRSV_1 | A3CQM2 | 4.86 | 76778.95 |
| EFG_PELTS_1 | A5D5I7 | 5.24 | 76548.23 |
| EFG_ACIC1_1 | A0LRL7 | 5.18 | 77371.12 |
| EFG_ACICJ_1 | A5FV42 | 5.26 | 76649.15 |
| EFG_AERHH_1 | A0KQ96 | 5.34 | 77555.47 |
| EFG_AGRFC_1 | Q8UE15 | 5.16 | 77985.93 |
| EFG_AGRVS_1 | B9JVN4 | 5.15 | 77859.78 |
| EFG_ALKEH_1 | Q0ABH8 | 4.89 | 77000.28 |
| EFG_ACIET_1 | B9MB70 | 5.21 | 77547.64 |
| EFG_ACISJ_1 | A1W2Q4 | 5.21 | 77576.64 |
| EFG_ACTP2_1 | A3N247 | 5.1 | 77278.69 |
| EF-4 | Accession number | pI | MW |
| LEPA_ECOLI_1 | P60785 | 5.4 | 66570.27 |
| LEPA_MYCTU_1 | P9WK97 | 5.55 | 72395.8 |
| LEPA_AQUAE_1 | O67618 | 6.13 | 67657.24 |
| LEPA_METFK_1 | Q1H2L5 | 5.43 | 66387.91 |
| LEPA_BACSU_1 | P37949 | 5.17 | 68586.92 |
| LEPA_BRADU_1 | Q89BJ8 | 5.55 | 66784.3 |
| LEPA_CAUVC_1 | Q9A9F4 | 5.31 | 66856.89 |
| LEPA_CLOTE_1 | Q892Q6 | 5.07 | 67729.73 |
| LEPA_GRABC_1 | Q0BRZ7 | 5.58 | 66367.27 |
| LEPA_BORBU_1 | O51115 | 6.15 | 68172.57 |
| LEPA_MARMM_1 | Q0ATE3 | 5.37 | 66708.8 |
| LEPA_MYCPU_1 | Q98QW3 | 7.11 | 67551.24 |
| LEPA_SYNP6_1 | Q5N390 | 5.14 | 67435.51 |
| LEPA_COXBU_1 | Q83BK3 | 6.64 | 66480.86 |
| LEPA_RHORT_1 | Q2RNY6 | 5.4 | 66419.56 |
| LEPA_BDEBA_1 | P60930 | 5.5 | 66815.48 |
| LEPA_MYCH2_1 | Q9ZHZ8 | 7.11 | 67341.08 |
| LEPA_MYCFP_1 | Q8GCP5 | 5.86 | 67682.96 |
| LEPA_GLUOX_1 | Q5FUC2 | 5.5 | 66365.53 |
| LEPA_HAEDU_1 | Q7VL73 | 5.23 | 66277.95 |
| LEPA_RHOP2_1 | Q2J2Y0 | 5.52 | 66769.22 |
| LEPA_FRACC_1 | Q2JDK2 | 5.42 | 70191.7 |
| LEPA_MANSM_1 | Q65VN2 | 5.35 | 66313.2 |
| LEPA_MYCPN_1 | P75498 | 7.59 | 68025.03 |
| LEPA_ACIBY_1 | B0VCT7 | 5.45 | 66657.93 |
| LEPA_BACHK_1 | Q6HDK2 | 5.14 | 67947.1 |
| LEPA_BURTA_1 | Q2SXT6 | 5.41 | 66153.18 |
| LEPA_CORA7_1 | C3PHY1 | 4.94 | 68300.25 |
| LEPA_COREF_1 | Q8FNA3 | 5.03 | 68461.39 |
| LEPA_AERHH_1 | A0KGF0 | 5.27 | 66025.93 |
| LEPA_HELPG_1 | B5ZAB8 | 5.74 | 66925.62 |
| LEPA_EHRRG_1 | Q5FHQ1 | 6.19 | 66807.26 |
| LEPA_ENT38_1 | A4WDE0 | 5.25 | 66579.38 |
| LEPA_ENTFA_1 | Q831Z0 | 4.99 | 68316.31 |
| LEPA_BIFA0_1 | B8DUL6 | 5.14 | 69430.26 |
| LEPA_MAGMM_1 | A0L631 | 5.62 | 66606.22 |
| LEPA_CAMJE_1 | Q9PNR1 | 5.64 | 66543.99 |
| LEPA_MESFL_1 | Q6F0Z2 | 5.56 | 67518.98 |
| LEPA_ALIF1_1 | Q5E312 | 5.1 | 65864.4 |
| LEPA_ALISL_1 | B6EKN1 | 5.21 | 65905.54 |
| LEPA_DESPS_1 | Q6AL53 | 5.17 | 66379.34 |
| LEPA_PSEAE_1 | Q9I5G8 | 5.43 | 66295.26 |
| LEPA_NOCFA_1 | Q5YZZ7 | 5.33 | 68399.28 |
| LEPA_NOSP7_1 | B2J0M4 | 5.28 | 67413.53 |
| LEPA_CHLTR_1 | O84067 | 5.95 | 67435.17 |
| LEPA_PSEHT_1 | Q3IDL4 | 5.78 | 66000.08 |
| LEPA_GEOTN_1 | A4IR35 | 5.32 | 68559.03 |
| LEPA_ORITI_1 | B3CRQ1 | 7.86 | 66988.51 |
| LEPA_ANAMM_1 | Q5PAX8 | 5.84 | 65994.9 |
| LEPA_PAEAT_1 | A1R6W8 | 5.58 | 68329.7 |
| LEPA_HAEIE_1 | A5UBC2 | 5.16 | 66314.1 |
| LEPA_BRUC2_1 | A9MCW5 | 5.31 | 66895 |
| LEPA_LEPBJ_1 | Q04SN9 | 5.48 | 66917.16 |
| LEPA_LEPBP_1 | B0SRL4 | 5.74 | 67090.57 |
| LEPA_PSYCK_1 | Q1QDV6 | 5.17 | 66443.55 |
| LEPA_PSYIN_1 | A1SSM6 | 5.76 | 65917.98 |
| LEPA_ACAM1_1 | B0C9R9 | 5.11 | 67153.12 |
| LEPA_RHOJR_1 | Q0SH84 | 5.52 | 69477.66 |
| LEPA_SALPC_1 | C0PYG5 | 5.4 | 66524.26 |
| LEPA_BACCR_1 | Q818E4 | 5.14 | 67947.98 |
| LEPA_NEIG1_1 | Q5F9P9 | 5.31 | 66002.14 |
| LEPA_BACFN_1 | Q5LC85 | 5.45 | 66515.85 |
| LEPA_SYNY3_1 | P74751 | 5.08 | 66968.21 |
| LEPA_STRA3_1 | P65273 | 4.99 | 68106.13 |
| LEPA_BACHD_1 | Q9KD76 | 4.97 | 68305.22 |
| LEPA_THEEB_1 | Q8DM20 | 5.45 | 67578.89 |
| LEPA_BARHE_1 | Q6G550 | 5.61 | 66924.29 |
| LEPA_CALS8_1 | A4XKA0 | 5.29 | 68039.77 |
| LEPA_CORU7_1 | B1VGK6 | 5.18 | 68692.52 |
| LEPA_AERS4_1 | A4SRD4 | 5.28 | 65967.75 |
| LEPA_CUPNH_1 | Q0K8N0 | 5.3 | 65911 |
| LEPA_CYAP4_1 | B8HLK8 | 5.2 | 67134.36 |
| LEPA_JANMA_1 | A6SXR0 | 5.41 | 65785.11 |
| LEPA_FRAAA_1 | Q0RP81 | 5.34 | 67375.44 |
| LEPA_CHLL7_1 | Q3B2V1 | 5.42 | 67862.24 |
| LEPA_CHLMU_1 | Q9PKX6 | 5.87 | 67379.05 |
| LEPA_LACJO_1 | P60790 | 5.09 | 68887.9 |
| LEPA_FRATF_1 | A7N9A4 | 5.42 | 65573.59 |
| LEPA_NITHX_1 | Q1QR19 | 5.6 | 67679.17 |
| LEPA_LACLS_1 | Q02Z80 | 4.95 | 67682.83 |
| LEPA_GEODF_1 | B9M4U5 | 5.38 | 67204.49 |
| LEPA_GEOKA_1 | Q5KWZ3 | 5.37 | 68462.78 |
| LEPA_PSEPF_1 | Q3KHM1 | 5.51 | 66160.13 |
| LEPA_ANASK_1 | B4UDQ7 | 5.6 | 67217.07 |
| LEPA_CLOB1_1 | A7FXL9 | 5.08 | 67158.19 |
| LEPA_ANOFW_1 | B7GKC4 | 5.36 | 68151.49 |
| LEPA_HAMD5_1 | C4K3Z1 | 6.43 | 66792.05 |
| LEPA_ARTS2_1 | A0JX50 | 5.57 | 68229.71 |
| LEPA_MYCMO_1 | Q6KHP1 | 6.01 | 67285.67 |
| LEPA_LISIN_1 | Q92BN4 | 5.04 | 67781.89 |
| LEPA_BUCAT_1 | B8D7F7 | 8.98 | 68607.04 |
| LEPA_PELUB_1 | Q4FNH3 | 5.95 | 66719.04 |
| LEPA_PHOLL_1 | Q7N1X3 | 5.34 | 66475.24 |
| LEPA_BURCA_1 | Q1BXU3 | 5.47 | 65894.82 |
| LEPA_LISMH_1 | B8DE32 | 5.04 | 67822.94 |
| LEPA_RHIE6_1 | B3PYC6 | 5.63 | 67453.74 |
| LEPA_SALA4_1 | B5F1G3 | 5.4 | 66538.28 |
| LEPA_STAAE_1 | A6QHC7 | 4.85 | 68175.08 |
| LEPA_MYCSK_1 | A1UIU2 | 5.63 | 70586.89 |
| LEPA_BURM7_1 | A3MM44 | 5.41 | 66221.26 |
| LEPA_PROM0_1 | A3PBD8 | 5.48 | 67581.86 |
| LEPA_BURP0_1 | A3NXL8 | 5.41 | 66221.26 |
| LEPA_STAEQ_1 | Q5HNW2 | 4.82 | 68275.14 |
| LEPA_NEIMF_1 | A1KT27 | 5.26 | 66002.09 |
| LEPA_YERPY_1 | B1JRC5 | 5.57 | 66709.47 |
| LEPA_RICCN_1 | Q92IQ1 | 5.8 | 67291.58 |
| LEPA_SHEON_1 | Q8EH83 | 5.42 | 65971.95 |
| LEPA_TREPA_1 | O83523 | 5.87 | 67622.58 |
| LEPA_SHEWM_1 | B1KI55 | 5.33 | 65633.53 |
| LEPA_UREU1_1 | B5ZBD4 | 6.42 | 67049.75 |
| LEPA_STRPD_1 | Q1JH37 | 5.15 | 68151.14 |
| LEPA_VIBC3_1 | A5F5G3 | 5.27 | 66029.93 |
| LEPA_XANCP_1 | Q8PB55 | 5.72 | 66430.58 |
| LEPA_BACSK_1 | Q5WHG5 | 5.14 | 68073.07 |
| LEPA_CORGL_1 | Q8NN68 | 5.02 | 68548.67 |
| LEPA_HELHP_1 | Q7VJZ1 | 5.42 | 67128.51 |
| LEPA_DECAR_1 | Q47EG0 | 5.37 | 66232.34 |
| LEPA_DEHM1_1 | Q3Z864 | 6.06 | 66928.34 |
| LEPA_METNO_1 | B8IMT0 | 5.54 | 66659.94 |
| LEPA_FLAJ1_1 | A5FLU8 | 5.3 | 66825.15 |
| LEPA_DEIGD_1 | Q1IXW5 | 5.1 | 66017.69 |
| LEPA_BLOFL_1 | Q7VRQ8 | 8.76 | 67528.02 |
| LEPA_LACBA_1 | Q03QU8 | 4.97 | 67882.49 |
| LEPA_ALKMQ_1 | A6TSM4 | 5.47 | 67494.81 |
| LEPA_MYCCT_1 | Q2SSF7 | 5.79 | 67700.53 |
| LEPA_BORPA_1 | Q7W5J4 | 5.51 | 65965.15 |
| LEPA_OCEIH_1 | Q8CXD0 | 5.01 | 67557.35 |
| LEPA_GLUDA_1 | A9HG78 | 5.57 | 66420.33 |
| LEPA_LEGPL_1 | Q5WVI1 | 6.31 | 67744.17 |
| LEPA_PARP8_1 | B2JFK0 | 5.42 | 66047.13 |
| LEPA_PSEU2_1 | Q4ZPD8 | 5.56 | 66245.32 |
| LEPA_PARUW_1 | Q6MEF3 | 6.17 | 67179.83 |
| LEPA_MYCLB_1 | B8ZUS2 | 5.25 | 71329.36 |
| LEPA_AROAE_1 | Q5P089 | 5.67 | 66097.26 |
| LEPA_LEPIC_1 | Q72QU8 | 5.56 | 67057.32 |
| LEPA_AYWBP_1 | Q2NJE6 | 8.94 | 67965.35 |
| LEPA_RUEST_1 | Q1GIV5 | 5.35 | 66850.84 |
| LEPA_PHYAS_1 | B1V9C5 | 9.11 | 67752.11 |
| LEPA_BURL3_1 | Q39I75 | 5.54 | 66043.2 |
| LEPA_RHILW_1 | B5ZXQ5 | 5.56 | 67395.62 |
| LEPA_PORGI_1 | Q7MV56 | 5.4 | 66354.47 |
| LEPA_RHOCS_1 | B6IUG3 | 5.55 | 66791.18 |
| LEPA_STAHJ_1 | Q4L6T4 | 4.84 | 68203.07 |
| LEPA_SHEAM_1 | A1S3X9 | 5.29 | 65841.59 |
| LEPA_STRAW_1 | Q82BZ3 | 5.32 | 68232.33 |
| LEPA_NITEC_1 | Q0AF67 | 5.3 | 66280.58 |
| LEPA_THEPX_1 | B0K3Y4 | 5.18 | 68010.52 |
| LEPA_SHEDO_1 | Q12KH9 | 5.63 | 66026.97 |
| LEPA_RICB8_1 | A8GW16 | 5.86 | 67144.42 |
| LEPA_RICCK_1 | A8EY28 | 6.03 | 67215.73 |
| LEPA_SHIB3_1 | B2TYI0 | 5.4 | 66570.27 |
| LEPA_SHIDS_1 | Q32CV2 | 5.4 | 66598.33 |
| LEPA_VIBPA_1 | Q87LN7 | 5.1 | 65914.59 |
| LEPA_WOLPM_1 | Q73HR8 | 5.51 | 66556.53 |
| LEPA_SYMTH_1 | Q67S76 | 5.29 | 67825.09 |
| LEPA_ACIET_1 | B9MDP9 | 5.42 | 66485.56 |
| LEPA_ACIF5_1 | B5EQB5 | 5.65 | 66938.98 |
| LEPA_CALS4_1 | Q8RB72 | 5.31 | 68165.79 |
| LEPA_BARQU_1 | Q6G1F5 | 5.85 | 66907.46 |
| LEPA_CAMFF_1 | A0RQX4 | 5.47 | 66000.36 |
| LEPA_ERWT9_1 | B2VI44 | 5.32 | 66587.32 |
| LEPA_ERYLH_1 | Q2N9U6 | 5.14 | 67316.06 |
| LEPA_MAGSA_1 | Q2W0F9 | 5.64 | 66399.31 |
| LEPA_EXIS2_1 | B1YKS5 | 5.17 | 67760 |
| LEPA_JANSC_1 | Q28LR4 | 5.57 | 66645.71 |
| LEPA_FLAPJ_1 | A6H1S4 | 5.66 | 66699.08 |
| LEPA_BORA1_1 | Q2KWY3 | 5.4 | 65706.84 |
| LEPA_DESAP_1 | B1I6E0 | 6.29 | 67546.97 |
| LEPA_DESRM_1 | A4J7F8 | 5.21 | 67233.7 |
| LEPA_GEOBB_1 | B5EB36 | 5.56 | 67012.49 |
| LEPA_LACP7_1 | A9KKU4 | 5.18 | 67508.88 |
| LEPA_NOSS1_1 | Q8YU48 | 5.23 | 67448.56 |
| LEPA_LARHH_1 | C1D7L2 | 5.38 | 66177.48 |
| LEPA_CITK8_1 | A8AD16 | 5.65 | 66604.53 |
| LEPA_CLAMS_1 | B0RCT0 | 5.21 | 67077.09 |
| LEPA_PASMU_1 | P57806 | 5.34 | 66211.03 |
| LEPA_HAHCH_1 | Q2SL35 | 5.46 | 66092.07 |
| LEPA2_LACPL_1 | Q88T65 | 6.47 | 65271.52 |
| LEPA_PSYWF_1 | A5WCD6 | 5.07 | 66767.79 |
| LEPA_RUBXD_1 | Q1AVV0 | 5.29 | 66672.45 |
| LEPA_CLOPE_1 | Q8XIS6 | 5.11 | 67049.22 |
| LEPA_ACIAD_1 | Q6F9B9 | 5.46 | 66815.05 |
| LEPA_SACD2_1 | Q21IH3 | 5.68 | 65613.92 |
| LEPA_SALAR_1 | A9MGX5 | 5.4 | 66574.36 |
| LEPA_PROA2_1 | B4S9B7 | 5.51 | 67316.37 |
| LEPA_RHOFT_1 | Q21XN4 | 5.4 | 66429.28 |
| LEPA_XYLF2_1 | B2I601 | 5.6 | 66713.71 |
| LEPA_STRP2_1 | Q04KB7 | 5.08 | 67608.75 |
| LEPA_SHESW_1 | A1RMC9 | 5.48 | 65946.86 |
| LEPA_SHIFL_1 | P60788 | 5.4 | 66570.27 |
| LEPA_SODGM_1 | Q2NS11 | 5.57 | 66352.22 |
| LEPA_VIBTL_1 | B7VK81 | 5.16 | 66100.03 |
| LEPA_XANOP_1 | B2SRY0 | 5.64 | 65748.73 |
| LEPA_HERAR_1 | A4G6S1 | 5.36 | 65470.85 |
| LEPA_HYDS0_1 | B4U6M2 | 5.95 | 67211.59 |
| LEPA_MARHV_1 | A1U2V8 | 5.35 | 66662.61 |
| LEPA_ALCBS_1 | Q0VP16 | 5.22 | 66453.4 |
| LEPA_KLEP7_1 | A6TCI3 | 5.39 | 66435.25 |
| LEPA_CHESB_1 | Q11AY3 | 5.39 | 66972.2 |
| LEPA_METPP_1 | A2SDH0 | 5.32 | 66962.03 |
| LEPA_CHLCH_1 | Q3ATB2 | 5.43 | 67715.14 |
| LEPA_METSB_1 | B8ENL1 | 5.62 | 66485.77 |
| LEPA_DESAD_1 | C6BST2 | 5.04 | 67050.1 |
| LEPA_FRAP2_1 | B0TW33 | 5.4 | 65445.23 |
| LEPA_CHLPM_1 | A4SFN5 | 5.46 | 67632.95 |
| LEPA_PSEP1_1 | A5W8F4 | 5.57 | 65801.66 |
| LEPA_BRUA2_1 | Q2YJP8 | 5.31 | 66906.99 |
| LEPA_LEIXX_1 | Q6AEB5 | 5.25 | 67521.81 |
| LEPA_PARPJ_1 | B2SZV7 | 5.41 | 66028.13 |
| LEPA_PSEU5_1 | A4VIX2 | 5.62 | 66299.29 |
| LEPA_PARXL_1 | Q13VM6 | 5.42 | 66051.16 |
| LEPA_BRUME_1 | Q8YDB8 | 5.31 | 66925.03 |
| LEPA_PECAS_1 | Q6D217 | 5.58 | 66652.57 |
| LEPA_BRUSI_1 | A9WW49 | 5.31 | 66895 |
| LEPA_PELPB_1 | B4SBG4 | 5.66 | 67729.26 |
| LEPA_CLOK5_1 | A5N6L8 | 5.69 | 67195.41 |
| LEPA_PEPD6_1 | Q182F4 | 5.19 | 67337.48 |
| LEPA_BACAA_1 | C3P8M5 | 5.14 | 67949.07 |
| LEPA_PHYMT_1 | B3QZT9 | 9.25 | 68533.05 |
| LEPA_MYCS5_1 | Q4A5S3 | 6.13 | 68349.88 |
| LEPA_SYNFM_1 | A0LI00 | 6.1 | 66703.7 |
| LEPA_RHILO_1 | Q98DV1 | 5.56 | 66576.63 |
| LEPA_BURM1_1 | A9ADE0 | 5.53 | 65888.89 |
| LEPA_SHEB9_1 | A9L5N4 | 5.5 | 65860.84 |
| LEPA_RICAE_1 | C3PMX3 | 5.87 | 67208.49 |
| LEPA_RICAH_1 | A8GMT1 | 6.03 | 67229.65 |
| LEPA_SHEPW_1 | B8CQJ7 | 5.15 | 65863.61 |
| LEPA_UREPA_1 | Q9PQG7 | 6.85 | 67043.96 |
| LEPA_SPHWW_1 | A5VB59 | 5.49 | 67450.56 |
| LEPA_WIGBR_1 | Q8D307 | 9.56 | 68258.56 |
| LEPA_WOLWR_1 | C0R5S3 | 5.52 | 66556.57 |
| LEPA_MYCGE_1 | P47384 | 8.43 | 67936.98 |
| LEPA_GEOSM_1 | C6DZ67 | 5.54 | 66982.41 |
| LEPA_LAWIP_1 | Q1MQF3 | 5.57 | 67905.35 |
| LEPA_CLOCE_1 | B8I3E6 | 5.15 | 67659.99 |
| LEPA2_RHOBA_1 | Q7UE01 | 5.6 | 66812.64 |
| LEPA_RALPJ_1 | B2U978 | 5.5 | 65843.95 |
| LEPA_RALSO_1 | Q8Y0I4 | 5.42 | 65832.86 |
| LEPA_POLNA_1 | A1VRT5 | 5.35 | 66006.87 |
| LEPA_STACT_1 | B9DNK4 | 4.93 | 67998.91 |
| LEPA_STAS1_1 | Q49Y26 | 4.87 | 68288.21 |
| LEPA_SALTI_1 | P0A1W5 | 5.4 | 66538.28 |
| LEPA_SALTY_1 | P0A1W4 | 5.4 | 66538.28 |
| LEPA_THEMA_1 | Q9X1V8 | 5.77 | 70086.13 |
| LEPA_STRE4_1 | C0M8H9 | 5.1 | 68043 |
| LEPA_RHOSK_1 | B9KNH9 | 5.8 | 67100.75 |
| LEPA_PSEA6_1 | Q15R31 | 5.4 | 66027.8 |
| LEPA_THIDA_1 | Q3SH47 | 5.26 | 66185.2 |
| LEPA_STRMU_1 | Q8DTF3 | 5.16 | 68357.5 |
| LEPA_RICPR_1 | Q9ZDQ1 | 6.19 | 67111.39 |
| LEPA_TRIEI_1 | Q112D2 | 5.27 | 67001.13 |
| LEPA_SORC5_1 | A9GWZ4 | 5.69 | 66371.35 |
| LEPA_STRT1_1 | Q5M008 | 4.92 | 68252.15 |
| LEPA_ACISJ_1 | A1WAW8 | 5.36 | 66489.51 |
| LEPA_CALBD_1 | B9MJZ5 | 5.34 | 67957.77 |
| LEPA_BACWK_1 | A9VHU6 | 5.27 | 68023.18 |
| LEPA_CORJK_1 | Q4JWS1 | 5.06 | 68444.51 |
| LEPA_BAUCH_1 | Q1LTI1 | 8.8 | 67795.67 |
| LEPA_EHRCJ_1 | Q3YSC2 | 5.89 | 66867.12 |
| LEPA_AGARV_1 | C4Z9E3 | 4.98 | 67484.6 |
| LEPA_MACCJ_1 | B9E6X5 | 4.99 | 67454.58 |
| LEPA_BIFLD_1 | B3DSA9 | 5.25 | 69258.18 |
| LEPA_METC4_1 | B7KR78 | 5.38 | 66879.16 |
| LEPA_EXISA_1 | C4L433 | 5.2 | 67511.73 |
| LEPA_ALKOO_1 | A8MFA6 | 5.18 | 67534.9 |
| LEPA_AMOA5_1 | B3ESU2 | 5.53 | 66420.88 |
| LEPA_LACS1_1 | Q1WUE6 | 4.96 | 68179.85 |
| LEPA_BORPD_1 | A9III9 | 5.52 | 65744.89 |
| LEPA_ONYPE_1 | P60792 | 8.91 | 67951.31 |
| LEPA_PARD8_1 | A6LC18 | 5.49 | 66451.65 |
| LEPA_SHEPC_1 | A4Y4K2 | 5.48 | 65932.83 |
| LEPA_RICTY_1 | Q68X95 | 6.28 | 67101.31 |
| LEPA_WOLSU_1 | Q7M8H5 | 5.36 | 66366.58 |
| LEPA_ACICJ_1 | A5FY07 | 5.44 | 66313.14 |
| LEPA_BACTN_1 | Q8AA33 | 5.42 | 66623.89 |
| LEPA_EHRCR_1 | Q2GGA6 | 5.94 | 66986.12 |
| LEPA_LISW6_1 | A0AIS8 | 5.07 | 67750.88 |
| LEPA_HISS2_1 | B0USR9 | 5.35 | 66290.06 |
| LEPA_CAMLR_1 | B9KD01 | 5.86 | 66596.22 |
| LEPA_EUBE2_1 | C4Z541 | 5.02 | 67621.76 |
| LEPA_METCA_1 | Q608M4 | 5.25 | 66210.39 |
| LEPA_KINRD_1 | A6WDJ3 | 5.3 | 68450.7 |
| LEPA_METPB_1 | B1ZC10 | 5.32 | 66947.17 |
| LEPA_KOCRD_1 | B2GHU9 | 5.13 | 68398.32 |
| LEPA_DEIRA_1 | Q9RV84 | 4.98 | 67036.7 |
| LEPA_MYCBP_1 | A1KL96 | 5.55 | 72395.8 |
| LEPA_DINSH_1 | A8LR11 | 5.59 | 66856.65 |
| LEPA_NITSB_1 | A6Q241 | 5.05 | 66200.28 |
| LEPA_PSEE4_1 | Q1I5V6 | 5.46 | 66161.97 |
| LEPA_GEOLS_1 | B3E9R0 | 5.52 | 67235.72 |
| LEPA_GEOMG_1 | Q39US7 | 5.55 | 67271.73 |
| LEPA_GEOSL_1 | P60789 | 5.55 | 67265.9 |
| LEPA_CHRSD_1 | Q1QX26 | 5.28 | 67411.17 |
| LEPA_CHRVO_1 | Q7NWC7 | 5.43 | 65929.07 |
| LEPA_BRUO2_1 | A5VVU4 | 5.31 | 66895 |
| LEPA_PECCP_1 | C6DC02 | 5.5 | 66662.5 |
| LEPA_PELPD_1 | A1ARG8 | 5.5 | 67225.59 |
| LEPA_CLONN_1 | A0Q1R8 | 5.21 | 67505.83 |
| LEPA_RUEPO_1 | Q5LUS0 | 5.51 | 66689.56 |
| LEPA_BURA4_1 | B1YVM2 | 5.48 | 65966.93 |
| LEPA_PHOPR_1 | Q6LMS0 | 5.23 | 65885.7 |
| LEPA_SALCH_1 | Q57LC8 | 5.4 | 66538.28 |
| LEPA_SALG2_1 | B5RD51 | 5.47 | 66594.35 |
| LEPA_SALNS_1 | B4T1G0 | 5.4 | 66538.28 |
| LEPA_XANP2_1 | A7IEG8 | 5.35 | 66328.63 |
| LEPA_SALSV_1 | B4TS16 | 5.4 | 66538.28 |
| LEPA_YERE8_1 | A1JKK1 | 5.56 | 66618.47 |
| LEPA_THEFY_1 | Q47RQ0 | 5.2 | 67744.66 |
| LEPA_STRCO_1 | Q9RDC9 | 5.45 | 68378.48 |
| LEPA_YERPG_1 | A9R400 | 5.57 | 66709.47 |
| LEPA_THET2_1 | Q72KV2 | 5.66 | 67711.07 |
| LEPA_STRGG_1 | B1VY28 | 5.31 | 68490.48 |
| LEPA_ZYMMO_1 | Q5NLP5 | 5.51 | 66714.73 |
| LEPA_RICFE_1 | Q4UKS2 | 5.83 | 67089.29 |
| LEPA_VEREI_1 | A1WMW4 | 5.91 | 66059.18 |
| LEPA_RICRO_1 | B0BWV5 | 5.87 | 67264.6 |
| LEPA_SOLUE_1 | Q01SV7 | 5.5 | 67243.37 |
| LEPA_CHLTE_1 | Q8KCH0 | 5.38 | 67767.99 |
| LEPA_LACRJ_1 | B2G6W5 | 5.15 | 68063.75 |
| LEPA_NEOSM_1 | Q2GD00 | 6.05 | 66916.17 |
| LEPA_NITEU_1 | Q820H8 | 5.58 | 66095.38 |
| LEPA_SHEHH_1 | B0TIV5 | 5.19 | 65798.58 |
| LEPA_STRMK_1 | B2FQC4 | 5.65 | 66516.5 |
| LEPA_SHESH_1 | A8FSD4 | 5.29 | 65843.62 |
| LEPA_SPHAL_1 | Q1GRH5 | 5.61 | 67044.08 |
| LEPA_XANAC_1 | Q8PMV3 | 5.69 | 66329.43 |
| LEPA_ACIC5_1 | C1F2I3 | 5.33 | 67102.25 |
| LEPA_BACP2_1 | A8FFD6 | 5.23 | 68636.16 |
| LEPA_EDWI9_1 | C5BAI0 | 5.28 | 66158.91 |
| LEPA_CAMC5_1 | A7GZW3 | 5.45 | 66115.29 |
| LEPA_CAMHC_1 | A7I1F0 | 5.94 | 66706.2 |
| LEPA_CUTAK_1 | Q6A9B2 | 5.24 | 66868.45 |
| LEPA_HYDCU_1 | Q31HP5 | 5.18 | 66480.4 |
| LEPA_HYPNA_1 | Q0C5X0 | 5.25 | 66961.95 |
| LEPA_KORVE_1 | Q1IV51 | 5.49 | 66982.42 |
| LEPA_BLOPB_1 | Q492C9 | 9.3 | 67900.32 |
| LEPA_MICLC_1 | C5CCD4 | 5.05 | 68158.06 |
| LEPA_CHLP8_1 | B3QPU3 | 5.45 | 67514.58 |
| LEPA_DESOH_1 | A9A0N0 | 5.75 | 66686.1 |
| LEPA_LACH4_1 | A8YVQ1 | 4.96 | 68796.55 |
| LEPA_DESVM_1 | B8DIZ5 | 5.54 | 67322.33 |
| LEPA_BORBR_1 | Q7WD30 | 5.51 | 65965.15 |
| LEPA_FUSNN_1 | Q8RFD1 | 5.23 | 67716.01 |
| LEPA_NITWN_1 | Q3SVT1 | 5.7 | 66801.23 |
| LEPA_OLICO_1 | B6JJT7 | 5.48 | 66672.13 |
| LEPA_BORPE_1 | P56865 | 5.51 | 65997.21 |
| LEPA_MYXXD_1 | Q1D6M1 | 5.73 | 66909.97 |
| LEPA_SYNWW_1 | Q0AWL9 | 5.18 | 67591.85 |
| LEPA_TERTT_1 | C5BRN3 | 5.32 | 65647.45 |
| LEPA_STRGC_1 | A8AWG3 | 4.9 | 67566.5 |
| LEPA_TOLAT_1 | C4LBZ6 | 5.14 | 66180.92 |
| LEPA_SHEPA_1 | A8H1C5 | 5.11 | 65869.61 |
| LEPA_TROW8_1 | Q83NI1 | 6.27 | 66231.06 |
| LEPA_VIBVU_1 | Q8DC78 | 5.29 | 65961.85 |
| LEPA_SYNE7_1 | Q31R08 | 5.14 | 67437.53 |
| LEPA_CLOTH_1 | A3DF29 | 5.2 | 67502.83 |
| LEPA_BACV8_1 | A6L744 | 5.53 | 66371.52 |
| LEPA_CAMC1_1 | A7ZCJ3 | 5.45 | 65836.01 |
| LEPA_CROS8_1 | A7MH13 | 5.29 | 66508.24 |
| LEPA_CHLAB_1 | Q5L659 | 6.08 | 67545.19 |
| LEPA_LACAC_1 | Q5FJP6 | 4.93 | 68622.21 |
| LEPA_MICAN_1 | B0JQT7 | 5.19 | 67222.44 |
| LEPA_MOOTA_1 | Q2RKX8 | 5.67 | 67229.51 |
| LEPA_LACDB_1 | Q049W8 | 4.94 | 68556.28 |
| LEPA_MYCAP_1 | A5IYS0 | 5.74 | 67503.58 |
| LEPA_CHLPB_1 | B3EPG7 | 5.31 | 67430.35 |
| LEPA_BORBP_1 | Q662S4 | 6.36 | 67708.12 |
| LEPA_LACP3_1 | Q038N6 | 4.98 | 68392.41 |
| LEPA_PSECP_1 | B8H8S3 | 5.53 | 68067.5 |
| LEPA_MYCGA_1 | Q7NAT2 | 7.1 | 67638.35 |
| LEPA_OCHA4_1 | A6WYK4 | 5.47 | 66987.19 |
| LEPA_ANAPZ_1 | Q2GJV7 | 5.84 | 66382.69 |
| LEPA_ANAVT_1 | Q3MG20 | 5.19 | 67388.49 |
| LEPA_HAES1_1 | Q0I4Z1 | 5.35 | 66290.06 |
| LEPA_LEPCP_1 | B1XZN0 | 5.26 | 66509.41 |
| LEPA_HELAH_1 | Q17X87 | 5.55 | 66922.53 |
| LEPA_MYCMS_1 | Q6MTR6 | 5.6 | 67715.45 |
| LEPA_ACIAC_1 | A1TLE7 | 5.31 | 66502.6 |
| LEPA_AZOVD_1 | C1DQS0 | 5.66 | 66212.32 |
| LEPA_MYCPE_1 | Q8EWZ9 | 5.93 | 67355.74 |
| LEPA_RUTMC_1 | A1AWP9 | 6.56 | 66498.67 |
| LEPA_POLSJ_1 | Q126K0 | 5.47 | 66295.33 |
| LEPA_SALEP_1 | B5QTV0 | 5.4 | 66538.28 |
| LEPA_SALHS_1 | B4TE17 | 5.4 | 66538.28 |
| LEPA_BACCN_1 | A7GT14 | 5.08 | 68143.24 |
| LEPA_RHOOB_1 | C1AUN7 | 5.52 | 69405.61 |
| LEPA_PSE14_1 | Q48EV0 | 5.62 | 65945.99 |
| LEPA_SHEFN_1 | Q07YZ4 | 5.67 | 65928.88 |
| LEPA_VARPS_1 | C5CSF2 | 5.35 | 66341.41 |
| LEPA_SHISS_1 | Q3YYU7 | 5.4 | 66560.24 |
| LEPA_COLP3_1 | Q47WP3 | 5.25 | 65743.43 |
| LEPA_CORDI_1 | P60931 | 4.99 | 68640.41 |
| LEPA_CUPMC_1 | Q1LKM8 | 5.48 | 65942.91 |
| LEPA_CUPTR_1 | B3R202 | 5.35 | 65859.85 |
| LEPA_ESCF3_1 | B7LUZ5 | 5.4 | 66570.27 |
| LEPA_CARHZ_1 | Q3AF13 | 5.43 | 67977.79 |
| LEPA_FINM2_1 | B0S1G2 | 5.07 | 67950.12 |
| LEPA_CHLFF_1 | Q254E1 | 5.99 | 67450.06 |
| LEPA_CHLL2_1 | B3EE17 | 5.44 | 67707.79 |
| LEPA_DESDA_1 | B8J444 | 5.44 | 67176 |
| LEPA_DESHD_1 | B8FUP2 | 5.19 | 66688.69 |
| LEPA_LACF3_1 | B2GBR9 | 5.03 | 68330.7 |
| LEPA_BORAP_1 | Q0SP76 | 6.56 | 67762.23 |
| LEPA_CHLPN_1 | Q9Z8I4 | 6.14 | 67286.01 |
| LEPA_NITMU_1 | Q2Y873 | 5.9 | 66483.89 |
| LEPA_ANAD2_1 | B8JAF3 | 5.54 | 67289.14 |
| LEPA_LACSS_1 | Q38W39 | 4.86 | 68204.93 |
| LEPA_PSEMY_1 | A4XSC1 | 5.58 | 66006.9 |
| LEPA_GEOUR_1 | A5G4G3 | 5.43 | 67211.64 |
| LEPA_GLOVI_1 | Q7NGX4 | 5.14 | 66663.62 |
| LEPA_GRAFK_1 | A0M6M2 | 5.15 | 66544.44 |
| LEPA_STRU0_1 | B9DSE0 | 4.99 | 67921.83 |
| LEPA_STRS2_1 | A4W0W0 | 4.93 | 68014.02 |
| LEPA_BACLD_1 | Q65H50 | 5.08 | 68343.64 |
| LEPA_BURVG_1 | A4JCQ9 | 5.54 | 65932.96 |
| LEPA_BACVZ_1 | A7Z6W5 | 5.29 | 68459.99 |
| LEPA_ACTSZ_1 | A6VLV6 | 5.24 | 66067.84 |
| LEPA_HELMI_1 | B0TAD2 | 5.12 | 66789.17 |
| LEPA_AGRFC_1 | Q8UIQ2 | 5.35 | 67123.09 |
| LEPA_IDILO_1 | Q5R104 | 5.28 | 66812.59 |
| LEPA_CYTH3_1 | Q11UD0 | 5.28 | 66807.55 |
| LEPA_CELJU_1 | B3PLG4 | 5.38 | 65934.95 |
| LEPA_METS4_1 | B0UFE0 | 5.36 | 66643.92 |
| LEPA_DELAS_1 | A9BNJ8 | 5.23 | 66442.42 |
| LEPA_CHLCV_1 | Q823H7 | 5.83 | 67568.18 |
| LEPA_ALKEH_1 | Q0A8Z4 | 5.96 | 69415.53 |
| LEPA_DESAG_1 | Q30XI4 | 5.43 | 67236.24 |
| LEPA_LACCB_1 | B3WEQ5 | 4.98 | 68392.41 |
| LEPA_MYCA5_1 | B3PLU0 | 5.67 | 67743.04 |
| LEPA_LACGA_1 | Q044A7 | 5.12 | 68885.88 |
| LEPA_ALTMD_1 | B4RVA8 | 5.17 | 66285.07 |
| LEPA_NITOC_1 | Q3J8D2 | 6.36 | 66915.14 |
| LEPA_NOVAD_1 | Q2G550 | 5.42 | 67063.04 |
| LEPA_CLOAB_1 | Q97JJ6 | 5.28 | 67611.88 |
| LEPA_PSYA2_1 | Q4FUV9 | 5.13 | 66376.47 |
| LEPA_PELCD_1 | Q3A445 | 5.45 | 67109.2 |
| LEPA_PELTS_1 | A5D3X6 | 5.91 | 67046.42 |
| LEPA_ROSDO_1 | Q16BA3 | 5.36 | 66740.57 |
| LEPA_AZOSB_1 | A1K601 | 5.36 | 66050.13 |
| LEPA_MYCPA_1 | P60791 | 5.34 | 72810.21 |
| LEPA_POLAQ_1 | A4SVW0 | 5.64 | 66222.26 |
| LEPA_POLNS_1 | B1XTL2 | 5.42 | 66094.21 |
| LEPA_SALDC_1 | B5FRC7 | 5.4 | 66538.28 |
| LEPA_RHIME_1 | Q92SU3 | 5.56 | 67152.54 |
| LEPA_PROMH_1 | B4F049 | 5.19 | 66101.89 |
| LEPA_SERP5_1 | A8GI27 | 5.59 | 66494.37 |
| LEPA_THEP3_1 | B0KA85 | 5.18 | 68008.5 |
| LEPA_SHELP_1 | A3QBS5 | 5.23 | 65775.52 |
| LEPA_TREDE_1 | P60794 | 5.18 | 67230.95 |
| LEPA_RICPU_1 | C4K153 | 5.87 | 67263.57 |
| LEPA_VESOH_1 | A5CWJ4 | 5.8 | 66490.88 |
| LEPA_VIBCB_1 | A7MZB0 | 5.14 | 65991.65 |
| LEPA_SULDN_1 | Q30Q17 | 5.47 | 66298.64 |
| LEPA_SYNAS_1 | Q2LTN3 | 5.77 | 67015.78 |
| EF-P | Accession number | pI | MW |
| EFP_ECOLI_1 | P0A6N4 | 4.9 | 20460.12 |
| EFP_SALTY_1 | P64036 | 4.9 | 20492.12 |
| EFP_BACSU_1 | P49778 | 5.04 | 20468.17 |
| EFP_MYCS2_1 | A0QWR4 | 4.98 | 20422 |
| EFP_YERPE_1 | Q8ZIY0 | 4.91 | 20711.4 |
| EFP_HAEIN_1 | P43771 | 4.87 | 20481.24 |
| EFP_SALTI_1 | P64037 | 4.9 | 20492.12 |
| EFP_SHIFL_1 | P0A6N7 | 4.9 | 20460.12 |
| EFP_BUCAI_1 | P57133 | 9.3 | 21721.09 |
| EFP_LEGPH_1 | Q5ZYS4 | 4.92 | 20886.73 |
| EFP_XYLFA_1 | P64044 | 4.94 | 20744.46 |
| EFP_PHOPR_1 | Q6LM11 | 4.87 | 20682.57 |
| EFP_SALPA_1 | Q5PL77 | 4.9 | 20492.12 |
| EFP_VIBCH_1 | Q9KNS1 | 4.93 | 20576.4 |
| EFP1_GEOSL_1 | Q74FY7 | 4.84 | 21058.95 |
| EFP_COXBU_1 | Q83AR4 | 5.74 | 21044.92 |
| EFP_PECAS_1 | Q6D025 | 5.05 | 20849.42 |
| EFP_ALIF1_1 | Q5E2B3 | 4.78 | 20650.37 |
| EFP_CROS8_1 | A7MMC3 | 4.9 | 20505.2 |
| EFP_BACFR_1 | P70889 | 5.18 | 21225.24 |
| EFP1_RHILO_1 | Q98F60 | 4.92 | 20978.87 |
| EFP_CLOBA_1 | B2V4S9 | 4.97 | 20832.75 |
| EFP_BURCC_1 | B1JYC0 | 4.83 | 20821.65 |
| EFP_BACTN_1 | Q8A1F7 | 5.17 | 21227.28 |
| EFP_BURM1_1 | A9ADD1 | 4.88 | 20874.72 |
| EFP_GEOSW_1 | C5D486 | 5.32 | 20834.81 |
| EFP_MYCSS_1 | Q1B9G8 | 4.96 | 20433.14 |
| EFP_MYCTU_1 | P9WNM3 | 5.54 | 20407.14 |
| EFP_BORHD_1 | B2RZS4 | 5.28 | 21654.06 |
| EFP_MAGMM_1 | A0L673 | 5.58 | 21102.32 |
| EFP_MANSM_1 | Q65V95 | 4.91 | 21215.18 |
| EFP_NITMU_1 | Q2Y9K0 | 5.19 | 21513.47 |
| EFP_NITOC_1 | Q3J7W7 | 4.85 | 20818.48 |
| EFP_CUPNH_1 | Q0K8N9 | 4.81 | 20983.86 |
| EFP_NOCSJ_1 | A1SJD7 | 5.15 | 20599.28 |
| EFP_RICPR_1 | Q9ZDT7 | 6.75 | 21213.53 |
| EFP_HELP2_1 | B6JPS3 | 5.43 | 20773.87 |
| EFP_SPHWW_1 | A5VAL5 | 4.86 | 21030.89 |
| EFP_STRZJ_1 | C1CCJ8 | 4.86 | 20600.43 |
| EFP_PSEAB_1 | Q02P14 | 4.82 | 20984.83 |
| EFP_STAA9_1 | A5IT57 | 4.75 | 20554.07 |
| EFP_ORITI_1 | B3CQC7 | 7.82 | 21442.87 |
| EFP_RUEST_1 | Q1GF47 | 5 | 20973.94 |
| EFP_CHLSY_1 | B9LAT1 | 4.78 | 21205.16 |
| EFP_PARL1_1 | A7HUY3 | 5.13 | 21052.8 |
| EFP_SALA4_1 | B5F2L4 | 4.9 | 20623.31 |
| EFP_PETMO_1 | A9BEN2 | 4.95 | 20999.85 |
| EFP_POLAQ_1 | A4SVW9 | 4.89 | 20848.8 |
| EFP_STRM5_1 | B4SQB1 | 4.79 | 20703.4 |
| EFP_EHRCR_1 | Q2GG58 | 6.09 | 21277.28 |
| EFP_FRATW_1 | A4J019 | 4.79 | 20902.67 |
| EFP_BURVG_1 | A4JCR8 | 4.81 | 20819.68 |
| EFP_CLOTH_1 | A3DDQ3 | 5.48 | 20653.64 |
| EFP_BORT9_1 | A1QZ08 | 5.16 | 21740.17 |
| EFP_NITEU_1 | Q82W02 | 4.94 | 21071.26 |
| EFP_PROMA_1 | Q7VEI7 | 5.2 | 20560.42 |
| EFP_CELJU_1 | B3PIK9 | 4.84 | 21065.03 |
| EFP_SINFN_1 | C3MCN8 | 5.23 | 20893.63 |
| EFP_CHLL2_1 | B3EER6 | 5.16 | 21100.83 |
| EFP_METPB_1 | B1ZHM8 | 5.39 | 20742.48 |
| EFP_MYCA5_1 | B3PMC6 | 5.12 | 20591.57 |
| EFP_SYNSC_1 | Q3ANM0 | 5 | 20653.47 |
| EFP_YERPY_1 | B1JMQ7 | 4.91 | 20711.4 |
| EFP_TOLAT_1 | C4LDX4 | 5.02 | 21096.31 |
| EFP_ELUMP_1 | B2KCP2 | 5.22 | 21388.46 |
| EFP_SHESA_1 | A0KX52 | 4.8 | 20633.41 |
| EFP_ACIAD_1 | Q6FAA9 | 4.87 | 21121.08 |
| EFP_ACTP7_1 | B3H1A1 | 4.76 | 20816.49 |
| EFP_BACSK_1 | Q5WF43 | 5.3 | 20704.43 |
| EFP2_LACAC_1 | Q5FJG2 | 5.07 | 20862.47 |
| EFP_BURP6_1 | A3NBS1 | 4.81 | 20791.63 |
| EFP_DESVV_1 | A1VDC3 | 5.31 | 20892.8 |
| EFP_CORDI_1 | Q6NH07 | 5.25 | 20602.27 |
| EFP_DICT6_1 | B5YDZ9 | 4.73 | 20939.78 |
| EFP_BORDL_1 | B5RL39 | 5.38 | 21668.17 |
| EFP_HELAH_1 | Q17YT0 | 5.31 | 20756.87 |
| EFP_CHLAD_1 | B8G711 | 4.79 | 21204.18 |
| EFP_CHLCH_1 | Q3ATP1 | 5.16 | 21048.73 |
| EFP_DEIDV_1 | C1CZB4 | 5.04 | 20502.53 |
| EFP_METNO_1 | B8IS61 | 5.41 | 20748.43 |
| EFP_DESAA_1 | B8FLV5 | 4.74 | 21209.45 |
| EFP_SULNB_1 | A6Q7Q7 | 5.3 | 20860.02 |
| EFP_RUTMC_1 | A1AVI4 | 4.68 | 20641.46 |
| EFP_PARP8_1 | B2JFL0 | 4.8 | 20841.85 |
| EFP_DESHY_1 | Q24UX6 | 5.62 | 20398.1 |
| EFP_HYPNA_1 | Q0BXH7 | 4.95 | 20869.7 |
| EFP_THESQ_1 | B1LAR0 | 4.86 | 20795.66 |
| EFP_SHEB9_1 | A9L1B3 | 4.79 | 20583.39 |
| EFP_RHOP2_1 | Q2IVV2 | 5.44 | 20655.36 |
| EFP_MYCMM_1 | B2HND2 | 5.34 | 20366.04 |
| EFP_RICAH_1 | A8GMN6 | 5.55 | 21265.5 |
| EFP_FLAPJ_1 | A6GY96 | 5.74 | 20684.54 |
| EFP_FRAP2_1 | B0TW77 | 4.85 | 20924.72 |
| EFP_ACIET_1 | B9MC94 | 4.9 | 20482.48 |
| EFP_AGARV_1 | C4Z8W1 | 4.88 | 20700.47 |
| EFP_ALCBS_1 | Q0VLQ4 | 4.8 | 20985.6 |
| EFP_AMOA5_1 | B3ES99 | 5.28 | 21435.45 |
| EFP_AYWBP_1 | Q2NJ31 | 8.84 | 21657.16 |
| EFP_CAUVN_1 | B8H1A0 | 5.07 | 20537.13 |
| EFP_RICBR_1 | Q1RHW1 | 5.24 | 21401.46 |
| EFP_HELMI_1 | B0TEH1 | 5.33 | 20736.55 |
| EFP_SACEN_1 | A4FBF7 | 4.88 | 20390.81 |
| EFP_PARPJ_1 | B2SZU7 | 4.98 | 20831.82 |
| EFP_KLEP3_1 | B5Y354 | 4.91 | 20603.37 |
| EFP_STRA1_1 | Q3JZJ4 | 4.87 | 20684.5 |
| EFP_LACF3_1 | B2GES0 | 5.14 | 20561.49 |
| EFP_STRGC_1 | A8AZB9 | 4.79 | 20654.46 |
| EFP_THENN_1 | B9K846 | 4.95 | 20755.6 |
| EFP_RHIME_1 | Q92ST6 | 5.25 | 20935.71 |
| EFP_THIDA_1 | Q3SKH1 | 4.82 | 20866.8 |
| EFP_MYCHJ_1 | Q4A9R0 | 5.83 | 20783.67 |
| EFP_MYCLE_1 | Q9CCS0 | 5.78 | 20397.14 |
| EFP_FLAJ1_1 | A5FFT9 | 5.37 | 20749.61 |
| EFP_FRASN_1 | A8LE05 | 4.88 | 20067.73 |
| EFP_ACIB5_1 | B7I499 | 4.88 | 20891.67 |
| EFP_BACC3_1 | C1ERR9 | 5.35 | 20661.5 |
| EFP_BRUC2_1 | A9M7K7 | 4.92 | 20729.64 |
| EFP_BRUME_1 | Q8YIW2 | 4.92 | 20699.57 |
| EFP_BRUSI_1 | A9WWI6 | 4.92 | 20729.64 |
| EFP_DESOH_1 | A8ZVH5 | 4.9 | 21220.29 |
| EFP_CALS8_1 | A4XJ18 | 5.19 | 20956.98 |
| EFP_CORA7_1 | C3PGM2 | 5.15 | 20894.53 |
| EFP_CORGB_1 | A4QEJ4 | 5.14 | 20640.23 |
| EFP_LISW6_1 | A0AIF6 | 5.14 | 20451.22 |
| EFP_BREBN_1 | C0ZBY1 | 5.1 | 20912.66 |
| EFP_NOCFA_1 | Q5YTL1 | 5.07 | 20367.01 |
| EFP_MARMS_1 | A6VTQ2 | 5.05 | 21012.74 |
| EFP_STRPZ_1 | B5XI32 | 4.93 | 20466.28 |
| EFP_HELHP_1 | Q7VJY4 | 5.42 | 20672.68 |
| EFP_METCA_1 | Q609B5 | 4.95 | 20763.66 |
| EFP_NOVAD_1 | Q2G6X5 | 4.83 | 20830.71 |
| EFP_PARD8_1 | A6LF43 | 5.08 | 21244.14 |
| EFP_SACD2_1 | Q21LT5 | 4.95 | 20807.45 |
| EFP_WOLSU_1 | Q7M904 | 5.4 | 21062.02 |
| EFP_HYDS0_1 | B4U7D5 | 5.15 | 22007.01 |
| EFP_SALDC_1 | B5FRK6 | 4.9 | 20623.31 |
| EFP_MYCBO_1 | P64035 | 5.54 | 20407.14 |
| EFP_POLNA_1 | A1VMR1 | 4.88 | 20673.62 |
| EFP_RHOCS_1 | B6INP2 | 5.05 | 20674.53 |
| EFP_SHEPW_1 | B8CPB0 | 4.61 | 20700.45 |
| EFP_RHORT_1 | Q2RVG7 | 4.85 | 20798.73 |
| EFP_SHIDS_1 | Q328H8 | 4.9 | 20591.31 |
| EFP_ACAM1_1 | B0C899 | 4.99 | 20546.21 |
| EFP_ACHLI_1 | A9NGL1 | 5.08 | 20998.06 |
| EFP_ALKEH_1 | Q0AAU9 | 4.79 | 20739.51 |
| EFP_ALKMQ_1 | A6TR25 | 5.18 | 20996.85 |
| EFP_AZOVD_1 | C1DRK5 | 4.78 | 21588.41 |
| EFP1_CHLCV_1 | Q822X6 | 4.78 | 20759.03 |
| EFP_GEMAT_1 | C1A9H6 | 5.57 | 21134.88 |
| EFP_CLOTE_1 | Q894F6 | 5.18 | 20932.83 |
| EFP_GLUDA_1 | A9H6E1 | 4.87 | 20946.9 |
| EFP_CAMJD_1 | A7H4J1 | 5.18 | 21098.2 |
| EFP_CORK4_1 | C4LIU5 | 5.11 | 20725.58 |
| EFP_BORPA_1 | Q7W7W9 | 4.85 | 20867.78 |
| EFP_HAES1_1 | Q0I4U4 | 4.9 | 20626.5 |
| EFP_HAHCH_1 | Q2SBA6 | 4.83 | 20779.3 |
| EFP_MESFL_1 | Q6F157 | 4.87 | 20513.41 |
| EFP_METC4_1 | B7KT38 | 5.55 | 20743.44 |
| EFP_CYAP8_1 | B7JVR7 | 4.81 | 20516.19 |
| EFP_RICTY_1 | Q68XD1 | 7.73 | 21155.5 |
| EFP_CHLP8_1 | B3QQR8 | 5.07 | 21121.93 |
| EFP_METI4_1 | B3E058 | 5.8 | 20674.91 |
| EFP_SULDN_1 | Q30S45 | 5.15 | 21095.12 |
| EFP_PSEE4_1 | Q1ID35 | 4.77 | 21334.12 |
| EFP_PSEP1_1 | A5W770 | 4.73 | 21303.1 |
| EFP_SALG2_1 | B5R995 | 4.9 | 20623.31 |
| EFP_SALNS_1 | B4T2P5 | 4.9 | 20623.31 |
| EFP_KOSOT_1 | C5CI92 | 5.06 | 20946.98 |
| EFP_PSESM_1 | Q885R6 | 4.68 | 21137.96 |
| EFP_CLAM3_1 | A5CRY6 | 4.93 | 20468.98 |
| EFP_SALSV_1 | B4TSD0 | 4.9 | 20623.31 |
| EFP_LACRJ_1 | B2G967 | 5.1 | 20518.45 |
| EFP_SERP5_1 | A8G8T0 | 5.05 | 20765.34 |
| EFP_RALSO_1 | Q8Y0H5 | 4.98 | 21164.13 |
| EFP_PROA2_1 | B4S4A1 | 4.91 | 21232.03 |
| EFP_SHEDO_1 | Q12MQ1 | 4.94 | 20614.41 |
| EFP_THEYD_1 | B5YJ32 | 5.29 | 21609.9 |
| EFP_SHEHH_1 | B0TUS6 | 4.75 | 20703.34 |
| EFP_RHOBA_1 | Q7UXN7 | 4.78 | 21704.54 |
| EFP_ERWT9_1 | B2VL81 | 5.28 | 20679.43 |
| EFP_EUBE2_1 | C4Z157 | 5.03 | 20485.14 |
| EFP_BACAN_1 | Q6KMS8 | 5.35 | 20691.59 |
| EFP_BACHK_1 | Q6HDW8 | 5.35 | 20691.59 |
| EFP_BACP2_1 | A8FF29 | 5.16 | 20412.13 |
| EFP_BACV8_1 | A6KXS5 | 5.14 | 21125.25 |
| EFP_BURMS_1 | A1V6B0 | 4.81 | 20791.63 |
| EFP_FUSNN_1 | Q8R5X5 | 4.81 | 21206.05 |
| EFP_GEOKA_1 | Q5KX91 | 5.34 | 20856.84 |
| EFP_GRABC_1 | Q0BT69 | 4.87 | 20874.71 |
| EFP_GRAFK_1 | A0M298 | 5.56 | 21261.24 |
| EFP_NATTJ_1 | B2A549 | 4.97 | 21144.87 |
| EFP_CORU7_1 | B1VDM5 | 5.11 | 20526.26 |
| EFP_HAEPS_1 | B8F3G9 | 4.82 | 20758.46 |
| EFP_CHLPD_1 | A1BD27 | 5.3 | 20897.73 |
| EFP_DESDA_1 | B8J031 | 5.21 | 20974.06 |
| EFP_SYNE7_1 | Q54760 | 5.09 | 20368.13 |
| EFP_SALAR_1 | A9MFR5 | 4.9 | 20623.31 |
| EFP_XANC8_1 | Q4UVL7 | 4.8 | 20833.63 |
| EFP_STAEQ_1 | Q5HP20 | 4.72 | 20594.09 |
| EFP_PELTS_1 | A5D339 | 5.95 | 20681.65 |
| EFP_THEAB_1 | B7IH59 | 5.05 | 20863.66 |
| EFP_PSYWF_1 | A5WCQ0 | 4.83 | 21113.66 |
| EFP_PHYMT_1 | B3R0E7 | 9.3 | 21717.35 |
| EFP_LAWIP_1 | Q1MSA2 | 5.34 | 21197.28 |
| EFP_THEP3_1 | B0K9C8 | 4.8 | 20877.83 |
| EFP_STRMU_1 | Q8DSE7 | 4.89 | 20780.62 |
| EFP_MYCGI_1 | A4TBX4 | 5.2 | 20435.25 |
| EFP_SHESH_1 | A8FVS5 | 4.8 | 20606.41 |
| EFP_UREP2_1 | B1AIT6 | 5.21 | 21221.14 |
| EFP_FRACC_1 | Q2J829 | 4.88 | 20071.67 |
| EFP_MYCVP_1 | A1T8F9 | 4.96 | 20373.03 |
| EFP_MACCJ_1 | B9E6R3 | 4.89 | 20660.24 |
| EFP_NEIMB_1 | Q9JZQ8 | 4.77 | 20879.75 |
| EFP_CARHZ_1 | Q3AAZ2 | 5.13 | 20927.84 |
| EFP_NOSS1_1 | Q44247 | 5.2 | 20496.41 |
| EFP_STRT1_1 | Q5LY60 | 4.93 | 20679.51 |
| EFP_DELAS_1 | A9BVZ6 | 4.99 | 20548.56 |
| EFP_WOLPM_1 | Q73FP5 | 5.98 | 21353.43 |
| EFP_CHLTE_1 | Q8KG11 | 5.06 | 21090.88 |
| EFP_MOOTA_1 | Q2RI92 | 5.12 | 20954.69 |
| EFP_JANMA_1 | A6SX19 | 5.09 | 21880.91 |
| EFP_SALEP_1 | B5R009 | 4.9 | 20623.31 |
| EFP_EHRRG_1 | Q5FHJ7 | 5.67 | 21391.4 |
| EFP_SHEPA_1 | A8H482 | 4.75 | 20703.34 |
| EFP_TROWT_1 | Q83MW6 | 8.67 | 20015.72 |
| EFP_UREU1_1 | B5ZBA7 | 5.37 | 21149.08 |
| EFP_MYCPU_1 | Q98Q55 | 5.57 | 21028.03 |
| EFP_VARPS_1 | C5CUK1 | 5 | 20721.71 |
| EFP_MYCPE_1 | Q8EWP5 | 5.64 | 21227.47 |
| EFP_ACIF2_1 | B7J6R6 | 4.98 | 20766.67 |
| EFP_AERS4_1 | A4SJY6 | 4.87 | 21064.93 |
| EFP_AGRRK_1 | B9JDI1 | 5.37 | 20892.83 |
| EFP_ANAVT_1 | Q3MAQ3 | 5.2 | 20512.41 |
| EFP2_CHLMU_1 | Q9PLH1 | 4.93 | 21550.52 |
| EFP_CLONN_1 | A0Q087 | 4.98 | 20783.78 |
| EFP_DESRM_1 | A4J3D4 | 5.1 | 20462.25 |
| EFP_BIFLD_1 | B3DQ29 | 5.05 | 20654.33 |
| EFP1_CHLTR_1 | O84124 | 5.03 | 20516.74 |
| EFP_GEOTN_1 | A4IQT4 | 5.22 | 20867.91 |
| EFP_LEPBP_1 | B0SU50 | 4.93 | 21295.46 |
| EFP_BLOPB_1 | Q493W6 | 9.71 | 21447.26 |
| EFP_DICTD_1 | B8E240 | 4.81 | 20967.88 |
| EFP_BORRA_1 | B5RR32 | 5.24 | 21712.18 |
| EFP_CAUSK_1 | B0SUL4 | 5.24 | 20761.55 |
| EFP_VIBVU_1 | Q8DCX6 | 4.85 | 20670.63 |
| EFP_RUEPO_1 | Q5LU15 | 5.01 | 20953.89 |
| EFP_PSEF5_1 | Q4K8S7 | 4.65 | 21164.99 |
| EFP_HISS2_1 | B0UWM5 | 4.9 | 20626.5 |
| EFP_PARXL_1 | Q13VN6 | 4.97 | 20847.81 |
| EFP_PECCP_1 | C6DFP1 | 5.05 | 20754.25 |
| EFP_JANSC_1 | Q28M91 | 4.92 | 20895.71 |
| EFP_STAS1_1 | Q49XX3 | 4.77 | 20580.09 |
| EFP_LACLA_1 | Q9CHN6 | 4.87 | 20653.55 |
| EFP_SYNY3_1 | Q55119 | 4.77 | 20406.2 |
| EFP_RALPJ_1 | B2U987 | 4.82 | 21368.34 |
| EFP_RHIRD_1 | P0A3B6 | 5.13 | 21105.94 |
| EFP_RHOFT_1 | Q21W89 | 4.93 | 20945.9 |
| EFP_ERYLH_1 | Q2NAZ6 | 4.61 | 20966.63 |
| EFP_ACIC1_1 | A0LUG7 | 5.09 | 20425.07 |
| EFP_ALKOO_1 | A8MFK4 | 4.99 | 21014.52 |
| EFP_ANAMF_1 | B9KIA2 | 5.32 | 20905.77 |
| EFP_ANAPZ_1 | Q2GKG2 | 5.68 | 21027.94 |
| EFP1_CHLPN_1 | Q9Z900 | 4.68 | 20672.91 |
| EFP1_LACJO_1 | Q74HT9 | 5.28 | 21065.22 |
| EFP_CLOCE_1 | B8I3C7 | 5.16 | 20985.91 |
| EFP_BAUCH_1 | Q1LSP7 | 9.18 | 21483 |
| EFP_LEPIC_1 | P0C099 | 4.9 | 21159.31 |
| EFP_DICNV_1 | A5EX42 | 4.81 | 21044.75 |
| EFP_CHESB_1 | Q11DG9 | 4.88 | 20727.52 |
| EFP_PSE14_1 | Q48FP6 | 4.68 | 21137.96 |
| EFP_XANAC_1 | Q8PJZ7 | 4.78 | 20879.66 |
| EFP_SALHS_1 | B4TF84 | 4.9 | 20623.31 |
| EFP_PERMH_1 | C0QQC2 | 5.05 | 21274.29 |
| EFP_MYCA9_1 | B1MCE5 | 5.2 | 20543.08 |
| EFP_PHOLL_1 | Q7MZX9 | 4.85 | 20700.56 |
| EFP_YERE8_1 | A1JIP6 | 4.91 | 20737.42 |
| EFP_STREM_1 | B4U161 | 4.86 | 20608.43 |
| EFP_MYCGE_1 | P47272 | 5.15 | 21914.2 |
| EFP_UNCTG_1 | B1GZJ0 | 6.98 | 21033.22 |
| EFP_FERNB_1 | A7HJ78 | 4.79 | 20936.69 |
| EFP_ACIC5_1 | C1F3T7 | 5.02 | 20738.56 |
| EFP_AQUAE_1 | O67376 | 5.39 | 21806.94 |
| EFP_AZOPC_1 | B6YQT1 | 6.44 | 21204.5 |
| EFP_BACHD_1 | Q9K951 | 5.16 | 20566.18 |
| EFP_BARBK_1 | A1UR93 | 5.1 | 21175.12 |
| EFP_CLOP1_1 | Q0TPC2 | 4.92 | 20960.98 |
| EFP_CAMFF_1 | A0RQC0 | 5.33 | 21175.33 |
| EFP_BORAP_1 | Q0SNU8 | 5.41 | 21562.11 |
| EFP_BORBR_1 | Q7WLA9 | 4.85 | 20867.78 |
| EFP_LYSSC_1 | B1HS10 | 4.96 | 20667.33 |
| EFP_BRASB_1 | A5EIT5 | 5.6 | 20844.69 |
| EFP_RICFE_1 | Q4UKM7 | 5.14 | 21503.66 |
| EFP_SHISS_1 | Q3YUJ2 | 4.9 | 20591.31 |
| EFP_RICPU_1 | C4K0V3 | 5.11 | 21426.53 |
| EFP_SODGM_1 | Q2NW91 | 4.93 | 20590.43 |
| EFP_VIBPA_1 | Q87KX9 | 4.84 | 20584.5 |
| EFP_RUBXD_1 | Q1AW08 | 5.46 | 20614.18 |
| EFP_CHLPM_1 | A4SGK2 | 5.14 | 20774.41 |
| EFP_DESAP_1 | B1I3C0 | 5.28 | 20280.13 |
| EFP_SHEFN_1 | Q082R1 | 4.68 | 20729.48 |
| EFP_EHRCJ_1 | Q3YSG0 | 6.43 | 21323.35 |
| EFP_CLOAB_1 | Q97HB8 | 4.98 | 21076 |
| EFP_GLOVI_1 | Q7NL41 | 5.09 | 20879.74 |
| EFP_LEPCP_1 | B1Y0U3 | 5.18 | 20604.61 |
| EFP_NEIG2_1 | B4RL51 | 4.75 | 20865.72 |
| EFP_CAMLR_1 | B9KD70 | 5.2 | 21155.25 |
| EFP_SHELP_1 | A3QE83 | 4.73 | 20701.37 |
| EFP_RHOE4_1 | C0ZZC0 | 5.06 | 20523.14 |
| EFP_SHEPC_1 | A4Y6L5 | 4.79 | 20571.34 |
| EFP_RHOSK_1 | B9KKM5 | 5.14 | 21031.77 |
| EFP_SHIBS_1 | Q31T82 | 4.9 | 20591.31 |
| EFP_ACTSZ_1 | A6VR54 | 4.91 | 20683.51 |
| EFP_ANOFW_1 | B7GHE5 | 5.19 | 20759.72 |
| EFP_ARTS2_1 | A0JX80 | 4.84 | 20735.37 |
| EFP_BORBP_1 | Q662F0 | 5.41 | 21451.91 |
| EFP_MYCUA_1 | A0PPH6 | 5.34 | 20400.06 |
| EFP_HAEDU_1 | Q7VLM1 | 4.9 | 20684.46 |
| EFP_HAMD5_1 | C4K7E2 | 5 | 20611.53 |
| EFP_VESOH_1 | A5CXM6 | 5.02 | 20722.73 |
| EFP_DEIGD_1 | Q1J163 | 5.01 | 20497.37 |
| EFP_STRU0_1 | B9DVJ6 | 4.86 | 20569.35 |
| EFP_PASMU_1 | P57811 | 4.87 | 20598.44 |
| EFP_PSEMY_1 | A4XU74 | 4.8 | 21414.36 |
| EFP_CHRSD_1 | Q1QUI0 | 4.74 | 20845.55 |
| EFP_XANOM_1 | Q2P2C1 | 4.79 | 20863.66 |
| EFP_SALRD_1 | Q2S413 | 4.43 | 21710.92 |
| EFP_PSYCK_1 | Q1Q9C6 | 5 | 20903.52 |
| EFP_SALTO_1 | A4X611 | 4.72 | 19977.57 |
| EFP_STRGG_1 | B1W455 | 5.07 | 20529.26 |
| EFP_RHILW_1 | B5ZU68 | 5.24 | 21013.86 |
| EFP_TRIEI_1 | Q116D6 | 4.91 | 20673.37 |
| EFP_ESCF3_1 | B7LLS9 | 4.9 | 20591.31 |
| EFP_ACISJ_1 | A1W9U1 | 4.9 | 20482.48 |
| EFP_AGRVS_1 | B9K2E5 | 5.13 | 21145.05 |
| EFP_BURCM_1 | Q0BGZ7 | 4.81 | 20840.72 |
| EFP_LEPBJ_1 | Q04NC1 | 4.98 | 21144.3 |
| EFP_COREF_1 | Q8FT34 | 5.11 | 20613.27 |
| EFP_LISMF_1 | Q71ZW7 | 5.14 | 20465.25 |
| EFP_BORBZ_1 | B7J1E4 | 5.41 | 21412.86 |
| EFP_BRUA1_1 | B2S7E6 | 4.92 | 20699.57 |
| EFP_NOSP7_1 | B2J711 | 5.37 | 20560.51 |
| EFP_SINMW_1 | A6UF66 | 5.25 | 20947.76 |
| EFP_RICRO_1 | B0BWQ9 | 5.12 | 21472.61 |
| EFP_VIBCB_1 | A7MZ47 | 4.82 | 20600.49 |
| EFP_DESAD_1 | C6BUE7 | 5.23 | 20678.54 |
| EFP_PSECP_1 | B8H8V3 | 4.86 | 20708.35 |
| EFP_SULSY_1 | B2V9Q7 | 5.33 | 21654.54 |
| EFP_METS4_1 | B0UJ99 | 5.57 | 20740.53 |
| EFP_STACT_1 | B9DNQ4 | 4.79 | 20563.18 |
| EFP_STRAW_1 | Q827R5 | 5.1 | 20621.44 |
| EFP_RHIE6_1 | B3PRW7 | 5.24 | 21038.85 |
| EFP_TREPS_1 | B2S3B8 | 5.85 | 21042.9 |
| EFP_RHOOB_1 | C1B4I4 | 4.92 | 20440.02 |
| EFP_FINM2_1 | B0S053 | 4.96 | 20919.63 |
| EFP2_PORGI_1 | Q7MV32 | 5.19 | 21237.26 |
| EFP_BLOFL_1 | Q7VQQ0 | 9.64 | 21419.25 |
| EFP_MAGSA_1 | Q2W7T6 | 4.88 | 20948.95 |
| EFP_HALHL_1 | A1WYI1 | 4.77 | 21042.66 |
| EFP_DECAR_1 | Q47EF1 | 4.91 | 20534.57 |
| EFP_METFK_1 | Q1H0G4 | 4.81 | 20787.8 |
| EFP_DEIRA_1 | Q9RY32 | 5.1 | 20475.27 |
| EFP_VIBTL_1 | B7VHR3 | 4.78 | 20585.39 |
| EFP_WOLTR_1 | Q5GTF2 | 5.65 | 21812.97 |
| EFP_KOCRD_1 | B2GI94 | 4.83 | 20516.01 |
| EFP_XANP2_1 | A7IID0 | 5.25 | 20965.82 |
| EFP_CITK8_1 | A8AMQ1 | 4.9 | 20578.32 |
| EFP_LACP7_1 | A9KMD6 | 5.12 | 20759.53 |
| EFP_PHYAS_1 | B1VAJ8 | 9.06 | 21703.06 |
| EFP_POLNS_1 | B1XTM0 | 4.87 | 20803.65 |
| EFP_THISH_1 | B8GQC3 | 4.93 | 20600.38 |
| EFP_SHEON_1 | Q8EEP9 | 4.79 | 20619.38 |
| EFP_ENT38_1 | A4W5P1 | 5.04 | 20659.4 |
| EFP_BACLD_1 | Q65HH4 | 5.3 | 20591.38 |
| EFP_BURL3_1 | Q39I66 | 4.81 | 20775.63 |
| EFP_COLP3_1 | Q487P4 | 4.99 | 20763.68 |
| EFP_COPPD_1 | B5Y8M4 | 4.83 | 20631.49 |
| EFP_NITWN_1 | Q3SS37 | 5.55 | 20731.52 |
| EFP_OCEIH_1 | Q8EQ28 | 5.15 | 20583.23 |
| EFP_OLICO_1 | B6JHH8 | 5.51 | 20698.39 |
| EFP_WIGBR_1 | Q8D2R7 | 9.64 | 22050.08 |
| EFP_METRJ_1 | B1M2B1 | 5.41 | 20717.5 |
| EFP_CHLT3_1 | B3QW61 | 5.2 | 21402.14 |
| EFP_SYNAS_1 | Q2LRN9 | 4.96 | 21472.48 |
| EFP_PSELT_1 | A8F7D2 | 5.06 | 20742.71 |
| EFP_MYCA1_1 | A0QI55 | 5.2 | 20472.14 |
| EFP_STAHJ_1 | Q4L6M9 | 4.8 | 20598.13 |
| EFP_PEPD6_1 | Q18BA9 | 4.98 | 20882.52 |
| EFP_RICAE_1 | C3PMT5 | 5.23 | 21457.6 |
| EFP_MYCPA_1 | Q741J3 | 5.2 | 20472.14 |
| EFP_ACICJ_1 | A5FZ78 | 4.93 | 20893.71 |
| EFP_AGRFC_1 | P0A3B5 | 5.13 | 21105.94 |
| EFP_AZOSB_1 | A1K1J9 | 4.85 | 20730.68 |
| EFP_BRUO2_1 | A5VS65 | 4.92 | 20743.63 |
| EFP_BACVZ_1 | A7Z6L3 | 5.06 | 20538.22 |
| EFP_BDEBA_1 | Q6MKB4 | 5.21 | 21154.02 |
| EFP_BIFA0_1 | B8DTW0 | 5.05 | 20517.26 |
| EFP_GLUOX_1 | Q5FUC7 | 4.87 | 21054.87 |
| EFP_BRAHW_1 | C0QW93 | 4.88 | 21267.09 |
| EFP_CUPTR_1 | B3R1Z3 | 4.81 | 20983.86 |
| EFP_RICM5_1 | A8F0Z8 | 5.22 | 21442.63 |
| EFP_DESAG_1 | Q30ZZ6 | 5.09 | 20890.85 |
| EFP_SYMTH_1 | Q67N94 | 4.84 | 20644.46 |
| EFP_TERTT_1 | C5BNT8 | 4.86 | 20776.26 |
| EFP_LACPL_1 | Q88WN1 | 5.03 | 20366.15 |
| EFP_THEMA_1 | Q9X284 | 4.87 | 20874.72 |
| EFP_MYCGA_1 | Q7NAX4 | 5.35 | 21253.4 |
| EFP_BACCN_1 | A7GSL5 | 5.23 | 20762.71 |
| EFP_BEUC1_1 | C5C680 | 4.94 | 20459.13 |
| EFP_CALBD_1 | B9MLY0 | 5.19 | 20955.01 |
| EFP_BORA1_1 | Q2KXA0 | 4.9 | 20898.85 |
| EFP_BORPD_1 | A9HX42 | 4.85 | 20850.75 |
| EFP_MARMM_1 | Q0ALG8 | 5.07 | 20852.6 |
| EFP_SALAI_1 | A8LY10 | 4.7 | 19980.58 |
| EFP_PELUB_1 | Q4FN36 | 5.62 | 21230.23 |
| EFP_PHEZH_1 | B4RHN9 | 5.14 | 20735.37 |
| EFP_LARHH_1 | C1DD68 | 4.8 | 20962.93 |
| EFP_THEP1_1 | A5ILJ5 | 4.86 | 20795.66 |
| EFP_LEIXX_1 | Q6AF92 | 4.86 | 20466.97 |
| EFP_RHOJR_1 | Q0S0M7 | 4.94 | 20466.1 |
| EFP_EXIS2_1 | B1YLP7 | 5.3 | 21042.06 |
| EFP_VEREI_1 | A1WMV6 | 5.09 | 20513.56 |
| EFP_ACIAC_1 | A1TMP7 | 5 | 20559.59 |
| EFP_BARQU_1 | Q6FYN9 | 5.18 | 21213.19 |
| EFP_BART1_1 | A9IYN9 | 5.1 | 21413.47 |
| EFP_LISIN_1 | P64033 | 5.14 | 20465.25 |
| EFP_BRADU_1 | Q89M07 | 5.41 | 20732.36 |
| EFP_NITHX_1 | Q1QL03 | 5.56 | 20859.71 |
| EFP_PROMH_1 | B4EXC9 | 4.85 | 20719.59 |
| EFP_ROSS1_1 | A5UYR6 | 5.1 | 21487.52 |
| EFP_PSEHT_1 | Q3IFP5 | 4.79 | 20566.34 |
| EFP_MICAN_1 | B0JHV3 | 4.95 | 20566.38 |
| EFP_PELPB_1 | B4SG07 | 5.32 | 20960.73 |
| EFP_MYCAP_1 | A5IY97 | 5.36 | 20967.95 |
| EFP_PSYA2_1 | Q4FR30 | 5 | 20902.5 |
| EFP_SHEAM_1 | A1S699 | 4.79 | 21185.1 |
| EFP_POLSJ_1 | Q128Q4 | 4.97 | 20700.67 |
| EFP_SHEWM_1 | B1KEH1 | 4.71 | 20569.31 |
| EFP_MYCMS_1 | Q6MTF7 | 4.96 | 20474.48 |
| EFP1_PARUW_1 | Q6MD56 | 4.82 | 20671.84 |
| EFP_BACWK_1 | A9VGY0 | 5.22 | 20785.63 |
| EFP_BARHE_1 | Q6G1Z7 | 5.21 | 21412.48 |
| EFP_CLOK5_1 | A5N7H7 | 5.19 | 20780.66 |
| EFP_CORJK_1 | Q4JVG5 | 5.26 | 20526.27 |
| EFP_RICCK_1 | A8EXY7 | 5.38 | 21430.65 |
| EFP_STRSV_1 | A3CL43 | 4.79 | 20652.48 |
| EFP_SPHAL_1 | Q1GTJ4 | 4.91 | 21085.91 |
| EFP_ROSCS_1 | A7NJE1 | 5.2 | 21463.5 |
| EFP_ROSDO_1 | Q169H6 | 5.05 | 21019.99 |
| EFP_ONYPE_1 | Q6YQU7 | 8.57 | 21591.02 |
| EFP_HERA2_1 | A9B1H0 | 4.85 | 20802.58 |
| EFP_DESMR_1 | C4XT92 | 5.19 | 20601.65 |
| EFP_IDILO_1 | Q5QVT8 | 4.8 | 20817.6 |
| EFP_SYNWW_1 | Q0AZH4 | 5.18 | 20443.44 |
| EFP_PSEU5_1 | A4VMG3 | 4.86 | 21120.99 |
| EFP_STRCO_1 | Q9KXQ9 | 5.01 | 20649.45 |
| EFP_THEPX_1 | B0K0T5 | 4.8 | 20877.83 |
| EFP_THET8_1 | Q76G20 | 4.85 | 20225.06 |
| EFP_EXISA_1 | C4L3G0 | 5.32 | 20918.82 |
| EFP_MYCMO_1 | Q6KH93 | 5.76 | 21103.15 |
| EFP_MYCS5_1 | Q4A5X8 | 5.19 | 21005.87 |
| EFP_AERHH_1 | A0KN67 | 4.88 | 21125.98 |
| EFP_AROAE_1 | Q5P4G4 | 4.83 | 20677.57 |
| EFP_DESPS_1 | Q6APZ9 | 4.82 | 21668.61 |
| EFP_BURTA_1 | Q2SXS7 | 4.8 | 20837.65 |
| EFP_CALS4_1 | Q8RAE2 | 4.8 | 20967.96 |
| EFP_BORPE_1 | Q7VX16 | 4.85 | 20867.78 |
| EFP_NITEC_1 | Q0AGI0 | 4.96 | 21142.33 |
| EFP_HALOH_1 | B8D2E5 | 4.96 | 20940.53 |
| EFP_CUPMC_1 | Q1LKN7 | 4.83 | 21071.03 |
| EFP_RICCN_1 | Q92IU8 | 5.23 | 21447.56 |
| EFP_PAEAT_1 | A1R701 | 4.78 | 20736.36 |
| EFP_DESAH_1 | C0QBX7 | 4.78 | 21072.79 |
| EFP_CHRVO_1 | Q7NY96 | 4.8 | 20906.72 |
| EFP_THEM4_1 | A6LLA5 | 4.88 | 20807.55 |
| EFP_ZYMMO_1 | Q5NQQ2 | 4.87 | 20924.88 |
| EFP_TREDE_1 | Q73P31 | 4.83 | 21100.99 |
| EFP_ENTFA_1 | Q838Z5 | 5 | 20735.85 |
| EFP_MYCPN_1 | P75085 | 5.14 | 21810.01 |
| RF1 | Accession number | pI | MW |
| RF1_ECOLI_1 | P0A7I0 | 5.13 | 40517.3 |
| RF1_THET8_1 | P96077 | 5.27 | 40092.5 |
| RF1_VIBCH_1 | Q9KQ25 | 5.08 | 40221.06 |
| RF1_BORBU_1 | O51214 | 6.66 | 41532.4 |
| RF1_MYCLE_1 | P45833 | 5.06 | 39491.95 |
| RF1_MYCTU_1 | P9WHG3 | 5.06 | 38905.29 |
| RF1_BACAN_1 | Q81JX1 | 5.1 | 40356.4 |
| RF1_BRUME_1 | Q8YJ95 | 4.99 | 39908.92 |
| RF1_CAUVC_1 | Q9A9V5 | 5.11 | 39460.35 |
| RF1_CUPMC_1 | Q1LIF3 | 5.12 | 40130.85 |
| RF1_HAEIN_1 | P43917 | 4.82 | 40916.81 |
| RF1_BACCR_1 | Q812I4 | 5.09 | 40314.36 |
| RF1_RHOP2_1 | Q2J423 | 5.21 | 39825.92 |
| RF1_ACIAD_1 | Q6F9S2 | 5.02 | 40724.43 |
| RF1_PSEAE_1 | P42806 | 5.09 | 40040.73 |
| RF1_BACHK_1 | Q6HAV4 | 5.1 | 40356.4 |
| RF1_VIBVY_1 | Q7MMY6 | 5.05 | 40138.14 |
| RF1_RHIEC_1 | Q2K3T1 | 5.15 | 39991.11 |
| RF1_BORA1_1 | Q2KZZ3 | 5.14 | 40062.07 |
| RF1_DESHY_1 | Q24ML9 | 4.94 | 40083.12 |
| RF1_BORHD_1 | B2RZQ6 | 6.14 | 41547.13 |
| RF1_CHLCV_1 | Q822M2 | 5.31 | 40569.74 |
| RF1_COXBR_1 | A9N9K2 | 5.36 | 40762.98 |
| RF1_MAGMM_1 | A0LDT7 | 5.72 | 39805.9 |
| RF1_EHRRW_1 | Q5HB80 | 5.77 | 40781.2 |
| RF1_BURCJ_1 | B4E6U6 | 5.51 | 40499.49 |
| RF1_HYDCU_1 | Q31IN8 | 5.07 | 41001.83 |
| RF1_CITK8_1 | A8AG02 | 5.13 | 40344.03 |
| RF1_MESFL_1 | Q6F0I3 | 4.93 | 40687.86 |
| RF1_CLAMS_1 | B0RAQ5 | 4.81 | 40025.66 |
| RF1_METC4_1 | B7L246 | 5.23 | 39270.13 |
| RF1_FLAPJ_1 | A6H0W3 | 4.95 | 40312.59 |
| RF1_KLEP7_1 | A6TAN9 | 5.09 | 40362.08 |
| RF1_CLOBB_1 | B2TJY5 | 5.03 | 41032.39 |
| RF1_BACSU_1 | P45872 | 5.01 | 40233.16 |
| RF1_ACIB3_1 | B7H0L9 | 5.01 | 40613.39 |
| RF1_CALS8_1 | A4XJN1 | 5.23 | 41107.47 |
| RF1_DESAD_1 | C6BYQ4 | 5.11 | 40235.41 |
| RF1_CAMC5_1 | A7GW12 | 5.25 | 39618.7 |
| RF1_DESAP_1 | B1I6L2 | 5.51 | 40455.61 |
| RF1_ALISL_1 | B6EHH5 | 5.06 | 40375.25 |
| RF1_CHLFF_1 | Q255B5 | 5.48 | 40797.87 |
| RF1_BRUC2_1 | A9M8K5 | 4.99 | 39894.89 |
| RF1_ARCB4_1 | A8EQZ4 | 5.11 | 40193.51 |
| RF1_BUCAI_1 | P57268 | 7.65 | 41261.91 |
| RF1_AROAE_1 | Q5P7G7 | 5.37 | 39687.51 |
| RF1_AZOVD_1 | C1DEV0 | 5.22 | 40264.12 |
| RF1_CROS8_1 | A7MKC1 | 5.2 | 40226.09 |
| RF1_MARHV_1 | A1U367 | 4.96 | 40710.44 |
| RF1_CYAP8_1 | B7K205 | 4.88 | 41562.8 |
| RF1_BACFR_1 | Q64XW5 | 4.97 | 41655.93 |
| RF1_FRAP2_1 | B0TX37 | 4.97 | 40472.35 |
| RF1_BURVG_1 | A4JB50 | 5.68 | 40553.59 |
| RF1_CALBD_1 | B9MR68 | 5.23 | 41076.54 |
| RF1_BACTN_1 | Q8A011 | 4.99 | 41661.94 |
| RF1_BARBK_1 | A1URS5 | 5.39 | 40400.73 |
| RF1_CAMFF_1 | A0RRK4 | 5.22 | 39880.94 |
| RF1_CAMHC_1 | A7I3Q0 | 5.41 | 39796.01 |
| RF1_CAMJ8_1 | A8FNS3 | 5.48 | 39909.28 |
| RF1_CLOP1_1 | Q0TNB0 | 5.02 | 40946.23 |
| RF1_ACIET_1 | B9ME05 | 5.31 | 40787.82 |
| RF1_HELPG_1 | B5Z680 | 5.43 | 39589.95 |
| RF1_AMOA5_1 | B3ES89 | 5.26 | 39636.06 |
| RF1_ANAD2_1 | B8JAU2 | 5.66 | 41158.39 |
| RF1_CHLL2_1 | B3EE55 | 5.54 | 40797.97 |
| RF1_BREBN_1 | C0Z828 | 5.33 | 40440.6 |
| RF1_BRUA1_1 | B2S825 | 4.99 | 39924.92 |
| RF1_CHLP8_1 | B3QRH7 | 5.42 | 40467.6 |
| RF1_ANAPZ_1 | Q2GKS8 | 7.77 | 40717.21 |
| RF1_AQUAE_1 | O67032 | 6.62 | 41833.78 |
| RF1_CHLSY_1 | B9LJ02 | 4.97 | 40585.51 |
| RF1_MACCJ_1 | B9E8G1 | 4.82 | 40653.61 |
| RF1_HYPNA_1 | Q0C4I8 | 5.14 | 38910.9 |
| RF1_BURM7_1 | A3MFT4 | 5.65 | 40483.49 |
| RF1_FINM2_1 | B0S2B6 | 4.91 | 40471.77 |
| RF1_BURP0_1 | A3NZS4 | 5.57 | 40484.48 |
| RF1_ACAM1_1 | B0C424 | 4.89 | 40510.64 |
| RF1_DEHMC_1 | Q3ZYA9 | 5.14 | 40649.05 |
| RF1_FRATM_1 | B2SFF6 | 5.09 | 40416.4 |
| RF1_DESAG_1 | Q30X18 | 5.38 | 40448.66 |
| RF1_ACICJ_1 | A5FX99 | 5.36 | 39103.15 |
| RF1_METPP_1 | A2SD46 | 5.93 | 39531.54 |
| RF1_GEOMG_1 | Q39YQ1 | 5.15 | 40174.41 |
| RF1_DESVM_1 | B8DLL6 | 5.38 | 40312.19 |
| RF1_AGARV_1 | C4Z911 | 4.94 | 40687.9 |
| RF1_AGRFC_1 | Q8U8B8 | 5.19 | 40044.22 |
| RF1_POLAQ_1 | A4SV43 | 5.37 | 40284.26 |
| RF1_MYCUA_1 | A0PUL1 | 5.04 | 39149.57 |
| RF1_EDWI9_1 | C5B814 | 5.24 | 40313.19 |
| RF1_MYXXA_1 | Q93NC7 | 5.59 | 40142.34 |
| RF1_SHESM_1 | Q0HFC8 | 4.93 | 40315.15 |
| RF1_MYCPE_1 | Q8EWY5 | 5.83 | 41021.77 |
| RF1_NAUPA_1 | B9L5Z9 | 5.16 | 40236.94 |
| RF1_STRPB_1 | Q1JBR9 | 4.89 | 40599.6 |
| RF1_SHIB3_1 | B2TZV9 | 5.23 | 40516.36 |
| RF1_NEIMA_1 | Q9JT75 | 5.03 | 39718.61 |
| RF1_THISH_1 | B8GLA1 | 5.18 | 40117.9 |
| RF1_STRPJ_1 | B8ZPH1 | 4.85 | 40629.68 |
| RF1_RICM5_1 | A8F1S1 | 5.72 | 40331.45 |
| RF1_UREPA_1 | Q9PRE0 | 5.27 | 40577.32 |
| RF1_STRS7_1 | C0MF07 | 4.9 | 40468.5 |
| RF1_STRTD_1 | Q03L79 | 4.91 | 40677.78 |
| RF1_OCEIH_1 | Q8EM60 | 4.86 | 40497.36 |
| RF1_SACD2_1 | Q21FM3 | 5.04 | 39880.81 |
| RF1_ORITB_1 | A5CCS8 | 6.35 | 40572.05 |
| RF1_PSEFS_1 | C3KDC6 | 5.05 | 39881.59 |
| RF1_PARDP_1 | A1B0G1 | 5.29 | 38743.65 |
| RF1_PARL1_1 | A7HTY3 | 5.28 | 39452.5 |
| RF1_SALG2_1 | B5R924 | 5.13 | 40446.31 |
| RF1_XANAC_1 | Q8PNU5 | 5.37 | 39876.51 |
| RF1_XANC5_1 | Q3BX07 | 5.31 | 39879.52 |
| RF1_SALPA_1 | Q5PCQ9 | 5.13 | 40477.28 |
| RF1_PECCP_1 | C6DHY1 | 5.03 | 40368.19 |
| RF1_PSEPW_1 | B1JEP6 | 5 | 40187.94 |
| RF1_PELPB_1 | B4SAR5 | 5.68 | 40715.89 |
| RF1_SALTI_1 | P0A288 | 5.13 | 40461.28 |
| RF1_SALTY_1 | P0A287 | 5.13 | 40461.28 |
| RF1_SYNSC_1 | Q3AMQ9 | 5 | 40699.39 |
| RF1_SHEAM_1 | A1S8R2 | 5.06 | 40542.34 |
| RF1_PSYCK_1 | Q1QAW3 | 4.79 | 40582.51 |
| RF1_SHEB5_1 | A3D0G1 | 4.95 | 40505.4 |
| RF1_YERPA_1 | Q1C854 | 5.06 | 40563.58 |
| RF1_THEEB_1 | Q8DMG9 | 5.42 | 41197.41 |
| RF1_SHEDO_1 | Q12QR8 | 4.97 | 40297.43 |
| RF1_SHEHH_1 | B0TR27 | 4.88 | 40599.4 |
| RF1_RHOFT_1 | Q21YY9 | 5.35 | 40625.73 |
| RF1_LACLM_1 | A2RIR4 | 4.93 | 40448.7 |
| RF1_LACP3_1 | Q03A29 | 4.92 | 40962.92 |
| RF1_LACPL_1 | Q88UT2 | 4.89 | 41068.82 |
| RF1_LACRJ_1 | B2G676 | 4.82 | 41026.84 |
| RF1_LEGPL_1 | Q5WUB1 | 5.08 | 41102.95 |
| RF1_LEPBL_1 | Q056R9 | 5.59 | 39997.26 |
| RF1_LEUCK_1 | B1MVN7 | 4.78 | 40203.14 |
| RF1_LEUMM_1 | Q03VA1 | 4.88 | 40344.29 |
| RF1_LISIN_1 | Q927V0 | 4.88 | 40722.53 |
| RF1_CELJU_1 | B3PJP4 | 4.96 | 40285.12 |
| RF1_ALKEH_1 | Q0AC04 | 5.11 | 40466.99 |
| RF1_ANADF_1 | A7HI45 | 5.55 | 41513.73 |
| RF1_CHLMU_1 | Q9PL16 | 5.34 | 40207.27 |
| RF1_CHLPD_1 | A1BJT9 | 5.95 | 40999.38 |
| RF1_ANASK_1 | B4UAY8 | 5.67 | 41172.42 |
| RF1_ANAVT_1 | Q3MF91 | 5.15 | 41233.41 |
| RF1_CHLT2_1 | B0B9D0 | 5.29 | 40022.11 |
| RF1_CHLT3_1 | B3QYI4 | 5.29 | 40709.7 |
| RF1_MANSM_1 | Q65TB1 | 4.86 | 40766.63 |
| RF1_ENT38_1 | A4WBC6 | 5.04 | 40375.04 |
| RF1_CUPNJ_1 | Q46WS9 | 5.07 | 39990.75 |
| RF1_BURCM_1 | Q0BIQ0 | 5.59 | 40641.65 |
| RF1_MARMS_1 | A6W1C2 | 4.93 | 40361.39 |
| RF1_EXISA_1 | C4KYV2 | 5.12 | 40400.62 |
| RF1_DECAR_1 | Q479R5 | 5.25 | 40029.1 |
| RF1_BACP2_1 | A8FID1 | 5.11 | 40398.45 |
| RF1_KOCRD_1 | B2GLX4 | 5.26 | 40651.27 |
| RF1_KORVE_1 | Q1II28 | 5.88 | 40612.72 |
| RF1_LACCB_1 | B3WDK7 | 4.92 | 40962.92 |
| RF1_CLOCE_1 | B8I562 | 5.09 | 41032 |
| RF1_BART1_1 | A9IMM3 | 5.61 | 40333.68 |
| RF1_FUSNN_1 | Q8R5W0 | 5.28 | 41863.79 |
| RF1_BIFAA_1 | A1A0I9 | 4.81 | 40212.85 |
| RF1_ACTP7_1 | B3GZJ0 | 4.9 | 40728.44 |
| RF1_METS4_1 | B0UAV4 | 5.25 | 39077.05 |
| RF1_AERS4_1 | A4SK64 | 5.06 | 40801.49 |
| RF1_CORGB_1 | A4QDG1 | 4.78 | 39677.04 |
| RF1_MICAN_1 | B0JYC5 | 4.99 | 41132.28 |
| RF1_AKKM8_1 | B2UL99 | 5.13 | 40973.11 |
| RF1_GRABC_1 | Q0BUJ8 | 5.21 | 39010.91 |
| RF1_MYCSS_1 | Q1B542 | 4.92 | 38831.01 |
| RF1_MYCMM_1 | B2HQL3 | 4.94 | 39030.44 |
| RF1_POLSJ_1 | Q12F86 | 5.22 | 40936.13 |
| RF1_MYCPN_1 | P75420 | 6.14 | 40811.58 |
| RF1_NEIG1_1 | Q5F750 | 4.95 | 39779.61 |
| RF1_PROM2_1 | A8G733 | 5.2 | 40823.15 |
| RF1_TOLAT_1 | C4LBL5 | 4.95 | 40786.7 |
| RF1_PROMH_1 | B4EVR7 | 4.87 | 40557.47 |
| RF1_SINMW_1 | A6UCF2 | 5.24 | 39950.91 |
| RF1_NITHX_1 | Q1QQY7 | 5.81 | 39632.82 |
| RF1_NITOC_1 | Q3JDR4 | 5.38 | 40294.31 |
| RF1_NITSB_1 | A6Q162 | 5.27 | 40359.67 |
| RF1_RICRO_1 | B0BXT9 | 5.72 | 39729.88 |
| RF1_STAA1_1 | A7X4W4 | 4.82 | 40349.93 |
| RF1_ROSDO_1 | Q164F8 | 4.91 | 38577.43 |
| RF1_RUTMC_1 | A1AX02 | 7.22 | 41010.75 |
| RF1_ONYPE_1 | Q6YRK0 | 8.62 | 40010.93 |
| RF1_PSEE4_1 | Q1IEX1 | 4.98 | 40100.9 |
| RF1_PARD8_1 | A6LDR9 | 5.06 | 41606.94 |
| RF1_SALEP_1 | B5R3J9 | 5.13 | 40461.28 |
| RF1_SYNE7_1 | Q31L36 | 4.99 | 41574.86 |
| RF1_PARXL_1 | Q13TU2 | 5.5 | 40353.38 |
| RF1_PECAS_1 | Q6D551 | 5.08 | 40290.12 |
| RF1_XANP2_1 | A7IIM2 | 5.11 | 39437.23 |
| RF1_STRAW_1 | Q82J70 | 5.04 | 39503.06 |
| RF1_SYNWW_1 | Q0AUB8 | 5.18 | 40230.36 |
| RF1_YERE8_1 | A1JRU0 | 5.06 | 40539.41 |
| RF1_PHOPR_1 | Q6LNB4 | 4.99 | 40553.33 |
| RF1_YERPB_1 | B2K2Z0 | 5.06 | 40563.58 |
| RF1_RALPJ_1 | B2UDM7 | 5.24 | 40001.85 |
| RF1_ZYMMO_1 | Q5NMC7 | 5.04 | 39320.14 |
| RF1_SHELP_1 | A3QH30 | 4.83 | 40231.1 |
| RF1_RHOBA_1 | Q7ULT3 | 5.08 | 40794.54 |
| RF1_LACF3_1 | B2GAT1 | 4.68 | 41068.76 |
| RF1_LACH4_1 | A8YUJ1 | 5 | 41575.83 |
| RF1_LACJO_1 | Q74K25 | 4.86 | 41598.67 |
| RF1_LACP7_1 | A9KHD4 | 4.98 | 40664.92 |
| RF1_LACS1_1 | Q1WUD6 | 4.86 | 41056.13 |
| RF1_LARHH_1 | C1D5R7 | 5.11 | 39921.8 |
| RF1_LEIXX_1 | Q6AG71 | 4.84 | 39745.24 |
| RF1_LEPBA_1 | B0SH84 | 5.44 | 39501.65 |
| RF1_LEPCP_1 | B1XYX1 | 5.34 | 39585.3 |
| RF1_LEPIC_1 | Q72W38 | 5.49 | 40099.28 |
| RF1_ENTFA_1 | Q831F6 | 4.84 | 40247.28 |
| RF1_HYDS0_1 | B4U955 | 5.83 | 40674.09 |
| RF1_BURM1_1 | A9AJ68 | 5.6 | 40640.71 |
| RF1_CLOB8_1 | A6LQG2 | 5.03 | 40993.31 |
| RF1_FRACC_1 | Q2J6M0 | 5 | 38701.21 |
| RF1_BACSK_1 | Q5WB55 | 5.05 | 40431.46 |
| RF1_ACIC1_1 | A0LSK4 | 5.26 | 40237.06 |
| RF1_CAMLR_1 | B9KE98 | 5.16 | 40011.37 |
| RF1_CLOTH_1 | A3DIL4 | 5.19 | 41024.11 |
| RF1_DICT6_1 | B5YDB2 | 5.29 | 42442.19 |
| RF1_GEOTN_1 | A4ITK8 | 5.04 | 40735.99 |
| RF1_CORDI_1 | Q6NHU0 | 4.67 | 39414.79 |
| RF1_COREF_1 | Q8FQ31 | 4.84 | 39862.3 |
| RF1_GLOVI_1 | Q7ND15 | 5.39 | 41131.26 |
| RF1_CORJK_1 | Q4JUI9 | 4.75 | 39968.25 |
| RF1_MYCA5_1 | B3PMD5 | 5.31 | 40234.79 |
| RF1_MYCAP_1 | A5IZH6 | 5.25 | 40471.96 |
| RF1_MYCBP_1 | A1KI87 | 5.06 | 39036.48 |
| RF1_HAEPS_1 | B8F526 | 4.93 | 40598.35 |
| RF1_MYCGE_1 | P47500 | 6.44 | 40807.69 |
| RF1_HAHCH_1 | Q2SL95 | 4.99 | 40361.42 |
| RF1_MYCH2_1 | Q601F9 | 7.75 | 40869.99 |
| RF1_RHOJR_1 | Q0SGN8 | 4.84 | 38941.22 |
| RF1_HELAH_1 | Q17VT1 | 5.52 | 39672.1 |
| RF1_POLNS_1 | B1XSY0 | 5.31 | 40419.42 |
| RF1_SHIDS_1 | Q32GZ6 | 5.13 | 40517.3 |
| RF1_THIDA_1 | Q3SG10 | 5.08 | 39692.38 |
| RF1_NEOSM_1 | Q2GE03 | 6.31 | 41691.48 |
| RF1_SHISS_1 | Q3Z0S9 | 5.13 | 40517.3 |
| RF1_TREPA_1 | O83090 | 6 | 40194.48 |
| RF1_UNCTG_1 | B1GZI5 | 6.26 | 40866.42 |
| RF1_UREU1_1 | B5ZAH6 | 5.27 | 40487.1 |
| RF1_SPHWW_1 | A5V9C3 | 5.22 | 39444.42 |
| RF1_NOVAD_1 | Q2GBN7 | 5.13 | 38633.52 |
| RF1_PSECP_1 | B8HB03 | 4.93 | 39604.07 |
| RF1_VIBTL_1 | B7VKH3 | 4.97 | 40364.2 |
| RF1_SULNB_1 | A6Q6B4 | 4.87 | 39609.71 |
| RF1_SALCH_1 | Q57NN5 | 5.13 | 40461.28 |
| RF1_PARP8_1 | B2JHK8 | 5.3 | 40554.51 |
| RF1_PARUW_1 | Q6MDH3 | 5.4 | 41477.76 |
| RF1_STAES_1 | Q8CNI7 | 4.79 | 40377.95 |
| RF1_STAS1_1 | Q49Z65 | 4.86 | 40733.41 |
| RF1_PELTS_1 | A5CYC3 | 5.25 | 39523.74 |
| RF1_STRAG_1 | Q9AG29 | 4.93 | 40672.7 |
| RF1_PEPD6_1 | Q180Y2 | 5.13 | 40022.28 |
| RF1_STRCO_1 | Q9K4E4 | 4.99 | 39459.97 |
| RF1_PHYAS_1 | B1V950 | 8.94 | 40358.31 |
| RF1_STRMK_1 | B2FQ14 | 5.31 | 39911.69 |
| RF1_SHEON_1 | Q8EAR3 | 4.93 | 40344.15 |
| RF1_CHESB_1 | Q11E04 | 5.05 | 40143.1 |
| RF1_CHLAB_1 | Q5L5L4 | 5.36 | 40559.66 |
| RF1_CHLAD_1 | B8G7K1 | 5 | 40755.68 |
| RF1_BRADU_1 | Q89XT9 | 5.44 | 39628.75 |
| RF1_BRAHW_1 | C0QWA1 | 5.4 | 40876.42 |
| RF1_ANAMF_1 | B9KIV2 | 5.66 | 40599.16 |
| RF1_BRUSI_1 | B0CIC3 | 4.99 | 39924.92 |
| RF1_ARTS2_1 | A0JY78 | 4.94 | 39619.18 |
| RF1_CHLTE_1 | Q8KG45 | 5.67 | 40600.78 |
| RF1_CHRSD_1 | Q1QXC6 | 5.16 | 40561.16 |
| RF1_BACCN_1 | A7GV79 | 5 | 40430.49 |
| RF1_ERWT9_1 | B2VEH7 | 4.97 | 40324.92 |
| RF1_BURL3_1 | Q39K18 | 5.51 | 40593.56 |
| RF1_ESCF3_1 | B7LSH9 | 5.13 | 40517.3 |
| RF1_JANMA_1 | A6SUF6 | 5.21 | 40225.37 |
| RF1_BACHD_1 | Q9K6F4 | 4.9 | 40597.54 |
| RF1_FLAJ1_1 | A5FHI8 | 4.96 | 40491.84 |
| RF1_DEIDV_1 | C1CWJ1 | 5.07 | 40043.89 |
| RF1_FRASN_1 | A8L3V1 | 5.22 | 38750.33 |
| RF1_DELAS_1 | A9BP60 | 5.17 | 40150.02 |
| RF1_LACBA_1 | Q03QX7 | 4.68 | 40844.56 |
| RF1_BDEBA_1 | Q6MRK9 | 5.52 | 40112.44 |
| RF1_BEII9_1 | B2IHL7 | 5.08 | 39537.44 |
| RF1_GEOLS_1 | B3E628 | 5.12 | 40215.32 |
| RF1_CARHZ_1 | Q3A928 | 5.19 | 40781.03 |
| RF1_AERHH_1 | A0KMZ8 | 4.99 | 40861.46 |
| RF1_METSB_1 | B8EI32 | 5.25 | 39868.77 |
| RF1_DICTD_1 | B8E003 | 5.16 | 42486.16 |
| RF1_GEOUR_1 | A5G8T7 | 5.12 | 39718.82 |
| RF1_GLUDA_1 | A9HJ50 | 5.17 | 39263.21 |
| RF1_GLUOX_1 | Q5FUR9 | 5.03 | 39444.22 |
| RF1_GRAFK_1 | A0LYL5 | 4.92 | 40697.79 |
| RF1_HAEDU_1 | Q7VMV9 | 5 | 40613.51 |
| RF1_HALHL_1 | A1WVQ8 | 5.17 | 40310.85 |
| RF1_SHEPC_1 | A4Y3J8 | 4.92 | 40400.22 |
| RF1_MYCMO_1 | Q6KIA0 | 5.88 | 40513.94 |
| RF1_MYCVP_1 | A1TD66 | 4.81 | 38931.09 |
| RF1_SHESH_1 | A8FYZ0 | 4.81 | 40454.27 |
| RF1_RHOS1_1 | A3PJZ6 | 5.25 | 38566.74 |
| RF1_SHEWM_1 | B1KDU3 | 4.78 | 40500.35 |
| RF1_MYCS5_1 | Q4A6L9 | 5.38 | 40557.18 |
| RF1_RICAH_1 | A8GNQ4 | 5.61 | 39701.82 |
| RF1_RICBR_1 | Q1RJT5 | 5.56 | 40217.28 |
| RF1_RICFE_1 | Q4ULG0 | 5.62 | 39657.72 |
| RF1_SOLUE_1 | Q01RX8 | 5.46 | 40745.17 |
| RF1_VARPS_1 | C5CXW3 | 5.33 | 40526.52 |
| RF1_RICTY_1 | Q68WK7 | 6.5 | 40062.4 |
| RF1_VESOH_1 | A5CW65 | 6.48 | 40680.23 |
| RF1_NOSS1_1 | Q8YPK9 | 5.09 | 41235.41 |
| RF1_OLICO_1 | B6JA06 | 5.29 | 39300.32 |
| RF1_SALA4_1 | B5F4H6 | 5.13 | 40461.28 |
| RF1_WOLPM_1 | Q73IC2 | 5.85 | 41208.69 |
| RF1_WOLSU_1 | Q7M9M4 | 5.27 | 39961.24 |
| RF1_PARPJ_1 | B2SZ27 | 5.5 | 40353.38 |
| RF1_SALRD_1 | Q2S2U3 | 4.89 | 40292.8 |
| RF1_PELPD_1 | A1ASD1 | 5.09 | 40065.14 |
| RF1_PSEU2_1 | Q4ZXW7 | 4.99 | 40024.74 |
| RF1_PELUB_1 | Q4FN99 | 5.52 | 40413.61 |
| RF1_SERP5_1 | A8GDA3 | 5.05 | 40370.37 |
| RF1_PSYA2_1 | Q4FSV3 | 4.78 | 40512.38 |
| RF1_RENSM_1 | A9WNB3 | 4.93 | 39811.37 |
| RF1_RHILO_1 | Q98G93 | 5.08 | 39745.76 |
| RF1_RHIME_1 | Q92MK5 | 5.24 | 40030.04 |
| RF1_LISMH_1 | B8DBG6 | 4.91 | 40679.5 |
| RF1_ALIF1_1 | Q5E6T3 | 4.92 | 40403.2 |
| RF1_ALKOO_1 | A8MJX9 | 4.92 | 40560.79 |
| RF1_ALTMD_1 | B4RSV6 | 4.78 | 40416.92 |
| RF1_CHLPM_1 | A4SCF1 | 5.47 | 41041.17 |
| RF1_CHLPN_1 | Q9Z968 | 5.63 | 40150.49 |
| RF1_CUPTR_1 | B3R722 | 5.1 | 40029.81 |
| RF1_ERYLH_1 | Q2N5U6 | 5.1 | 38724.71 |
| RF1_CYTH3_1 | Q11NS4 | 5.12 | 40595.84 |
| RF1_JANSC_1 | Q28RD9 | 4.8 | 38962.94 |
| RF1_ACIAC_1 | A1TTC4 | 5.26 | 40087.01 |
| RF1_DEIRA_1 | P56905 | 4.99 | 41126.15 |
| RF1_LACAC_1 | Q5FKZ0 | 4.92 | 41522.64 |
| RF1_DESAA_1 | B8FEA2 | 5.3 | 39693.73 |
| RF1_LACDB_1 | Q04BB3 | 4.85 | 41210.29 |
| RF1_ACIC5_1 | C1F2J1 | 5.31 | 40628.8 |
| RF1_METPB_1 | B1Z914 | 5.2 | 39324.23 |
| RF1_GEOKA_1 | Q5KUH3 | 4.97 | 40789.01 |
| RF1_CLOTE_1 | Q898Y5 | 4.97 | 40840.97 |
| RF1_BIFLS_1 | B7GP74 | 4.73 | 40039.62 |
| RF1_GEOSW_1 | C5D9N4 | 5.01 | 40985.26 |
| RF1_BLOPB_1 | Q492V8 | 8.37 | 41284.32 |
| RF1_DINSH_1 | A8LLU7 | 5.17 | 38295.32 |
| RF1_AGRRK_1 | B9JB75 | 5.07 | 39853.82 |
| RF1_AGRVS_1 | B9JT13 | 5.29 | 39687.86 |
| RF1_MYCA1_1 | A0QCW7 | 4.93 | 39303.61 |
| RF1_CORK4_1 | C4LJM5 | 4.73 | 39666.91 |
| RF1_MYCA9_1 | B1MLV1 | 4.87 | 38804.12 |
| RF1_SHEPA_1 | A8H7A5 | 4.84 | 40319.12 |
| RF1_MYCPA_1 | Q73X48 | 4.93 | 39313.64 |
| RF1_MYCPU_1 | Q98RA5 | 5.79 | 40530.15 |
| RF1_THEYD_1 | B5YIQ7 | 6.33 | 40283.15 |
| RF1_SHIF8_1 | Q0T5I4 | 5.13 | 40517.3 |
| RF1_TREDE_1 | Q73JU6 | 5.31 | 41352.87 |
| RF1_TRIEI_1 | Q110D7 | 4.91 | 41474.7 |
| RF1_RICPR_1 | Q9ZD21 | 6.31 | 39895.12 |
| RF1_SPHAL_1 | Q1GPK3 | 5.2 | 38473.55 |
| RF1_NOCFA_1 | Q5Z0Z2 | 4.93 | 39024.4 |
| RF1_NOCSJ_1 | A1SHH9 | 4.85 | 39286.73 |
| RF1_VIBCB_1 | A7MY68 | 4.87 | 40295.93 |
| RF1_OCHA4_1 | A6WXQ3 | 5.1 | 39777.77 |
| RF1_SYMTH_1 | Q67TD5 | 5.41 | 40562.79 |
| RF1_WOLTR_1 | Q5GSS8 | 5.91 | 41429.01 |
| RF1_SALHS_1 | B4TKA4 | 5.13 | 40461.28 |
| RF1_SALNS_1 | B4SUG2 | 5.13 | 40461.28 |
| RF1_PASMU_1 | P57852 | 5.02 | 40652.54 |
| RF1_STAHJ_1 | Q4L7Z9 | 4.8 | 40486.14 |
| RF1_XANOM_1 | Q2NZW2 | 5.36 | 39961.63 |
| RF1_PSEU5_1 | A4VPB9 | 5.01 | 40220.02 |
| RF1_XYLFM_1 | B0U5Z3 | 5.89 | 40613.57 |
| RF1_PERMH_1 | C0QTI3 | 5.88 | 41728.48 |
| RF1_PSYIN_1 | A1SV89 | 5.09 | 40457.37 |
| RF1_STRGC_1 | P47850 | 4.85 | 40700.69 |
| RF1_STRMU_1 | Q8DU64 | 4.92 | 40650.64 |
| RF1_THEP3_1 | B0K7G6 | 5.1 | 40540.9 |
| RF1_BORBR_1 | Q7WQE9 | 5.2 | 40130.24 |
| RF1_BORPD_1 | A9IER8 | 5.27 | 40285.32 |
| RF1_BORPE_1 | Q7W023 | 5.2 | 40130.24 |
| RF1_BORT9_1 | A1QYZ0 | 6.03 | 41671.34 |
| RF1_CHLL7_1 | Q3B6R6 | 5.55 | 41104.24 |
| RF1_AZOSB_1 | A1K443 | 5.25 | 39977.79 |
| RF1_MAGSA_1 | Q2W6V3 | 5.15 | 39538.4 |
| RF1_CHRVO_1 | Q7P1Y2 | 5.02 | 39513.5 |
| RF1_EUBE2_1 | C4Z1S4 | 4.82 | 40097.12 |
| RF1_EXIS2_1 | B1YEH9 | 5.35 | 39681.74 |
| RF1_METFK_1 | Q1GYE8 | 5.1 | 40332.18 |
| RF1_BACWK_1 | A9VSC7 | 5.09 | 40341.35 |
| RF1_BARQU_1 | Q6G0R0 | 5.35 | 40355.86 |
| RF1_DESDA_1 | B8J4R4 | 5.27 | 40054.01 |
| RF1_CLONN_1 | A0Q312 | 5.11 | 40590.85 |
| RF1_METNO_1 | B8IUB4 | 5.19 | 39100 |
| RF1_DESOH_1 | A8ZTL8 | 5.63 | 40311.23 |
| RF1_DESPS_1 | Q6AJM5 | 5.3 | 40369.78 |
| RF1_COLP3_1 | Q47Y94 | 5.02 | 40848.79 |
| RF1_CORA7_1 | C3PFQ4 | 4.65 | 40184.65 |
| RF1_BORBP_1 | Q662G9 | 7.69 | 41680.47 |
| RF1_HAMD5_1 | C4K7Y8 | 5.95 | 40838.24 |
| RF1_HELHP_1 | Q7VFA0 | 5.31 | 39551.7 |
| RF1_HELMI_1 | B0TI71 | 5.11 | 39837.07 |
| RF1_PORGI_1 | Q7MXT5 | 5.17 | 40367.79 |
| RF1_PROA2_1 | B4S385 | 5.37 | 40810 |
| RF1_NITEC_1 | Q0AHU1 | 5.52 | 40335.35 |
| RF1_NITMU_1 | Q2Y5P3 | 5.99 | 40073.18 |
| RF1_SORC5_1 | A9G9L1 | 5.66 | 40630.19 |
| RF1_PSE14_1 | Q48MV4 | 4.99 | 40081.79 |
| RF1_STRSV_1 | A3CN05 | 4.88 | 40688.68 |
| RF1_NOSP7_1 | B2ITM5 | 4.98 | 40968.94 |
| RF1_STRU0_1 | B9DS45 | 4.81 | 40739.72 |
| RF1_SALDC_1 | B5FU19 | 5.13 | 40461.28 |
| RF1_PSEHT_1 | Q3IK95 | 4.97 | 40545.34 |
| RF1_PSEMY_1 | A4XR61 | 5.06 | 39954.68 |
| RF1_SYNFM_1 | A0LIZ8 | 5.83 | 40427.69 |
| RF1_PHOLL_1 | Q7N586 | 5.1 | 40532.35 |
| RF1_STRGG_1 | B1W0B6 | 5.1 | 39471.99 |
| RF1_THEFY_1 | Q47M70 | 5 | 39621.18 |
| RF1_THEPX_1 | B0K1F7 | 5.11 | 40599.92 |
| RF1_ALCBS_1 | Q0VS77 | 5.11 | 39993.53 |
| RF1_BORPA_1 | Q7WCE3 | 5.2 | 40158.29 |
| RF1_ALKMQ_1 | A6TK43 | 4.98 | 40304.62 |
| RF1_CHLCH_1 | Q3AUE2 | 5.26 | 40602.66 |
| RF1_EHRCJ_1 | Q3YS23 | 6 | 40884.2 |
| RF1_EHRCR_1 | Q2GGM5 | 6.2 | 40806.21 |
| RF1_HISS2_1 | B0UUE6 | 4.93 | 40802.52 |
| RF1_MARMM_1 | Q0ARZ8 | 4.98 | 39036.8 |
| RF1_IDILO_1 | Q5QUZ8 | 4.84 | 41120.5 |
| RF1_CLOAB_1 | Q97F68 | 5.17 | 40975.16 |
| RF1_METCA_1 | Q60A21 | 5.5 | 40508.35 |
| RF1_KINRD_1 | A6W7F6 | 5.14 | 38457.84 |
| RF1_BACLD_1 | Q65DV1 | 4.97 | 40377.27 |
| RF1_ACHLI_1 | A9NEP7 | 5 | 40085.21 |
| RF1_BURTA_1 | Q2SUG2 | 5.57 | 40528.53 |
| RF1_DEIGD_1 | Q1J0S3 | 5.05 | 40481.39 |
| RF1_CALS4_1 | Q8RD98 | 5.18 | 40632.19 |
| RF1_CAMC1_1 | A7ZB10 | 5.15 | 39763.91 |
| RF1_BARHE_1 | Q6G5T7 | 5.52 | 40450.97 |
| RF1_CLOK5_1 | A5N3J7 | 5.32 | 40882.25 |
| RF1_BAUCH_1 | Q1LTH5 | 5.73 | 41528.87 |
| RF1_DESMR_1 | C4XIQ1 | 5.5 | 39966.96 |
| RF1_BIFA0_1 | B8DTL4 | 4.79 | 40947.64 |
| RF1_ACISJ_1 | A1W4B8 | 5.31 | 40777.78 |
| RF1_METRJ_1 | B1M766 | 5.19 | 39335.4 |
| RF1_GEOSL_1 | Q748B1 | 5.15 | 40282.45 |
| RF1_BLOFL_1 | Q7VR74 | 9.08 | 41404.32 |
| RF1_BORAP_1 | Q0SNW8 | 6.66 | 41692.35 |
| RF1_MOOTA_1 | Q2RFW0 | 5.13 | 40572.77 |
| RF1_MYCCT_1 | P71496 | 5.31 | 41157.49 |
| RF1_MYCGA_1 | Q7NBX3 | 5.49 | 41021.82 |
| RF1_HAES1_1 | Q0I3B5 | 4.93 | 40812.56 |
| RF1_MYCGI_1 | A4T8L3 | 4.77 | 38763.94 |
| RF1_POLNA_1 | A1VKB6 | 5.42 | 41062.2 |
| RF1_SHEPW_1 | B8CQV2 | 4.79 | 40451.21 |
| RF1_MYCMS_1 | Q6MU89 | 5.25 | 41296.64 |
| RF1_RHORT_1 | Q2RWE1 | 5.25 | 38735.54 |
| RF1_NATTJ_1 | B2A3J0 | 4.72 | 41235.08 |
| RF1_RICAE_1 | C3PNN0 | 5.65 | 39763.89 |
| RF1_RICCK_1 | A8EYR1 | 5.96 | 39739.93 |
| RF1_RICCN_1 | Q92HK9 | 5.53 | 39745.83 |
| RF1_NITEU_1 | Q82TH4 | 5.65 | 40318.35 |
| RF1_SODGM_1 | Q2NRS4 | 5.29 | 40443.43 |
| RF1_RICPU_1 | C4K0C9 | 5.46 | 39737.81 |
| RF1_NITWN_1 | Q3SVP7 | 5.66 | 39436.6 |
| RF1_PSEA6_1 | Q15SQ9 | 4.89 | 40717.33 |
| RF1_VEREI_1 | A1WI56 | 6.06 | 40057.15 |
| RF1_RUBXD_1 | Q1AVG5 | 5.19 | 40789.31 |
| RF1_RUEPO_1 | Q5LQM7 | 5 | 38861.89 |
| RF1_RUEST_1 | Q1GI42 | 4.9 | 38675.51 |
| RF1_VIBPA_1 | Q87RN4 | 4.9 | 40294 |
| RF1_SULDN_1 | Q30NW2 | 5.18 | 39743.16 |
| RF1_SALAR_1 | A9MPA1 | 5.14 | 40475.31 |
| RF1_WIGBR_1 | Q8D2K9 | 9.57 | 42417.41 |
| RF1_SYNAS_1 | Q2LWU9 | 5.75 | 40046.33 |
| RF1_STACT_1 | B9DMD1 | 4.71 | 40494.07 |
| RF1_PELCD_1 | Q3A129 | 5.19 | 39944.92 |
| RF1_SALSV_1 | B4TXU2 | 5.13 | 40477.28 |
| RF1_SYNY3_1 | P74707 | 4.91 | 41126.09 |
| RF1_TERTT_1 | C5BSZ6 | 4.94 | 40351.19 |
| RF1_PSYWF_1 | A5WFG1 | 4.72 | 40986.61 |
| RF1_SHEFN_1 | Q087I4 | 4.89 | 40638.46 |
| RF1_THEMA_1 | Q9X183 | 5.35 | 39656.8 |
| RF1_RALSO_1 | Q8XVD0 | 5.36 | 40076.01 |
| RF1_RHIL3_1 | Q1MBB2 | 5.06 | 39995.1 |
| RF1_LACGA_1 | Q042K5 | 4.92 | 41540.67 |
| RF1_LACSS_1 | Q38WJ4 | 4.77 | 41433.39 |
| RF1_LAWIP_1 | Q1MSG7 | 5.38 | 40842.39 |
| RF1_LISW6_1 | A0ALM8 | 4.88 | 40722.53 |
| RF1_LYSSC_1 | B1I069 | 4.96 | 41073.19 |
| RF2 | Accession number | pI | MW |
| RF2_ECOLI_1 | P07012 | 4.64 | 41250.73 |
| RF2_THET8_1 | Q5SM01 | 5.33 | 42733.52 |
| RF2_BACSU_1 | P28367 | 5.03 | 42073.15 |
| RF2_MYCTU_1 | P9WHG1 | 4.82 | 41473.19 |
| RF2_LISIN_1 | Q927Y4 | 4.83 | 42036.09 |
| RF2_CHLPN_1 | P56906 | 5.16 | 42342.83 |
| RF2_LISMO_1 | Q8Y4D8 | 4.86 | 41996.09 |
| RF2_SYNY3_1 | P74476 | 4.73 | 41759.98 |
| RF2_YERPE_1 | Q8ZHK4 | 4.74 | 41396.12 |
| RF2_BUCAI_1 | P57511 | 8.52 | 42798.7 |
| RF2_SALTY_1 | P0A289 | 4.67 | 41148.68 |
| RF2_LACPL_1 | Q88YL5 | 4.91 | 42652.36 |
| RF2_STAAM_1 | Q99VM1 | 4.83 | 42331.22 |
| RF2_RICTY_1 | Q68X96 | 5.3 | 41973.35 |
| RF2_STAES_1 | Q8CPZ1 | 4.65 | 42734.44 |
| RF2_PASMU_1 | Q9CP66 | 4.79 | 41237.06 |
| RF2_STAHJ_1 | Q4L4H9 | 4.68 | 42556.17 |
| RF2_HAEDU_1 | Q7VNG1 | 4.75 | 41354.42 |
| RF2_STAS1_1 | Q49VV3 | 4.67 | 42693.38 |
| RF2_AERHH_1 | A0KNK6 | 4.73 | 41063.84 |
| RF2_ACHLI_1 | A9NF23 | 5.01 | 41586.78 |
| RF2_ACIC1_1 | A0LVP4 | 5.12 | 41611.72 |
| RF2_NEIG2_1 | B4RQU4 | 4.69 | 41362.16 |
| RF2_CAMC1_1 | A7ZE19 | 4.9 | 41208.14 |
| RF2_EXIS2_1 | B1YLH0 | 4.91 | 42141.29 |
| RF2_EXISA_1 | C4L5K9 | 4.84 | 41859.82 |
| RF2_CAMJR_1 | Q5HSX6 | 4.96 | 41069.97 |
| RF2_HELHP_1 | Q7VIE6 | 5.13 | 41478.36 |
| RF2_CAUSK_1 | B0SXB7 | 4.93 | 40251.74 |
| RF2_PSELT_1 | A8F531 | 5.23 | 42494.31 |
| RF2_RALPJ_1 | B2U8V1 | 4.94 | 41167.03 |
| RF2_CHLAB_1 | Q5L6U9 | 5.31 | 42273.73 |
| RF2_HELPS_1 | B2US00 | 5.01 | 41326.23 |
| RF2_RHOJR_1 | Q0S2Q6 | 4.7 | 41390.89 |
| RF2_HYPNA_1 | Q0C0Y0 | 5.01 | 40799.48 |
| RF2_CHLTA_1 | Q3KLN9 | 5.36 | 42608.34 |
| RF2_LACLA_1 | Q9CGX1 | 4.59 | 41569.61 |
| RF2_RUEST_1 | Q1GIB5 | 4.75 | 41457.14 |
| RF2_SACEN_1 | A4F8R2 | 4.78 | 41203.94 |
| RF2_CORGB_1 | A4QCD3 | 4.6 | 41136.9 |
| RF2_LEPIN_1 | Q8EZT4 | 5.54 | 42032.47 |
| RF2_CUTAK_1 | Q6A808 | 4.67 | 41141.76 |
| RF2_LEUMM_1 | Q03ZQ7 | 4.62 | 42343.81 |
| RF2_SHEWM_1 | B1KFR6 | 4.71 | 40995.65 |
| RF2_MYCA9_1 | B1MEU8 | 4.62 | 41087.7 |
| RF2_SULDN_1 | Q30TP0 | 5.06 | 41318.29 |
| RF2_SULSY_1 | B2V5M0 | 4.98 | 42608.37 |
| RF2_MYCMM_1 | B2HGJ2 | 4.79 | 41525.24 |
| RF2_MYCS2_1 | A0QU58 | 4.65 | 41242.82 |
| RF2_THEAB_1 | B7IE81 | 5.15 | 42844.54 |
| RF2_MYCSK_1 | A1UDD2 | 4.64 | 41520.03 |
| RF2_THEMA_1 | Q9X1R5 | 5.2 | 42637.27 |
| RF2_VARPS_1 | C5CUX0 | 5.04 | 41042.61 |
| RF2_WOLSU_1 | Q7MAP4 | 4.88 | 41747.61 |
| RF2_AQUAE_1 | O67695 | 5.46 | 43309.44 |
| RF2_NEIMB_1 | Q9JXB3 | 4.69 | 41489.27 |
| RF2_BURCJ_1 | B4EDB1 | 4.96 | 41080.77 |
| RF2_NEOSM_1 | Q2GDG2 | 5.67 | 41763.57 |
| RF2_BURP0_1 | A3NX26 | 5.04 | 41090.67 |
| RF2_PECAS_1 | Q6D946 | 4.67 | 41171.73 |
| RF2_PECCP_1 | C6D8Z5 | 4.69 | 41133.69 |
| RF2_FRACC_1 | Q2JEW7 | 4.76 | 40488.97 |
| RF2_PHEZH_1 | B4RCR3 | 4.87 | 40677.9 |
| RF2_HAEIN_1 | P43918 | 4.68 | 41281.02 |
| RF2_HELAH_1 | Q17YT5 | 5.15 | 41285.35 |
| RF2_CAMLR_1 | B9KEY7 | 4.94 | 41266.25 |
| RF2_CAUVC_1 | Q9A752 | 4.91 | 40922.26 |
| RF2_RENSM_1 | A9WPT6 | 4.75 | 41298.14 |
| RF2_CHLAD_1 | B8G607 | 5 | 41731.79 |
| RF2_KOSOT_1 | C5CGS4 | 5.03 | 42512.82 |
| RF2_RICPR_1 | Q9ZDQ2 | 5.71 | 42094.59 |
| RF2_CLOP1_1 | Q0TNE1 | 5.17 | 40968.35 |
| RF2_COLP3_1 | Q47WT4 | 4.73 | 41113.81 |
| RF2_LEPBJ_1 | Q04V47 | 5.47 | 42149.71 |
| RF2_SALPA_1 | Q5PJG1 | 4.67 | 41148.68 |
| RF2_LEUCK_1 | B1MW26 | 4.61 | 41973.46 |
| RF2_SALTO_1 | A4X3K6 | 4.72 | 41382.12 |
| RF2_STRAW_1 | Q82D82 | 4.72 | 41106.84 |
| RF2_SHIFL_1 | P66025 | 4.64 | 41220.7 |
| RF2_MYCA1_1 | A0QJS1 | 4.72 | 41276.91 |
| RF2_THEP1_1 | A5IM04 | 5.15 | 42493.01 |
| RF2_THERP_1 | B9L0E3 | 5.1 | 42358.78 |
| RF2_TOLAT_1 | C4LCN9 | 4.77 | 41210.91 |
| RF2_TREPA_1 | O83585 | 7.68 | 41596.1 |
| RF2_XANC5_1 | Q3BUB7 | 4.84 | 41735.53 |
| RF2_YERPS_1 | Q666T2 | 4.74 | 41440.16 |
| RF2_ACIET_1 | B9MBK7 | 5.03 | 41518.47 |
| RF2_AERS4_1 | A4SJK1 | 4.73 | 41083.81 |
| RF2_AROAE_1 | Q5NZU2 | 4.94 | 41546.27 |
| RF2_BORBR_1 | Q7WK44 | 4.87 | 41465.12 |
| RF2_MYCVP_1 | A1T6D1 | 4.65 | 41221.77 |
| RF2_BORPA_1 | Q7W8T4 | 4.87 | 41493.18 |
| RF2_BORPE_1 | Q7VZ35 | 4.87 | 41465.12 |
| RF2_BURMA_1 | Q62J00 | 5.04 | 41090.67 |
| RF2_NITSB_1 | A6Q582 | 5 | 42143.32 |
| RF2_ESCF3_1 | B7LPA4 | 4.64 | 41220.7 |
| RF2_CAMFF_1 | A0RQM7 | 5.17 | 41999.16 |
| RF2_CAMHC_1 | A7I0P7 | 5.43 | 41416.84 |
| RF2_PSEAE_1 | P0C348 | 12.23 | 41276.73 |
| RF2_PSECP_1 | B8HBH8 | 4.66 | 41144.91 |
| RF2_PSEHT_1 | Q3ILD8 | 4.7 | 40951.38 |
| RF2_RHOE4_1 | C0ZXD0 | 4.66 | 41244.93 |
| RF2_CHLMU_1 | P58105 | 5.44 | 42716.44 |
| RF2_RHOOB_1 | C1B1L3 | 4.7 | 41434.95 |
| RF2_JANMA_1 | A6SXF4 | 4.88 | 41635.5 |
| RF2_RICBR_1 | Q1RIY5 | 5.14 | 41378.54 |
| RF2_KLEP3_1 | B5XUF3 | 4.67 | 41203.86 |
| RF2_RICFE_1 | Q4UKS1 | 5.03 | 41628.69 |
| RF2_SALAI_1 | A8M3R1 | 4.74 | 41417.12 |
| RF2_STRGC_1 | A8AY61 | 4.65 | 41503.52 |
| RF2_STRGG_1 | B1VV08 | 4.7 | 40811.46 |
| RF2_STRM5_1 | B4SHV0 | 4.91 | 41720.51 |
| RF2_STRPG_1 | A2RFN2 | 4.74 | 41733.88 |
| RF2_STRPN_1 | Q2MGI2 | 4.77 | 41359.53 |
| RF2_MYCBO_1 | P66027 | 4.82 | 41473.19 |
| RF2_MYCGI_1 | A4TDE5 | 4.62 | 41470.96 |
| RF2_SULNB_1 | A6Q718 | 4.93 | 41535.45 |
| RF2_MYCLE_1 | O32885 | 4.98 | 41871.85 |
| RF2_SYNPX_1 | Q7U3W6 | 4.55 | 41775.6 |
| RF2_THEM4_1 | A6LN93 | 5.07 | 43068.86 |
| RF2_ACIF2_1 | B7J4M2 | 4.98 | 40566.58 |
| RF2_ACISJ_1 | A1W9H4 | 5.03 | 41518.47 |
| RF2_ALIF1_1 | Q5E7P9 | 4.69 | 41290.88 |
| RF2_MYCUA_1 | A0PQZ6 | 4.79 | 41555.26 |
| RF2_BORBU_1 | O51101 | 8.57 | 41652.37 |
| RF2_NAUPA_1 | B9L5Y7 | 4.97 | 42165.38 |
| RF2_NOCFA_1 | Q5YR52 | 4.85 | 41969.71 |
| RF2_OENOB_1 | Q04GC3 | 4.7 | 42212.81 |
| RF2_PETMO_1 | A9BHH8 | 4.92 | 42226.61 |
| RF2_PROMH_1 | B4F0M7 | 4.76 | 41223.96 |
| RF2_RICAE_1 | C3PMX2 | 5.19 | 41576.79 |
| RF2_KINRD_1 | A6WEK6 | 4.68 | 40785.28 |
| RF2_ROSDO_1 | Q165J6 | 4.74 | 41476.06 |
| RF2_CORA7_1 | C3PES4 | 4.68 | 40945.61 |
| RF2_CORDI_1 | Q6NIM2 | 4.61 | 41329.09 |
| RF2_SALTI_1 | P0A290 | 4.67 | 41148.68 |
| RF2_CYAP8_1 | B7K0A6 | 4.84 | 41144.28 |
| RF2_SHEB2_1 | B8E5Q1 | 4.73 | 41150.83 |
| RF2_ARCB4_1 | A8EWG5 | 5.01 | 41380.29 |
| RF2_ARTS2_1 | A0JYC5 | 4.61 | 41142.71 |
| RF2_BEUC1_1 | C5BYB6 | 4.68 | 41353.23 |
| RF2_BORA1_1 | Q2L224 | 4.93 | 41451.15 |
| RF2_STRA1_1 | Q3K2F4 | 4.68 | 41725.86 |
| RF2_STRCO_1 | Q53915 | 4.78 | 41084.76 |
| RF2_MAGSA_1 | Q2W3F8 | 4.91 | 41728.6 |
| RF2_STRU0_1 | B9DRN6 | 4.69 | 41791.95 |
| RF2_THESQ_1 | B1LB88 | 5.2 | 42449.02 |
| RF2_YERE8_1 | A1JPL5 | 4.68 | 41171.87 |
| RF3 | Accession number | pI | MW |
| RF3_ECOLI_1 | P0A7I4 | 5.65 | 59442.89 |
| RF3_SALTY_1 | Q56121 | 5.54 | 59431.84 |
| RF3_VIBCH_1 | Q9KU64 | 5.6 | 59626.02 |
| RF3_COXBU_1 | Q83DC7 | 6.32 | 59477.14 |
| RF3_XYLFA_1 | Q9PGX4 | 5.6 | 59284.76 |
| RF3_SALTI_1 | Q8Z0U8 | 5.54 | 59459.86 |
| RF3_BORPE_1 | Q7W0G3 | 6.14 | 59263.67 |
| RF3_SHIFL_1 | Q83P06 | 5.8 | 59441.95 |
| RF3_SYMTH_1 | Q67MT5 | 6.07 | 59895.34 |
| RF3_PSEAE_1 | Q9HXB0 | 5.6 | 59865.28 |
| RF3_LISMO_1 | Q8Y8C0 | 5.3 | 59399.62 |
| RF3_GLOVI_1 | Q7NGL0 | 6.01 | 59736.38 |
| RF3_YERPE_1 | Q8ZIR0 | 6.05 | 59640.1 |
| RF3_GEOSL_1 | Q74GV6 | 5.69 | 59536.69 |
| RF3_STAA8_1 | Q2FZP4 | 4.87 | 59601.58 |
| RF3_STRR6_1 | Q8DR09 | 5.09 | 58482.45 |
| RF3_XANCP_1 | Q8P6V6 | 5.74 | 58983.3 |
| RF3_HAEIN_1 | P43928 | 5.56 | 59174.48 |
| RF3_SYNY3_1 | P73473 | 5.22 | 61189.44 |
| RF3_DESVM_1 | B8DIL5 | 6.01 | 59250.74 |
| RF3_LACLA_1 | Q9CIK7 | 5.06 | 59502.61 |
| RF3_NEIMB_1 | Q9K0H6 | 5.43 | 59588.12 |
| RF3_CUPNH_1 | Q0JY21 | 5.63 | 59313.62 |
| RF3_CUPTR_1 | B3RDA4 | 5.56 | 59155.4 |
| RF3_CYAP4_1 | B8HY03 | 5.49 | 61174.69 |
| RF3_ACIAD_1 | Q6F823 | 5.72 | 60374.97 |
| RF3_ACTSZ_1 | A6VN03 | 5.6 | 59147.44 |
| RF3_ALISL_1 | B6ENF7 | 5.45 | 58854.04 |
| RF3_ALTMD_1 | B4RU35 | 5.47 | 59532.67 |
| RF3_AROAE_1 | Q5P409 | 5.69 | 61116.94 |
| RF3_BORA1_1 | Q2KTQ5 | 5.88 | 59462.98 |
| RF3_BORPD_1 | A9HW20 | 5.79 | 59392.73 |
| RF3_HYDCU_1 | Q31H08 | 5.66 | 58752.5 |
| RF3_PEDPA_1 | Q03GI2 | 5.06 | 59091.08 |
| RF3_KLEP7_1 | A6THZ0 | 5.6 | 59629.27 |
| RF3_PHOLL_1 | Q7MZN3 | 5.93 | 59861.36 |
| RF3_FRAT1_1 | Q14JV7 | 6.12 | 59382.01 |
| RF3_LACP3_1 | Q037T6 | 5.96 | 59458.8 |
| RF3_PSEHT_1 | Q3IHI5 | 5.67 | 59061.25 |
| RF3_PSEMY_1 | A4XQE4 | 5.69 | 59755.07 |
| RF3_GEOUR_1 | A5G9T7 | 6.02 | 60013.38 |
| RF3_PSEPG_1 | B0KQD8 | 5.79 | 59685.07 |
| RF3_LEGPC_1 | A5IG41 | 5.97 | 59403.06 |
| RF3_PSESM_1 | Q87WH1 | 5.58 | 59622.87 |
| RF3_LISIN_1 | Q92D33 | 5.3 | 59461.71 |
| RF3_PSYCK_1 | Q1QDP8 | 5.71 | 59900.77 |
| RF3_MACCJ_1 | B9EAS8 | 5.15 | 59520.92 |
| RF3_NEIG1_1 | Q5FA25 | 5.42 | 59506 |
| RF3_SALG2_1 | B5R9U3 | 5.54 | 59575.09 |
| RF3_SALPB_1 | A9N7C9 | 5.54 | 59563.04 |
| RF3_NITOC_1 | Q3J9L6 | 6.54 | 60765.53 |
| RF3_SHESM_1 | Q0HLF4 | 5.6 | 59387.87 |
| RF3_SHEB2_1 | B8E6N9 | 5.94 | 59233.78 |
| RF3_SHEDO_1 | Q12QG7 | 5.78 | 59414.14 |
| RF3_SHIB3_1 | B2TZQ6 | 5.65 | 59590.08 |
| RF3_SHEHH_1 | B0TQ95 | 5.57 | 59319.87 |
| RF3_SHELP_1 | A3QGT8 | 5.69 | 59312.75 |
| RF3_SHEPA_1 | A8H733 | 5.58 | 59351.9 |
| RF3_SHEPC_1 | A4Y9B3 | 5.74 | 59270.71 |
| RF3_SHISS_1 | Q3YU19 | 5.65 | 59563.06 |
| RF3_STRU0_1 | B9DUR6 | 5.21 | 58445.5 |
| RF3_SYNE7_1 | Q31KM4 | 5.29 | 63079.64 |
| RF3_SYNP6_1 | Q5N192 | 5.39 | 63100.72 |
| RF3_STACT_1 | B9DQA0 | 4.99 | 59738.88 |
| RF3_TERTT_1 | C5BNW9 | 5.5 | 59055.17 |
| RF3_STAES_1 | Q8CPR1 | 5.01 | 59779.76 |
| RF3_THISH_1 | B8GTY2 | 6.18 | 59413.67 |
| RF3_STAS1_1 | Q49WE9 | 4.94 | 59469.47 |
| RF3_STRA3_1 | Q8E635 | 5.1 | 58301.07 |
| RF3_VIBPA_1 | Q87M18 | 5.4 | 59245.47 |
| RF3_STRMK_1 | B2FR00 | 5.93 | 58939.24 |
| RF3_VIBVU_1 | Q8DBT6 | 5.34 | 59197.25 |
| RF3_STRP1_1 | P66021 | 5.07 | 58271.05 |
| RF3_XANAC_1 | Q8PI56 | 5.88 | 59029.41 |
| RF3_XANOM_1 | Q2P4Q8 | 6.06 | 58948.3 |
| RF3_ACAM1_1 | B0C6Z1 | 5.3 | 61189.54 |
| RF3_DECAR_1 | Q47CH1 | 5.4 | 58394.52 |
| RF3_ACTPJ_1 | B0BRI9 | 5.61 | 58998.27 |
| RF3_DESPS_1 | Q6AJD2 | 5.63 | 59639.47 |
| RF3_ALIFM_1 | B5FA93 | 5.28 | 58624.55 |
| RF3_ANAVT_1 | Q3M7Y0 | 5.43 | 61191.75 |
| RF3_BUCAI_1 | P57608 | 9.44 | 60359.1 |
| RF3_HAEDU_1 | Q7VNX4 | 5.7 | 59218.84 |
| RF3_CITK8_1 | A8ALY7 | 5.59 | 59743.41 |
| RF3_HAES1_1 | Q0I2G9 | 5.97 | 59354.82 |
| RF3_HAHCH_1 | Q2S9X0 | 5.79 | 59080.53 |
| RF3_HALHL_1 | A1WSZ8 | 5.31 | 58434.25 |
| RF3_PECAS_1 | Q6D9Z5 | 5.65 | 59684.18 |
| RF3_EDWI9_1 | C5BHI4 | 5.59 | 59638.18 |
| RF3_ENT38_1 | A4W691 | 5.71 | 59931.75 |
| RF3_ENTFA_1 | Q837X4 | 4.94 | 59647.63 |
| RF3_ERWT9_1 | B2VH43 | 5.88 | 59567.06 |
| RF3_FRAP2_1 | B0TWY6 | 5.76 | 59183.8 |
| RF3_LACGA_1 | Q041Z5 | 5.96 | 58856.09 |
| RF3_PSEE4_1 | Q1IEF7 | 5.85 | 59744.14 |
| RF3_GEODF_1 | B9M7Q2 | 5.78 | 60070.26 |
| RF3_GEOMG_1 | Q39Z86 | 5.91 | 59516.87 |
| RF3_LACRD_1 | A5VL77 | 5.16 | 59477.37 |
| RF3_DESAA_1 | B8FGS8 | 5.48 | 59301.83 |
| RF3_ALCBS_1 | Q0VRV7 | 5.43 | 58773.7 |
| RF3_DICNV_1 | A5EVN8 | 5.81 | 59959.53 |
| RF3_CHRVO_1 | Q7NVF7 | 5.26 | 59905.52 |
| RF3_HISS2_1 | B0USE8 | 5.97 | 59328.74 |
| RF3_PECCP_1 | C6DJU6 | 5.72 | 59726.26 |
| RF3_LACAC_1 | Q5FLA9 | 5.37 | 59365.45 |
| RF3_PROMH_1 | B4EWB0 | 5.64 | 59827.28 |
| RF3_LACCB_1 | B3WFB1 | 5.96 | 59492.81 |
| RF3_PSEA6_1 | Q15W54 | 5.68 | 59408.72 |
| RF3_LACDB_1 | Q04BM6 | 5.13 | 58793.65 |
| RF3_PSEU5_1 | A4VI89 | 5.77 | 59891.61 |
| RF3_PSYA2_1 | Q4FUQ9 | 5.67 | 59889.68 |
| RF3_PSYWF_1 | A5WH45 | 5.63 | 59809.49 |
| RF3_RALSO_1 | Q8XPH8 | 5.63 | 59276.53 |
| RF3_SACD2_1 | Q21HQ0 | 5.89 | 58911.24 |
| RF3_MARMS_1 | A6VU13 | 5.46 | 60096.38 |
| RF3_SALAR_1 | A9MRB6 | 5.6 | 59622.1 |
| RF3_METFK_1 | Q1H0I2 | 5.41 | 58754.85 |
| RF3_SALDC_1 | B5FTB7 | 5.54 | 59563.04 |
| RF3_SALHS_1 | B4TGZ2 | 5.54 | 59563.04 |
| RF3_NITEC_1 | Q0ADD1 | 6.48 | 59643.31 |
| RF3_NOSP7_1 | B2J573 | 5.51 | 61388.08 |
| RF3_NOSS1_1 | Q8YP23 | 5.43 | 61270.8 |
| RF3_SHESH_1 | A8FYR3 | 5.57 | 59147.69 |
| RF3_SERP5_1 | A8G9G8 | 5.74 | 59621.16 |
| RF3_SHEWM_1 | B1KRQ3 | 5.38 | 59307.7 |
| RF3_SHEFN_1 | Q086G5 | 5.9 | 59228.77 |
| RF3_SHIDS_1 | Q327M2 | 5.65 | 59574.08 |
| RF3_STRS2_1 | A4W2D1 | 5.11 | 58233.09 |
| RF3_SODGM_1 | Q2NW08 | 5.73 | 59516.04 |
| RF3_STRT2_1 | Q5M364 | 5.02 | 58443.24 |
| RF3_SYNAS_1 | Q2LWC5 | 5.82 | 59990.49 |
| RF3_SYNFM_1 | A0LLL8 | 6.21 | 59591.85 |
| RF3_THIDA_1 | Q3SK69 | 5.93 | 60102.67 |
| RF3_STAHJ_1 | Q4L527 | 4.94 | 59768.68 |
| RF3_TRIEI_1 | Q110K2 | 5.74 | 61416.11 |
| RF3_VIBCB_1 | A7MUW8 | 5.34 | 59206.4 |
| RF3_STRGC_1 | A8AYN4 | 5.08 | 58433.29 |
| RF3_VIBTL_1 | B7VJG2 | 5.3 | 58869.8 |
| RF3_STRMU_1 | Q8DV91 | 5.09 | 58564.45 |
| RF3_YERE8_1 | A1JJ92 | 5.86 | 59714.16 |
| RF3_YERP3_1 | A7FMI1 | 6.02 | 59617.06 |
| RF3_CROS8_1 | A7MGA3 | 5.46 | 59634.09 |
| RF3_DESAG_1 | Q30V54 | 6.06 | 59510.99 |
| RF3_AZOVD_1 | C1DQ00 | 5.65 | 59686.31 |
| RF3_BORBR_1 | Q7WDS1 | 6.14 | 59263.67 |
| RF3_BORPA_1 | Q7W2S3 | 6.14 | 59293.7 |
| RF3_COLP3_1 | Q487B0 | 5.76 | 59375.31 |
| RF3_PASMU_1 | P57879 | 5.57 | 59516.7 |
| RF3_IDILO_1 | Q5QXU1 | 5.5 | 59629.83 |
| RF3_PELCD_1 | Q3A709 | 5.58 | 60167.54 |
| RF3_PELPD_1 | A1AME1 | 5.95 | 59588.92 |
| RF3_LACBA_1 | Q03Q83 | 5.17 | 59247.21 |
| RF3_PSE14_1 | Q48DZ2 | 5.76 | 59704.06 |
| RF3_LACH4_1 | A8YU90 | 5.47 | 59271.37 |
| RF3_LACJO_1 | Q74IG8 | 5.55 | 58991.08 |
| RF3_PSEF5_1 | Q4KIC9 | 5.82 | 59589.99 |
| RF3_LACPL_1 | Q88XF3 | 5.27 | 59561.61 |
| RF3_LACS1_1 | Q1WUZ8 | 5.44 | 59551.89 |
| RF3_LACSS_1 | Q38VL2 | 5.18 | 59228.38 |
| RF3_PSYIN_1 | A1ST34 | 5.98 | 59481.49 |
| RF3_LISW6_1 | A0AHA7 | 5.21 | 59543.75 |
| RF3_MANSM_1 | Q65SF0 | 5.52 | 59558.92 |
| RF3_SALA4_1 | B5F516 | 5.54 | 59563.04 |
| RF3_METCA_1 | Q606M6 | 5.82 | 59347.57 |
| RF3_SALCH_1 | Q57G48 | 5.54 | 59563.04 |
| RF3_MICAN_1 | B0JIY7 | 5.34 | 61499.08 |
| RF3_SALEP_1 | B5R2J0 | 5.54 | 59563.04 |
| RF3_SALNS_1 | B4T4G3 | 5.54 | 59563.04 |
| RF3_NITEU_1 | Q82S73 | 6.27 | 59730.41 |
| RF3_SALRD_1 | Q2S204 | 4.74 | 60328.67 |
| RF3_SALSV_1 | B4TU33 | 5.54 | 59563.04 |
| RF3_STRSV_1 | A3CLS9 | 5.06 | 58555.46 |
| RRF | Accession number | pI | MW |
| RRF_ECOLI_1 | P0A805 | 6.44 | 20638.57 |
| RRF_BACSU_1 | P81101 | 5.52 | 20635.6 |
| RRF_MYCTU_1 | P9WGY1 | 5.71 | 20827.64 |
| RRF_PSEAE_1 | O82853 | 5.85 | 20485.41 |
| RRF_VIBPA_1 | Q8GRF5 | 6.04 | 20602.75 |
| RRF_STRP6_1 | Q5XDH3 | 5.68 | 20572.45 |
| RRF_RHOBA_1 | Q7UTH0 | 5.09 | 20612.35 |
| RRF_BRADU_1 | Q89KP6 | 7.04 | 20961.1 |
| RRF_SALTY_1 | P66738 | 7.76 | 20555.54 |
| RRF_CHLTR_1 | O84684 | 8.59 | 20082.15 |
| RRF_AQUAE_1 | O66928 | 5.94 | 21493.86 |
| RRF_THEMA_1 | Q9X1B9 | 5.44 | 21513 |
| RRF_BACTN_1 | Q8A5J0 | 6.2 | 20723.93 |
| RRF_STRCO_1 | O86770 | 5.22 | 20786.63 |
| RRF_CHLAA_1 | A9WAG2 | 6.19 | 21147.27 |
| RRF_DEIRA_1 | Q9RU82 | 6 | 20620.54 |
| RRF_SHEON_1 | Q8EGH2 | 6.76 | 20586.68 |
| RRF_LISMO_1 | Q8Y7G7 | 5.25 | 20755.67 |
| RRF_YERPE_1 | Q8ZH63 | 6.01 | 20709.68 |
| RRF_GEOSL_1 | P61304 | 8.94 | 21116.42 |
| RRF_GLOVI_1 | Q7NNX0 | 7.88 | 20536.41 |
| RRF_STRR6_1 | Q8DQ49 | 5.76 | 20642.57 |
| RRF_COXBU_1 | Q83BV4 | 6.36 | 20945.15 |
| RRF_STAA8_1 | Q2FZ21 | 5.04 | 20352.98 |
| RRF_ACTSZ_1 | A6VM32 | 6.65 | 20707.71 |
| RRF_AKKM8_1 | B2UKL9 | 6.21 | 20903.17 |
| RRF_ALIFM_1 | B5F9X3 | 5.69 | 20544.67 |
| RRF_ALTMD_1 | B4RVI6 | 5.07 | 20613.6 |
| RRF_ANASK_1 | B4UMC6 | 5.69 | 21065.27 |
| RRF_AYWBP_1 | Q2NIS5 | 9.5 | 20811.24 |
| RRF_BACAC_1 | C3L7A3 | 5.79 | 20619.55 |
| RRF_BACC0_1 | B7JJA2 | 5.79 | 20619.55 |
| RRF_BORBR_1 | Q7WJ91 | 6.78 | 20708.73 |
| RRF_BORBZ_1 | B7J154 | 8.94 | 21349.62 |
| RRF_BRUA1_1 | B2S608 | 5.98 | 20663.72 |
| RRF_BRUSI_1 | B0CGV6 | 5.98 | 20663.72 |
| RRF_CAMJ8_1 | A8FK24 | 7.79 | 20727.55 |
| RRF_CARHZ_1 | Q3AB80 | 7.84 | 20889.27 |
| RRF_CHLCV_1 | Q824U6 | 7.75 | 20143.01 |
| RRF_CHLL2_1 | B3EFU4 | 9.11 | 21246.69 |
| RRF_CLAM3_1 | A5CQS7 | 5.33 | 20581.37 |
| RRF_CLOB8_1 | A6LSP3 | 8.78 | 20652.12 |
| RRF_CYTH3_1 | Q11XK1 | 5.46 | 20793.73 |
| RRF_DEIDV_1 | C1CVP1 | 6.2 | 20371.27 |
| RRF_DESHD_1 | B8FRG2 | 7.83 | 21148.38 |
| RRF_NEIMA_1 | P66736 | 6.22 | 20730.86 |
| RRF_PARXL_1 | Q13XB9 | 7.8 | 20947.05 |
| RRF_ACIB3_1 | B7H1U3 | 5.66 | 20641.66 |
| RRF_AERHH_1 | A0KHG6 | 6.01 | 20723.75 |
| RRF_AGRVS_1 | B9JX30 | 6.77 | 20565.56 |
| RRF_ALCBS_1 | Q0VQF4 | 5.02 | 20984.92 |
| RRF_ALKMQ_1 | A6TRM0 | 6.04 | 21141.41 |
| RRF_AMOA5_1 | B3ETV4 | 7.76 | 21315.58 |
| RRF_ANAD2_1 | B8J9V9 | 5.69 | 21051.24 |
| RRF_AROAE_1 | Q5NZH2 | 7.93 | 21128.32 |
| RRF_ARTS2_1 | A0JUP4 | 5.2 | 20491.28 |
| RRF_AZOSB_1 | A1K6R8 | 7.8 | 21093.33 |
| RRF_BACFR_1 | Q64YJ0 | 5.86 | 20764.06 |
| RRF_BACHK_1 | Q6HEZ1 | 5.79 | 20619.55 |
| RRF_BACWK_1 | A9VT62 | 5.78 | 20651.55 |
| RRF_BORPD_1 | A9INV3 | 6.78 | 20848.85 |
| RRF_BUCAP_1 | Q8K9S8 | 9.37 | 21509.86 |
| RRF_BURCA_1 | Q1BHH9 | 7.88 | 20856.96 |
| RRF_MYCMM_1 | B2HJP0 | 5.75 | 20952.76 |
| RRF_MYCMS_1 | P61306 | 6.85 | 20812.28 |
| RRF_NEOSM_1 | Q2GCI7 | 9.32 | 20635.08 |
| RRF_NITMU_1 | Q2YBA4 | 9 | 20798.07 |
| RRF_NOVAD_1 | Q2G8K7 | 7.86 | 20386.27 |
| RRF_PHYAS_1 | B1VA77 | 9.54 | 21085.74 |
| RRF_PROA2_1 | B4S4H8 | 9.24 | 21054.2 |
| RRF_PSECP_1 | B8HFN8 | 5.13 | 20676.46 |
| RRF_PSEF5_1 | Q4KHH3 | 7.87 | 20347.39 |
| RRF_PSEPK_1 | Q88MH7 | 7.88 | 20152.07 |
| RRF_PSYCK_1 | Q1QA13 | 5.27 | 20858.79 |
| RRF_RALSO_1 | Q8XZI8 | 7.86 | 20844.93 |
| RRF_ACIAD_1 | Q6FCH2 | 5.65 | 20660.58 |
| RRF_BACLD_1 | Q65JJ6 | 5.33 | 20912.83 |
| RRF_BACP2_1 | A8FDB6 | 5.35 | 20841.78 |
| RRF_BARHE_1 | Q6G5C6 | 6.18 | 20665.73 |
| RRF_BEII9_1 | B2ID97 | 7.95 | 20641.76 |
| RRF_BLOFL_1 | Q7VRE3 | 9.2 | 22003.77 |
| RRF_BORA1_1 | Q2L159 | 6.86 | 20739.65 |
| RRF_BRUMB_1 | C0RJC7 | 6.34 | 20722.79 |
| RRF_BURVG_1 | A4JF72 | 7.88 | 20828.91 |
| RRF_CAMHC_1 | A7I0C2 | 7.68 | 20631.5 |
| RRF_CHLAD_1 | B8G461 | 6.18 | 21159.24 |
| RRF_CHLMU_1 | P71148 | 8.47 | 20025.99 |
| RRF_CHRSD_1 | Q1R030 | 5.89 | 20837.77 |
| RRF_CLOK1_1 | B9E1J1 | 8.95 | 20856.43 |
| RRF_CLONN_1 | A0Q0R7 | 7.75 | 20775.93 |
| RRF_EHRRG_1 | Q5FG66 | 9.04 | 20748.16 |
| RRF_ENT38_1 | A4W6R7 | 6.44 | 20519.48 |
| RRF_ENTFA_1 | Q831V2 | 5.21 | 20803.69 |
| RRF_ERYLH_1 | Q2NBU1 | 5.31 | 20521.44 |
| RRF_EXIS2_1 | B1YI73 | 5.44 | 20900.94 |
| RRF_FINM2_1 | B0S187 | 5.42 | 21125.29 |
| RRF_MYCGE_1 | P47673 | 9.1 | 21518.88 |
| RRF_BACHD_1 | Q9KA66 | 5.53 | 20855.7 |
| RRF_BACV8_1 | A6L1H8 | 6.21 | 20737.01 |
| RRF_BDEBA_1 | P61302 | 9.08 | 20732.04 |
| RRF_BIFLD_1 | B3DRT4 | 5.77 | 20128.9 |
| RRF_BRASB_1 | A5EK52 | 6.99 | 20865.88 |
| RRF_BREBN_1 | C0ZF63 | 6.65 | 20866.94 |
| RRF_BURM1_1 | A9AIL7 | 7.88 | 20806.95 |
| RRF_CAUSK_1 | B0SZ18 | 8.68 | 20870.04 |
| RRF_CAUVN_1 | B8GWS0 | 8.75 | 20935.94 |
| RRF_CHESB_1 | Q11IJ5 | 5.82 | 20746.59 |
| RRF_CHLPB_1 | B3EMY2 | 8.79 | 20982.23 |
| RRF_CLOBB_1 | B2TJ43 | 8.75 | 20770.26 |
| RRF_CLOTE_1 | Q895K9 | 5.78 | 20703.06 |
| RRF_CLOTH_1 | A3DE56 | 6.49 | 20941.23 |
| RRF_CORDI_1 | P61303 | 4.99 | 20822.8 |
| RRF_COREF_1 | Q8FP75 | 5 | 20904.89 |
| RRF_CORGL_1 | Q8NP03 | 5.07 | 20748.81 |
| RRF_CUPNJ_1 | Q470E0 | 8.51 | 20835.09 |
| RRF_CYAP4_1 | B8HL79 | 6.63 | 20140.83 |
| RRF_DEHM1_1 | Q3Z9H9 | 7.78 | 20868.13 |
| RRF_DESAA_1 | B8FGE3 | 5.49 | 20815.09 |
| RRF_DESMR_1 | C4XMX9 | 9.33 | 20866.96 |
| RRF_DESVH_1 | Q72DQ9 | 6.47 | 21042.1 |
| RRF_EHRCJ_1 | Q3YR65 | 8.81 | 20853.1 |
| RRF_FLAPJ_1 | A6GWS4 | 5.06 | 20771.9 |
| RRF_FRASN_1 | A8L6D6 | 5.37 | 20744.46 |
| RRF_MYCPA_1 | P61307 | 5.41 | 20651.41 |
| RRF_MYCPE_1 | Q8EUH0 | 5.93 | 21631.83 |
| RRF_NITEU_1 | Q82TZ8 | 8.69 | 20991.39 |
| RRF_NITWN_1 | Q3SRH5 | 6.99 | 20713.72 |
| RRF_ONYPE_1 | P61308 | 9.5 | 20799.28 |
| RRF_PARPJ_1 | B2T5J1 | 7.79 | 20950.05 |
| RRF_ALKEH_1 | Q0A7I3 | 5.49 | 21087.07 |
| RRF_ALKOO_1 | A8MHH2 | 7.8 | 21070.33 |
| RRF_BACCN_1 | A7GRF4 | 5.5 | 20652.6 |
| RRF_BARBK_1 | Q8RT64 | 6 | 20838.15 |
| RRF_BART1_1 | A9ISK9 | 5.99 | 20723.75 |
| RRF_BIFAA_1 | A1A1I2 | 6.45 | 20153.95 |
| RRF_BLOPB_1 | Q493C9 | 8.58 | 21831.34 |
| RRF_BORBP_1 | Q662P1 | 8.94 | 21363.65 |
| RRF_BORHD_1 | B2RZI6 | 7.77 | 21334.6 |
| RRF_BURM9_1 | A2SB75 | 7.88 | 20899 |
| RRF_NOCFA_1 | Q5YS64 | 5.2 | 20507.35 |
| RRF_NOCSJ_1 | A1SLQ7 | 5.81 | 20878.76 |
| RRF_ACIET_1 | B9MGM0 | 8.71 | 20608.77 |
| RRF_ACTPJ_1 | B0BUC8 | 6.03 | 20676.64 |
| RRF_ACHLI_1 | A9NHC3 | 5.08 | 20724.68 |
| RRF_AERS4_1 | A4SQH9 | 6.01 | 20667.64 |
| RRF_BARQU_1 | Q6FZN3 | 5.9 | 20711.75 |
| RRF_BORDL_1 | B5RLJ5 | 8.94 | 21315.61 |
| RRF_MYCLB_1 | B8ZRV0 | 5.52 | 20891.84 |
| RRF_NOSS1_1 | Q8YXK4 | 7.83 | 20237.03 |
| RRF_PASMU_1 | P57984 | 6.03 | 20850.88 |
| RRF_PEDPA_1 | Q03FT3 | 5.13 | 20630.39 |
| RRF_PORG3_1 | B2RLV6 | 5.6 | 20797.98 |
| RRF_PROM5_1 | A2BVI3 | 5.8 | 20612.46 |
| RRF_PSEE4_1 | Q1I629 | 7.88 | 20146.05 |
| RRF_PSEMY_1 | A4XWT8 | 6.77 | 20200.18 |
| RRF_AGARV_1 | C4ZA73 | 5.47 | 20704.67 |
| RRF_AGRRK_1 | B9JEX3 | 6.21 | 20777.77 |
| RRF_ARCB4_1 | A8EX12 | 8.57 | 20811.79 |
| RRF_AZOC5_1 | A8I468 | 6.86 | 20585.56 |
| RRF_AZOVD_1 | C1DSU3 | 6.21 | 20392.37 |
| RRF_BACSK_1 | Q5WFT1 | 6.46 | 20711.78 |
| RRF_BRUO2_1 | A5VQT0 | 5.98 | 20663.72 |
| RRF_BURPS_1 | Q63T15 | 7.88 | 20899 |
| RRF_CALS8_1 | A4XLZ9 | 5.68 | 21302.84 |
| RRF_CHLFF_1 | Q252Q4 | 7.75 | 20155.13 |
| RRF_CLOAB_1 | Q97I63 | 7.76 | 20830.16 |
| RRF_DESAH_1 | C0QB20 | 6.03 | 21144.34 |
| RRF_DESOH_1 | A8ZTM3 | 5.31 | 21038.21 |
| RRF_DESPS_1 | Q6AP38 | 5.74 | 20820.12 |
| RRF_ERWT9_1 | B2VE11 | 6 | 20721.64 |
| RRF_ESCF3_1 | B7LWB1 | 6.44 | 20638.57 |
| RRF_FLAJ1_1 | A5FMF6 | 4.9 | 20698.66 |
| RRF_FRACC_1 | Q2J711 | 5.38 | 20830.55 |
| RRF_FRATF_1 | A7N9R7 | 5.58 | 20552.63 |
| RRF_MYCGI_1 | A4TC77 | 5.38 | 20859.47 |
| RRF_MYCMO_1 | Q6KIN5 | 6.48 | 21410.86 |
| RRF_MYCPN_1 | P75161 | 7.82 | 21667.03 |
| RRF_MYCSJ_1 | A3PXZ5 | 5.4 | 20891.51 |
| RRF_MYCUA_1 | A0PQ87 | 5.75 | 20938.73 |
| RRF_MYCVP_1 | A1T785 | 5.39 | 20839.53 |
| RRF_NEIG1_1 | Q5F5X3 | 6.22 | 20642.76 |
| RRF_NOSP7_1 | B2IVW4 | 8.73 | 20301.99 |
| RRF_OLICO_1 | B6JH69 | 6.86 | 20399.44 |
| RRF_PARL1_1 | A7HY16 | 5.98 | 20922.99 |
| RRF_PELTS_1 | A5D2T8 | 9 | 20968.27 |
| RRF_PETMO_1 | A9BJD6 | 5.55 | 21774.88 |
| RRF_POLNA_1 | A1VN43 | 7.92 | 20853.85 |
| RRF_PROMH_1 | B4F2D0 | 5.48 | 20681.75 |
| RRF_BACVZ_1 | A7Z4S3 | 5.33 | 20654.52 |
| RRF_BRUC2_1 | A9M5H1 | 5.98 | 20663.72 |
| RRF_BURA4_1 | B1YS71 | 7.88 | 20798.88 |
| RRF_BURTA_1 | Q2SWZ5 | 7.88 | 20809.91 |
| RRF_CALBD_1 | B9MKP8 | 5.68 | 21308.8 |
| RRF_CAMC5_1 | A7H147 | 6.75 | 20373.36 |
| RRF_CAMFF_1 | A0RRR3 | 6.63 | 20709.56 |
| RRF_CAMLR_1 | B9KE24 | 7.78 | 20752.58 |
| RRF_CHLCH_1 | Q3ATS6 | 9.14 | 21096.55 |
| RRF_CHLL7_1 | Q3B5Z5 | 9 | 21233.34 |
| RRF_CHLPM_1 | A4SD71 | 8.94 | 21211.54 |
| RRF_CHLPN_1 | Q9Z7K6 | 8.44 | 20169.24 |
| RRF_CHRVO_1 | Q7NVZ1 | 6.77 | 20916.1 |
| RRF_CLOPS_1 | Q0SSC3 | 8.98 | 20925.41 |
| RRF_CORA7_1 | C3PH48 | 4.94 | 20981.99 |
| RRF_CUTAK_1 | Q6A7K0 | 5.5 | 20929.87 |
| RRF_DEIGD_1 | Q1IZI9 | 6.21 | 20581.55 |
| RRF_EHRCR_1 | Q2GHJ5 | 9.05 | 20709.99 |
| RRF_MYCCT_1 | Q2SSA4 | 7.85 | 20840.33 |
| RRF_MYXXD_1 | Q1D1I2 | 9.17 | 20913.16 |
| RRF_PAEAT_1 | A1R4W2 | 5.21 | 20524.3 |
| RRF_PSEU2_1 | Q4ZWS5 | 6.77 | 20483.46 |
| RRF_PSYA2_1 | Q4FRH6 | 5.25 | 20873.72 |
| RRF_ACIC1_1 | A0LV51 | 5.47 | 20998.79 |
| RRF_ACISJ_1 | A1W915 | 8.71 | 20608.77 |
| RRF_BAUCH_1 | Q1LSV4 | 9.44 | 21573 |
| RRF_BEUC1_1 | C5BWT8 | 4.94 | 20550.31 |
| RRF_BIFA0_1 | B8DUA6 | 6.66 | 20218.04 |
| RRF_BORAP_1 | Q0SP43 | 8.94 | 21349.62 |
| RRF_CALS4_1 | Q8RA24 | 7.81 | 21229.58 |
| RRF_COPPD_1 | B5Y8J2 | 6.79 | 21836.94 |
| RRF_CORJK_1 | Q4JV21 | 4.94 | 20796.62 |
| RRF_CORK4_1 | C4LJA6 | 4.95 | 21069.95 |
| RRF_CORU7_1 | B1VG92 | 4.82 | 21157 |
| RRF_CUPMC_1 | Q1LNF5 | 8.67 | 20814.98 |
| RRF_CUPTR_1 | B3R2B4 | 8.53 | 20856.07 |
| RRF_EDWI9_1 | C5BHB9 | 6.65 | 20764.81 |
| RRF_FRAP2_1 | B0U0Z8 | 5.57 | 20523.59 |
| RRF_MYCGA_1 | Q7NC19 | 9.15 | 21144.5 |
| RRF_MYCPU_1 | Q98QZ1 | 6.78 | 21820.08 |
| RRF_NATTJ_1 | B2A383 | 5.21 | 20824.74 |
| RRF_PARD8_1 | A6LHB5 | 6.23 | 21138.53 |
| RRF_PELCD_1 | Q3A398 | 7.78 | 20893.12 |
| RRF_PHOPR_1 | Q6LN27 | 5.67 | 20958.15 |
| RRF_NITOC_1 | Q3JCX2 | 5.5 | 21418.51 |
| RRF_NITSB_1 | A6Q638 | 5.69 | 21208.23 |
| RRF_OCHA4_1 | A6X0J4 | 5.98 | 20675.72 |
| RRF_PECCP_1 | C6DAI6 | 6.45 | 20653.57 |
| RRF_PELPB_1 | B4SER6 | 9.13 | 21395.8 |
| RRF_PHOLL_1 | Q7N8P4 | 5.8 | 20874.91 |
| RRF_POLAQ_1 | A4SYU8 | 6.87 | 20832.8 |
| RRF_PSE14_1 | Q48F62 | 6.77 | 20483.46 |
| RRF_PARP8_1 | B2JIC3 | 7.87 | 20840.9 |
| RRF_PARUW_1 | Q6M9Z9 | 9.11 | 20638.13 |
| RRF_PELPD_1 | A1AQN5 | 9.11 | 21201.61 |
| RRF_POLSJ_1 | Q12A34 | 7.89 | 20785.82 |
| RRF_PSEA6_1 | Q15WG0 | 5.39 | 20649.7 |
| RRF_PSYIN_1 | A1SYW0 | 5.83 | 20703.67 |
| RRF_PSYWF_1 | A5WGF0 | 5.18 | 20853.76 |
| RRF_RALPJ_1 | B2UB09 | 7.84 | 20745.9 |
| RRF_AGRFC_1 | Q8UFM0 | 6.19 | 20644.52 |
| RRF_ALISL_1 | B6EK54 | 6.02 | 20671.86 |
| RRF_NITEC_1 | Q0AEH7 | 8.73 | 20984.27 |
| RRF_OCEIH_1 | Q8EQV0 | 5.09 | 20571.19 |
| RRF_PARZE_1 | Q8L1H5 | 5.03 | 20877.81 |
| RRF_PSELT_1 | A8F863 | 8.61 | 21289.57 |
| RRF_ACICJ_1 | A5G1L3 | 6.64 | 20944.09 |
| RRF_BORPA_1 | Q7WA57 | 6.78 | 20708.73 |
| RRF_BORPE_1 | Q7VYC7 | 6.78 | 20708.73 |
| RRF_BORRA_1 | B5RQU6 | 8.94 | 21315.61 |
| RRF_BORT9_1 | A1QYS0 | 8.58 | 21230.53 |
| RRF_BURL3_1 | Q39F46 | 7.88 | 20823.88 |
| RRF_CAMC1_1 | A7ZG18 | 7.71 | 20532.47 |
| RRF_CHLAB_1 | Q5L766 | 8.87 | 20134.15 |
| RRF_CHLP8_1 | B3QL17 | 8.92 | 21224.46 |
| RRF_CHLTE_1 | Q8KC49 | 8.96 | 21222.51 |
| RRF_COLP3_1 | Q485G7 | 5.23 | 20681.67 |
| RRF_DELAS_1 | A9BMM9 | 7.79 | 20700.75 |
| RRF_DESAG_1 | Q313G7 | 7.8 | 20821.02 |
| RRF_DESRM_1 | A4J5Z0 | 8.98 | 20730.93 |
| RRF_DICNV_1 | A5EV26 | 5.89 | 21159.26 |
| RRF_DINSH_1 | A8LK37 | 5.02 | 21151.06 |
| RRF_FERNB_1 | A7HN62 | 7.78 | 21315.71 |
| RRF_FRAAA_1 | Q0RDQ6 | 5.37 | 20754.48 |
| RRF_ORITB_1 | A5CFN3 | 7.71 | 21382.85 |
| RRF_PARDP_1 | A1B966 | 5.13 | 20891.83 |
| RRF_PHEZH_1 | B4RBZ1 | 7.89 | 21257.35 |
| RRF_SALSV_1 | B4TYD2 | 7.76 | 20555.54 |
| RRF_SALTI_1 | P66739 | 7.76 | 20555.54 |
| RRF_STRGC_1 | A8AY70 | 5.9 | 20652.61 |
| RRF_THEP1_1 | A5IMH5 | 5.89 | 21450.93 |
| RRF_SHEPW_1 | B8CQ82 | 6.77 | 20700.78 |
| RRF_RHOOB_1 | C1B2U5 | 5.2 | 20596.29 |
| RRF_SHIB3_1 | B2U315 | 6.44 | 20638.57 |
| RRF_SHIDS_1 | Q32JT7 | 6.44 | 20638.57 |
| RRF_VIBCB_1 | A7MXZ7 | 5.68 | 20589.67 |
| RRF_VIBCM_1 | C3LQ29 | 6.03 | 20637.74 |
| RRF_VIBVY_1 | Q7MIG3 | 6.03 | 20479.59 |
| RRF_RICAE_1 | C3PMJ3 | 8.65 | 20838.92 |
| RRF_SPHWW_1 | A5V3G5 | 6.77 | 20310.11 |
| RRF_RICFE_1 | Q4UKF3 | 6.93 | 20863.88 |
| RRF_RICRO_1 | B0BWC7 | 7.89 | 20928.01 |
| RRF_SYNAS_1 | Q2LTQ4 | 6.65 | 21185.32 |
| RRF_ROSS1_1 | A5UUU9 | 6.34 | 21064.23 |
| RRF_XYLFA_1 | Q9PEH7 | 6.64 | 20620.57 |
| RRF_STAEQ_1 | Q5HPT2 | 4.99 | 20331.93 |
| RRF_SYNPX_1 | Q7U5F4 | 5.43 | 20478.1 |
| RRF_YERE8_1 | A1JP79 | 6.01 | 20781.79 |
| RRF_THEFY_1 | Q47S50 | 5.22 | 20814.64 |
| RRF_STRM5_1 | B4SQ19 | 8.67 | 20363.19 |
| RRF_RHIL3_1 | Q1MH51 | 6.78 | 20792.83 |
| RRF_STRMU_1 | Q8DSY2 | 5.58 | 20642.55 |
| RRF_SHEDO_1 | Q12NY4 | 6.21 | 20775.86 |
| RRF_THESQ_1 | B1LBS6 | 5.44 | 21513 |
| RRF_SHEHH_1 | B0TP80 | 6.2 | 20625.67 |
| RRF_TOLAT_1 | C4L861 | 5.99 | 20670.73 |
| RRF_SHESW_1 | A1RLM4 | 6.77 | 20659.73 |
| RRF_SHISS_1 | Q3Z5I6 | 6.44 | 20638.57 |
| RRF_RHOS1_1 | A3PJF7 | 5.5 | 20994.03 |
| RRF_WOLWR_1 | C0R2L2 | 7.92 | 20927.06 |
| RRF_RICM5_1 | A8F0R2 | 7.89 | 20926.98 |
| RRF_XANC5_1 | Q3BVK7 | 6.76 | 20352.28 |
| RRF_SULNB_1 | A6Q688 | 5.36 | 20969.79 |
| RRF_SALAI_1 | A8M6B4 | 5.34 | 21063.07 |
| RRF_SALCH_1 | Q57T36 | 7.76 | 20555.54 |
| RRF_ACIAC_1 | A1TN72 | 8.71 | 20569.73 |
| RRF_OENOB_1 | Q04F85 | 5.57 | 20380.3 |
| RRF_PECAS_1 | Q6D8E0 | 6.65 | 20779.69 |
| RRF_PEPD6_1 | Q185S5 | 6.79 | 21108.36 |
| RRF_POLNS_1 | B1XTU6 | 6.86 | 20811.83 |
| RRF_SHEPA_1 | A8H6L3 | 6.2 | 20667.75 |
| RRF_TROWT_1 | Q83G73 | 8.81 | 20208.39 |
| RRF_RHOPB_1 | Q215E6 | 6.54 | 20847.9 |
| RRF_STRS2_1 | A4W2B1 | 6.78 | 20663.71 |
| RRF_SYNFM_1 | A0LJ66 | 5.81 | 21240.48 |
| RRF_RUTMC_1 | A1AVT6 | 8.59 | 21060.19 |
| RRF_STAS1_1 | Q49X44 | 4.93 | 20363.84 |
| RRF_SALNS_1 | B4SV01 | 7.76 | 20555.54 |
| RRF_YERPS_1 | Q667J2 | 6.01 | 20709.68 |
| RRF_SHEAM_1 | A1S4P3 | 6.22 | 20789.84 |
| RRF_SHEB2_1 | B8E7R3 | 7.74 | 20697.78 |
| RRF_RHIEC_1 | Q2K8Y4 | 6.78 | 20735.71 |
| RRF_TREPS_1 | B2S3J4 | 7.8 | 20503.54 |
| RRF_XANOR_1 | Q5H1E4 | 6.76 | 20352.28 |
| RRF_RUBXD_1 | Q1AW65 | 5.45 | 21414.41 |
| RRF_RUEST_1 | Q1GGS0 | 5.04 | 20967 |
| RRF_SACEN_1 | A4FMD4 | 5.21 | 20678.55 |
| RRF_SALPA_1 | Q5PD60 | 6.44 | 20518.54 |
| RRF_STRA1_1 | Q3K011 | 5.45 | 20793.59 |
| RRF_SHEFN_1 | Q085D9 | 7.69 | 20645.71 |
| RRF_THET2_1 | Q72KE0 | 6.99 | 20994.04 |
| RRF_RHOE4_1 | C0ZY18 | 5.31 | 20655.47 |
| RRF_UREP2_1 | B1AJF3 | 7.86 | 21086.39 |
| RRF_UREU1_1 | B5ZC20 | 7.86 | 20942.11 |
| RRF_STRSV_1 | A3CPA2 | 5.9 | 20668.61 |
| RRF_STRT1_1 | Q5M140 | 5.45 | 20591.5 |
| RRF_SPHAL_1 | Q1GRQ3 | 6.86 | 20435.36 |
| RRF_WOLPP_1 | B3CNH2 | 6.61 | 20957.02 |
| RRF_RICBR_1 | Q1RHF1 | 7.87 | 20920.99 |
| RRF_RICPR_1 | Q9ZE08 | 8.61 | 21044.24 |
| RRF_XANP2_1 | A7INR3 | 6.34 | 20774.71 |
| RRF_SALEP_1 | B5R3I5 | 7.76 | 20555.54 |
| RRF_SALHS_1 | B4TK47 | 7.76 | 20555.54 |
| RRF_LACP3_1 | Q038L5 | 5.44 | 20593.32 |
| RRF_FUSNN_1 | Q8R5Z9 | 7.71 | 21437.82 |
| RRF_LACPL_1 | Q88VJ7 | 5.85 | 20593.52 |
| RRF_GEODF_1 | B9M5C3 | 8.65 | 21066.42 |
| RRF_LACRE_1 | Q8VS52 | 5.42 | 20576.29 |
| RRF_GEOLS_1 | B3E719 | 8.58 | 21112.51 |
| RRF_LACS1_1 | Q1WUG4 | 6.03 | 20979.05 |
| RRF_GEOSM_1 | C6E515 | 8.67 | 20939.2 |
| RRF_GEOSW_1 | C5D9B8 | 5.67 | 20902.08 |
| RRF_GEOTN_1 | A4IMC6 | 6.66 | 20823.99 |
| RRF_GEOUR_1 | A5G7W5 | 8.65 | 21077.36 |
| RRF_LEGPH_1 | Q5ZUT1 | 6.35 | 20862.89 |
| RRF_LEIXX_1 | Q6AEV4 | 5.08 | 20418.31 |
| RRF_GLUOX_1 | Q5FPZ4 | 6.43 | 20821.6 |
| RRF_GRABC_1 | Q0BTL9 | 6.21 | 21060.99 |
| RRF_LEPBL_1 | Q053N2 | 8.63 | 20640.87 |
| RRF_LEPBP_1 | B0SM63 | 8.79 | 20696.98 |
| RRF_HAEI8_1 | Q4QM92 | 6.78 | 20742.84 |
| RRF_LEPIC_1 | Q72U11 | 8.63 | 20600.78 |
| RRF_HAEPS_1 | B8F3D4 | 6.66 | 20883.92 |
| RRF_HAES1_1 | Q0I379 | 6.03 | 20874.9 |
| RRF_HAHCH_1 | Q2SBQ0 | 6.65 | 20719.87 |
| RRF_HALHL_1 | A1WX18 | 5.28 | 20988.88 |
| RRF_HALOH_1 | B8CW56 | 5.59 | 21422.53 |
| RRF_HAMD5_1 | C4K5W6 | 9.23 | 21058.14 |
| RRF_HELAH_1 | Q17Z48 | 8.58 | 20943.02 |
| RRF_HELHP_1 | Q7VJ10 | 7.69 | 21212.29 |
| RRF_MACCJ_1 | B9EBD9 | 5.16 | 20601.48 |
| RRF_MAGMM_1 | A0L8Q8 | 6.19 | 20737 |
| RRF_HELPG_1 | B5Z8Q1 | 8.58 | 20889.91 |
| RRF_MARMM_1 | Q0APW2 | 5.29 | 20869.78 |
| RRF_MESFL_1 | Q6F0R0 | 5.4 | 20581.8 |
| RRF_METC4_1 | B7KZH3 | 5.84 | 20855.85 |
| RRF_METCA_1 | Q60BA7 | 7.96 | 21142.2 |
| RRF_METFK_1 | Q1H142 | 6.77 | 20812.84 |
| RRF_METI4_1 | B3DXF7 | 5.46 | 21286.75 |
| RRF_METNO_1 | B8INK3 | 6.93 | 20917.12 |
| RRF_IDILO_1 | Q5QXS3 | 5.2 | 20745.61 |
| RRF_METPP_1 | A2SH95 | 8.8 | 20915.87 |
| RRF_JANSC_1 | Q28PI7 | 4.72 | 20691.5 |
| RRF_METS4_1 | B0UPZ4 | 6.93 | 20904.04 |
| RRF_KLEP3_1 | B5Y1J9 | 6.44 | 20630.57 |
| RRF_MICLC_1 | C5C9Q7 | 5.33 | 20873.59 |
| RRF_KOSOT_1 | C5CF34 | 6.8 | 21793.25 |
| RRF_MYCA1_1 | A0QJ18 | 5.41 | 20651.41 |
| RRF_LACDA_1 | Q1G9N9 | 6.03 | 20739.82 |
| RRF_MYCBP_1 | A1KMM6 | 5.71 | 20843.68 |
| RRF_THEAB_1 | B7IDI0 | 5.77 | 21524.85 |
| RRF_STRE4_1 | C0MBT1 | 5.87 | 20554.44 |
| RRF_THENN_1 | B9K8T5 | 5.9 | 21468.97 |
| RRF_RHILO_1 | Q98MB8 | 6.11 | 20629.38 |
| RRF_THERP_1 | B9L003 | 5.58 | 21240.26 |
| RRF_SHEPC_1 | A4Y546 | 6.77 | 20659.73 |
| RRF_UNCTG_1 | B1H056 | 9.08 | 21038.42 |
| RRF_SHEWM_1 | B1KNU0 | 5.54 | 20573.59 |
| RRF_VEREI_1 | A1WHU5 | 9.3 | 20700.91 |
| RRF_SHIFL_1 | P0A808 | 6.44 | 20638.57 |
| RRF_SINFN_1 | C3MBQ5 | 6.35 | 20842.91 |
| RRF_SODGM_1 | Q2NRL0 | 6.65 | 20761.89 |
| RRF_STACT_1 | B9DPH1 | 5.08 | 20454.17 |
| RRF_RUEPO_1 | Q5LSV2 | 5.03 | 21017.04 |
| RRF_STAHJ_1 | Q4L5W1 | 4.9 | 20368.9 |
| RRF_LACP7_1 | A9KMX5 | 7.78 | 20909.1 |
| RRF_GEOBB_1 | B5EHV8 | 7.86 | 21019.2 |
| RRF_GEOKA_1 | Q5L0J9 | 6.04 | 20801.92 |
| RRF_GEOMG_1 | Q39W85 | 8.92 | 21021.36 |
| RRF_LACSS_1 | Q38W67 | 5.2 | 20453.28 |
| RRF_LARHH_1 | C1D9F3 | 6.35 | 20960.01 |
| RRF_LAWIP_1 | Q1MRD7 | 7.72 | 21613.85 |
| RRF_GLUDA_1 | A9HKW6 | 6.44 | 20797.63 |
| RRF_GRAFK_1 | A0LXJ3 | 4.98 | 20550.48 |
| RRF_HAEDU_1 | Q7VL83 | 6.66 | 20766.78 |
| RRF_LEPCP_1 | B1XXJ2 | 8.73 | 20928.03 |
| RRF_LEUCK_1 | B1MZ62 | 5.14 | 20306.2 |
| RRF_LEUMM_1 | Q03WX8 | 5.18 | 20308.18 |
| RRF_LISIN_1 | Q92C40 | 5.24 | 20755.67 |
| RRF_LISW6_1 | A0AIB5 | 5.24 | 20794.7 |
| RRF_HELMI_1 | B0THE0 | 6.87 | 21267.47 |
| RRF_MAGSA_1 | Q2W4C6 | 8.56 | 21341.57 |
| RRF_MANSM_1 | Q65R74 | 6.66 | 20793.85 |
| RRF_MARMS_1 | A6VUS5 | 5.67 | 20824.59 |
| RRF_HERAR_1 | A4G4S4 | 7.76 | 20937.23 |
| RRF_HISS2_1 | B0UUI5 | 6.03 | 20874.9 |
| RRF_HYDCU_1 | Q31G46 | 4.83 | 20990.68 |
| RRF_HYDS0_1 | B4U9X4 | 5.58 | 20820.84 |
| RRF_HYPNA_1 | Q0C1B8 | 5.98 | 20178.99 |
| RRF_METPB_1 | B1ZLC8 | 5.59 | 20783.78 |
| RRF_JANMA_1 | A6SZP8 | 7.76 | 20919.19 |
| RRF_METRJ_1 | B1LTQ4 | 6.34 | 20998.05 |
| RRF_KINRD_1 | A6W7W7 | 5.32 | 20712.64 |
| RRF_METSB_1 | B8EMN5 | 6.93 | 20774.86 |
| RRF_MICAN_1 | B0JKT7 | 6.64 | 20342.22 |
| RRF_KOCRD_1 | B2GKT3 | 5.72 | 20329.93 |
| RRF_MOOTA_1 | Q2RJP0 | 8.7 | 20905.14 |
| RRF_LACCB_1 | B3WES5 | 5.44 | 20593.32 |
| RRF_MYCA9_1 | B1MDE3 | 5.19 | 20714.46 |
| RRF_LACGA_1 | Q044C7 | 5.62 | 20358.22 |
| RRF_LACH4_1 | A8YVR6 | 5.64 | 20770.57 |
| RRF_LACJO_1 | P61305 | 5.61 | 20358.22 |
| RRF_RENSM_1 | A9WL12 | 5.04 | 20584.31 |
| RRF_SALRD_1 | Q2S6J3 | 4.28 | 21439.76 |
| RRF_SHELP_1 | A3QGA0 | 5.85 | 20651.7 |
| RRF_THIDA_1 | Q3SKN6 | 6.86 | 21011.05 |
| RRF_TREDE_1 | P61310 | 8.41 | 20879.09 |
| RRF_RHOCS_1 | B6ISU8 | 6.65 | 20624.58 |
| RRF_RHOFT_1 | Q21WY6 | 7.82 | 20674.84 |
| RRF_SORC5_1 | A9GGK7 | 5.7 | 20716.9 |
| RRF_WIGBR_1 | Q8D2G5 | 9.78 | 21650.31 |
| RRF_RICCK_1 | A8EXM3 | 8.65 | 20995.17 |
| RRF_SULDN_1 | Q30NU0 | 7.74 | 20720.72 |
| RRF_SYNE7_1 | Q31QY0 | 5.69 | 20169.88 |
| RRF_MYCS2_1 | A0QVE0 | 5.31 | 20824.51 |
| RRF_SYMTH_1 | Q67PB4 | 8.7 | 21214.46 |
| RRF_RICTY_1 | Q68XL4 | 8.92 | 21001.18 |
| RRF_SACD2_1 | Q21HH5 | 5.59 | 20667.63 |
| RRF_SYNWW_1 | Q0AYK1 | 7.86 | 21262.64 |
| RRF_SALAR_1 | A9MPI9 | 7.76 | 20555.54 |
| RRF_SALDC_1 | B5FJ19 | 7.76 | 20555.54 |
| RRF_SALG2_1 | B5RHF7 | 7.76 | 20555.54 |
| RRF_SALTO_1 | A4X4J5 | 5.33 | 21009.96 |
| RRF_THEM4_1 | A6LMH6 | 7.78 | 21578.94 |
| RRF_SERP5_1 | A8GIE3 | 5.78 | 20765.61 |
| RRF_THEP3_1 | B0K9R3 | 8.58 | 21079.44 |
| RRF_RHIME_1 | Q92Q52 | 6.01 | 20832.76 |
| RRF_RHOJR_1 | Q0S282 | 5.2 | 20539.23 |
| RRF_VESOH_1 | A5CXD6 | 8.94 | 21255.44 |
| RRF_RICAH_1 | A8GMC3 | 6.93 | 20875.97 |
| RRF_STRU0_1 | B9DRF1 | 6.03 | 20730.66 |
| RRF_RICCN_1 | Q92J72 | 8.65 | 20811.89 |
| RRF_XANAC_1 | Q8PMK8 | 6.76 | 20352.28 |
| RRF_RICPU_1 | C4K0R2 | 7.89 | 20869.93 |
| RRF_ROSCS_1 | A7NQW5 | 6.34 | 21098.27 |
| RRF_SYNY3_1 | P74456 | 5.65 | 20185.96 |
| RRF_TERTT_1 | C5BQF9 | 5.43 | 20682.7 |
| RRF_STRAW_1 | Q82JY0 | 5.2 | 20740.63 |
| RRF_ZYMMO_1 | Q9X5F0 | 8.71 | 20669.62 |
| RRF_THEPX_1 | B0K1P6 | 8.58 | 21079.44 |
| RRF_THISH_1 | B8GQ53 | 6.36 | 20808.82 |
| RRF_TRIEI_1 | Q10Y49 | 9.13 | 20359.28 |
| RRF_SINMW_1 | A6U8K5 | 6.01 | 20818.74 |
| RRF_WOLSU_1 | Q7MAD8 | 6.77 | 21114.21 |
| RRF_ROSDO_1 | Q166F5 | 5.02 | 20999.89 |
| RRF_SALA4_1 | B5F8T3 | 7.76 | 20555.54 |
