# Supplementary material for: In silico analysis of bacterial translation factors reveal distinct translation event specific pI values
Source: BMC Genomics. 2021 Mar 29;22:220. doi: 10.1186/s12864-021-07472-x (PMC8008671; doi:10.1186/s12864-021-07472-x)
Supplement: Supplementary file 2 — Additional file 2: Proteins of the process of replication. Fig. S1 and S2. Box plot diagram of pI value and molecular weight value distribution respectively of the proteins of the process of replication. Table S2. Accession numbers, pI values and MW values of the proteins of replication factors. [file 12864_2021_7472_MOESM2_ESM.docx]

Additional file 2: Table S2. Accession numbers, pI values and MW values of the proteins of replication factors.

| DnaA | Accession Number | pI | MW |
| --- | --- | --- | --- |
| DNAA_ECOLI_1 | P03004 | 8.77 | 52550.81 |
| DNAA_MYCTU_1 | P9WNW3 | 5.45 | 56566.68 |
| DNAA_BACSU_1 | P05648 | 6.05 | 50859.11 |
| DNAA_THET8_1 | Q9X9D5 | 6.2 | 49387.7 |
| DNAA_STRAQ_1 | Q9ZH75 | 6.14 | 69922.17 |
| DNAA_AQUAE_1 | O66659 | 9.09 | 46840.45 |
| DNAA_SERMA_1 | P29440 | 8.57 | 52173.41 |
| DNAA_VIBCH_1 | Q9KVX6 | 7.25 | 52886.14 |
| DNAA_MYCS2_1 | A0R7K1 | 5.39 | 56664.1 |
| DNAA_TREPA_1 | O83047 | 6.98 | 52965.5 |
| DNAA_SALTY_1 | P35891 | 8.91 | 52598 |
| DNAA_YERPE_1 | Q8Z9U7 | 8.77 | 52176.48 |
| DNAA_VIBHA_1 | P49996 | 7.24 | 53077.29 |
| DNAA_HELPY_1 | O26057 | 8.36 | 51683.33 |
| DNAA1_CHLPN_1 | Q9Z8M9 | 6.45 | 52940.75 |
| DNAA_STRU0_1 | B9DSN7 | 5.26 | 51625.84 |
| DNAA_SYNS9_1 | Q3AYH5 | 8.64 | 51268.74 |
| DNAA_THESQ_1 | B1LC08 | 9.02 | 50256.13 |
| DNAA_TREDE_1 | O87546 | 8.48 | 53782.34 |
| DNAA_TROW8_1 | Q83NZ5 | 6.12 | 54217.89 |
| DNAA_TERTT_1 | C5BKL9 | 8.15 | 66583.31 |
| DNAA_THEM4_1 | A6LIY2 | 9.22 | 50482.71 |
| DNAA_THEYD_1 | B5YGT9 | 7.03 | 49899.65 |
| DNAA_VIBPA_1 | Q87TQ7 | 6.91 | 53049.29 |
| DNAA_VIBTL_1 | B7VGI4 | 8.26 | 53721.12 |
| DNAA_STRT2_1 | Q5M6L8 | 5.33 | 52195.48 |
| DNAA_SYNY3_1 | P49995 | 5.98 | 50050.31 |
| DNAA_THEFY_1 | Q47U23 | 5.72 | 67695.5 |
| DNAA_THEP3_1 | B0KAG0 | 6.64 | 51384.84 |
| DNAA_XANAC_1 | Q8PRG2 | 8.32 | 49538.73 |
| DNAA_THEEB_1 | Q8DL93 | 6.26 | 51053.53 |
| DNAA_TOLAT_1 | C4L755 | 8.75 | 52220.73 |
| DNAA_VIBCB_1 | A7N1E9 | 6.91 | 52990.16 |
| DNAA_XANC5_1 | Q3BZT1 | 8.33 | 49516.66 |
| DNAA_SULDN_1 | Q30UP9 | 9.15 | 49940.72 |
| DNAA_THEMA_1 | P46798 | 9.02 | 50304.26 |
| DNAA_TRIEI_1 | Q11AE3 | 5.99 | 51887.48 |
| DNAA_VIBVY_1 | Q7MQJ7 | 7.77 | 53116.45 |
| DNAA_UREP2_1 | B1AHY7 | 7.98 | 52655.36 |
| DNAA_SYMTH_1 | Q67TK7 | 6.69 | 51596.94 |
| DNAA_STRS7_1 | C0MC62 | 5.23 | 51479.51 |
| DNAA_SYNFM_1 | A0LE53 | 9.16 | 51080.93 |
| DNAA_THEP1_1 | A5IIK7 | 8.96 | 50313.14 |
| DNAA_WOLPM_1 | Q73IZ0 | 9.22 | 52806.67 |
| DNAA_STRRE_1 | Q9ZH76 | 5.47 | 71317.98 |
| DNAA_STRZJ_1 | C1CH81 | 5.74 | 51782.29 |
| DNAA_THEAB_1 | B7IF65 | 9.11 | 50319.33 |
| DNAA_THEPX_1 | B0K0W8 | 6.64 | 51384.84 |
| DNAA_THERP_1 | B9L0U6 | 7.34 | 52220.68 |
| DNAA_WOLSU_1 | Q7MSY2 | 9.21 | 50100.54 |
| DNAA1_PARUW_1 | Q6MC93 | 8.69 | 51316.72 |
| DNAA_ACAM1_1 | B0CDM2 | 6.25 | 51335.54 |
| DNAA_CAMC5_1 | A7GVR3 | 8.55 | 50114.66 |
| DNAA_CHLTE_1 | Q8KGG6 | 6.59 | 55427.34 |
| DNAA_PHOPR_1 | Q6LW50 | 9 | 53877.59 |
| DNAA_BACC2_1 | B7IS20 | 6.05 | 50492.59 |
| DNAA_CLOB1_1 | A7FPR6 | 8.31 | 51323.71 |
| DNAA_STAA3_1 | Q2FKQ5 | 5.3 | 51965.78 |
| DNAA_RICRS_1 | A8GSY1 | 6.85 | 52942.68 |
| DNAA_RICTY_1 | Q68WD8 | 8.46 | 53050.01 |
| DNAA_MYCBT_1 | C1AIZ8 | 5.47 | 56585.72 |
| DNAA_ROSDO_1 | Q16DK6 | 8.93 | 51671.24 |
| DNAA_ROSS1_1 | A5UP91 | 6.66 | 54280.77 |
| DNAA_SACD2_1 | Q21PW4 | 7.74 | 58702.64 |
| DNAA_MYCMM_1 | B2HI46 | 5.42 | 56618.84 |
| DNAA_PSEFS_1 | B0B0A5 | 8.58 | 56287.15 |
| DNAA_PSELT_1 | A8F346 | 8.8 | 51379.52 |
| DNAA_LACRD_1 | A5VHF3 | 5.5 | 49942.5 |
| DNAA_BRUAB_1 | Q57G10 | 8.82 | 55121.18 |
| DNAA_STACT_1 | B9DPX4 | 5.13 | 52188.11 |
| DNAA_LAWIP_1 | Q1MSG8 | 9.15 | 53684.62 |
| DNAA_SALPK_1 | B5BIL4 | 8.91 | 52598 |
| DNAA_FRATN_1 | A0Q3U7 | 8.53 | 55826.6 |
| DNAA_CLOPE_1 | Q8XPG2 | 5.81 | 52245.54 |
| DNAA_BURL3_1 | Q39L82 | 7.27 | 57655.74 |
| DNAA_BURM1_1 | A9AI97 | 7.27 | 57626.66 |
| DNAA_SHEHH_1 | B0TLA4 | 8.63 | 52281.59 |
| DNAA_SHELP_1 | A3Q8S6 | 8.8 | 51684.78 |
| DNAA_STRMK_1 | B2FUW1 | 7.18 | 49960.95 |
| DNAA_BURP1_1 | Q3JXI6 | 7.25 | 58352.4 |
| DNAA_GLOVI_1 | Q7NKK4 | 6.17 | 50134.43 |
| DNAA_NITOC_1 | Q3JF39 | 8.91 | 51356.72 |
| DNAA_NITWN_1 | Q3SMV0 | 9.41 | 52924.34 |
| DNAA_CORJK_1 | Q4JYF7 | 5.38 | 65013.54 |
| DNAA_STRPC_1 | Q1JP60 | 5.56 | 51664.94 |
| DNAA_COXBR_1 | A9N900 | 9.03 | 51095.67 |
| DNAA_RHOS1_1 | A3PFL5 | 8.95 | 51581.45 |
| DNAA_BACVZ_1 | A7Z0C3 | 6.01 | 50843.13 |
| DNAA_ACIBT_1 | A3M0Q4 | 7.03 | 52277.32 |
| DNAA1_CHLCV_1 | Q823P0 | 7.99 | 51099.86 |
| DNAA2_CHLMU_1 | Q9PKB9 | 8.77 | 51446.42 |
| DNAA_BACSK_1 | Q5WM31 | 5.91 | 51183.27 |
| DNAA_CAMFF_1 | A0RLX8 | 8.62 | 49879.28 |
| DNAA_AGRFC_1 | Q8UIH1 | 9.4 | 54639.65 |
| DNAA_BORA1_1 | Q2KTI9 | 8.9 | 53677.57 |
| DNAA_ARCB4_1 | A8EQT0 | 9.02 | 50087.75 |
| DNAA_AROAE_1 | Q5P4P0 | 8.91 | 53724.54 |
| DNAA_RICAE_1 | C3PP41 | 7.21 | 52964.73 |
| DNAA_BORDL_1 | B5RLZ3 | 9.15 | 56365.89 |
| DNAA_PROM4_1 | A9BEI9 | 7.11 | 50638.99 |
| DNAA_LACDA_1 | Q1GC43 | 6.02 | 51558.73 |
| DNAA_CAMJE_1 | Q9PJB0 | 8.86 | 49686.26 |
| DNAA_CYAP7_1 | B7K7Y7 | 5.8 | 51542.93 |
| DNAA_ALCBS_1 | Q0VT30 | 8.3 | 53133.42 |
| DNAA_ALKEH_1 | Q0ACS7 | 8.58 | 51398.73 |
| DNAA_DEIRA_1 | Q9RYE7 | 5.81 | 52042.06 |
| DNAA_ALKOO_1 | A8MEA0 | 6.45 | 51322.66 |
| DNAA_DESPS_1 | Q6ARL8 | 9.53 | 52819.53 |
| DNAA_BIFLS_1 | B7GSF9 | 5.53 | 55268.78 |
| DNAA_BORAP_1 | Q0SN72 | 9.05 | 56523.18 |
| DNAA_AYWBP_1 | Q2NKC5 | 9.33 | 59584.93 |
| DNAA_PASMU_1 | Q9CLQ4 | 7.74 | 51378.78 |
| DNAA_PECAS_1 | Q6CYR4 | 8.77 | 52888.35 |
| DNAA_CHRSD_1 | Q1R1P2 | 8.87 | 55685.34 |
| DNAA_MANSM_1 | Q65VB8 | 7.74 | 51805.97 |
| DNAA_PORG3_1 | B2RGM5 | 7.18 | 53362.31 |
| DNAA_BORBZ_1 | B7J203 | 9.05 | 56912.56 |
| DNAA_MARMS_1 | A6VR65 | 6.07 | 57946.92 |
| DNAA_BACCN_1 | A7GJR9 | 6.06 | 50705.84 |
| DNAA_METI4_1 | B3DWG6 | 8.93 | 51539.7 |
| DNAA_METPP_1 | A2SBM4 | 9.2 | 51849.4 |
| DNAA_KOSOT_1 | C5CH91 | 9.54 | 51060.5 |
| DNAA_ACHLI_1 | A9NE65 | 6.58 | 51804.75 |
| DNAA_BACWK_1 | A9VM90 | 6.27 | 50537.65 |
| DNAA_BARHE_1 | Q6G526 | 8.9 | 55576.96 |
| DNAA_BDEBA_1 | Q6MRS1 | 6.99 | 53997.58 |
| DNAA_CHLAA_1 | A9WAN1 | 6.49 | 53289.81 |
| DNAA_ANAVT_1 | Q3MHA9 | 5.63 | 52241.48 |
| DNAA_ANOFW_1 | B7GFK8 | 6.1 | 51145.43 |
| DNAA_HYDCU_1 | Q31JS5 | 7.83 | 53057.79 |
| DNAA_RICB8_1 | A8GVN1 | 7.1 | 52993.76 |
| DNAA_RICCN_1 | Q92H56 | 6.85 | 52942.68 |
| DNAA_LACCB_1 | B3W6N4 | 5.39 | 50690.8 |
| DNAA_PSE14_1 | Q48QK0 | 8.32 | 56999.01 |
| DNAA_MYCGE_1 | P35888 | 8.75 | 50762.27 |
| DNAA_MYCLE_1 | P46388 | 5.39 | 56313.6 |
| DNAA_ELUMP_1 | B2KAM4 | 6.51 | 51789.38 |
| DNAA_LACSS_1 | Q38ZS4 | 5.26 | 50674.63 |
| DNAA_LARHH_1 | C1D6I2 | 8.66 | 53174.89 |
| DNAA_EXIS2_1 | B1YGB2 | 5.69 | 52421.49 |
| DNAA_STAES_1 | Q8CQK7 | 5.51 | 52096.87 |
| DNAA_SALSV_1 | B4TN07 | 8.91 | 52598 |
| DNAA_STAS1_1 | Q4A180 | 5.54 | 52210.14 |
| DNAA_LEIXX_1 | Q6AHN6 | 5.55 | 53010.22 |
| DNAA_BURCJ_1 | B4E7D1 | 7.81 | 57700.78 |
| DNAA_SHEB9_1 | A9KU72 | 8.81 | 52175.46 |
| DNAA_GEODF_1 | B9M7S1 | 8.81 | 51121.99 |
| DNAA_LISIN_1 | Q92FV2 | 5.73 | 51427.49 |
| DNAA_NATTJ_1 | B2A2Y6 | 6.56 | 52091.18 |
| DNAA_COREF_1 | Q8FUL7 | 5.76 | 63650.54 |
| DNAA_SHESM_1 | Q0HPD4 | 8.61 | 52013.21 |
| DNAA_OCEIH_1 | Q8EU88 | 5.46 | 50692.75 |
| DNAA_RHILO_1 | Q98BG9 | 9.65 | 55023.16 |
| DNAA_CUTAK_1 | Q6ABL5 | 5.12 | 56018.34 |
| DNAA_RHOPA_1 | Q6NDV3 | 9.36 | 52623.98 |
| DNAA_BACHD_1 | Q9RCA2 | 5.87 | 51263.43 |
| DNAA_BACHK_1 | Q6HQ03 | 5.87 | 50494.51 |
| DNAA_BARQU_1 | Q6G0V6 | 9.04 | 55372.88 |
| DNAA_CAUVN_1 | B8GWW5 | 8.94 | 53883.55 |
| DNAA_DESAG_1 | Q30YX9 | 9.32 | 51835.67 |
| DNAA_BACAA_1 | C3P8P5 | 5.87 | 50494.51 |
| DNAA_CHRVO_1 | Q7P259 | 8.22 | 51986.7 |
| DNAA_PELPD_1 | A1AJX2 | 5.72 | 51291.81 |
| DNAA_HERAR_1 | A4G154 | 8.28 | 51830.16 |
| DNAA_SPHAL_1 | Q1GN68 | 9.44 | 50093.79 |
| DNAA_METFK_1 | Q1GXK1 | 9.1 | 53071.01 |
| DNAA_SPICI_1 | P34028 | 7.64 | 51537.13 |
| DNAA_CLOAB_1 | Q97N35 | 6.15 | 50869.04 |
| DNAA_MICLU_1 | P21173 | 5.53 | 56876.27 |
| DNAA_LACF3_1 | B2GEU8 | 5.8 | 50056.77 |
| DNAA_LACH4_1 | A8YW41 | 6.36 | 52370.7 |
| DNAA_MYCCT_1 | P24116 | 9.12 | 51764.53 |
| DNAA_EHRRG_1 | Q5FHH8 | 8.52 | 52674.61 |
| DNAA_PSEE4_1 | Q1I2G4 | 6.73 | 57096.16 |
| DNAA_SALCH_1 | Q57I00 | 8.9 | 52654.07 |
| DNAA_LACP7_1 | A9KPP1 | 5.85 | 51996.66 |
| DNAA_SALEP_1 | B5QUQ0 | 8.91 | 52628.03 |
| DNAA_SALG2_1 | B5RFY7 | 9.03 | 52697.14 |
| DNAA_SALHS_1 | B4TAU9 | 8.91 | 52598 |
| DNAA_CLOK1_1 | B9DXS7 | 6.82 | 51306.84 |
| DNAA_FRAP2_1 | B0TWF7 | 8.39 | 56012.59 |
| DNAA_LEGPL_1 | Q5X0L8 | 8.28 | 51379.9 |
| DNAA_LEPBL_1 | Q056V2 | 8.26 | 50814.67 |
| DNAA_LEPIC_1 | Q72WD6 | 8.26 | 51621.65 |
| DNAA_SHEDO_1 | Q12TC8 | 8.97 | 52242.49 |
| DNAA_LISMF_1 | Q725H0 | 5.73 | 51381.46 |
| DNAA_NEIG1_1 | Q5FAJ2 | 6.08 | 58057.45 |
| DNAA_STRMU_1 | Q8DWN9 | 5.53 | 51370.62 |
| DNAA_NEIMB_1 | Q9JXS7 | 5.93 | 58028.4 |
| DNAA_XYLFT_1 | Q87FC6 | 8.61 | 50227.47 |
| DNAA_NITEC_1 | Q0AK27 | 9.21 | 52380.58 |
| DNAA_YERP3_1 | A7FPB7 | 8.77 | 52176.48 |
| DNAA_NITHX_1 | Q1QS94 | 9.44 | 52756.41 |
| DNAA_NITMU_1 | Q2YD61 | 8.55 | 53730.51 |
| DNAA_NOCFA_1 | Q5Z3Z8 | 5.74 | 73439.67 |
| DNAA_SHIBS_1 | Q31UV5 | 8.77 | 52549.87 |
| DNAA_RHOOB_1 | C1B7S7 | 5.68 | 59120.88 |
| DNAA_ACTSZ_1 | A6VK86 | 8.27 | 51625.79 |
| DNAA_DESVM_1 | B8DN19 | 9.59 | 54696.88 |
| DNAA_PHYMT_1 | B3R0L6 | 9.49 | 54642.95 |
| DNAA_MARHV_1 | A1TWJ0 | 7.75 | 55366.37 |
| DNAA_SODGM_1 | Q2NX49 | 8.77 | 52441.75 |
| DNAA_METCA_1 | Q602N0 | 9.3 | 49980.38 |
| DNAA_BORPA_1 | Q7W2K5 | 8.91 | 52395.21 |
| DNAA_RICPR_1 | Q59758 | 8.46 | 53004.97 |
| DNAA_PROMH_1 | B4F0U5 | 8.26 | 52974.24 |
| DNAA_RUEPO_1 | Q5LWV4 | 7.14 | 52599.51 |
| DNAA_PSEAB_1 | Q02V80 | 8.6 | 57754.33 |
| DNAA_MYCMS_1 | Q6MUM7 | 9.26 | 51754.71 |
| DNAA_CLOCE_1 | B8I3R2 | 5.72 | 50088.2 |
| DNAA_BRUME_1 | Q8YED5 | 8.82 | 55121.18 |
| DNAA_EXISA_1 | C4KZZ3 | 5.65 | 51868 |
| DNAA_BUCAI_1 | P57128 | 9.55 | 52931.2 |
| DNAA_STRAW_1 | Q82FD8 | 5.86 | 72544.93 |
| DNAA_LEPCP_1 | B1Y547 | 9.4 | 55914.25 |
| DNAA_BURCM_1 | Q0BJW1 | 7.29 | 57698.68 |
| DNAA_GEOKA_1 | Q5L3Z2 | 6.05 | 51610.81 |
| DNAA_PSESM_1 | Q88BK3 | 8.61 | 57111.24 |
| DNAA_XANOP_1 | B2SUW3 | 8.63 | 49512.73 |
| DNAA_COLP3_1 | Q48AS7 | 9.1 | 52288.57 |
| DNAA_GEOSW_1 | C5D327 | 6.34 | 51643.91 |
| DNAA_PSYIN_1 | A1T0X4 | 8.3 | 52114.5 |
| DNAA_CORGB_1 | A4Q9R9 | 5.25 | 58435.96 |
| DNAA_PARP8_1 | B2JJ97 | 7.25 | 57212.08 |
| DNAA2_CHLTR_1 | O84277 | 8.67 | 51330.3 |
| DNAA_BACP2_1 | A8F8Y4 | 6.16 | 50968.35 |
| DNAA_ALIF1_1 | Q5E8Z2 | 7.24 | 52803.02 |
| DNAA_ALISL_1 | B6EP46 | 8.22 | 52993.34 |
| DNAA_DEIDV_1 | C1CXJ1 | 6.15 | 51481.54 |
| DNAA_ALKMQ_1 | A6TJ76 | 6.49 | 50732.75 |
| DNAA_HAEIN_1 | P43742 | 7.19 | 51724.06 |
| DNAA_BORBP_1 | Q661I4 | 9.06 | 56483.04 |
| DNAA_PEDPA_1 | Q03I60 | 5.5 | 50946.63 |
| DNAA_BACFR_1 | Q64PL4 | 8.38 | 54182.98 |
| DNAA_RUEST_1 | Q1GKT2 | 6.18 | 53245.93 |
| DNAA_LACLM_1 | A2RH74 | 5.12 | 51316.88 |
| DNAA_BRUSU_1 | Q8G3E7 | 8.62 | 55179.22 |
| DNAA_PSEPU_1 | P0A117 | 7.81 | 56473.51 |
| DNAA_STAHJ_1 | Q4LAL5 | 5.58 | 52114.02 |
| DNAA_STRA1_1 | Q3K425 | 5.27 | 51596.58 |
| DNAA_SERP5_1 | A8G7Q2 | 8.77 | 52209.57 |
| DNAA_STRCO_1 | P27902 | 5.58 | 73182.69 |
| DNAA_MYCUA_1 | A0PKB2 | 5.36 | 56777.03 |
| DNAA_SHEFN_1 | Q08A51 | 8.96 | 52166.44 |
| DNAA_BURM9_1 | A2S8D2 | 7.24 | 58354.43 |
| DNAA_YERE8_1 | A1JT80 | 8.77 | 52127.43 |
| DNAA_NITEU_1 | Q82Y84 | 9.16 | 52536.46 |
| DNAA_AERHH_1 | A0KEC3 | 8.25 | 51864.92 |
| DNAA_HELAH_1 | Q17ZQ6 | 8.55 | 51148.75 |
| DNAA_HELHP_1 | Q7VH57 | 9.1 | 52456.51 |
| DNAA_LISW6_1 | A0AEI7 | 5.73 | 51427.49 |
| DNAA_PHOLL_1 | Q7NAD3 | 8.27 | 52315.57 |
| DNAA_HERA2_1 | A9B496 | 6.29 | 52820.95 |
| DNAA_SHISS_1 | Q3YWB2 | 8.77 | 52550.81 |
| DNAA_JANMA_1 | A6STW2 | 7.75 | 51681.02 |
| DNAA_LACAC_1 | Q5FN15 | 5.84 | 52200.41 |
| DNAA_BRASO_1 | A4YJ98 | 9.51 | 52690.23 |
| DNAA_ENTFA_1 | Q839Z5 | 5.26 | 50409.36 |
| DNAA_MYCPN_1 | Q59549 | 8.92 | 50624.28 |
| DNAA_SALTI_1 | Q8Z2N6 | 8.91 | 52628.03 |
| DNAA_MYCSK_1 | A1U8S0 | 5.44 | 55645.19 |
| DNAA_MYCVP_1 | A1T102 | 5.44 | 55278.51 |
| DNAA_GEOMG_1 | Q39ZS3 | 8.7 | 50942.59 |
| DNAA_CORA7_1 | C3PE72 | 5.7 | 60675.82 |
| DNAA_CORDI_1 | Q6NKL7 | 5.63 | 63029.65 |
| DNAA_HAEDU_1 | Q7VMW1 | 8.17 | 51440.52 |
| DNAA_NOSP7_1 | B2J2B2 | 6.09 | 52313.5 |
| DNAA_OENOB_1 | Q04HR6 | 5.59 | 50900.87 |
| DNAA_RHIME_1 | P35890 | 9.64 | 53579.67 |
| DNAA_BACLD_1 | Q65PM2 | 6.22 | 50923.21 |
| DNAA_CALS4_1 | Q8RDL6 | 6.42 | 51363.61 |
| DNAA_CAMC1_1 | A7ZAW7 | 8.46 | 49918.88 |
| DNAA_AERS4_1 | A4SH46 | 8.73 | 51861.06 |
| DNAA_DESAA_1 | B8FFL8 | 9.11 | 51064.99 |
| DNAA_ANAMM_1 | Q5PB48 | 7.22 | 52836.82 |
| DNAA_CHLAD_1 | B8GBK7 | 6.59 | 53501.19 |
| DNAA_HAES1_1 | Q0I0Y7 | 8.64 | 51862.15 |
| DNAA_AZOSB_1 | A1K1B4 | 8.55 | 54043.82 |
| DNAA_MACCJ_1 | B9E8Z7 | 5.66 | 50985.88 |
| DNAA_RICAH_1 | A8GP75 | 6.82 | 53049.92 |
| DNAA_KINRD_1 | A6W3V4 | 5.65 | 57769.06 |
| DNAA_RICM5_1 | A8F284 | 7.16 | 52869.71 |
| DNAA_BORPD_1 | A9HVA1 | 9.04 | 52714.63 |
| DNAA_BORPE_1 | Q7VSE0 | 8.91 | 52395.21 |
| DNAA_MYCA9_1 | B1MDH6 | 5.31 | 55031.26 |
| DNAA_MYCAV_1 | P49990 | 6.36 | 56796.54 |
| DNAA_ROSCS_1 | A7NFB8 | 6.97 | 54090.59 |
| DNAA_SALA4_1 | B5EYX2 | 8.91 | 52598 |
| DNAA_LACP3_1 | Q03D55 | 5.45 | 50613.72 |
| DNAA_LACPL_1 | Q890K8 | 5.69 | 51462.56 |
| DNAA_STRGG_1 | B1VPF0 | 5.99 | 69566.86 |
| DNAA_GEOSL_1 | Q74GG6 | 8.57 | 50190.79 |
| DNAA_SHEON_1 | Q8EKT2 | 8.61 | 51970.18 |
| DNAA_SHEPC_1 | A4Y1A4 | 8.6 | 52238.5 |
| DNAA_GEOUR_1 | A5GDX1 | 8.66 | 51361.52 |
| DNAA_SHESH_1 | A8FP46 | 8.94 | 52421.71 |
| DNAA_NOSS1_1 | Q8YVG9 | 5.55 | 52349.71 |
| DNAA_ZYMMO_1 | Q9S493 | 8.77 | 55195.45 |
| DNAA_RHIL3_1 | Q1MMD6 | 9.74 | 54079.32 |
| DNAA_SHEWM_1 | B1KCX3 | 8.79 | 52210.49 |
| DNAA_BIFA0_1 | B8DV06 | 5 | 64801.4 |
| DNAA_DECAR_1 | Q47K71 | 8.82 | 52294.64 |
| DNAA_HALHL_1 | A1WWE0 | 6.35 | 50743.6 |
| DNAA_BORBR_1 | Q7WDJ9 | 8.91 | 53307.26 |
| DNAA_MAGMM_1 | A0L3I7 | 6.04 | 49675.58 |
| DNAA_CLAMS_1 | B0RH69 | 6.17 | 53079.32 |
| DNAA_BORHD_1 | B2S0D9 | 9.05 | 56363.71 |
| DNAA_KLEP3_1 | B5XT51 | 8.76 | 52394.79 |
| DNAA_RICPU_1 | C4K1R9 | 6.85 | 52914.63 |
| DNAA_CLOB8_1 | A6LPB1 | 6.54 | 51003.16 |
| DNAA_BORT9_1 | A1QZM2 | 9 | 56341.84 |
| DNAA_EHRCR_1 | Q2GG27 | 8.37 | 52767.7 |
| DNAA_GEOSM_1 | C6E7Q5 | 7.14 | 52616.57 |
| DNAA_BURTA_1 | Q2STL6 | 7.22 | 58461.52 |
| DNAA_RALPJ_1 | B2UCF1 | 7.27 | 59131.49 |
| DNAA_ACIAD_1 | Q6FG21 | 8.15 | 52442.61 |
| DNAA_BACTN_1 | Q8A5U5 | 7.27 | 53251.12 |
| DNAA_DESRM_1 | A4J0F0 | 6.13 | 50667.94 |
| DNAA_AZOVD_1 | C1DFU2 | 8.31 | 54006.77 |
| DNAA_PECCP_1 | C6DGH7 | 8.76 | 52780.15 |
| DNAA_IDILO_1 | Q5QY39 | 8.73 | 51510.61 |
| DNAA_JANSC_1 | Q28WI0 | 7.77 | 53129.06 |
| DNAA_RICFE_1 | Q4UMJ3 | 6.82 | 52931.7 |
| DNAA_LACBA_1 | Q03UE4 | 5.22 | 51078.93 |
| DNAA_LACJO_1 | Q74M34 | 6.02 | 51656 |
| DNAA_SALNS_1 | B4SYA8 | 8.91 | 52598 |
| DNAA_ERWT9_1 | B2VCE3 | 8.77 | 52288.76 |
| DNAA_FLAPJ_1 | A6GYW8 | 8.85 | 55019.23 |
| DNAA_CLONN_1 | A0Q3U6 | 6.7 | 51300.71 |
| DNAA_LEPBA_1 | B0S907 | 8.22 | 51325.8 |
| DNAA_SHEAM_1 | A1S1G9 | 8.61 | 51548.69 |
| DNAA_NAUPA_1 | B9L735 | 9.1 | 50490.63 |
| DNAA_GEOTN_1 | A4IJ84 | 6.45 | 51351.71 |
| DNAA_SHEPW_1 | B8CH71 | 8.78 | 52098.54 |
| DNAA_GLUOX_1 | Q5FUU1 | 6.02 | 53923.39 |
| DNAA_RALSO_1 | Q8XTV4 | 6.91 | 58491.06 |
| DNAA_ONYPE_1 | Q6YRL3 | 9.23 | 59878 |
| DNAA_PARPJ_1 | B2SZ75 | 7.79 | 59566.43 |
| DNAA_SHIDS_1 | Q329B6 | 8.77 | 52535.84 |
| DNAA_RHOE4_1 | C0ZLE1 | 5.66 | 58240.78 |
| DNAA_RHOJR_1 | Q0SAG7 | 5.73 | 59214.95 |
| DNAA_CUPMC_1 | Q1LSI9 | 7.01 | 63550.87 |
| DNAA_CUPNH_1 | Q0KFR8 | 7.05 | 64647.38 |
| DNAA_CUPTR_1 | B2AFZ7 | 7.12 | 64954.65 |
| DNAA_CAUSK_1 | B0T135 | 9.25 | 53790.4 |
| DNAA_HAHCH_1 | Q2SQZ9 | 7.72 | 52806.51 |
| DNAA_PARXL_1 | Q147F0 | 7.8 | 59605.46 |
| DNAA_HELMI_1 | B0TAK8 | 6.35 | 50373.58 |
| DNAA_MESFL_1 | Q6F2A9 | 8.83 | 50263.14 |
| DNAA_RICCK_1 | A8EY77 | 7.16 | 53039.86 |
| DNAA_MICAN_1 | B0JGA6 | 6.03 | 50645.98 |
| DNAA_BORRA_1 | B5RRN9 | 9.15 | 56398.97 |
| DNAA_EDWI9_1 | C5BHC5 | 8.77 | 52054.38 |
| DNAA_BRADU_1 | Q89W63 | 9.53 | 52409.83 |
| DNAA_SALDC_1 | B5FN10 | 8.91 | 52598 |
| DNAA_MYCPA_1 | Q9L7L7 | 5.35 | 56620.13 |
| DNAA_GEOBB_1 | B5E7P6 | 7.68 | 52407.36 |
| DNAA_CLOTH_1 | A3DHZ4 | 5.85 | 50281.37 |
| DNAA_XANP2_1 | A7IB67 | 8.84 | 55420.28 |
| DNAA_SHEPA_1 | A8GYE3 | 8.62 | 52232.53 |
| DNAA_BURVG_1 | A4J9S6 | 7.84 | 57775.94 |
| DnaB | Accession Number | pI | MW |
| DNAB_ECOLI_1 | P0ACB0 | 4.93 | 52390.08 |
| DNAB_MYCTU_1 | P9WMR3 | 5.32 | 43777.05 |
| DNAB_MYCBO_1 | P59966 | 5.32 | 43777.05 |
| DNAB_SYNY3_1 | Q55418 | 5.39 | 41942.27 |
| DNAB_NOSS1_1 | Q8YZA1 | 5.15 | 42763.04 |
| DNAB_RHOMR_1 | O30477 | 6.31 | 46556.39 |
| DNAB_SALTY_1 | P0A1Q4 | 4.95 | 52687.41 |
| DNAB_MYCLE_1 | P46394 | 4.52 | 24716.78 |
| DNAB_TREPA_1 | O83097 | 5.48 | 47795.94 |
| DNAB_MYCGE_1 | P47340 | 6.6 | 53884.43 |
| DNAB_HAEIN_1 | P45256 | 5.25 | 52362.46 |
| DNAB_HELPY_1 | O25916 | 5.2 | 55726.47 |
| DNAB_BUCAI_1 | P57611 | 6.32 | 51986.44 |
| DNAB_SALTI_1 | P0A1Q5 | 4.95 | 52687.41 |
| DNAB_PASMU_1 | Q9CNL6 | 5.38 | 52011.25 |
| DNAB_SHIFL_1 | P0ACB1 | 4.93 | 52390.08 |
| DNAB_LEIXX_1 | Q6ABX1 | 5.37 | 50007.03 |
| DNAB_RICCN_1 | Q92HG8 | 5.61 | 55755.82 |
| DNAB_RICPR_1 | Q9ZD08 | 6.7 | 54940.27 |
| DNAB_MYCPN_1 | P75539 | 5.55 | 54490.21 |
| DNAB_RICBR_1 | Q1RI04 | 5.73 | 56183.22 |
| DNAB_RICFE_1 | Q4UL62 | 5.62 | 55839.93 |
| DNAB_RICTY_1 | Q68WJ2 | 6.16 | 56193.61 |
| DnaC | Accession Number | pI | MW |
| DNAC_ECOLI_1 | P0AEF0 | 9.09 | 27935.09 |
| DNAC_BACSU_1 | P37469 | 4.78 | 50576.34 |
| DNAC_SHIFL_1 | P0AEF3 | 9.09 | 27935.09 |
| DNAC_BUCAI_1 | P57134 | 9.14 | 28444.53 |
| SSB | Accession Number | pI | MW |
| SSB_ECOLI_1 | P0AGE0 | 5.45 | 18843.8 |
| SSBB_BACSU_1 | C0SPB6 | 6.57 | 12520.15 |
| SSB_DEIRA_1 | Q9RY51 | 5.34 | 32722.38 |
| SSB_MYCTU_1 | P9WGD5 | 5.12 | 17353.11 |
| SSB_PSEAE_1 | P40947 | 5.47 | 18426.27 |
| SSB2_STRCO_1 | Q9X8U3 | 5.27 | 19906.61 |
| SSB_OCEIH_1 | Q8CX55 | 4.82 | 18138.79 |
| SSB1_STRP6_1 | Q5XE77 | 7.85 | 14787.96 |
| SSB_RALSO_1 | Q8Y2B4 | 5.94 | 18697.54 |
| SSB_RHILO_1 | Q98M41 | 6.63 | 18563.49 |
| SSB_CLOPE_1 | Q8XH44 | 4.65 | 16023.57 |
| SSB_CLOTE_1 | Q899R2 | 4.94 | 16180.96 |
| SSB_RHOBA_1 | P59932 | 5.53 | 18108.06 |
| SSB_RICFE_1 | Q4UJW3 | 6.21 | 17393.32 |
| SSB_HAEDU_1 | P59930 | 5.71 | 19697.52 |
| SSB_STRR6_1 | P66855 | 5.41 | 17351.21 |
| SSB_THEAQ_1 | Q9KH06 | 5.21 | 30028.73 |
| SSB_MYCLE_1 | P46390 | 5.14 | 17700.54 |
| SSB_MYCPE_1 | Q8EWT6 | 4.71 | 21421.54 |
| SSB1_CHLTE_1 | Q8KB47 | 6.74 | 17804.61 |
| SSB_BACCR_1 | Q814G6 | 5.19 | 18494.29 |
| SSB_BORBR_1 | P66847 | 5.9 | 18100.09 |
| SSB1_NOSS1_1 | P0A4K0 | 8.79 | 13253.92 |
| SSB_BORPE_1 | P66846 | 5.9 | 18100.09 |
| SSB1_SALTI_1 | P0A2F7 | 5.46 | 18942.95 |
| SSB2_PSEPU_1 | Q8VMM4 | 6.13 | 20445.74 |
| SSB2_STAAC_1 | Q5HJ26 | 6.59 | 15608.21 |
| SSB_NEIMA_1 | P66848 | 5.78 | 19452.65 |
| SSB_BRADU_1 | Q89L50 | 6.61 | 17522.49 |
| SSB1_STRA3_1 | P66850 | 5.19 | 18165.81 |
| SSB_PASMU_1 | Q9CJP4 | 5.54 | 18687.7 |
| SSB_CALS4_1 | Q8R6M2 | 4.49 | 16961.01 |
| SSB_CAMJE_1 | O69302 | 5.27 | 20683.58 |
| SSB_ENTFA_1 | Q839Y9 | 4.84 | 20604.2 |
| SSB_STAES_1 | Q8CNK0 | 5.43 | 15159.02 |
| SSB_HELPJ_1 | Q9ZJY2 | 5.74 | 20381.59 |
| SSB_VIBCH_1 | Q9KUW2 | 5.69 | 19672.9 |
| SSB_VIBVU_1 | Q8DCJ0 | 5.44 | 19794.97 |
| SSB1_SYNY3_1 | Q55499 | 6.73 | 13656.31 |
| SSB_BUCBP_1 | Q89A53 | 5.91 | 18716.96 |
| SSB_PROVU_1 | Q8L2A6 | 6.91 | 21129.31 |
| SSB_RICTY_1 | Q68Y11 | 6.21 | 17452.43 |
| SSB_SHIFL_1 | P0AGE3 | 5.45 | 18843.8 |
| SSB_ANAVA_1 | P0A4K1 | 8.79 | 13253.92 |
| SSB_AQUAE_1 | O66475 | 4.85 | 17132.35 |
| SSB_BIFLO_1 | Q8G757 | 4.9 | 22583.36 |
| SSB1_LISIN_1 | Q92FR5 | 4.98 | 19604.25 |
| SSB_XYLFT_1 | Q87DQ5 | 5.89 | 18227.2 |
| SSB_PROMI_1 | P28046 | 5.2 | 18699.71 |
| SSB_CORGL_1 | Q8NLG0 | 4.81 | 23300.91 |
| SSB_RICBR_1 | Q1RK72 | 6.09 | 16846.81 |
| SSB_CUPNH_1 | P59927 | 8.89 | 20451.61 |
| SSB_HAEIN_1 | P44409 | 5.41 | 18753.55 |
| SSB_LACPL_1 | Q890K1 | 4.99 | 21684.07 |
| SSB_THEEB_1 | Q8DIU1 | 7.86 | 13371.22 |
| SSB_THETH_1 | O85824 | 5.53 | 30263.02 |
| SSB_TREPA_1 | O83101 | 5.04 | 19043.04 |
| SSB_VIBPA_1 | Q87LA3 | 5.18 | 19563.62 |
| SSB_WOLSU_1 | P59933 | 5.25 | 17789.78 |
| SSB_XANCP_1 | Q8P778 | 5.5 | 18632.53 |
| SSB1_LACLA_1 | Q9CGS5 | 8.76 | 16643.67 |
| SSB_BORBU_1 | O51141 | 5.21 | 17166.22 |
| SSB1_LISMO_1 | Q8YAR8 | 4.98 | 19493.14 |
| SSB1_SALTY_1 | P0A2F6 | 5.46 | 18942.95 |
| SSB_NITEU_1 | Q82S98 | 5.23 | 16811.73 |
| SSB_BRUSU_1 | Q8G0J1 | 5.54 | 18371.15 |
| SSB2_TROW8_1 | P66857 | 9.09 | 16649.61 |
| SSB_CAUVC_1 | Q9A894 | 5.54 | 17970.78 |
| SSB_CHLCV_1 | Q823K0 | 5.07 | 17828.09 |
| SSB_CHLMU_1 | Q9PKZ4 | 4.88 | 17265.22 |
| SSB_COREF_1 | Q8FLP9 | 4.85 | 23513.23 |
| SSB_HELHP_1 | P59931 | 5.91 | 18848.77 |
| SSB_MYCBO_1 | P0A611 | 5.12 | 17353.11 |
| SSB_MYCGE_1 | P47337 | 4.67 | 17973.02 |
| SSB_PAEAU_1 | Q8KSB6 | 5.12 | 18266.86 |
| SSB_PSESM_1 | Q889U1 | 5.88 | 20974.1 |
| SSB3_CLOAB_1 | Q97CX3 | 4.86 | 15954.78 |
| SSB_RICPR_1 | Q9ZCC2 | 6.3 | 17477.44 |
| SSB_SHEHA_1 | P77953 | 5.87 | 24865.25 |
| SSB_SHEON_1 | Q8EA81 | 5.9 | 25773.34 |
| SSB_BACTN_1 | Q8A7M7 | 5.62 | 17761.82 |
| SSB_MYCPU_1 | Q98PV9 | 5.32 | 21091.03 |
| SSB2_STRAW_1 | Q82CI4 | 6.3 | 17111.92 |
| SSB_BRUME_1 | Q8YHC2 | 5.54 | 18401.18 |
| SSB_ONYPE_1 | P60471 | 8.71 | 12051.79 |
| SSB_RHIME_1 | P56898 | 5.54 | 18962.68 |
| SSB_RHOS4_1 | Q9ZAQ8 | 5.94 | 18628.35 |
| SSB_FUSNN_1 | Q8RE26 | 4.86 | 17019.73 |
| SSB_STRMU_1 | Q8DSD8 | 4.98 | 18331.97 |
| SSB_XANAC_1 | Q8PIJ2 | 5.48 | 19095.94 |
| SSB_BACAN_1 | Q81JI3 | 5.19 | 18692.41 |
| SSB_BORPA_1 | P59928 | 6.6 | 17783.82 |
| SSB_MYCPN_1 | P75542 | 4.3 | 18424.29 |
| SSB_BRUA2_1 | Q2YPX7 | 5.93 | 18398.22 |
| SSB_PAENI_1 | Q8GAN5 | 5.32 | 18352.97 |
| SSB_CHLPN_1 | Q9Z8F7 | 5.41 | 17823.11 |
| SSB_CHLTR_1 | O84048 | 4.83 | 17147.03 |
| SSB_COXBU_1 | Q83EP4 | 5.61 | 17437.12 |
| SSB_RICCN_1 | Q92G30 | 6.07 | 17435.33 |
| SSB_SERMA_1 | P25762 | 5.45 | 18677.52 |
| SSB_THEMA_1 | Q9WZ73 | 4.8 | 16298.4 |
| SSB_WIGBR_1 | Q8D254 | 6.63 | 18782.13 |
| SSB_YERPE_1 | Q8ZJ06 | 5.21 | 19164.15 |
| SSB_BACHD_1 | Q9K5N9 | 4.88 | 18219.95 |
| SSB_MYCS2_1 | Q9AFI5 | 5.15 | 17401.12 |
| SSB_LEPIC_1 | Q72UU3 | 8.86 | 13134.97 |
| SSB_UREPA_1 | Q9PPT7 | 4.63 | 18500.29 |
| Gyrase subunit A | Accession Number | pI | Mw |
| GYRA_ECOLI_1 | P0AES4 | 5.08 | 96832.32 |
| GYRA_MYCTU_1 | P9WG47 | 5.41 | 92143.12 |
| GYRA_MYCLE_1 | Q57532 | 6.4 | 14486.38 |
| GYRA_MICLC_1 | C5C7X9 | 5.06 | 99097.3 |
| GYRA_MYCBP_1 | A0A0G2Q9F8 | 5.41 | 92345.39 |
| GYRA_MYCSM_1 | Q59556 | 6.52 | 94924.71 |
| GYRA_THET8_1 | Q5SIL4 | 6.14 | 89119.93 |
| GYRA_SALTY_1 | P37411 | 4.98 | 97064.23 |
| GYRA_BUCBP_1 | Q89AS3 | 9.81 | 95829.53 |
| GYRA_CAMJE_1 | Q03470 | 5.17 | 96973.8 |
| GYRA_KLEOX_1 | P14829 | 4.99 | 97155.39 |
| GYRA_STAAW_1 | Q8NKW8 | 5.03 | 99350.97 |
| GYRA_PECCA_1 | P41513 | 5.02 | 97588.83 |
| GYRA_STRP8_1 | Q8P116 | 6.78 | 92743.77 |
| GYRA_AMICL_1 | D5ECW5 | 5.43 | 91065.33 |
| GYRA_RICTY_1 | Q68XG2 | 5.49 | 101154.9 |
| GYRA_THEMA_1 | O33926 | 7.7 | 90448.24 |
| GYRA_BORBU_1 | O51396 | 6.85 | 91379.5 |
| GYRA_CHLMU_1 | Q9PKK4 | 6.21 | 93883.88 |
| GYRA_DEHLB_1 | D8K235 | 8.08 | 90330.1 |
| GYRA_DEIDV_1 | C1CVF4 | 5.9 | 89873.87 |
| GYRA_HELPY_1 | P48370 | 5.64 | 92500.22 |
| GYRA_LAWIP_1 | Q1MQ89 | 6.67 | 91735.35 |
| GYRA_MYCGA_1 | P47719 | 5.91 | 94332.58 |
| GYRA_STAES_1 | P0C0R0 | 4.85 | 100113.6 |
| GYRA_STRPN_1 | P72524 | 5.85 | 92053.3 |
| GYRA_AERSA_1 | P48369 | 4.87 | 101333.6 |
| GYRA_UREPA_1 | Q9PR63 | 6.05 | 94251.95 |
| GYRA_HAEIN_1 | P43700 | 5.1 | 97818.14 |
| GYRA_MYCPN_1 | P22446 | 8.52 | 93356.56 |
| GYRA_NEIGO_1 | P48371 | 5.39 | 101530.4 |
| GYRA_SHIFL_1 | P0AES5 | 5.08 | 96963.51 |
| GYRA_BRAHW_1 | C0R046 | 6.7 | 93275.26 |
| GYRA_CHLPN_1 | Q9Z8R4 | 6.62 | 93838.73 |
| GYRA_KOSOT_1 | C5CHA8 | 8.54 | 90484.38 |
| GYRA_MYCGE_1 | P47250 | 7.62 | 93681.6 |
| GYRA_RICFE_1 | Q4UKM1 | 5.27 | 101121.4 |
| GYRA_TREPA_1 | O83051 | 8.25 | 89926.46 |
| GYRA_BORHD_1 | B2S0D7 | 6.09 | 91509.75 |
| GYRA_CAMFE_1 | P47235 | 5.12 | 95820.71 |
| GYRA_CLOAB_1 | P94605 | 5.59 | 92612.17 |
| GYRA_FIBSS_1 | P35810 | 5.17 | 99633.45 |
| GYRA_GRABC_1 | Q0BST5 | 5.09 | 103174.6 |
| GYRA_NOCSJ_1 | A1SCM2 | 4.89 | 100698 |
| GYRA_RICBR_1 | Q1RID9 | 5.14 | 100679.4 |
| GYRA_SYNY3_1 | Q55738 | 5.1 | 95073.35 |
| GYRA_LEPBA_1 | B0S912 | 5.66 | 93818.42 |
| GYRA_MYCA5_1 | B3PN30 | 5.47 | 100871.2 |
| GYRA_RICPR_1 | P41080 | 5.42 | 101080.7 |
| GYRA_RICCN_1 | Q92IZ6 | 5.37 | 100701 |
| GYRA_BACHD_1 | O50628 | 5.57 | 93873.05 |
| GYRA_CHLTR_1 | O84192 | 6.26 | 94233.15 |
| GYRA_STRCO_1 | P35885 | 5.38 | 94583.3 |
| GYRA_BACSU_1 | P05653 | 5.37 | 92098.99 |
| Gyrase subunit B | Accession number | pI | Molecular Weight |
| GYRB_ECOLI_1 | P0AES6 | 5.72 | 89818.72 |
| GYRB_MYCSM_1 | P0C559 | 5.74 | 74381.01 |
| GYRB_MYCTU_1 | P9WG45 | 5.98 | 74090.76 |
| GYRB_MICLC_1 | C5C7X8 | 4.95 | 78896.24 |
| GYRB_THET8_1 | Q5SHZ4 | 5.96 | 70524.32 |
| GYRB_SALTY_1 | P0A2I3 | 5.78 | 89764.57 |
| GYRB_MYCLE_1 | Q59533 | 5.78 | 74674.38 |
| GYRB_MYCBP_1 | A0A0G2Q9D6 | 6.18 | 78455.74 |
| GYRB_STAAU_1 | P0A0K8 | 5.61 | 72408.67 |
| GYRB_ENTFA_1 | Q839Z1 | 5.54 | 71878.35 |
| GYRB_BORBU_1 | P33769 | 6.4 | 71451.02 |
| GYRB_CLOAB_1 | P94604 | 5.8 | 71614.14 |
| GYRB_STRCO_1 | P35886 | 5.24 | 75464.48 |
| GYRB_CAUVN_1 | B8GXQ0 | 5.49 | 88211.75 |
| GYRB_STAEQ_1 | Q5HK03 | 5.7 | 72503.67 |
| GYRB_STRPQ_1 | P0DG05 | 5.41 | 72352.89 |
| GYRB_MYCAT_1 | Q9ZAQ6 | 8.37 | 71661.79 |
| GYRB_RICTY_1 | Q68WF6 | 6.09 | 90952.28 |
| GYRB_VIBCH_1 | Q9KVX3 | 5.7 | 89519.49 |
| GYRB_BACHD_1 | O50627 | 5.76 | 71232.55 |
| GYRB_RICPR_1 | Q9ZCX2 | 6.03 | 90823.14 |
| GYRB_HAEIN_1 | P43701 | 5.53 | 89884.9 |
| GYRB_BUCAI_1 | P57126 | 9.16 | 92411.13 |
| GYRB_RICFE_1 | Q4UKX5 | 5.68 | 90595.34 |
| GYRB_SALTI_1 | P0A2I4 | 5.78 | 89764.57 |
| GYRB_VIBPA_1 | O51859 | 5.54 | 89442.22 |
| GYRB_BORHE_1 | Q9ZFK1 | 6.26 | 70848.11 |
| GYRB_MYCGE_1 | P47249 | 5.93 | 73605.47 |
| GYRB_SYNY3_1 | P77966 | 6.19 | 122819.3 |
| GYRB_TREDE_1 | O87545 | 6.22 | 71391.77 |
| GYRB_BACAN_1 | Q9X3Y6 | 6.02 | 71896.49 |
| GYRB_MYCPN_1 | P22447 | 5.73 | 73809.67 |
| GYRB_PSEAE_1 | Q9I7C2 | 5.62 | 90188.96 |
| GYRB_RICBR_1 | Q1RHT8 | 5.86 | 90842.68 |
| GYRB_SPICI_1 | P34031 | 6.45 | 72763.12 |
| GYRB_STRR6_1 | P0A4M0 | 5.37 | 72237.51 |
| GYRB_MYCHP_1 | P43053 | 5.65 | 72639.84 |
| GYRB_TREPA_1 | O08399 | 7.65 | 70910.42 |
| GYRB_MYCPA_1 | Q9L7L3 | 6.06 | 74531.12 |
| GYRB_PSEPU_1 | P13364 | 5.64 | 90077.94 |
| GYRB_CAMJE_1 | O87667 | 5.39 | 86648.59 |
| GYRB_CHLMU_1 | Q9PKK3 | 5.43 | 89583.72 |
| GYRB_CHLTR_1 | O84193 | 5.4 | 89774.23 |
| GYRB_HELPJ_1 | Q9ZLX3 | 5.9 | 87439.48 |
| GYRBR_STRNV_1 | P50074 | 5.76 | 74600.43 |
| GYRB_THEMA_1 | P77993 | 6.41 | 72427.73 |
| GYRB_MYCGA_1 | P47720 | 6.3 | 73712.77 |
| GYRB_RICCN_1 | Q92H87 | 5.78 | 90794.91 |
| GYRB_SHIFL_1 | P0AES8 | 5.72 | 89818.72 |
| GYRB_CHLPN_1 | Q9Z8R3 | 5.33 | 90571.43 |
| GYRB_BACSU_1 | P05652 | 5.5 | 71503.65 |
| GYRB_MYXXA_1 | O33367 | 8.41 | 89636.59 |
| GYRB_NEIGO_1 | P22118 | 5.45 | 88164.32 |
| DNA Primase | Accession number | pI | Molecular Weight |
| DNAG_ECOLI_1 | P0ABS5 | 5.68 | 65564.63 |
| DNAG_GEOSE_1 | Q9X4D0 | 6.47 | 67110.68 |
| DNAG_BACSU_1 | P05096 | 6.68 | 68736.19 |
| DNAG_MYCTU_1 | P9WNW1 | 6.38 | 69592.95 |
| DNAG_STAAU_1 | O05338 | 5 | 70001.57 |
| DNAG_STAAM_1 | P63964 | 4.95 | 70031.55 |
| DNAG_LISIN_1 | Q92BQ5 | 5.18 | 71801.36 |
| DNAG_BACHD_1 | Q9KD44 | 6.38 | 68958.79 |
| DNAG_STAES_1 | Q8CP23 | 5.41 | 70421.56 |
| DNAG_STAEQ_1 | Q5HNY6 | 5.41 | 70421.56 |
| DNAG_STAAC_1 | Q5HFJ8 | 4.97 | 69293.82 |
| DNAG_STAAN_1 | P63965 | 4.95 | 70031.55 |
| DNAG_STAAW_1 | Q8NWB7 | 4.94 | 70073.67 |
| DNAG_AQUAE_1 | O67465 | 8.45 | 57206.03 |
| DNAG_STAAR_1 | Q6GGD7 | 4.96 | 69275.76 |
| DNAG_STAAS_1 | Q6G904 | 4.94 | 70115.71 |
| DNAG_RICPR_1 | P30103 | 8.92 | 68684.09 |
| DNAG_SYNE7_1 | P74893 | 6.24 | 78524.17 |
| DNAG_LACLA_1 | Q04505 | 4.94 | 72751.22 |
| DNAG_CLOAB_1 | P33655 | 8.15 | 68627.69 |
| DNAG_ENTFA_1 | P52308 | 5.12 | 73058.31 |
| DNAG_HAEIN_1 | Q08346 | 6.47 | 68000.92 |
| DNAG_PSEAE_1 | Q9I5W0 | 6.79 | 74176 |
| DNAG_HELPY_1 | P56064 | 8.97 | 63723.39 |
| DNAG_MYCPN_1 | P75426 | 6.28 | 71537.34 |
| DNAG_BUCAI_1 | P57164 | 9.48 | 67772.2 |
| DNAG_BUCAP_1 | P32000 | 9.54 | 68269.6 |
| DNAG_DEIRA_1 | Q9RWR5 | 5.89 | 62508.28 |
| DNAG_ECO57_1 | P0ABS6 | 5.68 | 65564.63 |
| DNAG_MYCLE_1 | Q9CCG2 | 5.58 | 70761.22 |
| DNAG_MYXXA_1 | P50070 | 7.24 | 66846.81 |
| DNAG_PASMU_1 | Q9CLI9 | 6.54 | 66308.19 |
| DNAG_SALTY_1 | P07362 | 6.15 | 65362.5 |
| DNAG_CHLMU_1 | Q9PLC9 | 8.04 | 68810.41 |
| DNAG_CHLTR_1 | O84799 | 8.09 | 68037.19 |
| DNAG_MYCTO_1 | P9WNW0 | 6.38 | 69592.95 |
| DNAG_TREPA_1 | O83505 | 8.02 | 67893.04 |
| DNAG_UREPA_1 | Q9PPZ6 | 9.1 | 75390.94 |
| DNAG_NEIMA_1 | P57028 | 6.01 | 65725.97 |
| DNAG_PSEPK_1 | P0A118 | 6.38 | 73829.52 |
| DNAG_LEGPN_1 | P71481 | 8.81 | 65674.28 |
| DNAG_LISMO_1 | P47762 | 5.09 | 71756.43 |
| DNAG_RICCN_1 | Q92FZ7 | 7.24 | 68423.34 |
| DNAG_BORBU_1 | O51653 | 9.08 | 59980.5 |
| DNAG_MYCGE_1 | P47492 | 8.82 | 71061.73 |
| DNAG_MYCPU_1 | Q98QB3 | 8.99 | 70907.41 |
| DNAG_HELPJ_1 | Q9ZN49 | 8.92 | 63815.56 |
| DNAG_RICBR_1 | Q1RKH8 | 6.11 | 68874.8 |
| DNAG_RICTY_1 | Q68VQ4 | 8.74 | 68658.95 |
| DNAG_SHIFL_1 | P0ABS7 | 5.68 | 65564.63 |
| DNAG_STRCO_1 | Q9S1N4 | 5.98 | 69744.01 |
| DNAG_BUCBP_1 | Q89B09 | 9.59 | 67638.03 |
| DNAG_MYCBO_1 | P63963 | 6.38 | 69592.95 |
| DNAG_CAMJE_1 | Q9PM37 | 8.61 | 69055.6 |
| DNAG_SYNY3_1 | P74143 | 6.07 | 73079.09 |
| DNAG_CHLPN_1 | Q9Z6W4 | 7.32 | 67642.12 |
| DNAG_MYCS2_1 | O52200 | 6.04 | 69815.76 |
| DNAG_THEMA_1 | Q9X1G3 | 7.05 | 65132.73 |
| DNAG_ECOL6_1 | Q8FDG5 | 5.68 | 65550.6 |
| DNAG_NEIMB_1 | P57029 | 5.97 | 65915.11 |
| DNAG_PSEPU_1 | P0A119 | 6.38 | 73829.52 |
| DNAG_RICFE_1 | Q4UJT0 | 7.22 | 68399.29 |
| DNA Polymerase III subunit alpha | Accession number | pI | Molecular Weight |
| DPO3A_ECOLI_1 | P10443 | 5.16 | 129904.6 |
| DPO3A_CAUVN_1 | B8GWS6 | 5.87 | 125538.6 |
| DPO3A_MYCTU_1 | P9WNT7 | 5.5 | 129323 |
| DPO3A_SALTY_1 | P14567 | 5.19 | 130217.9 |
| DPO3A_DEIRA_1 | Q9RX08 | 5.18 | 149277.1 |
| DPO3A_VIBCH_1 | P52022 | 5.2 | 130056.9 |
| DPO3A_MYCBO_1 | P63978 | 5.5 | 129323 |
| DPO3A_SACEN_1 | P34699 | 5.29 | 131196.8 |
| DPO3A_CHLPN_1 | Q9Z7N8 | 6 | 140075.7 |
| DPO3A_LACLA_1 | Q9CI70 | 6.06 | 121571.7 |
| DPO3A_NEIMA_1 | Q9JVX8 | 5.27 | 126976.3 |
| DPO3A_PSEAE_1 | Q9HXZ1 | 5.27 | 130904.7 |
| DPO3A_HELPJ_1 | Q9ZJF9 | 5.92 | 138043.1 |
| DPO3A_RICCN_1 | Q92GB2 | 5.74 | 132593.9 |
| DPO3A_CAMJE_1 | Q9PPI9 | 5.53 | 137340.9 |
| DPO3A_STAAC_1 | Q5HF71 | 6.05 | 122963.9 |
| DPO3A_XYLFA_1 | Q9PGU4 | 6 | 133503.3 |
| DPO3A_CHLMU_1 | Q9PJJ7 | 5.86 | 139893.7 |
| DPO3A_CHLTR_1 | O84549 | 5.79 | 139545.5 |
| DPO3A_RICPR_1 | O05974 | 7.28 | 133463.4 |
| DPO3A_STAEQ_1 | Q5HNK2 | 6.1 | 123361.1 |
| DPO3A_BACSU_1 | O34623 | 6.05 | 125350 |
| DPO3A_STRPY_1 | P0C0F2 | 6.13 | 118964.5 |
| DPO3A_UREPA_1 | Q9PQ74 | 7.01 | 112344.5 |
| DPO3A_MYCGE_1 | Q49405 | 8.57 | 100435.1 |
| DPO3A_MYCPN_1 | P75404 | 6.73 | 99257.45 |
| DPO3A_THEAQ_1 | Q9XDH5 | 5.56 | 137389.8 |
| DPO3A_THEMA_1 | Q9ZHG4 | 7.64 | 96499.54 |
| DPO3A_BACHD_1 | Q9K838 | 5.92 | 127531.9 |
| DPO3A_BUCAI_1 | P57332 | 8.96 | 132796.4 |
| DPO3A_PASMU_1 | Q9CPK3 | 5.26 | 129280.9 |
| DPO3A_HAEIN_1 | P43743 | 5.25 | 129788.7 |
| DPO3A_PSEFL_1 | Q9XDH6 | 5.28 | 131217.3 |
| DPO3A_RICBR_1 | Q1RKF9 | 5.61 | 131796.7 |
| DPO3A_RICFE_1 | Q4UK40 | 5.84 | 135675.1 |
| DPO3A_STRCO_1 | Q9Z618 | 5.55 | 130796.3 |
| DPO3A_BORBU_1 | O51526 | 6.15 | 130656.1 |
| DPO3A_YERPE_1 | O68770 | 5.73 | 130370.4 |
| DPO3A_MYCLE_1 | Q9X7F0 | 5.65 | 128972 |
| DPO3A_RICTY_1 | Q68VX1 | 6.72 | 133515.3 |
| DPO3A_AQUAE_1 | O67125 | 6.03 | 133207.7 |
| DPO3A_TREPA_1 | O83675 | 6.35 | 132313 |
| DNA Polymerase III subunit beta | Accession number | pI | Molecular Weight |
| DPO3B_ECOLI_1 | P0A988 | 5.25 | 40586.6 |
| DPO3B_MYCTU_1 | P9WNU1 | 4.76 | 42113.09 |
| DPO3B_CAUVN_1 | B8GXP6 | 4.95 | 40425.26 |
| DPO3B_MYCS2_1 | A0QND6 | 4.72 | 41324.96 |
| DPO3B_BACSU_1 | P05649 | 4.91 | 42103.25 |
| DPO3B_MYCGE_1 | P47247 | 5.83 | 44294.54 |
| DPO3B_PSEAE_1 | Q9I7C4 | 5.13 | 40694.49 |
| DPO3B_SYNE7_1 | P52023 | 4.9 | 40465.86 |
| DPO3B_LACLM_1 | O54376 | 5.16 | 42209.27 |
| DPO3B_HELPY_1 | O25242 | 5.51 | 42184.54 |
| DPO3B_CHLPN_1 | Q9Z8K0 | 4.95 | 40364.56 |
| DPO3B_BUCAP_1 | P29439 | 9.13 | 42180.33 |
| DPO3B_CHLMU_1 | Q9PKW4 | 5.07 | 40493.46 |
| DPO3B_MYCBO_1 | O33914 | 4.81 | 42055.05 |
| DPO3B_MYCLE_1 | P46387 | 4.85 | 41941.83 |
| DPO3B_RICCN_1 | Q92I37 | 5.26 | 42236.56 |
| DPO3B_STAAR_1 | Q6GKU3 | 4.66 | 41913.63 |
| DPO3B_STRPN_1 | O06672 | 5.04 | 42072.75 |
| DPO3B_PROMI_1 | P22838 | 5.19 | 40747.79 |
| DPO3B_RICPR_1 | Q9ZDB3 | 5.7 | 42968.89 |
| DPO3B_CHLTR_1 | O84078 | 5.92 | 46529.42 |
| DPO3B_AQUAE_1 | O67725 | 4.64 | 41300.14 |
| DPO3B_SYNY3_1 | P72856 | 4.7 | 42087.76 |
| DPO3B_SPICI_1 | P34029 | 7.6 | 41329.03 |
| DPO3B_BORBU_1 | P33761 | 5.42 | 44641.34 |
| DPO3B_TREPA_1 | O83048 | 5.79 | 41290.49 |
| DPO3B_MYCPU_1 | Q98RK6 | 7.66 | 42912.57 |
| DPO3B_PSEPU_1 | P0A121 | 5.16 | 40718.68 |
| DPO3B_RICBR_1 | Q1RIS7 | 4.96 | 41849.2 |
| DPO3B_MYCPA_1 | Q9L7L6 | 4.56 | 41570.13 |
| DPO3B_MYCCT_1 | P24117 | 4.96 | 42716.25 |
| DPO3B_RICFE_1 | Q4ULS3 | 5.26 | 42264.67 |
| DPO3B_STAEQ_1 | Q5HK00 | 4.62 | 42101.77 |
| DPO3B_MYCPN_1 | Q50313 | 5.8 | 43856.24 |
| DPO3B_SALTY_1 | P26464 | 5.25 | 40548.49 |
| DPO3B_HAEIN_1 | P43744 | 4.99 | 41631.21 |
| DPO3B_BACHD_1 | Q9RCA1 | 5.09 | 42382.45 |
| DPO3B_VIBCH_1 | Q9KVX5 | 5 | 40642.37 |
| DPO3B_RICTY_1 | Q68WW0 | 5.87 | 42821.77 |
| DPO3B_STRCO_1 | P27903 | 4.6 | 39956.39 |
| DNA Polymerase III subunit gamma | Accession number | pI | Molecular Weight |
| DPO3X_ECOLI_1 | P06710 | 6.39 | 71006.55 |
| DPO3X_SALTY_1 | P74876 | 6.5 | 70595.82 |
| DPO3X_MYCGE_1 | P47658 | 8.8 | 69043.2 |
| DPO3X_MYCTU_1 | P9WNT9 | 5.61 | 61923.37 |
| DPO3X_BACSU_1 | P09122 | 5.54 | 62763.04 |
| DPO3X_MYCBO_1 | P63976 | 5.61 | 61923.37 |
| DPO3X_BUCAP_1 | Q8K983 | 9.56 | 42185.9 |
| DPO3X_HAEIN_1 | P43746 | 5.81 | 77042.42 |
| DPO3X_MYCPN_1 | P75177 | 6.86 | 76212.59 |
| DNA Polymerase III subunit delta | Accession number | pI | Molecular Weight |
| HOLA_ECOLI_1 | P28630 | 6.51 | 38703.69 |
| HOLA_BUCAI_1 | P57520 | 9.7 | 40359.42 |
| HOLA_HAEIN_1 | P43747 | 6.16 | 39845.28 |
| DNA Polymerase III subunit delta' | Accession number | pI | Molecular Weight |
| HOLB_ECOLI_1 | P28631 | 6.56 | 36936.56 |
| HOLB_BACSU_1 | P37540 | 6.32 | 37621.47 |
| HOLB_YERPE_1 | O69170 | 6.88 | 38114.03 |
| HOLB_BUCAI_1 | P57435 | 9.39 | 38452.96 |
| HOLB_PSEAE_1 | P52024 | 6.19 | 35746.36 |
| HOLB_HAEIN_1 | P43748 | 5.48 | 37038.51 |
| DNA Polymerase III subunit epsilon | Accession number | pI | Molecular Weight |
| DPO3E_ECOLI_1 | P03007 | 5.54 | 27098.93 |
| DPO3E_SALTY_1 | P0A1G9 | 5.68 | 27225.08 |
| DPO3E_BUCAP_1 | Q08880 | 9.81 | 27169.79 |
| DPO3E_HAEIN_1 | P43745 | 4.97 | 29133.45 |
| DPO3E_AQUAE_1 | O67074 | 8.95 | 23044.91 |
| DPO3E_SALTI_1 | P0A1H0 | 5.68 | 27225.08 |
| DPO3E_RICPR_1 | Q9ZCJ9 | 8.73 | 26078.43 |
| DPO3E_RICCN_1 | Q92GL1 | 7.85 | 26216.32 |
| DPO3E_RICBR_1 | Q1RJM1 | 8.52 | 26078.21 |
| DPO3E_RICTY_1 | Q68W16 | 8.65 | 26118.51 |
| DPO3E_PASMU_1 | Q9CPE0 | 5.06 | 28559.63 |
| DPO3E_RICFE_1 | Q4UN31 | 6.72 | 25943.89 |
| DPO3E_TREPA_1 | O83649 | 6.16 | 24238.75 |
| DNA Polymerase III subunit psi | Accession number | pI | Molecular Weight |
| HOLD_ECOLI_1 | P28632 | 5.48 | 15174.35 |
| HOLD_HAEIN_1 | P43750 | 5.65 | 15717.91 |
| DNA Polymerase III subunit chi | Accession number | pI | Molecular Weight |
| HOLC_ECOLI_1 | P28905 | 5.91 | 16632.78 |
| HOLC_PSEAE_1 | O68823 | 5.57 | 16141.31 |
| HOLC_HAEIN_1 | P43749 | 5.23 | 16635.83 |
| DNA Polymerase III subunit theta | Accession number | pI | Molecular Weight |
| HOLE_ECOLI_1 | P0ABS8 | 9.15 | 8846.29 |
| HOLE_SHIFL_1 | P0ABT1 | 9.15 | 8846.29 |
| DNA polymerase I | Accession number | pI | Molecular Weight |
| DPO1_ECOLI_1 | P00582 | 5.4 | 103118.1 |
| DPO1_GEOSE_1 | P52026 | 5.65 | 98670.31 |
| DPO1_MYCTU_1 | P9WNU5 | 5.01 | 98472.04 |
| DPO1_DEIRA_1 | P52027 | 5.17 | 105660.1 |
| DPO1_SALTY_1 | Q9F173 | 5.49 | 103129.2 |
| DPO1_STRR6_1 | P59200 | 4.96 | 99163.63 |
| DPO1_CHLAA_1 | O08307 | 5.19 | 103698.3 |
| DPO1_HAEIN_1 | P43741 | 5.37 | 103740.6 |
| DPO1_HELPY_1 | P56105 | 8.53 | 101975.5 |
| DPO1_BACCA_1 | Q04957 | 5.49 | 99475.19 |
| DPO1_MYCLE_1 | P46835 | 5.1 | 99792.1 |
| DPO1_MYCBO_1 | P0A551 | 5.01 | 98472.04 |
| DPO1_BACSU_1 | O34996 | 5.1 | 99092.06 |
| DPO1_PSEAE_1 | Q9HT80 | 5 | 99792.93 |
| DPO1_SYNY3_1 | Q55971 | 5.26 | 110287 |
| DPO1_THEFI_1 | O52225 | 5.81 | 93890.9 |
| DPO1_THECA_1 | P80194 | 6.11 | 93799.07 |
| DPO1_RHILE_1 | Q9S1G2 | 5.42 | 111492.5 |
| DPO1_LACLA_1 | Q9CDS1 | 5.1 | 98732.96 |
| DPO1T_THET8_1 | P52028 | 6.23 | 94006.39 |
| DPO1_THEAQ_1 | P19821 | 6.03 | 93910.16 |
| DPO1_RICFE_1 | Q9RAA9 | 5.95 | 104005.8 |
| DPO1_RICBR_1 | Q1RH76 | 5.9 | 98632.42 |
| DPO1_RICHE_1 | Q9RLB6 | 6.23 | 104450.5 |
| DPO1_AQUAE_1 | O67779 | 7.65 | 65833 |
| DPO1_BORBU_1 | O51498 | 6.46 | 105503.7 |
| DPO1_RICCN_1 | Q92GB7 | 6.82 | 99029.71 |
| DPO1_TREPA_1 | P74933 | 6.36 | 112217.4 |
| DPO1_CALBD_1 | Q59156 | 5.47 | 98130.1 |
| DPO1_RICPR_1 | O05949 | 9.29 | 98652.03 |
| DPO1_RICTY_1 | Q9RLA0 | 9.13 | 99309.66 |
| DNA Ligase | Accession number | pI | Molecular Weight |
| DNLJ_MYCTU_1 | P9WNV1 | 5.42 | 75289.16 |
| DNLJ_ECOLI_1 | P15042 | 5.39 | 73606.07 |
| DNLJ_THEFI_1 | Q9ZHI0 | 6.12 | 76593.88 |
| DNLJ_THET8_1 | P26996 | 6.66 | 76905.47 |
| DNLJ_MYCS2_1 | A0QUW7 | 5.09 | 76268.94 |
| DNLJ_AQUAE_1 | O66880 | 8.81 | 82318.35 |
| DNLJ_VIBC3_1 | A5F2W3 | 5.44 | 73371.16 |
| DNLJ_ENTFA_1 | Q837V6 | 5 | 75583.78 |
| DNLJ_HAEIN_1 | P43813 | 5.5 | 74042.53 |
| DNLJ_RHOMR_1 | P49421 | 5.77 | 79487.86 |
| DNLJ_STAAU_1 | Q9AIU7 | 5.13 | 75081 |
| DNLJ_HELPJ_1 | Q9ZLM1 | 6.53 | 74156.16 |
| DNLJ_BACSU_1 | O31498 | 4.9 | 74876.1 |
| DNLJ_DEIRA_1 | Q9RSQ5 | 5.12 | 75570.13 |
| DNLJ_ZYMMO_1 | P28719 | 5.75 | 82190.5 |
| DNLJ_BACVZ_1 | A7Z261 | 4.93 | 75236.58 |
| DNLJ_SYNY3_1 | P72588 | 5.46 | 74602.23 |
| DNLJ2_STRCO_1 | Q93IZ8 | 5.28 | 73776.72 |
| DNLJ_THESC_1 | P49422 | 6.27 | 76525.84 |
| DNLJ_LEGPH_1 | Q5ZWX6 | 5.97 | 74648.66 |
| DNLJ_SYNFM_1 | A0LI67 | 6.12 | 74172.41 |
| DNLJ_RICFE_1 | Q4UN15 | 5.55 | 77633.59 |
| DNLJ_DESAG_1 | Q30ZK6 | 5.51 | 78051.8 |
| DNLJ_LEPBL_1 | Q04XH0 | 8.46 | 77025.42 |
| DNLJ_PETMO_1 | A9BJX5 | 6.03 | 75882.16 |
| DNLJ_PROM3_1 | A2CDS6 | 6.15 | 75582.08 |
| DNLJ_PSE14_1 | Q48KR2 | 5.5 | 86348.62 |
| DNLJ_PSEA7_1 | A6V7X3 | 5.25 | 86747.49 |
| DNLJ_PSEMY_1 | A4XVY5 | 5.2 | 85535.29 |
| DNLJ_RALSO_1 | Q8XZJ7 | 5.5 | 87470.52 |
| DNLJ_RENSM_1 | A9WTZ3 | 5.04 | 82589.35 |
| DNLJ_RHILW_1 | B5ZWH9 | 5.5 | 79214.61 |
| DNLJ_RHOOB_1 | C1B1S4 | 4.97 | 75696.93 |
| DNLJ_RHOP5_1 | Q07PR9 | 5.54 | 77981.55 |
| DNLJ_SALAI_1 | A8M5E3 | 5.24 | 76662.6 |
| DNLJ_SALNS_1 | B4SZU5 | 5.53 | 73453.12 |
| DNLJ_SALRD_1 | Q2S459 | 4.59 | 76246.88 |
| DNLJ_SALTI_1 | Q8Z4W4 | 5.52 | 73439.09 |
| DNLJ_SHEB9_1 | A9L5Z1 | 5.37 | 74396.13 |
| DNLJ_SHEON_1 | Q8ED70 | 5.36 | 75338.21 |
| DNLJ_SHEPC_1 | A4Y802 | 5.44 | 74622.43 |
| DNLJ_THEMA_1 | Q9WXV5 | 7.97 | 78906.3 |
| DNLJ_THEP3_1 | B0KBN6 | 5.92 | 75031.64 |
| DNLJ_THERP_1 | B9KXM2 | 5.76 | 77976.05 |
| DNLJ_THESQ_1 | B1LA51 | 6.8 | 78807.11 |
| DNLJ_TREDE_1 | Q73MH0 | 5.89 | 71747.73 |
| DNLJ_VESOH_1 | A5CXB8 | 7.11 | 71178.76 |
| DNLJ_WOLSU_1 | Q7M8A0 | 5.67 | 72896.47 |
| DNLJ_WOLTR_1 | Q5GS88 | 7.11 | 76527.55 |
| DNLJ_XANC8_1 | Q4UTB0 | 5.39 | 90347.74 |
| DNLJ_YERP3_1 | A7FGC2 | 5.42 | 73953.44 |
| DNLJ_CAMLR_1 | B9KG17 | 5.47 | 74049 |
| DNLJ_CARHZ_1 | Q3AD38 | 5.75 | 74177.2 |
| DNLJ_CHESB_1 | Q11GT5 | 5.47 | 77412.99 |
| DNLJ_CHLAD_1 | B8GAC1 | 5.77 | 74710.2 |
| DNLJ_CHLPM_1 | A4SG85 | 5.49 | 74840.2 |
| DNLJ_CUPMC_1 | Q1LNG5 | 5.23 | 77305.68 |
| DNLJ_CYAP8_1 | B7K0A2 | 5.66 | 75077.72 |
| DNLJ_DEHM1_1 | Q3Z8W1 | 5.84 | 74965.32 |
| DNLJ_DESPS_1 | Q6API9 | 5.76 | 75363.5 |
| DNLJ_ESCF3_1 | B7LL73 | 5.39 | 73595.04 |
| DNLJ_EXIS2_1 | B1YJ17 | 4.88 | 73665.29 |
| DNLJ_FINM2_1 | B0RZW0 | 5.09 | 75905.16 |
| DNLJ_FLAPJ_1 | A6H0N9 | 5.54 | 75008.13 |
| DNLJ_FRACC_1 | Q2J6U3 | 5.42 | 75836.89 |
| DNLJ_FRATW_1 | A4IWW8 | 5.85 | 76454.52 |
| DNLJ_GLUOX_1 | Q5FUI5 | 5.23 | 75381.02 |
| DNLJ_GRABC_1 | Q0BV35 | 5.46 | 76035.23 |
| DNLJ_GRAFK_1 | A0LZ23 | 5.11 | 75218.91 |
| DNLJ_HAES1_1 | Q0I2H6 | 6.81 | 74559.76 |
| DNLJ_LEPBA_1 | B0SA75 | 8.15 | 75387.73 |
| DNLJ_PHEZH_1 | B4RFE9 | 5.41 | 75989.09 |
| DNLJ_POLSJ_1 | Q12AD4 | 5.98 | 76985.78 |
| DNLJ_PORGI_1 | Q7MV47 | 5.3 | 75085.32 |
| DNLJ_RHILO_1 | Q98KC4 | 5.38 | 80495.85 |
| DNLJ_RICRO_1 | B0BUZ1 | 5.55 | 77850.88 |
| DNLJ_RUEPO_1 | Q5LST6 | 5.21 | 79825.81 |
| DNLJ_RUEST_1 | Q1GGT5 | 5.23 | 79736.56 |
| DNLJ_SALCH_1 | Q57LT1 | 5.48 | 73540.24 |
| DNLJ_SHEDO_1 | Q12L82 | 5.71 | 72949.61 |
| DNLJ_SHEFN_1 | Q080K9 | 5.59 | 73466.93 |
| DNLJ_SHEHH_1 | B0TKB2 | 5.37 | 72973.29 |
| DNLJ_SORC5_1 | A9F3W3 | 6.36 | 72419.91 |
| DNLJ_STRP6_1 | Q5XCY8 | 5.31 | 72428.42 |
| DNLJ_STRP7_1 | C1C7B1 | 4.99 | 72279.88 |
| DNLJ_STRSY_1 | A4VVK7 | 4.84 | 71559.21 |
| DNLJ_SULAA_1 | C1DW63 | 8.7 | 79860.52 |
| DNLJ_SULNB_1 | A6Q7G2 | 5.26 | 73353.37 |
| DNLJ_SYNAS_1 | Q2LTN8 | 8.48 | 74508.57 |
| DNLJ_THENN_1 | B9K734 | 8.4 | 79428.71 |
| DNLJ_UNCTG_1 | B1H072 | 8.93 | 76317.65 |
| DNLJ_GEOLS_1 | B3E8F8 | 5.21 | 74777.31 |
| DNLJ_HELHP_1 | Q7VF74 | 5.57 | 73553.62 |
| DNLJ_JANSC_1 | Q28PE1 | 5.18 | 80820.47 |
| DNLJ_KOCRD_1 | B2GL64 | 4.91 | 83591.05 |
| DNLJ_LACCB_1 | B3WD54 | 5.52 | 73640.15 |
| DNLJ_LACDB_1 | Q04BW0 | 5.16 | 74279.37 |
| DNLJ_LACS1_1 | Q1WSH6 | 5.15 | 75169.45 |
| DNLJ_LEIXX_1 | Q6AEE5 | 5.24 | 80467.19 |
| DNLJ_POLNA_1 | A1VN98 | 5.73 | 77203.1 |
| DNLJ_PSEA6_1 | Q15UZ1 | 5.17 | 74235.64 |
| DNLJ_PSEPK_1 | Q88F25 | 5.43 | 84112.92 |
| DNLJ_RHIEC_1 | Q2K6D3 | 5.52 | 79151.64 |
| DNLJ_RICB8_1 | A8GXT8 | 5.45 | 77640.6 |
| DNLJ_RICM5_1 | A8F2K5 | 5.55 | 77711.74 |
| DNLJ_RICTY_1 | Q68W27 | 8.48 | 78483.48 |
| DNLJ_ROSCS_1 | A7NHP6 | 5.74 | 77952.65 |
| DNLJ_SACD2_1 | Q21JI8 | 5.09 | 73568.79 |
| DNLJ_SACEN_1 | A4FMS5 | 4.95 | 79743.51 |
| DNLJ_SALHS_1 | B4TCF6 | 5.53 | 73442.09 |
| DNLJ_XANAC_1 | Q8PM07 | 5.57 | 90483.94 |
| DNLJ_XYLFA_1 | Q9PAG2 | 5.86 | 91496.56 |
| DNLJ_PSEFS_1 | C3JXX6 | 5.55 | 85568.58 |
| DNLJ_PSEHT_1 | Q3IKB8 | 5.27 | 73855.95 |
| DNLJ_PSEU5_1 | A4VKN0 | 5.5 | 86098.93 |
| DNLJ_PSYIN_1 | A1SX61 | 5.54 | 71531.54 |
| DNLJ_RHOFT_1 | Q21WC8 | 5.7 | 75199.42 |
| DNLJ_RICAH_1 | A8GPM9 | 5.76 | 77420.68 |
| DNLJ_RICPR_1 | Q9ZCK9 | 7.56 | 77911.44 |
| DNLJ_SALAR_1 | A9MIG0 | 5.48 | 73466.12 |
| DNLJ_SALG2_1 | B5RCP9 | 5.47 | 73414.03 |
| DNLJ_SALPA_1 | Q5PNE2 | 5.52 | 73476.15 |
| DNLJ_SHESA_1 | A0KVI4 | 5.4 | 75120.02 |
| DNLJ_SINMW_1 | A6UB73 | 5.55 | 79295.89 |
| DNLJ_STACT_1 | B9DMU3 | 5.44 | 75530.72 |
| DNLJ_STRA5_1 | Q8E084 | 5.37 | 72579.57 |
| DNLJ_STRAW_1 | Q82JK7 | 5.17 | 80422.9 |
| DNLJ_STRE4_1 | C0M6L2 | 5.07 | 72092.12 |
| DNLJ_STRMU_1 | Q8DT49 | 5.53 | 71842.31 |
| DNLJ_SYNE7_1 | Q31RL0 | 5.31 | 74311.95 |
| DNLJ_SYNJA_1 | Q2JW63 | 6.11 | 74347.27 |
| DNLJ_THEAB_1 | B7IDX6 | 8.28 | 75662.76 |
| DNLJ_THEYD_1 | B5YIF8 | 7.58 | 76154.81 |
| DNLJ_UREPA_1 | Q9PR23 | 5.88 | 77450.52 |
| DNLJ_VIBVU_1 | Q8DFK5 | 5.35 | 73535.18 |
| DNLJ_WOLPM_1 | Q73H01 | 5.62 | 74347.75 |
| DNLJ_XANOM_1 | Q2P334 | 5.66 | 92259.21 |
| DNLJ_PROMH_1 | B4EZR2 | 5.52 | 74720.48 |
| DNLJ_PSESM_1 | Q87YY6 | 5.44 | 86312.53 |
| DNLJ_PSYA2_1 | Q4FUW9 | 5.04 | 76019.5 |
| DNLJ_SOLUE_1 | Q01NV7 | 6.4 | 73842.94 |
| DNLJ_SPHAL_1 | Q1GVQ2 | 5.32 | 77528.92 |
| DNLJ_STAES_1 | Q8CRU0 | 5.34 | 75109.32 |
| DNLJ_STRSV_1 | A3CNX3 | 4.99 | 72173.93 |
| DNLJ_STRT2_1 | Q5M382 | 5.05 | 71943.63 |
| DNLJ_SULSY_1 | B2V5U1 | 8.23 | 80267.99 |
| DNLJ_XANP2_1 | A7IG64 | 5.5 | 77769.04 |
| DNLJ_SPHWW_1 | A5VFJ8 | 5.27 | 76284.25 |
| DNLJ_STRU0_1 | B9DRS2 | 5.12 | 72600.47 |
| DNLJ_SULDN_1 | Q30QV9 | 5.1 | 73423.1 |
| DNLJ_SYNWW_1 | Q0AZZ5 | 5.5 | 73988.69 |
| DNLJ_THEM4_1 | A6LN55 | 9.19 | 75476.83 |
| DNLJ_THEP1_1 | A5IKX0 | 6.8 | 78807.11 |
| DNLJ_VEREI_1 | A1WRA7 | 8.11 | 75032.78 |
| DNLJ_VIBPA_1 | Q87RJ4 | 5.27 | 73988.22 |
| DNLJ_VIBTL_1 | B7VIK3 | 5.04 | 73180.54 |
| DNLJ_WIGBR_1 | Q8D2B9 | 9.83 | 68483.88 |
| DNLJ_YERPA_1 | Q1C5X9 | 5.52 | 73898.37 |
| DNLJ_RALPJ_1 | B2UB00 | 5.36 | 88733.86 |
| DNLJ_RHOBA_1 | Q7UKD1 | 5.05 | 78277.54 |
| DNLJ_RHOE4_1 | C0ZXK3 | 4.99 | 76277.12 |
| DNLJ_RHOS5_1 | A4WRP6 | 5.66 | 76569.19 |
| DNLJ_RICAE_1 | C3PLJ2 | 5.66 | 77711.77 |
| DNLJ_SALA4_1 | B5F0F5 | 5.48 | 73498.15 |
| DNLJ_SHEAM_1 | A1S5R7 | 5.29 | 73671.4 |
| DNLJ_SHEWM_1 | B1KJT5 | 5.08 | 73316.42 |
| DNLJ_SHIBS_1 | Q31Y68 | 5.31 | 73623.11 |
| DNLJ_SHIFL_1 | Q83K81 | 5.31 | 73623.11 |
| DNLJ_SHISS_1 | Q3YZD1 | 5.35 | 73692.16 |
| DNLJ_SPICI_1 | Q14L37 | 5.97 | 76389.1 |
| DNLJ_STAS1_1 | Q49YU8 | 5.14 | 75238.13 |
| DNLJ_THEFY_1 | Q47SC6 | 5.32 | 82089.09 |
| DNLJ_THISH_1 | B8GTL0 | 5.41 | 74048.04 |
| DNLJ_TREPS_1 | B2S3M4 | 8.54 | 91098.3 |
| DNLJ_TRIEI_1 | Q112N5 | 6.22 | 86086.67 |
| DNLJ_TROW8_1 | Q83HX4 | 6.08 | 70186.29 |
| DNLJ_VIBCB_1 | A7MT54 | 5.15 | 73921.3 |
| DNLJ_POLAQ_1 | A4SYV5 | 5.45 | 73743.02 |
| DNLJ_PSEE4_1 | Q1ICK0 | 5.28 | 85515.42 |
| DNLJ_CELJU_1 | B3PGQ8 | 5.16 | 74195.36 |
| DNLJ_CHLAA_1 | A9WCA1 | 5.48 | 76790.6 |
| DNLJ_CHLL2_1 | B3EFT6 | 5.69 | 75698.38 |
| DNLJ_CHLT2_1 | B0B9Q4 | 6.61 | 73497.53 |
| DNLJ_CHLT3_1 | B3QSH0 | 6.04 | 75267.33 |
| DNLJ_CLAM3_1 | A5CQU5 | 4.89 | 90323.61 |
| DNLJ_CLOB1_1 | A7FYL7 | 5.42 | 75846.84 |
| DNLJ_CLOCE_1 | B8I3I1 | 5.17 | 74137.6 |
| DNLJ_CLONN_1 | A0Q2Q3 | 5.21 | 74755.53 |
| DNLJ_CORDI_1 | Q6NHQ4 | 5.15 | 74766.05 |
| DNLJ_CROS8_1 | A7MKW4 | 5.5 | 73775.52 |
| DNLJ_CUPTR_1 | B3R2C2 | 5.46 | 76253.8 |
| DNLJ_CYTH3_1 | Q11VI1 | 5.62 | 74930.77 |
| DNLJ_DESVH_1 | Q72BM7 | 5 | 86099.13 |
| DNLJ_EHRRG_1 | Q5FG20 | 7.88 | 76732.46 |
| DNLJ_GEMAT_1 | C1A6H2 | 5.28 | 74173.62 |
| DNLJ_GEODF_1 | B9M861 | 5.69 | 74843.9 |
| DNLJ_GEOKA_1 | Q5L3B9 | 5.22 | 74642.9 |
| DNLJ_GLOVI_1 | Q7NMN8 | 5.98 | 73224.22 |
| DNLJ_GLUDA_1 | A9H0L6 | 5.5 | 75946.01 |
| DNLJ_HAEDU_1 | Q7VMX7 | 6.32 | 76288.64 |
| DNLJ_HAHCH_1 | Q2SD47 | 5.16 | 75318.4 |
| DNLJ_HERA2_1 | A9AXP4 | 5.4 | 73337.26 |
| DNLJ_HERAR_1 | A4G4R4 | 5.54 | 75648.12 |
| DNLJ_HISS2_1 | B0USD9 | 6.81 | 73897.95 |
| DNLJ_HYDS0_1 | B4U8X0 | 8.11 | 79140.24 |
| DNLJ_IDILO_1 | Q5QUK1 | 5.31 | 74075.61 |
| DNLJ_LACAC_1 | Q5FLL4 | 5.01 | 74361.41 |
| DNLJ_LACBA_1 | Q03Q13 | 5 | 73459.19 |
| DNLJ_LACF3_1 | B2GDS9 | 5.05 | 75038.14 |
| DNLJ_LACGA_1 | Q041K2 | 5.28 | 74692.84 |
| DNLJ_LACH4_1 | A8YTZ3 | 5.04 | 74323.29 |
| DNLJ_LACJO_1 | Q74I49 | 5.29 | 74607.78 |
| DNLJ_LACRJ_1 | B2G8T9 | 5.12 | 76002.91 |
| DNLJ_POLNS_1 | B1XTU0 | 5.6 | 73724.19 |
| DNLJ_PROA2_1 | B4S4Y6 | 5.77 | 75377.77 |
| DNLJ_PSYCK_1 | Q1QDW8 | 5.19 | 75896.61 |
| DNLJ_RHIME_1 | Q92NM8 | 5.6 | 78968.47 |
| DNLJ_ROSS1_1 | A5V1U3 | 6.06 | 78636.47 |
| DNLJ_RUTMC_1 | A1AVV5 | 6.17 | 68937.62 |
| DNLJ_SALEP_1 | B5R3V4 | 5.53 | 73428.06 |
| DNLJ_SALSV_1 | B4TQF8 | 5.46 | 73509.13 |
| DNLJ_SHEPW_1 | B8CN91 | 5.17 | 72967.27 |
| DNLJ_SHIDS_1 | Q32DE2 | 5.34 | 73580.07 |
| DNLJ_SINFN_1 | C3MEL9 | 5.63 | 79267.84 |
| DNLJ_STAHJ_1 | Q4L7L9 | 5.1 | 75260.22 |
| DNLJ_STRMK_1 | B2FKJ1 | 5.35 | 88821.58 |
| DNLJ_SYMTH_1 | Q67KI8 | 5.92 | 74734.15 |
| DNLJ_THEPX_1 | B0K3S1 | 5.92 | 74985.58 |
| DNLJ_THESK_1 | Q9ZFY8 | 6.04 | 76588.9 |
| DNLJ_THIDA_1 | Q3SL40 | 6.16 | 74111.33 |
| DNLJ_YERE8_1 | A1JLA4 | 5.48 | 74041.63 |
| DNLJ_DINSH_1 | A8LK52 | 5.1 | 79936.01 |
| DNLJ_ERWT9_1 | B2VE40 | 5.38 | 73388.88 |
| DNLJ_FLAJ1_1 | A5FE87 | 5.37 | 75661.78 |
| DNLJ_FRAP2_1 | B0TY02 | 5.38 | 76359.06 |
| DNLJ_GEOSE_1 | O87703 | 5.32 | 74230.55 |
| DNLJ_GEOTN_1 | A4IJY6 | 5.35 | 74524.79 |
| DNLJ_HALOH_1 | B8D122 | 5.81 | 74872.49 |
| DNLJ_LACSS_1 | Q38VC5 | 4.92 | 74135.17 |
| DNLJ_PSYWF_1 | A5WCN7 | 5.09 | 75543.6 |
| DNLJ_RHORT_1 | Q2RVV5 | 5.46 | 75625.29 |
| DNLJ_RICCK_1 | A8EZT5 | 6.49 | 78019.25 |
| DNLJ_RICCN_1 | Q92GM7 | 5.61 | 77837.97 |
| DNLJ_RICPU_1 | C4K0T1 | 5.58 | 77790.88 |
| DNLJ_ROSDO_1 | Q166E0 | 5.36 | 76411.09 |
| DNLJ_RUBXD_1 | Q1AZ75 | 5.49 | 76996.16 |
| DNLJ_SALDC_1 | B5FQB9 | 5.53 | 73470.14 |
| DNLJ_SALTO_1 | A4X487 | 5.4 | 76364.25 |
| DNLJ_SALTY_1 | Q8ZN89 | 5.52 | 73452.13 |
| DNLJ_SERP5_1 | A8GHF2 | 5.27 | 74139.6 |
| DNLJ_CAUSK_1 | B0T1N5 | 5.36 | 85801.04 |
| DNLJ_CAUVN_1 | B8H5Y7 | 5.22 | 84901.07 |
| DNLJ_CHLAB_1 | Q5L5Q2 | 6.36 | 74175.24 |
| DNLJ_CHLCH_1 | Q3ATX4 | 5.79 | 75313.98 |
| DNLJ_CHLL7_1 | Q3B278 | 5.67 | 75818.4 |
| DNLJ_CHLMU_1 | Q9PKP2 | 6.91 | 73785.11 |
| DNLJ_CHLP8_1 | B3QLI5 | 5.62 | 74449.87 |
| DNLJ_CHRSD_1 | Q1QZP4 | 5.09 | 75767.3 |
| DNLJ_CLOP1_1 | Q0TN59 | 5.18 | 74963.68 |
| DNLJ_CLOTE_1 | Q891I0 | 5.32 | 76198.32 |
| DNLJ_CLOTH_1 | A3DE88 | 5.77 | 74841.65 |
| DNLJ_COLP3_1 | Q47YI0 | 5.8 | 75223 |
| DNLJ_COPPD_1 | B5Y6V5 | 5.79 | 74775.75 |
| DNLJ_CORGL_1 | Q8NR20 | 4.87 | 74706.06 |
| DNLJ_COXB1_1 | B6J878 | 9.15 | 75629.59 |
| DNLJ_CUPNH_1 | Q0KA11 | 5.24 | 76255.68 |
| DNLJ_CUTAK_1 | Q6A7A2 | 5.17 | 84449.17 |
| DNLJ_DECAR_1 | Q47FA0 | 5.37 | 73792.35 |
| DNLJ_DELAS_1 | A9BZW4 | 6.32 | 75728.55 |
| DNLJ_DESAA_1 | B8FKD5 | 5.15 | 69522.82 |
| DNLJ_DESAH_1 | C0QJ86 | 6.29 | 75284.27 |
| DNLJ_DESHY_1 | Q24QL5 | 5.22 | 72979.18 |
| DNLJ_DESOH_1 | A9A0L9 | 5.6 | 74242.49 |
| DNLJ_EHRCJ_1 | Q3YRC1 | 7.63 | 76854.37 |
| DNLJ_ENT38_1 | A4WD23 | 5.42 | 73591.14 |
| DNLJ_FRASN_1 | A8L538 | 5.3 | 75716.63 |
| DNLJ_FUSNN_1 | Q8RI94 | 5.56 | 79396.22 |
| DNLJ_GEOMG_1 | Q39S28 | 5.47 | 74224.65 |
| DNLJ_GEOSL_1 | Q74ER9 | 5.53 | 74576.22 |
| DNLJ_HALHL_1 | A1WY80 | 5.23 | 76887.9 |
| DNLJ_HELAH_1 | Q17XL1 | 7 | 74018.16 |
| DNLJ_HELMI_1 | B0TDL0 | 5.66 | 74672.28 |
| DNLJ_HYDCU_1 | Q31G89 | 5.17 | 75407.84 |
| DNLJ_HYPNA_1 | Q0C577 | 5.18 | 75959.02 |
| DNLJ_JANMA_1 | A6SZQ8 | 5.46 | 75144.54 |
| DNLJ_KINRD_1 | A6W7L4 | 5.1 | 75120.41 |
| DNLJ_KLEP3_1 | B5XVT4 | 5.41 | 73630.05 |
| DNLJ_KORVE_1 | Q1IHJ4 | 6.59 | 74925.79 |
| DNLJ_LACLS_1 | Q031P9 | 5.33 | 76824.62 |
| DNLJ_LACPL_1 | Q88XQ0 | 5.08 | 74521.5 |
| DNLJ_RHOCS_1 | B6IRF1 | 5.52 | 78136.57 |
| DNLJ_RHOJR_1 | Q0S2J2 | 4.95 | 75729.92 |
| DNLJ_CLOB8_1 | A6LQB0 | 5.07 | 74656.11 |
| DNLJ_CORA7_1 | C3PFU5 | 4.87 | 74635.81 |
| DNLJ_COREF_1 | Q8FPZ7 | 4.93 | 101222.8 |
| DNLJ_CORU7_1 | B1VG01 | 4.97 | 77277.14 |
| DNLJ_DEIGD_1 | Q1J0I6 | 5.58 | 74532.35 |
| DNLJ_DESAP_1 | B1I551 | 6.53 | 74579.12 |
| DNLJ_DESDA_1 | B8J0Q0 | 5.56 | 77624.27 |
| DNLJ_DICNV_1 | A5EVZ5 | 6.59 | 74232.85 |
| DNLJ_HAEPS_1 | B8F314 | 5.35 | 74701.12 |
| DNLJ_HAMD5_1 | C4K4K4 | 8.63 | 78090.9 |
| DNLJ_LACP3_1 | Q03AC3 | 5.52 | 73626.12 |
| DNLJ_LACP7_1 | A9KPL0 | 5.14 | 73434.02 |
| DNLJ_LARHH_1 | C1D4R5 | 5.69 | 73986.05 |
| DNLJ_PHYMT_1 | B3QZS5 | 9.49 | 76485.33 |
| DNLJ_CHLFF_1 | Q254X9 | 6.2 | 74548.55 |
| DNLJ_CHLPB_1 | B3EM49 | 6.4 | 76146.9 |
| DNLJ_CITK8_1 | A8ADI2 | 5.29 | 73443 |
| DNLJ_CLOK1_1 | B9DWM9 | 5.69 | 74994.88 |
| DNLJ_CORJK_1 | Q4JUL8 | 5.06 | 75610.59 |
| DNLJ_DESRM_1 | A4J703 | 6.12 | 74379.93 |
| DNLJ_EHRCR_1 | Q2GHG1 | 7.57 | 76930.41 |
| DNLJ_ELUMP_1 | B2KE31 | 7.18 | 72029.25 |
| DNLJ_FERNB_1 | A7HJG6 | 5.93 | 77872.62 |
| DNLJ_FRAAA_1 | Q0RDH8 | 5.17 | 81426.5 |
| DNLJ_GEOBB_1 | B5EIJ0 | 5.33 | 74326.9 |
| DNLJ_GEOUR_1 | A5GA98 | 5.65 | 75073.68 |
| DNLJ_CHLCV_1 | Q822R2 | 5.78 | 74056.15 |
| DNLJ_CHLPN_1 | Q9Z934 | 5.98 | 73909.65 |
| DNLJ_CHLTE_1 | Q8KF74 | 5.59 | 74783.62 |
| DNLJ_CHRVO_1 | Q7NR81 | 5.26 | 88505.86 |
| DNLJ_BORPA_1 | Q7W0T4 | 5.91 | 75503.1 |
| DNLJ1_OPITP_1 | B1ZWH2 | 5.99 | 74233.63 |
| DNLJ_AZOPC_1 | B6YRR9 | 8.91 | 75825 |
| DNLJ2_CLOAB_1 | Q97FQ5 | 5.38 | 75900.5 |
| DNLJ_BACAH_1 | A0R906 | 5.1 | 75152.21 |
| DNLJ_BRUAB_1 | Q57C89 | 5.59 | 78681.29 |
| DNLJ_BRUC2_1 | A9M679 | 5.59 | 78681.29 |
| DNLJ_ACIAD_1 | Q6FDW0 | 5.41 | 75659.21 |
| DNLJ_BACCN_1 | A7GKJ1 | 5.36 | 74934.22 |
| DNLJ_BRUSI_1 | B0CHK8 | 5.59 | 78681.29 |
| DNLJ_BACCR_1 | Q81IP2 | 5.1 | 75199.31 |
| DNLJ_ACIC1_1 | A0LSQ7 | 6.05 | 75299.62 |
| DNLJ_ACIF5_1 | B5EP61 | 6.36 | 73917.15 |
| DNLJ_ACTP7_1 | B3H275 | 5.25 | 75718.29 |
| DNLJ_BARBK_1 | A1UTB5 | 6.36 | 80394.94 |
| DNLJ_BARQU_1 | Q6FZ81 | 6.25 | 80127.78 |
| DNLJ_AGRRK_1 | B9JH39 | 5.28 | 78353.58 |
| DNLJ_BDEBA_1 | Q6MRL9 | 5.84 | 76371.41 |
| DNLJ_BEII9_1 | B2IGH4 | 5.62 | 76839.14 |
| DNLJ_BIFA0_1 | B8DU32 | 5.05 | 95952.81 |
| DNLJ_BLOPB_1 | Q492G5 | 9.33 | 67381.52 |
| DNLJ_ANAD2_1 | B8JD56 | 6.37 | 76420.76 |
| DNLJ_LEPCP_1 | B1Y6D4 | 5.44 | 82237.33 |
| DNLJ_ANAMM_1 | Q5PBN8 | 6.66 | 74628.04 |
| DNLJ_LEPIN_1 | Q8EYU4 | 6.83 | 76912.2 |
| DNLJ_MYCSJ_1 | A3PXM2 | 5.19 | 75898.56 |
| DNLJ_BORPD_1 | A9II69 | 6.08 | 75215.44 |
| DNLJ_ANASK_1 | B4UDH6 | 6.33 | 76329.65 |
| DNLJ_AZOSB_1 | A1K713 | 5.53 | 74906.99 |
| DNLJ_BACAC_1 | C3L543 | 5.12 | 75110.17 |
| DNLJ_ACHLI_1 | A9NHW0 | 5.66 | 74008.77 |
| DNLJ_ALISL_1 | B6EJQ6 | 5.06 | 73676.15 |
| DNLJ_BLOFL_1 | Q7VRU2 | 9.47 | 67657.14 |
| DNLJ_BORAP_1 | Q0SMV7 | 9.27 | 75434.99 |
| DNLJ_BORBR_1 | Q7WCJ7 | 5.98 | 75556.15 |
| DNLJ_BORBU_1 | O51502 | 9.01 | 75316.51 |
| DNLJ_MYCUA_1 | A0PPW9 | 5.3 | 74625.24 |
| DNLJ_MYXXD_1 | Q1D0P7 | 6.11 | 74045.46 |
| DNLJ_NAUPA_1 | B9L8R4 | 6.05 | 75065.81 |
| DNLJ_MAGSA_1 | Q2W0G3 | 5.39 | 75279.63 |
| DNLJ_MANSM_1 | Q65RN7 | 6 | 74739.55 |
| DNLJ_MARMS_1 | A6W0S9 | 5.14 | 74174.6 |
| DNLJ_METC4_1 | B7KU78 | 5.37 | 87641.28 |
| DNLJ_NITHX_1 | Q1QNT2 | 5.84 | 78180.76 |
| DNLJ_OCEIH_1 | Q8CXK6 | 4.89 | 74917.98 |
| DNLJ_BURCJ_1 | B4ECN6 | 5.29 | 75465.67 |
| DNLJ_OENOB_1 | Q04GN6 | 5.67 | 76288.54 |
| DNLJ_MYCA1_1 | A0QJE3 | 5.31 | 75258.33 |
| DNLJ_PARD8_1 | A6LA55 | 5.49 | 74714.77 |
| DNLJ_MYCA9_1 | B1MDV2 | 5.23 | 74082.62 |
| DNLJ_MYCAP_1 | A5IY71 | 6.15 | 74269.85 |
| DNLJ_MYCBO_1 | P63974 | 5.42 | 75289.16 |
| DNLJ_PARPJ_1 | B2T5K0 | 5.35 | 75029.2 |
| DNLJ_PARUW_1 | Q6MAB5 | 7.28 | 75277.86 |
| DNLJ_PARXL_1 | Q13XB0 | 5.35 | 75268.48 |
| DNLJ_PASMU_1 | Q9CKA9 | 5.77 | 74609.62 |
| DNLJ_MYCGI_1 | A4TDZ8 | 5.02 | 77282 |
| DNLJ_PELCD_1 | Q3A2F5 | 6.02 | 74672.35 |
| DNLJ_PELPB_1 | B4SCX5 | 5.61 | 75751.54 |
| DNLJ_CALBD_1 | B9MK54 | 5.97 | 76749.43 |
| DNLJ_MYCLE_1 | O33102 | 5.49 | 75912.4 |
| DNLJ_CAMC5_1 | A7GXS4 | 5.41 | 72773.39 |
| DNLJ_MYCMO_1 | Q6KI95 | 8.8 | 77598.29 |
| DNLJ_CAMHC_1 | A7I236 | 5.65 | 72444.4 |
| DNLJ_MYCPE_1 | Q8EWL9 | 6.23 | 80848.15 |
| DNLJ_CAMJE_1 | Q0PAT1 | 5.59 | 73939.32 |
| DNLJ_MYCPU_1 | Q98QH2 | 8.28 | 79499.48 |
| DNLJ_BORPE_1 | Q7VRX7 | 5.98 | 75586.17 |
| DNLJ_BORT9_1 | A1QZY4 | 8.8 | 76712.17 |
| DNLJ_ANOFW_1 | B7GFV1 | 5.81 | 75546.16 |
| DNLJ1_PSECP_1 | B8HFJ3 | 4.93 | 83455.99 |
| DNLJ_AZOC5_1 | A8IGY0 | 5.76 | 80555.87 |
| DNLJ_BRAHW_1 | C0QVE5 | 5.67 | 75016.78 |
| DNLJ_BACFR_1 | Q64TM7 | 5.62 | 75356.79 |
| DNLJ_BUCBP_1 | Q89B02 | 9.6 | 76902.99 |
| DNLJ_ACIET_1 | B9MIW4 | 6.6 | 79294.47 |
| DNLJ_BACSK_1 | Q5WJ30 | 5.14 | 74638.05 |
| DNLJ_ACISJ_1 | A1W7N4 | 6.28 | 78461.4 |
| DNLJ_BACWK_1 | A9VRG3 | 5.03 | 75251.25 |
| DNLJ_AGRFC_1 | A9CIB3 | 5.69 | 79391.87 |
| DNLJ_AGRVS_1 | B9JY41 | 5.45 | 78786.17 |
| DNLJ_ALCBS_1 | Q0VR01 | 5.1 | 84627.97 |
| DNLJ_ALIF1_1 | Q5E3L1 | 5.13 | 73610.14 |
| DNLJ_LISMO_1 | Q8Y6D0 | 4.92 | 74855.28 |
| DNLJ_LISW6_1 | A0AJL1 | 4.87 | 74720.09 |
| DNLJ_NITEC_1 | Q0AHH6 | 5.91 | 75296.63 |
| DNLJ_NITOC_1 | Q3JAI1 | 6.68 | 75171.32 |
| DNLJ_NITWN_1 | Q3STR7 | 5.6 | 78588.95 |
| DNLJ_NOCSJ_1 | A1SMA5 | 5.33 | 81360.87 |
| DNLJ_NOSP7_1 | B2J3P0 | 5.61 | 75992.78 |
| DNLJ_NOSS1_1 | Q8YW98 | 6.18 | 75791.65 |
| DNLJ_NOVAD_1 | Q2G7J5 | 5.21 | 78115.39 |
| DNLJ_METS4_1 | B0UQ51 | 6.49 | 88540.4 |
| DNLJ_OLICO_1 | B6JB45 | 6.64 | 77723.57 |
| DNLJ_MICAN_1 | B0JTW2 | 6.01 | 74966.71 |
| DNLJ_ORITB_1 | A5CEY0 | 8.92 | 78109.85 |
| DNLJ_MOOTA_1 | Q2RGY0 | 6.08 | 72386.15 |
| DNLJ_BURM1_1 | A9AIK9 | 5.37 | 75499.74 |
| DNLJ_PARL1_1 | A7HVV8 | 5.55 | 78597.02 |
| DNLJ_BURM9_1 | A2SB66 | 5.76 | 75583.89 |
| DNLJ_BURP0_1 | A3NWN7 | 5.76 | 75595.94 |
| DNLJ_MYCGE_1 | P47496 | 9.16 | 75388.18 |
| DNLJ_PEDPA_1 | Q03DT9 | 4.95 | 74957.23 |
| DNLJ_BURTA_1 | Q2SX03 | 5.64 | 75655.82 |
| DNLJ_MYCH7_1 | Q4A899 | 9.33 | 79467.64 |
| DNLJ_PELTS_1 | A5CZ68 | 6.79 | 75382.2 |
| DNLJ_CALS8_1 | A4XK85 | 6.06 | 76668.26 |
| DNLJ_ANAVT_1 | Q3MGE7 | 6.34 | 75547.34 |
| DNLJ_BORHD_1 | B2S0Q2 | 8.71 | 75700.99 |
| DNLJ_BREBN_1 | C0Z4D6 | 5.24 | 74178.66 |
| DNLJ2_STRGG_1 | B1VZW1 | 5.16 | 81427.8 |
| DNLJ_BRUMB_1 | C0RE59 | 5.64 | 78623.26 |
| DNLJ_ACIB5_1 | B7I790 | 5.57 | 75377.09 |
| DNLJ_ACIC5_1 | C1F7S7 | 5.75 | 74882.65 |
| DNLJ_AERHH_1 | A0KHL9 | 5.53 | 72369 |
| DNLJ_BART1_1 | A9IW95 | 5.68 | 80427.93 |
| DNLJ_BAUCH_1 | Q1LU20 | 9.54 | 76270.96 |
| DNLJ_ALKEH_1 | Q0AAV2 | 5.41 | 73795.58 |
| DNLJ_BIFLO_1 | Q8G830 | 4.99 | 99855.24 |
| DNLJ_ANAPZ_1 | Q2GLI8 | 6.16 | 74768.03 |
| DNLJ_LEUMM_1 | Q03YP0 | 4.94 | 74408.46 |
| DNLJ_NATTJ_1 | B2A5W5 | 5.64 | 74760.94 |
| DNLJ_NEIG2_1 | B4RJQ9 | 5.55 | 90301.15 |
| DNLJ_MARHV_1 | A1TZT9 | 5.1 | 75064.02 |
| DNLJ_NEIMA_1 | A1IQR7 | 5.78 | 92372.69 |
| DNLJ_MESFL_1 | Q6F1V7 | 5.26 | 75210.68 |
| DNLJ_METCA_1 | Q604U8 | 6.17 | 74248.93 |
| DNLJ_NITEU_1 | Q82TW6 | 5.88 | 75128.61 |
| DNLJ_METFK_1 | Q1H1G6 | 5.85 | 75729.22 |
| DNLJ_METI4_1 | B3DWU2 | 7.55 | 77417.85 |
| DNLJ_METNO_1 | B8ICE5 | 6.02 | 89489.34 |
| DNLJ_METPB_1 | B1Z9P1 | 5.45 | 87861.53 |
| DNLJ_METRJ_1 | B1M575 | 5.52 | 86772.22 |
| DNLJ_METSB_1 | B8ETN6 | 6.18 | 76137.69 |
| DNLJ_ONYPE_1 | Q6YQD6 | 9.41 | 75992.75 |
| DNLJ_BURCM_1 | Q0BE10 | 5.2 | 75376.4 |
| DNLJ_PAEAT_1 | A1R4P6 | 4.87 | 83412.06 |
| DNLJ_PARDP_1 | A1B9F5 | 5.2 | 81897.26 |
| DNLJ_PARP8_1 | B2JID1 | 5.46 | 75013.26 |
| DNLJ_MYCGA_1 | Q7NAF8 | 8.08 | 82782.91 |
| DNLJ_PELPD_1 | A1ATJ6 | 5.5 | 79186.06 |
| DNLJ_CALS4_1 | Q8RC42 | 5.71 | 74963.48 |
| DNLJ_PELUB_1 | Q4FPL3 | 9.61 | 77338.72 |
| DNLJ_CAMC1_1 | A7ZED8 | 5.19 | 71346.58 |
| DNLJ_MYCMM_1 | B2HIF0 | 5.27 | 75053.72 |
| DNLJ_PERMH_1 | C0QSF6 | 6.22 | 81702.88 |
| DNLJ_CAMFF_1 | A0RPY7 | 5.46 | 72478.25 |
| DNLJ_MYCMS_1 | Q6MSK8 | 7.92 | 75656 |
| DNLJ_MYCPN_1 | P78021 | 8.4 | 73970.11 |
| DNLJ_ARCB4_1 | A8ETQ3 | 5.17 | 73519.12 |
| DNLJ1_NOCFA_1 | Q5YVN7 | 5.03 | 70584.34 |
| DNLJ_BORRA_1 | B5RPQ2 | 8.92 | 76413.06 |
| DNLJ_BRADU_1 | Q89FV8 | 5.81 | 79565.39 |
| DNLJ_AZOVD_1 | C1DN27 | 5.63 | 85405.51 |
| DNLJ_BACHD_1 | Q9KF37 | 5.18 | 74241.51 |
| DNLJ_AKKM8_1 | B2UL69 | 5.87 | 86424.66 |
| DNLJ_BIFAA_1 | A1A200 | 4.98 | 97284.44 |
| DNLJ_LEUCK_1 | B1MXQ8 | 5.08 | 74459.76 |
| DNLJ_LISIN_1 | Q92AQ0 | 5.01 | 74860.45 |
| DNLJ_ACICJ_1 | A5FWJ5 | 5.64 | 73602.69 |
| DNLJ_BACP2_1 | A8FAQ1 | 4.98 | 74781.32 |
| DNLJ_BACTN_1 | Q8A9C1 | 5.34 | 75133.36 |
| DNLJ_AERS4_1 | A4SKA6 | 5.63 | 72102.65 |
| DNLJ_ALKOO_1 | A8MLJ8 | 5.48 | 74558.71 |
| DNLJ_AMOA5_1 | C3L496 | 6.98 | 75432.95 |
| DNLJ_BORBP_1 | Q660X2 | 8.91 | 75524.77 |
| DNLJ_MYCS5_1 | Q4A5J6 | 8.13 | 80464.13 |
| DNLJ_MYCVP_1 | A1T6X7 | 5.14 | 76837.62 |
| DNLJ_LYSSC_1 | B1HTW6 | 4.86 | 73732.01 |
| DNLJ_MACCJ_1 | B9E830 | 5.15 | 74195.78 |
| DNLJ_MAGMM_1 | A0LDY1 | 6.25 | 82444.06 |
| DNLJ_MARMM_1 | Q0AMX9 | 5.19 | 76147.01 |
| DNLJ_NEOSM_1 | Q2GDR2 | 6.74 | 75576.47 |
| DNLJ_NITMU_1 | Q2YBB7 | 6.15 | 77729.32 |
| DNLJ_NITSB_1 | A6Q433 | 5.87 | 75770.4 |
| DNLJ_METPP_1 | A2SGT2 | 6.5 | 73209.54 |
| DNLJ_OCHA4_1 | A6WZR6 | 5.42 | 79287.69 |
| DNLJ_BURL3_1 | Q39F38 | 5.23 | 75559.78 |
| DNLJ_MYCA5_1 | B3PMJ0 | 6.15 | 75736.87 |
| DNLJ_MYCCT_1 | Q2SRE1 | 6.29 | 75777.98 |
| DNLJ_PECAS_1 | Q6D165 | 5.35 | 74483.18 |
| DNLJ_BURVG_1 | A4JF81 | 5.25 | 75507.71 |
| DNLJ1_DEIDV_1 | C1D1Y6 | 5.83 | 74918.96 |
| DNLJ_AROAE_1 | Q5NYU3 | 6.11 | 73901.24 |
| DNLJ_ARTS2_1 | A0JUH2 | 4.89 | 85847.22 |
| DNLJ_BACLD_1 | Q65MR2 | 5.13 | 74782.41 |
| DNLJ_BACV8_1 | A6L5G8 | 5.53 | 75171.53 |
| DNLJ_BARHE_1 | Q6G2R5 | 5.71 | 79736.2 |
| DNLJ_ALKMQ_1 | A6TLU4 | 5.28 | 74291.08 |
| DNLJ_BORA1_1 | Q2KUU5 | 5.84 | 75424.86 |
| DNLJ_BORDL_1 | B5RMA6 | 8.72 | 76300.78 |
| DNLJ_ACAM1_1 | B0C308 | 5.27 | 74770.78 |
| DNLJ_AYWBP_1 | Q2NJF5 | 9.38 | 76061.54 |
| DNLJ_BRASB_1 | A5EPJ2 | 5.84 | 79657.41 |
| DNLJ_ACIAC_1 | A1TR21 | 6.35 | 78896.87 |
| DNLJ_BRUO2_1 | A5VRG5 | 5.59 | 78709.35 |
| DNLJ_LAWIP_1 | Q1MRS8 | 9.21 | 76924.84 |
| DNLJ_PHOLL_1 | Q7MBH2 | 5.32 | 74267.91 |
| DNLJ_PHOPR_1 | Q6LTU5 | 4.93 | 74028.4 |
| DNLJ_PHYAS_1 | B1VAY1 | 9.42 | 75821.19 |
| DNLJ_PSELT_1 | A8F541 | 8.74 | 77212.01 |
| DNLJ_SHELP_1 | A3QFJ5 | 5.13 | 72969.22 |
| DNLJ_SHEPA_1 | A8H376 | 5.29 | 73112.46 |
| DNLJ_SHESH_1 | A8FU26 | 5.29 | 73264.66 |
| DNLJ_SODGM_1 | Q2NSC2 | 5.44 | 74812.34 |
| DNLJ_STRGC_1 | A8AY10 | 5.09 | 71994.86 |
| DNLJ_UREU1_1 | B5ZAV3 | 6.05 | 77284.17 |
| RNaseH | Accession number | pI | Molecular Weight |
| RNH_ECOLI_1 | P0A7Y4 | 8.43 | 17596.99 |
| RNH_WIGBR_1 | Q8D3D3 | 9.81 | 18309.53 |
| RNH_MYCSM_1 | Q07705 | 7.02 | 17528.83 |
| RNH1_BACHD_1 | Q9KEI9 | 8.42 | 22373.36 |
| RNH_RHIME_1 | Q92RG0 | 8.91 | 17178.34 |
| RNH_SALTY_1 | P0A2B9 | 7.75 | 17509.82 |
| RNH_THET8_1 | P29253 | 9.44 | 18728.39 |
| RNH_STRCO_1 | Q9X7R6 | 9.16 | 24297.32 |
| RNH_HAEIN_1 | P43807 | 8.37 | 17640.08 |
| RNH_RHOBA_1 | Q7UF86 | 8.33 | 17665.06 |
| RNH_SHEON_1 | Q8EE30 | 8.43 | 17782.34 |
| RNH_DESAG_1 | Q30X61 | 9.1 | 16976.25 |
| RNH_XYLFA_1 | Q9PBI6 | 8.4 | 16921.19 |
| RNH_CHLL7_1 | Q3B2H0 | 9.3 | 16453.63 |
| RNH_SHIFL_1 | P0A7Y7 | 8.43 | 17596.99 |
| RNH_PSYIN_1 | A1SS86 | 6.59 | 17266.58 |
| RNH_PSEAE_1 | Q9I2S9 | 8.6 | 16696.93 |
| RNH_EHRRG_1 | Q5FG88 | 8.99 | 16549.11 |
| RNH_RICCN_1 | Q92GL5 | 8.31 | 17057.41 |
| RNH_BRADU_1 | Q89UU3 | 9.14 | 17263.58 |
| RNH_ANAMM_1 | Q5PBQ8 | 8.49 | 17026.41 |
| RNH_YERPE_1 | Q8ZH30 | 6.51 | 17463.56 |
| RNH_GEOSL_1 | Q74BH0 | 8.41 | 16747.83 |
| RNH_BLOPB_1 | Q493H7 | 8.8 | 17620.38 |
| RNH_SALDC_1 | B5FJ58 | 7.75 | 17509.82 |
| RNH_CAUVN_1 | B8H4W7 | 9.22 | 16674.05 |
| RNH_VIBVU_1 | Q8DBD5 | 8.42 | 17647.01 |
| RNH_PSEU5_1 | A4VLR0 | 9.17 | 17175.54 |
| RNH_ENT38_1 | A4W6V4 | 7.05 | 17544.82 |
| RNH_SOLUE_1 | Q01RW8 | 8.93 | 16636.93 |
| RNH_FRATN_1 | A0Q6W0 | 9.23 | 16976.32 |
| RNH_RICTY_1 | Q68W20 | 9.26 | 17176.76 |
| RNH_SALAR_1 | A9MPF1 | 7.75 | 17509.82 |
| RNH_HAHCH_1 | Q2SJ45 | 7.74 | 16674.91 |
| RNH_KLEP3_1 | B5Y1G2 | 8.42 | 17657 |
| RNH_LACP7_1 | A9KLJ9 | 6.51 | 17484.7 |
| RNH_LEGPL_1 | Q5WWW5 | 7.79 | 16255.48 |
| RNH_RHIEC_1 | Q2KBL2 | 8.4 | 16800.06 |
| RNH_BORPE_1 | Q7VRX8 | 9.56 | 17486.75 |
| RNH_HELPJ_1 | Q9ZLH3 | 9.41 | 16208.63 |
| RNH_BRUSU_1 | P66674 | 8.83 | 17172.43 |
| RNH_SALPK_1 | B5BDW5 | 7.75 | 17509.82 |
| RNH_JANSC_1 | Q28V43 | 8.69 | 17173.33 |
| RNH_PSEU2_1 | Q4ZVL1 | 7.75 | 16777.03 |
| RNH_WOLWR_1 | C0R2X2 | 8.43 | 16548.78 |
| RNH_ERYLH_1 | Q2ND39 | 6.38 | 16249.33 |
| RNH_FRAAA_1 | Q0RJ31 | 8.05 | 17315.35 |
| RNH_SYNWW_1 | Q0AV47 | 9.1 | 16399.64 |
| RNH_NITWN_1 | Q3SP51 | 9.25 | 17187.53 |
| RNH_RHIL3_1 | Q1MKH6 | 8.38 | 16611.86 |
| RNH_BART1_1 | A9IQR5 | 8.61 | 17425.7 |
| RNH_BRASB_1 | A5EAL2 | 9.42 | 17127.44 |
| RNH_BRUO2_1 | A5VP47 | 8.83 | 17172.43 |
| RNH_CALS8_1 | A4XKQ3 | 8.81 | 16696.39 |
| RNH_IDILO_1 | Q5QZL3 | 8.75 | 18079.29 |
| RNH_PSEA6_1 | Q15TA7 | 8.43 | 17256.46 |
| RNH_CAMJE_1 | Q9PM39 | 7.71 | 16978.45 |
| RNH_SHEB2_1 | B8E599 | 8.43 | 17584.18 |
| RNH_PSEF5_1 | Q4KBI1 | 7.74 | 16959.1 |
| RNH_SHEFN_1 | Q081L4 | 7.76 | 17889.5 |
| RNH3_LACLA_1 | Q9CDG3 | 9.06 | 32205.12 |
| RNH_SHIB3_1 | B2U352 | 8.43 | 17596.99 |
| RNH_ACIF2_1 | B7J4E2 | 9.03 | 17832.22 |
| RNH_FRAP2_1 | B0TZ91 | 9.26 | 16804.04 |
| RNH_SINMW_1 | A6U6V5 | 7.85 | 17124.2 |
| RNH_SULNB_1 | A6QCI9 | 6.22 | 16671.7 |
| RNH_GEOMG_1 | Q39X47 | 8.69 | 17168.5 |
| RNH_SYNFM_1 | A0LGJ7 | 8.94 | 18837.39 |
| RNH_YERPB_1 | B2KAC9 | 6.51 | 17463.56 |
| RNH_HAEDU_1 | Q7VM15 | 9.05 | 17452.97 |
| RNH_NITOC_1 | Q3J7D4 | 9.46 | 16743.97 |
| RNH_RHOPS_1 | Q131J2 | 9.33 | 16781.97 |
| RNH_ANASK_1 | B4UMK8 | 6.85 | 19428.79 |
| RNH_POLNA_1 | A1VMW5 | 7.81 | 17380.73 |
| RNH_CALS4_1 | Q8RA67 | 7.67 | 17560.9 |
| RNH_PSEE4_1 | Q1I7T4 | 8.55 | 16809.19 |
| RNH_VIBCM_1 | C3LPN8 | 8.43 | 17953.4 |
| RNH_PSEP1_1 | A5W169 | 8.56 | 16913.28 |
| RNH_SHEPC_1 | A4Y6C1 | 8.44 | 17685.24 |
| RNH_SHESH_1 | A8FUS8 | 9.12 | 17565.31 |
| RNH_COXB1_1 | B6J5V1 | 9.4 | 17662.04 |
| RNH_EDWI9_1 | C5BEV5 | 8.47 | 17319.57 |
| RNH_XANOM_1 | Q2P6Y2 | 6.97 | 16899.94 |
| RNH_GRABC_1 | Q0BQX7 | 6.14 | 16865.93 |
| RNH_NEIMB_1 | Q9JYE5 | 8.79 | 16251.26 |
| RNH_THEEB_1 | Q8DM24 | 8.67 | 17790.2 |
| RNH_ANAD2_1 | B8J731 | 6.85 | 19432.78 |
| RNH_NOVAD_1 | Q2G9E3 | 8.52 | 16085.42 |
| RNH_RUEPO_1 | Q5LNJ2 | 9.3 | 17151.61 |
| RNH_CAMC1_1 | A8Z6F7 | 7.69 | 16276.5 |
| RNH_MARHV_1 | A1U0U9 | 9.03 | 16424.59 |
| RNH_TREPS_1 | B2S2V0 | 6.18 | 18185.6 |
| RNH_PECAS_1 | Q6D1V7 | 8.77 | 17371.59 |
| RNH_RUEST_1 | Q1GDG1 | 9.21 | 17133.37 |
| RNH_SACEN_1 | A4FMU3 | 8.46 | 17293.42 |
| RNH_SALA4_1 | B5F8X2 | 7.75 | 17509.82 |
| RNH_CYAP4_1 | B8HPS9 | 7.72 | 17052.16 |
| RNH_SALEP_1 | B5R449 | 7.75 | 17509.82 |
| RNH_DESVV_1 | A1VFS4 | 8.91 | 17413.84 |
| RNH_TROW8_1 | Q83HK9 | 6.42 | 18086.25 |
| RNH_PROMA_1 | Q7VDY9 | 7.79 | 18671.01 |
| RNH_DINSH_1 | A8LLC1 | 9.3 | 17163.4 |
| RNH_HYDCU_1 | Q31H49 | 5.93 | 16177.23 |
| RNH_CITK8_1 | A8AKR0 | 7.01 | 17537.83 |
| RNH_SHEWM_1 | B1KHK0 | 8.75 | 17946.53 |
| RNH_XANAC_1 | Q8PNH8 | 7.79 | 16856.03 |
| RNH_EHRCJ_1 | Q3YR62 | 7.72 | 16549.95 |
| RNH_EHRCR_1 | Q2GHJ9 | 6.72 | 16638.1 |
| RNH_ESCF3_1 | B7LW89 | 8.43 | 17596.99 |
| RNH_NEOSM_1 | Q2GDA1 | 8.5 | 17019.28 |
| RNH_NITMU_1 | Q2Y8K1 | 9.14 | 17662.18 |
| RNH_PECCP_1 | C6DC65 | 8.35 | 17367.62 |
| RNH_PHOLL_1 | Q7N807 | 6.59 | 17840.92 |
| RNH_AROAE_1 | Q5NYP6 | 8.89 | 16940.08 |
| RNH_BARBK_1 | A1URX4 | 8.33 | 17591.81 |
| RNH_CUPNH_1 | Q0K8W6 | 8.97 | 16053.11 |
| RNH_BRUAB_1 | Q57EP4 | 8.83 | 17172.43 |
| RNH_SALHS_1 | B4TK85 | 7.75 | 17509.82 |
| RNH_BRUME_1 | P66673 | 8.83 | 17172.43 |
| RNH_CHRVO_1 | Q7NYL8 | 8.89 | 16668.84 |
| RNH_SHESW_1 | A1RK75 | 8.44 | 17685.24 |
| RNH_XANC5_1 | Q3BWP3 | 6.97 | 16928.08 |
| RNH_ACISJ_1 | A1W6Q8 | 9 | 16409.71 |
| RNH_SODGM_1 | Q2NVF9 | 8.91 | 18367.79 |
| RNH_GLUOX_1 | Q5FUH9 | 5.61 | 16877.91 |
| RNH_NITEC_1 | Q0AE34 | 6.66 | 18501.68 |
| RNH_TREDE_1 | Q73K21 | 8.61 | 17924.4 |
| RNH_RICCK_1 | A8EXT7 | 8.64 | 17381.79 |
| RNH_BARHE_1 | Q6G4C3 | 8.59 | 17503.88 |
| RNH_BORBR_1 | Q7WCJ8 | 9.56 | 17454.69 |
| RNH_DESRM_1 | A4J7B3 | 9.05 | 18017.57 |
| RNH_VEREI_1 | A1WFG9 | 9.03 | 16794.03 |
| RNH_PROMH_1 | B4EUG3 | 7.76 | 18308.74 |
| RNH_VESOH_1 | A5CX29 | 9.26 | 17183.71 |
| RNH_METCA_1 | Q60AW8 | 9.14 | 17507.77 |
| RNH_OCHA4_1 | A6WWG8 | 8.49 | 17232.44 |
| RNH_RICBR_1 | Q1RJL7 | 7.68 | 17520 |
| RNH_POLAQ_1 | A4SXM6 | 7.19 | 17208.39 |
| RNH_DICNV_1 | A5EXP9 | 9.48 | 17037.65 |
| RNH_JANMA_1 | A6SXA4 | 7.83 | 15955.07 |
| RNH_CHESB_1 | Q11KC5 | 8.57 | 18135.19 |
| RNH_METEP_1 | A9W185 | 9 | 24204.6 |
| RNH_AERS4_1 | A4SPI4 | 6.96 | 17326.63 |
| RNH_NITHX_1 | Q1QH30 | 9.17 | 16792.93 |
| RNH_THIDA_1 | Q3SIB2 | 9.37 | 16596.7 |
| RNH_BORA1_1 | Q2KV56 | 8.64 | 17086.45 |
| RNH_POLSJ_1 | Q12B88 | 8.97 | 17460.78 |
| RNH_CAMC5_1 | A7H185 | 6.83 | 16550.73 |
| RNH_SHEAM_1 | A1S6T0 | 8.73 | 17532.04 |
| RNH_SHEHH_1 | B0TRM1 | 8.99 | 18049.52 |
| RNH_MOOTA_1 | Q2RKU0 | 8.84 | 17113.39 |
| RNH_SYMTH_1 | Q67K93 | 8.49 | 16583.67 |
| RNH_ALIF1_1 | Q5E3G5 | 8.39 | 17914.36 |
| RNH_PASMU_1 | P57813 | 8.42 | 17555.91 |
| RNH_RHOS5_1 | A4WNV1 | 9.16 | 16727.99 |
| RNH_RICAH_1 | A8GPT6 | 8.84 | 17018.42 |
| RNH_RUTMC_1 | A1AW38 | 8.91 | 16448.74 |
| RNH_SALG2_1 | B5R5L3 | 7.75 | 17509.82 |
| RNH_SALSV_1 | B4TYH0 | 7.75 | 17509.82 |
| RNH_SERP5_1 | A8GA77 | 8.77 | 17519.9 |
| RNH_SHELP_1 | A3QET0 | 8.43 | 17515.07 |
| RNH_CHRSD_1 | Q1QW64 | 7.86 | 18272.57 |
| RNH_CLOB1_1 | A7FR34 | 8.64 | 17131.63 |
| RNH_METFK_1 | Q1H190 | 7.8 | 16854.11 |
| RNH_SYNAS_1 | Q2LWY9 | 9.37 | 19094.73 |
| RNH_GEOUR_1 | A5G5F6 | 8.72 | 16514.97 |
| RNH_ALKEH_1 | Q0A753 | 7.93 | 16609.53 |
| RNH_OLICO_1 | B6JJ39 | 7.92 | 16748.87 |
| RNH_TRIEI_1 | Q115G0 | 9.08 | 17720.3 |
| RNH_HELAH_1 | Q17XJ7 | 9.18 | 16250.75 |
| RNH_HERAR_1 | A4G7G3 | 7.85 | 16195.25 |
| RNH_SALTI_1 | P0A2C0 | 7.75 | 17509.82 |
| RNH_SHEDO_1 | Q12MM4 | 9.13 | 18115.69 |
| RNH_PSEMY_1 | A4XU11 | 7.78 | 16756.93 |
| RNH_CHLL2_1 | B3EH35 | 9.17 | 16377.61 |
| RNH_CHLP8_1 | B3QM41 | 9.13 | 16595.99 |
| RNH_WOLPM_1 | Q73I74 | 8.43 | 16590.87 |
| RNH_RALSO_1 | Q8XZ91 | 8.55 | 16597.82 |
| RNH_AERHH_1 | A0KIK4 | 8.79 | 17296.73 |
| RNH_YERE8_1 | A1JKB1 | 6.2 | 17504.65 |
| RNH_ALCBS_1 | Q0VQ76 | 7.79 | 16861.12 |
| RNH_THEPX_1 | B0K1A7 | 8.35 | 17785.08 |
| RNH_ORITB_1 | A5CEP5 | 9.35 | 19335.08 |
| RNH_PARL1_1 | A7HQX4 | 7.83 | 16816.97 |
| RNH_PELPD_1 | A1AT66 | 7.75 | 18269.51 |
| RNH_PHEZH_1 | B4R8T3 | 8.83 | 16888.18 |
| RNH_RICM5_1 | A8F2L0 | 8.64 | 17017.4 |
| RNH_PSE14_1 | Q48KX6 | 7.79 | 16698.94 |
| RNH_ACTP2_1 | A3MZE1 | 8.38 | 17385.8 |
| RNH_NITEU_1 | Q82XV8 | 6.1 | 18194.58 |
| RNH_ZYMMO_1 | O69014 | 8.43 | 17250.65 |
| RNH_RHOFT_1 | Q21YF6 | 8.62 | 16513.74 |
| RNH_ROSDO_1 | Q16AK0 | 9.1 | 16398.47 |
| RNH_BARQU_1 | Q6G0C8 | 8.9 | 17742.21 |
| RNH_BAUCH_1 | Q1LT02 | 9.22 | 17995.74 |
| RNH_BUCAP_1 | Q08885 | 9.52 | 18733.84 |
| RNH_VIBPA_1 | Q87MG2 | 7.75 | 17508.73 |
| RNH_CHLPD_1 | A1BE10 | 9.13 | 16382.66 |
| RNH_ACICJ_1 | A5FZ26 | 7.86 | 17857.09 |
| RNH_SHIDS_1 | Q32JP9 | 8.43 | 17596.99 |
| RNH_NEIG1_1 | Q5F7K9 | 7.74 | 16196.17 |
| RNH_SYNY3_1 | Q55801 | 8.39 | 17856.22 |
| RNH_RHORT_1 | Q2RPU6 | 8.85 | 18482.69 |
| RNH_PELCD_1 | Q3A827 | 6.52 | 16958.14 |
| RNH_SALCH_1 | Q57SZ6 | 7.75 | 17509.82 |
| RNH_PSEHT_1 | Q3IIS1 | 8.68 | 17402.95 |
| RNH_CHLCH_1 | Q3APT0 | 9.35 | 16235.53 |
| RNH_VIBTL_1 | B7VIP1 | 8.43 | 17532.81 |
| RNH_MARMM_1 | Q0AMI4 | 9.13 | 16706.87 |
| RNH_ACIAC_1 | A1TQI7 | 9.33 | 16122.47 |
| RNH_ERWT9_1 | B2VHJ5 | 8.78 | 17328.73 |
| RNH_SHISS_1 | Q3Z5E9 | 8.43 | 17596.99 |
| RNH_ACTSZ_1 | A6VMP9 | 8.43 | 17750.12 |
| RNH_ALISL_1 | B6EJV2 | 7.73 | 17711.08 |
| RNH_THEP3_1 | B0K9M0 | 8.35 | 17785.08 |
| RNH_BLOFL_1 | Q7VQB6 | 8.99 | 19480.35 |
| RNH_DECAR_1 | Q47FN9 | 7.86 | 16621.91 |
| RNH_HALHL_1 | A1WXD7 | 5.74 | 16961.87 |
| RNH_MANSM_1 | Q65S82 | 8.73 | 18089.57 |
| RNH_TOLAT_1 | C4LC60 | 6.96 | 17481.79 |
| RNH_PARDP_1 | A1B840 | 8.85 | 17153.34 |
| RNH_RICFE_1 | Q4UN27 | 8.31 | 17057.41 |
| RNH_RICPR_1 | Q9ZCK3 | 9.12 | 17013.56 |
| RNH_SALNS_1 | B4SV39 | 7.75 | 17509.82 |
| RNH_PROA2_1 | B4S5K2 | 9.38 | 16490.91 |
| RNH_HYPNA_1 | Q0C3M1 | 8.52 | 16853.06 |
| RNH_VIBCB_1 | A7MY21 | 8.4 | 17453.69 |
| RNH_CAUSK_1 | B0T025 | 9.6 | 17070.64 |
| RNH_MAGSA_1 | Q2W9A9 | 8.52 | 17050.32 |
| RNH_CLOTH_1 | A3DD79 | 8.33 | 16913.16 |
| RNH_CROS8_1 | A7MI34 | 8.44 | 17951.31 |
| RNH_CUPMC_1 | Q1LL89 | 8.59 | 15966.01 |
| RNH_MYCGI_1 | A4T6Y5 | 7.82 | 16680.97 |
| RNH_AGRFC_1 | Q8UHA7 | 7.79 | 16261.38 |
| RNH_RHILO_1 | Q985W1 | 9.44 | 19457.97 |
| DNA Topoisomerase I | Accession number | pI | Molecular Weight |
| TOP1_ECOLI_1 | P06612 | 8.68 | 97349.76 |
| TOP1_MYCTU_1 | P9WG49 | 8.19 | 102335.5 |
| TOP1_MYCS2_1 | A0R5D9 | 6.28 | 102368 |
| TOP1_STAAB_1 | Q2YXL8 | 9.12 | 79122.86 |
| TOP1_PSEAE_1 | Q9HZJ5 | 8.7 | 97282.76 |
| TOP1_THEMA_1 | P46799 | 9.41 | 72694.77 |
| TOP1_VIBCH_1 | Q9KRB2 | 7.96 | 97909.56 |
| TOP1_ZYMMO_1 | Q9X3X7 | 6.1 | 130689.1 |
| TOP1_SALTY_1 | P0A2I1 | 8.56 | 97302.61 |
| TOP1_STAS1_1 | Q49X35 | 8.89 | 79129.5 |
| TOP1_SALTI_1 | P0A2I2 | 8.56 | 97302.61 |
| TOP1_SYNE7_1 | P34185 | 7.62 | 98295.62 |
| TOP1_HELPY_1 | P55991 | 9.04 | 83195.85 |
| TOP1_BACSU_1 | P39814 | 8.97 | 79078.22 |
| TOP1_RICPR_1 | Q9ZDK2 | 8.88 | 88722.22 |
| TOP1_BORBU_1 | O51768 | 9.51 | 97663.21 |
| TOP1_BUCAP_1 | Q8K9P7 | 9.75 | 98816.38 |
| TOP1_LACLA_1 | Q9CG80 | 9.1 | 80741.32 |
| TOP1_MYCLE_1 | O69548 | 8.06 | 104055.8 |
| TOP1_RICFE_1 | Q4UM42 | 8.53 | 88141.14 |
| TOP1_RICTY_1 | Q68X45 | 8.95 | 89082.62 |
| TOP1_BACPE_1 | P34184 | 9.13 | 80167.55 |
| TOP1_PASMU_1 | Q9CN30 | 9.01 | 98048.1 |
| TOP1_AQUAE_1 | O66893 | 9.52 | 63427.08 |
| TOP1_HAEIN_1 | P43012 | 8.78 | 98144.05 |
| TOP1_MYCGE_1 | P47368 | 9.27 | 82544.56 |
| TOP1_RICCN_1 | Q92IH1 | 8.78 | 88145.27 |
| TOP1_FERIS_1 | O34204 | 9.08 | 79612.06 |
| TOP1_MYCBO_1 | P0A621 | 8.19 | 102335.5 |
| TOP1_MYCGA_1 | Q9JN65 | 9.1 | 82772.9 |
| TOP1_MYCPN_1 | P78032 | 9.26 | 81965.44 |
| TOP1_BACAN_1 | P40114 | 8.77 | 100092.2 |
| TOP1_BACHD_1 | Q9KA23 | 8.29 | 78910.51 |
| TOP1_CAMJE_1 | Q9PLZ2 | 9.06 | 79148.21 |
| TOP1_RICBR_1 | Q1RIM1 | 8.71 | 91228.7 |
| TOP1_STAES_1 | Q8CSU3 | 9.11 | 79519 |
| TOP1_STAHJ_1 | Q4L5V2 | 9.09 | 79373.04 |
| TOP1_TREPA_1 | O83409 | 9.5 | 82441.22 |
| TOP1_XYLFA_1 | Q9PEV8 | 9.07 | 91802.63 |
| TOP1_STRCO_1 | Q9X909 | 8.77 | 103584.8 |
| TOP1_SYNY3_1 | P73810 | 8.88 | 99340.48 |
| Tus Protein | Accession number | pI | Molecular Weight |
| TUS_ECOLI_1 | P16525 | 9.57 | 35783.18 |
| TUS_YERPE_1 | Q9L6X9 | 8.76 | 36180.03 |
| TUS_SALTY_1 | O52714 | 9.58 | 35486.71 |
| TUS_CROS8_1 | A7MMM5 | 9.08 | 35235.78 |
| TUS_YERE8_1 | A1JML7 | 8.97 | 35310.83 |
| TUS_SHIDS_1 | Q32FG8 | 9.65 | 35678.05 |
| TUS_ENT38_1 | A4W9Y2 | 7.74 | 35463.98 |
| TUS_SALTI_1 | Q8Z6R7 | 9.47 | 35331.51 |
| TUS_CITK8_1 | A8AGY9 | 9.49 | 35543.85 |
| TUS_SALAR_1 | A9MRV0 | 9.49 | 35383.57 |
| TUS_KLEP7_1 | A6T8M8 | 9.51 | 35259.83 |
| TUS_SHIFL_1 | Q83KZ2 | 9.57 | 35771.13 |
| TUS_SALPB_1 | A9N011 | 9.58 | 35500.74 |
| TUS_ESCF3_1 | B7LQQ4 | 9.08 | 35802.23 |
| TUS_YERP3_1 | A7FHW9 | 8.92 | 36173 |
| TUS_SALNS_1 | B4T5A5 | 9.58 | 35486.71 |
| TUS_SALCH_1 | Q57PG8 | 9.58 | 35512.79 |
| TUS_SALHS_1 | B4THQ0 | 9.58 | 35470.71 |
| TUS_SALA4_1 | B5F6H7 | 9.58 | 35482.77 |
| TUS_SHISS_1 | Q3Z1W3 | 9.57 | 35741.1 |
| TUS_SALEP_1 | B5QUF5 | 9.58 | 35486.71 |
| TUS_SHIBS_1 | Q320W6 | 9.57 | 35727.07 |
| TUS_SALSV_1 | B4TVD5 | 9.58 | 35486.71 |
| TUS_SALDC_1 | B5FID6 | 9.58 | 35486.71 |
| TUS_SALG2_1 | B5RAJ1 | 9.58 | 35486.71 |
| DNA Topoisomerase IV | Accession number | pI | Molecular Weight |
| PARE_STRPN_1 | Q59961 | 6.34 | 71664.36 |
| PARE_ECOLI_1 | P20083 | 5.44 | 70243.91 |
| PARC_ECOLI_1 | P0AFI2 | 6.24 | 83831.27 |
| PARC_STRPN_1 | P72525 | 5.49 | 93133.12 |
| PARE_SALTY_1 | P0A2I5 | 5.55 | 70088.66 |
| PARE_FRATH_1 | A0A0J9WZF0 | 6.43 | 70355.3 |
| PARE_BARBK_1 | P94281 | 6.74 | 77445.66 |
| PARE_PSEAE_1 | Q9HUJ8 | 5.72 | 69178.98 |
| PARE_STAA8_1 | Q2FYS5 | 6.46 | 74363.84 |
| PARE_ENTFA_1 | H7C794 | 5.48 | 76257.3 |
| PARE_CAUVC_1 | O54479 | 6.61 | 76163.71 |
| PARE_BACSU_1 | Q59192 | 6.19 | 73006.69 |
| PARC_MYCPN_1 | P75352 | 9 | 88668.78 |
| PARC_RICCN_1 | Q92JH0 | 8.47 | 83245.52 |
| PARC_SYNY3_1 | P73077 | 5.33 | 101162.6 |
| PARC_BORBU_1 | O51066 | 8.54 | 72042.23 |
| PARC_NEIGO_1 | P48374 | 8.95 | 84895.84 |
| PARC_RICFE_1 | Q4UNA0 | 8.18 | 83127.35 |
| PARE_MYCCT_1 | P50028 | 7.64 | 72352.12 |
| PARC_RICTY_1 | Q68XU1 | 8.94 | 83414.09 |
| PARE_RICFE_1 | Q4UKT4 | 6.81 | 73953.85 |
| PARC_MYCGE_1 | P47446 | 9.2 | 88512.63 |
| PARE_HAEIN_1 | P43703 | 5.71 | 70173.05 |
| PARC_CAUVC_1 | O54478 | 5.92 | 83521.13 |
| PARC_HAEIN_1 | P43702 | 6.06 | 83367.16 |
| PARC_SHIFL_1 | P0AFI4 | 6.24 | 83831.27 |
| PARE_RICCN_1 | Q92IW1 | 8.37 | 73826.91 |
| PARE_RICTY_1 | Q68XE1 | 8.61 | 74506.76 |
| PARC_SALTY_1 | P26973 | 6.12 | 84037.66 |
| PARE_MYCGE_1 | P47445 | 9.06 | 71569.33 |
| PARE_RICPR_1 | Q9ZDU7 | 8.69 | 74251.6 |
| PARE_STAEQ_1 | Q5HPI6 | 6.21 | 74470.08 |
| PARE_BORBU_1 | Q59189 | 8.22 | 68774.56 |
| PARC_PSEAE_1 | Q9HUK1 | 5.76 | 83365.26 |
| PARC_RICPR_1 | Q9ZE79 | 8.59 | 83347.85 |
| PARC_STAAW_1 | Q8NWU8 | 5.95 | 90945.05 |
| PARC_RICBR_1 | Q1RGX8 | 7.94 | 82955.11 |
| PARE_MYCPN_1 | P78016 | 9.03 | 72412.84 |
| PARE_RICBR_1 | Q1RK03 | 7.26 | 73985.97 |
| PARC_STAEQ_1 | Q5HPI5 | 5.72 | 91145 |
| PARC_BACSU_1 | Q45066 | 6.32 | 91320.54 |
| PARE_MYCGA_1 | Q59526 | 8.99 | 71372.79 |
| PARC_STRP1_1 | Q9L7Q4 | 5.52 | 92478.15 |
| PARE_SALTI_1 | P0A2I6 | 5.55 | 70088.66 |
| PARC_RHIME_1 | Q59749 | 5.96 | 83753.88 |
