# Supplementary material for: In silico analysis of bacterial translation factors reveal distinct translation event specific pI values
Source: BMC Genomics. 2021 Mar 29;22:220. doi: 10.1186/s12864-021-07472-x (PMC8008671; doi:10.1186/s12864-021-07472-x)
Supplement: Supplementary file 3 — Additional file 3: Proteins of the process of transcription. Fig. S3 and S4. Box plot diagram of pI values, and molecular weight value distribution of the proteins of the process of transcription. Table S3. Accession numbers, pI values and MW values of the proteins of transcription factors. [file 12864_2021_7472_MOESM3_ESM.docx]

**Additional file 3 of Proteins of the process of transcription.**

Additional file 3: Fig. S3. Box plot diagram of pI value distribution of the proteins of the process of transcription.


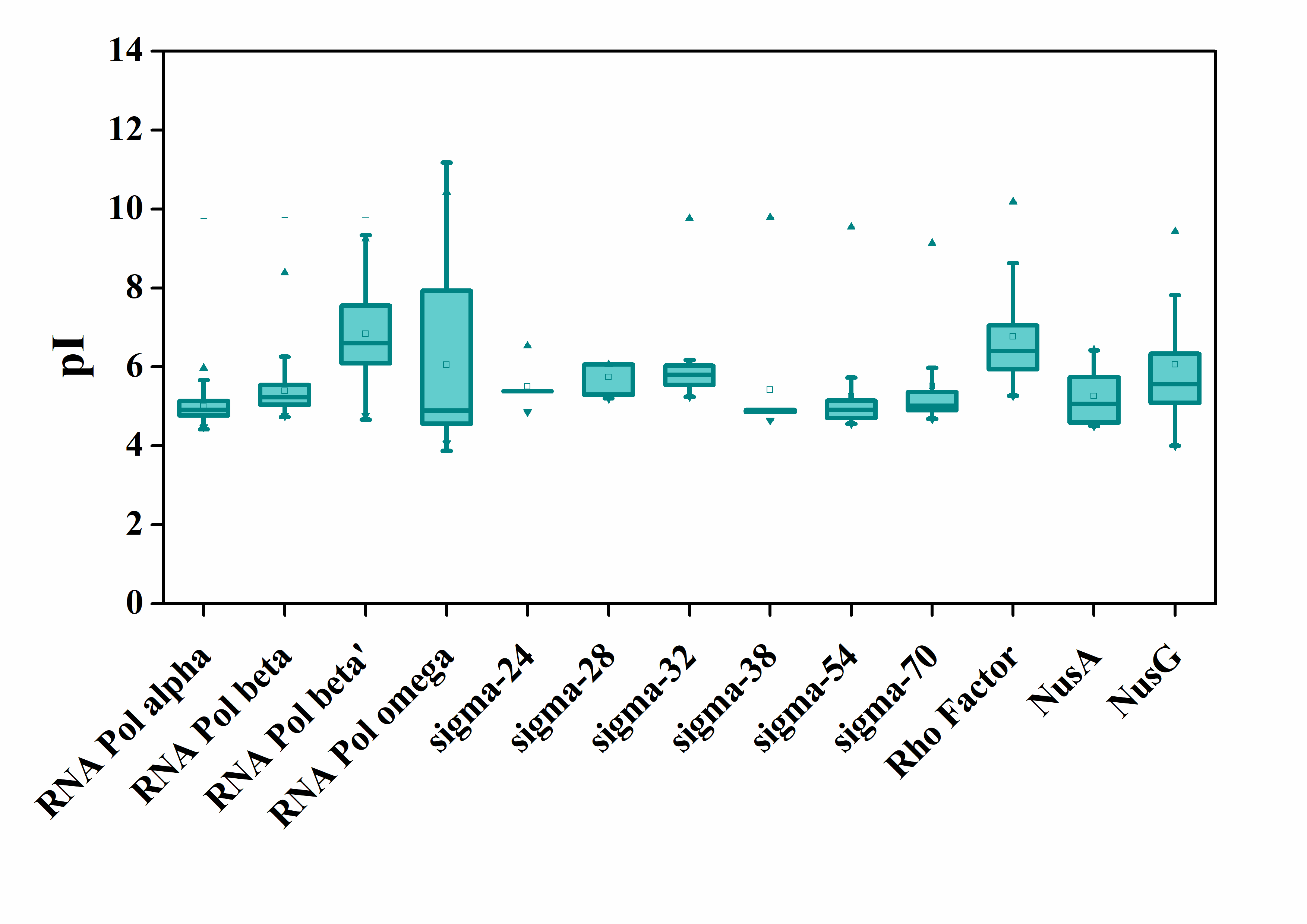


Additional file 3: Fig. S4. Box plot diagram of molecular weight value distribution of the proteins of the process of transcription.


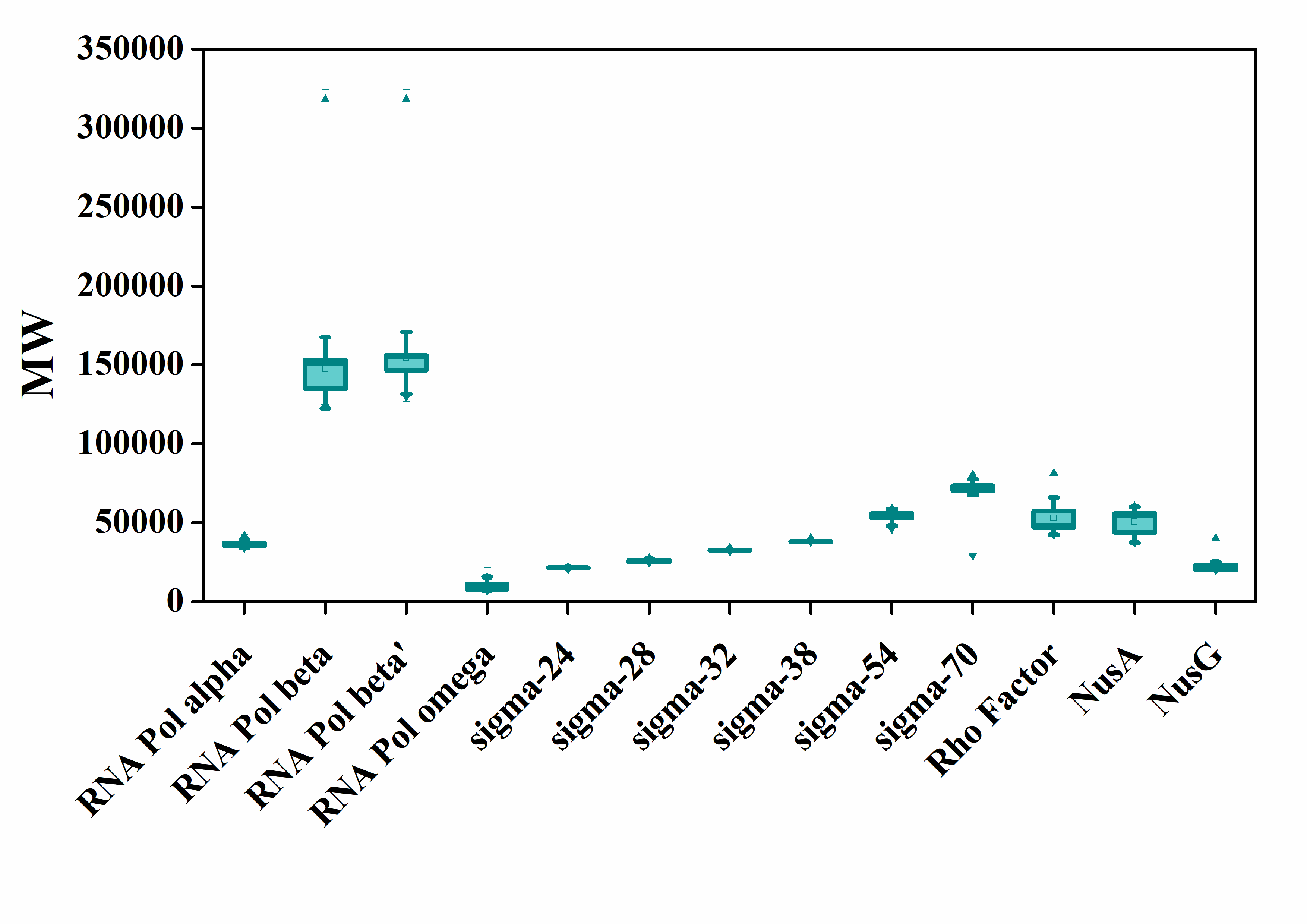


Additional file 3: Table S3. Accession numbers, pI values and MW values of the proteins of transcription factors.

| RNA Polymerase subunit alpha | Accession number | pI | Molecular Weight |
| --- | --- | --- | --- |
| RPOA_ECOLI_1 | P0A7Z4 | 4.97 | 36511.72 |
| RPOA_MYCTU_1 | P9WGZ1 | 4.64 | 37706.48 |
| RPOA_THETH_1 | Q9Z9H6 | 4.93 | 35013.26 |
| RPOA_XANCP_1 | P0A0Y1 | 5.58 | 36232.33 |
| RPOA_THEAQ_1 | Q9KWU8 | 5.1 | 34786.96 |
| RPOA_BACSU_1 | P20429 | 4.79 | 34799.45 |
| RPOA_MYCS2_1 | A0QSL8 | 4.62 | 37919.59 |
| RPOA_STRGT_1 | Q9X4V6 | 4.66 | 36709.79 |
| RPOA_LEPBL_1 | Q055B7 | 4.86 | 36617.53 |
| RPOA_MYCSP_1 | P38018 | 5.07 | 40097.44 |
| RPOA_YERPE_1 | Q8ZJ87 | 4.97 | 36508.72 |
| RPOA_SHEVD_1 | Q9S0Q8 | 4.78 | 36239.31 |
| RPOA_RHIME_1 | Q925Z2 | 4.77 | 37171.3 |
| RPOA_YERPS_1 | Q664U6 | 4.97 | 36508.72 |
| RPOA_YERE8_1 | A1JS01 | 4.97 | 36595.8 |
| RPOA_SALTI_1 | P0A7Z8 | 4.97 | 36511.72 |
| RPOA_CHLPN_1 | Q9Z7S8 | 5.07 | 41874.51 |
| RPOA_ZYMMO_1 | Q5NQ40 | 5.12 | 38299.69 |
| RPOA_PARD8_1 | A6LEG5 | 4.94 | 37189.75 |
| RPOA_PROMA_1 | Q7V9Y5 | 4.67 | 34274.72 |
| RPOA_SYNPW_1 | A5GIS2 | 4.61 | 34136.51 |
| RPOA_SYNWW_1 | Q0AUK9 | 4.81 | 35581.4 |
| RPOA_UREPA_1 | Q9PQN4 | 5.58 | 36817.44 |
| RPOA_VIBCH_1 | Q9KP08 | 4.82 | 36415.53 |
| RPOA_VIBVY_1 | Q7MPG4 | 4.77 | 36459.49 |
| RPOA_XANOP_1 | B2SQT4 | 5.58 | 36363.52 |
| RPOA_XYLFA_1 | P66712 | 5.62 | 36553.88 |
| RPOA_ALKOO_1 | A8MLG9 | 4.75 | 34965.08 |
| RPOA_NATTJ_1 | B2A4Q0 | 4.77 | 35209.07 |
| RPOA_NITWN_1 | Q3SSU2 | 4.94 | 37516.88 |
| RPOA_NOCSJ_1 | A1SNI8 | 4.58 | 36847.61 |
| RPOA_NOVAD_1 | Q2G5B0 | 5.01 | 38727.33 |
| RPOA_OCEIH_1 | Q8ETV9 | 4.62 | 34746.41 |
| RPOA_PAEAT_1 | A1R8R5 | 4.68 | 36187.95 |
| RPOA_PARUW_1 | Q6ME42 | 5.47 | 42444.4 |
| RPOA_PELCD_1 | Q3A6M0 | 5.03 | 37659.04 |
| RPOA_PEPD6_1 | Q18CI5 | 4.62 | 34919.98 |
| RPOA_STRA5_1 | Q8E2B1 | 4.84 | 34515.55 |
| RPOA_STRGG_1 | B1W3Y0 | 4.66 | 36695.76 |
| RPOA_STRM5_1 | B4SLH5 | 5.59 | 36301.48 |
| RPOA_STRP3_1 | P0DF28 | 4.9 | 34545.58 |
| RPOA_STRTD_1 | Q03IH7 | 4.74 | 34433.26 |
| RPOA_SULNB_1 | A6QCS4 | 4.8 | 37008.15 |
| RPOA_TRIEI_1 | Q110D1 | 4.82 | 34971.74 |
| RPOA_TROW8_1 | Q820D8 | 4.78 | 35889.64 |
| RPOA_WIGBR_1 | Q8D1Y8 | 5.57 | 36927.62 |
| RPOA_WOLTR_1 | Q5GSW7 | 4.84 | 39281.79 |
| RPOA_ACIB5_1 | B7IA14 | 5.1 | 37260.49 |
| RPOA_AERHH_1 | A0KF45 | 4.91 | 36195.34 |
| RPOA_AGRFC_1 | P0A4E3 | 4.77 | 37171.3 |
| RPOA_ALIF1_1 | Q5E889 | 4.76 | 36358.38 |
| RPOA_ALKEH_1 | Q0ABF0 | 4.75 | 36511.32 |
| RPOA_ANADE_1 | Q2IJ57 | 5.32 | 37363.86 |
| RPOA_ANAPZ_1 | Q2GL36 | 5.32 | 37856.17 |
| RPOA_OENOB_1 | Q04G60 | 4.73 | 34515.95 |
| RPOA_PELPD_1 | A1ALW7 | 5.29 | 38203.56 |
| RPOA_PSE14_1 | Q48D61 | 4.91 | 36673.78 |
| RPOA_PSEE4_1 | Q1IFU1 | 4.91 | 36629.72 |
| RPOA_SORC5_1 | A9FGB8 | 5.29 | 38476.1 |
| RPOA_STAA1_1 | A7X5C4 | 4.68 | 35011.72 |
| RPOA_ACIAC_1 | A1TJU2 | 6.04 | 36247.44 |
| RPOA_PSESM_1 | Q889U6 | 4.91 | 36673.78 |
| RPOA_SHEON_1 | Q8EK47 | 4.77 | 36160.2 |
| RPOA_SHESA_1 | A0KRP9 | 4.77 | 36160.2 |
| RPOA_SHIBS_1 | Q31VY1 | 4.97 | 36511.72 |
| RPOA_SHIDS_1 | Q32B56 | 5.01 | 36539.78 |
| RPOA_SINMW_1 | A6U883 | 4.77 | 37171.3 |
| RPOA_SODGM_1 | Q2NQP7 | 5 | 36471.75 |
| RPOA_SPHWW_1 | A5V5X5 | 4.89 | 38000.34 |
| RPOA_NITEU_1 | Q82X69 | 5.29 | 36934.05 |
| RPOA_ONYPE_1 | Q6YQZ5 | 5.41 | 36761.36 |
| RPOA_PARL1_1 | A7HWT5 | 4.86 | 37601.85 |
| RPOA_PEDPA_1 | Q03EE2 | 4.71 | 34909.63 |
| RPOA_PSEMY_1 | A4XZ65 | 4.87 | 36569.63 |
| RPOA_SALCH_1 | Q57J56 | 4.93 | 36512.71 |
| RPOA_SHIFL_1 | P0A7Z9 | 4.97 | 36511.72 |
| RPOA_SINFN_1 | C3MB03 | 4.77 | 37187.29 |
| RPOA_THEM4_1 | A6LLP1 | 5.03 | 37440.18 |
| RPOA_TREDE_1 | Q73PK8 | 4.77 | 39289.27 |
| RPOA_VIBCB_1 | A7N0H8 | 4.77 | 36484.55 |
| RPOA_VIBTL_1 | B7VLD2 | 4.77 | 36500.55 |
| RPOA_PECAS_1 | Q6CZZ5 | 4.97 | 36521.81 |
| RPOA_STRSV_1 | A3CK90 | 4.66 | 34259.96 |
| RPOA_THEP1_1 | A5IMB1 | 5.25 | 38529.44 |
| RPOA_THEPX_1 | B0K5S1 | 5.37 | 35093.35 |
| RPOA_THIDA_1 | Q3SLM4 | 5.27 | 35553.7 |
| RPOA_TOLAT_1 | C4L7V5 | 4.96 | 36055.34 |
| RPOA_XANAC_1 | P0A0Y2 | 5.58 | 36232.33 |
| RPOA_NEIMA_1 | P66703 | 4.94 | 36076.12 |
| RPOA_NITHX_1 | Q1QN06 | 5 | 37482.93 |
| RPOA_NITOC_1 | Q3J8T8 | 5.23 | 36885.37 |
| RPOA_PHEZH_1 | B4R8P1 | 4.99 | 37287.77 |
| RPOA_POLAQ_1 | A4SUY6 | 5.32 | 35780.17 |
| RPOA_PORGI_1 | Q7MTP0 | 4.88 | 36798.18 |
| RPOA_STAS1_1 | Q49ZE2 | 4.74 | 34979.79 |
| RPOA_STRPN_1 | P66708 | 4.64 | 34206.85 |
| RPOA_ACIAD_1 | Q6F7T7 | 5.1 | 37216.44 |
| RPOA_AGRVS_1 | B9JVR1 | 4.81 | 37162.29 |
| RPOA_ALISL_1 | B6EPU9 | 4.76 | 36370.44 |
| RPOA_ANADF_1 | A7HBP5 | 5.33 | 37494.03 |
| RPOA_POLNS_1 | B1XSS7 | 5.31 | 35743.15 |
| RPOA_PSEF5_1 | Q4K557 | 4.91 | 36629.72 |
| RPOA_PSEP1_1 | A5VXS2 | 4.91 | 36629.72 |
| RPOA_STAES_1 | Q8CRI4 | 4.71 | 34985.73 |
| RPOA_STRGC_1 | A8AZJ9 | 4.7 | 34502.27 |
| RPOA_THISH_1 | B8GV33 | 5.43 | 36856.14 |
| RPOA_VARPS_1 | C5CQ75 | 5.8 | 36182.5 |
| RPOA_VESOH_1 | A5CXI9 | 4.54 | 35693.67 |
| RPOA_NEOSM_1 | Q2GEB6 | 5.85 | 35795.97 |
| RPOA_NITSB_1 | A6Q1K4 | 4.74 | 37086.57 |
| RPOA_PSEAE_1 | O52760 | 4.88 | 36649.63 |
| RPOA_STAHJ_1 | Q4L888 | 4.71 | 34993.73 |
| RPOA_SYNFM_1 | A0LIL7 | 5.06 | 37927.28 |
| RPOA_VEREI_1 | A1WK92 | 5.97 | 36315.55 |
| RPOA_NEIG1_1 | Q5F5V2 | 4.99 | 36001.01 |
| RPOA_NOCFA_1 | Q5Z1K9 | 4.68 | 37985.74 |
| RPOA_PARP8_1 | B2JI39 | 5.64 | 35611.87 |
| RPOA_SHEB5_1 | A3DA47 | 4.77 | 36156.19 |
| RPOA_SHEFN_1 | Q089M9 | 4.75 | 36072.02 |
| RPOA_SHEPC_1 | A4YBV8 | 4.77 | 36142.17 |
| RPOA_SOLUE_1 | Q01WC0 | 4.98 | 38239.22 |
| RPOA_STRMU_1 | Q8DS36 | 4.76 | 34570.5 |
| RPOA_STRS2_1 | A4VYR8 | 4.78 | 34285.2 |
| RPOA_SYMTH_1 | Q67JX1 | 4.93 | 35030.07 |
| RPOA_SYNAS_1 | Q2LQC9 | 4.99 | 38599.14 |
| RPOA_TREPA_1 | O83242 | 5 | 39544.86 |
| RPOA_PROA2_1 | B4S5A1 | 4.88 | 36934.9 |
| RPOA_PSEA6_1 | Q15X48 | 4.75 | 36249.33 |
| RPOA_SALPA_1 | Q5PK10 | 4.97 | 36511.72 |
| RPOA_SERP5_1 | A8GKH3 | 4.97 | 36465.7 |
| RPOA_SHEAM_1 | A1S243 | 4.83 | 36104.12 |
| RPOA_SHEDO_1 | Q12ST4 | 4.77 | 36245.26 |
| RPOA_SHELP_1 | A3Q9A7 | 4.77 | 36201.21 |
| RPOA_SHISS_1 | Q3YWW4 | 4.97 | 36511.72 |
| RPOA_SPHAL_1 | Q1GVN6 | 5 | 38361.93 |
| RPOA_NITMU_1 | Q2YAX2 | 5.66 | 36639.07 |
| RPOA_NOSS1_1 | Q8YPK3 | 4.82 | 35072.65 |
| RPOA_OLICO_1 | B6JEY9 | 4.9 | 37613.98 |
| RPOA_PARXL_1 | Q13TJ6 | 5.64 | 35655.93 |
| RPOA_SALRD_1 | Q2S3N9 | 4.45 | 36701.1 |
| RPOA_SALTY_1 | P0A7Z7 | 4.97 | 36511.72 |
| RPOA_SULDN_1 | Q30TS3 | 4.73 | 36546.4 |
| RPOA_SYNY3_1 | P73297 | 4.72 | 35003.77 |
| RPOA_THEFY_1 | Q47LM1 | 4.91 | 36881.87 |
| RPOA_VIBPA_1 | Q87SZ0 | 4.77 | 36472.49 |
| RPOA_WOLSU_1 | Q7M8F7 | 4.93 | 36685.98 |
| RPOA_XANP2_1 | A7IPP6 | 5.02 | 37486.95 |
| RPOA_ACICJ_1 | A5FZU1 | 4.95 | 38133.58 |
| RPOA_ACIET_1 | B9MBW2 | 5.87 | 36056.29 |
| RPOA_ACISJ_1 | A1W333 | 5.87 | 36056.29 |
| RPOA_AERS4_1 | A4SSY1 | 4.91 | 36195.34 |
| RPOA_AGRRK_1 | B9JDV2 | 4.75 | 37124.28 |
| RPOA_ALCBS_1 | Q0VSH8 | 4.91 | 37284.46 |
| RPOA_ALKMQ_1 | A6TWF3 | 4.58 | 34819.83 |
| RPOA_ACTP2_1 | A3N382 | 5.18 | 36539.7 |
| RPOA_NITEC_1 | Q0AIH1 | 5.25 | 37027.22 |
| RPOA_OCHA4_1 | A6X0E2 | 4.85 | 37333.52 |
| RPOA_PELUB_1 | Q4FLP1 | 4.98 | 37814.47 |
| RPOA_PHOPR_1 | Q6LV91 | 4.82 | 36388.42 |
| RPOA_POLNA_1 | A1VJ40 | 5.75 | 36305.45 |
| RPOA_ORITI_1 | B3CT29 | 5.7 | 38082.09 |
| RPOA_PASMU_1 | P57941 | 4.9 | 36494.64 |
| RPOA_PELPB_1 | B4SBX4 | 5.03 | 37046.27 |
| RPOA_PHOLL_1 | Q7MYH5 | 4.97 | 36480.75 |
| RPOA_POLSJ_1 | Q12G77 | 5.75 | 36257.4 |
| RPOA_PSECP_1 | B8HCX4 | 4.68 | 36096.86 |
| RPOA_PSEHT_1 | Q3IJI6 | 4.65 | 36230.16 |
| RPOA_PSELT_1 | A8F4T9 | 5.5 | 37894.04 |
| RPOA_ACIC1_1 | A0LRP8 | 4.89 | 37534.66 |
| RPOA_ACTSZ_1 | A6VLL3 | 4.9 | 36449.51 |
| RPOA_ANAMM_1 | Q5PA81 | 5.02 | 39795.96 |
| RPOA_PARDP_1 | A1B052 | 4.75 | 37106.21 |
| RPOA_PARPJ_1 | B2T725 | 5.64 | 35655.93 |
| RPOA1_FRATF_1 | A7N9U9 | 4.93 | 35357.51 |
| RPOA1_PSYIN_1 | A1SXW8 | 4.73 | 36335.58 |
| RPOA_COXBR_1 | A9NAZ5 | 5.61 | 35555.77 |
| RPOA_CHLCV_1 | Q824N1 | 5.07 | 41653.93 |
| RPOA_AYWBP_1 | Q2NIY1 | 5.41 | 36837.46 |
| RPOA_BRUME_1 | Q8YHL6 | 4.85 | 37312.52 |
| RPOA_CHLPD_1 | A1BJ07 | 5.06 | 37132.38 |
| RPOA_CUPNH_1 | Q0K645 | 5.49 | 35673.9 |
| RPOA_BACHD_1 | O50634 | 4.68 | 34805.47 |
| RPOA_CUTAK_1 | Q6A6R1 | 4.42 | 36905.35 |
| RPOA_BACP2_1 | A8F9B2 | 4.79 | 34948.64 |
| RPOA_KINRD_1 | A6W5W5 | 4.69 | 36565.26 |
| RPOA_BARBK_1 | A1USR8 | 4.89 | 37551.01 |
| RPOA_BURCH_1 | A0K3Q1 | 5.76 | 35696.98 |
| RPOA_LACCB_1 | B3WAJ2 | 5.01 | 34758.67 |
| RPOA_CLOBA_1 | B2UYD9 | 4.95 | 35157.38 |
| RPOA_LACDB_1 | Q04BY9 | 4.81 | 34774.62 |
| RPOA_BURCM_1 | Q0BJ20 | 5.76 | 35696.98 |
| RPOA_METPP_1 | A2SLD1 | 6.28 | 36903.32 |
| RPOA_BLOPB_1 | Q493I4 | 5.21 | 37072.55 |
| RPOA_FERNB_1 | A7HM24 | 5.35 | 37356.13 |
| RPOA_LACPL_1 | Q88XW0 | 4.79 | 34842.48 |
| RPOA_LACRJ_1 | B2G8V2 | 4.74 | 34901.57 |
| RPOA_FLAPJ_1 | A6GZ73 | 4.98 | 37391.87 |
| RPOA_BORPE_1 | P0A4E5 | 5.6 | 36158.54 |
| RPOA_LAWIP_1 | Q1MPP1 | 5.2 | 38940.27 |
| RPOA_BRASB_1 | A5ELK3 | 4.91 | 37916.33 |
| RPOA_BURP6_1 | A3NEF3 | 5.76 | 35684.97 |
| RPOA_MYCPA_1 | Q73S43 | 4.67 | 37704.49 |
| RPOA_LEGPA_1 | Q5X834 | 5.21 | 36746.94 |
| RPOA_DESHY_1 | Q250K4 | 4.78 | 35094.23 |
| RPOA_LEIXX_1 | Q6AD21 | 4.67 | 36216.77 |
| RPOA_GLOVI_1 | Q7NFF5 | 4.83 | 34975.68 |
| RPOA_DESOH_1 | A8ZV82 | 4.87 | 37434.67 |
| RPOA_CAMC1_1 | A7ZFY7 | 4.74 | 37733.22 |
| RPOA_CAMC5_1 | A7H0Y9 | 4.75 | 37923.41 |
| RPOA_DESRM_1 | A4J139 | 4.83 | 35431.36 |
| RPOA_LEPCP_1 | B1Y7M9 | 6.18 | 36308.73 |
| RPOA_GRAFK_1 | A0M573 | 5.03 | 37400.75 |
| RPOA_CARHZ_1 | Q3A9U4 | 4.91 | 34907.26 |
| RPOA_CAUSK_1 | B0T2E6 | 4.97 | 37247.65 |
| RPOA_HAEIE_1 | A5UDS2 | 4.99 | 36402.63 |
| RPOA_CAUVN_1 | B8H4F8 | 4.97 | 37272.62 |
| RPOA_CELJU_1 | B3PK62 | 5.14 | 36234.33 |
| RPOA_CHESB_1 | Q11HS6 | 4.84 | 37204.46 |
| RPOA_LISMF_1 | Q71WH2 | 4.8 | 34905.71 |
| RPOA_HELMI_1 | B0TC85 | 4.96 | 35242.33 |
| RPOA_HELPH_1 | Q1CRW7 | 5.01 | 38485.2 |
| RPOA_PSEU5_1 | A4VHQ5 | 4.96 | 36528.62 |
| RPOA_PSYCK_1 | Q1QDG1 | 4.71 | 37372.55 |
| RPOA_HERAR_1 | A4G9R3 | 5.42 | 35593.84 |
| RPOA1_STRAW_1 | P60313 | 4.66 | 36695.76 |
| RPOA_ANAVT_1 | Q3MF97 | 4.82 | 35045.62 |
| RPOA_CHLAB_1 | Q5L6Z9 | 5.18 | 41765.19 |
| RPOA_ARCB4_1 | A8ETK6 | 4.76 | 36925.25 |
| RPOA_BRUA2_1 | Q2YRU0 | 4.9 | 37384.61 |
| RPOA_CHLMU_1 | Q9PJN4 | 5.34 | 41833.3 |
| RPOA_CROS8_1 | A7MPF8 | 4.97 | 36467.67 |
| RPOA_BACCR_1 | Q81J18 | 4.88 | 34935.82 |
| RPOA_CUPTR_1 | B3R7E3 | 5.49 | 35673.9 |
| RPOA_HYDCU_1 | Q31IV8 | 4.79 | 36173.34 |
| RPOA_ENTFA_1 | Q839D9 | 4.87 | 35050.21 |
| RPOA_ERYLH_1 | Q2N9D7 | 4.83 | 38474.02 |
| RPOA_MANSM_1 | Q65QY0 | 4.9 | 36468.6 |
| RPOA_MARHV_1 | A1TYM2 | 4.89 | 37032.99 |
| RPOA_MARMM_1 | Q0ANS4 | 4.75 | 37769.92 |
| RPOA_DEHM1_1 | Q3Z953 | 4.78 | 36359.43 |
| RPOA_LACJO_1 | Q74L64 | 4.7 | 34812.66 |
| RPOA_BURL3_1 | Q39KE1 | 5.76 | 35696.98 |
| RPOA_METRJ_1 | B1LWP9 | 4.93 | 37217.57 |
| RPOA_METS4_1 | B0UHU5 | 5.06 | 37326.79 |
| RPOA_CLOK1_1 | B9DYD7 | 4.83 | 35260.21 |
| RPOA_MOOTA_1 | Q2RFS6 | 4.77 | 34930.9 |
| RPOA_BORBP_1 | Q661B8 | 5.14 | 38716.06 |
| RPOA_MYCA1_1 | A0QKU5 | 4.67 | 37704.49 |
| RPOA_LACS1_1 | Q1WSB6 | 4.75 | 34872.64 |
| RPOA_BORPA_1 | P0A4E7 | 5.6 | 36158.54 |
| RPOA_FRACC_1 | Q2JFE8 | 4.58 | 37858.67 |
| RPOA_MYCLE_1 | Q9X798 | 4.63 | 37702.49 |
| RPOA_CORGL_1 | Q8NSV2 | 4.48 | 36670.36 |
| RPOA_CORJK_1 | Q4JTC2 | 4.49 | 36817.31 |
| RPOA_CAMJD_1 | A7H5U5 | 4.88 | 37790.12 |
| RPOA_HAEDU_1 | Q7VKF8 | 5.06 | 36417.6 |
| RPOA_LISIN_1 | P66700 | 4.8 | 34905.71 |
| RPOA_HELAH_1 | Q17ZB4 | 4.97 | 38535.38 |
| RPOA_PSYA2_1 | Q4FUD1 | 4.71 | 37372.55 |
| RPOA_EHRCJ_1 | Q3YRN2 | 4.98 | 41393.12 |
| RPOA_EHRRG_1 | Q5FFS1 | 5.09 | 41807.65 |
| RPOA_RHIE6_1 | B3PWU5 | 4.75 | 37239.37 |
| RPOA_RHIL3_1 | Q1MIB7 | 4.75 | 37239.37 |
| RPOA_RHILO_1 | Q98N33 | 4.81 | 37119.48 |
| RPOA_RHIRD_1 | P0A4E4 | 4.77 | 37171.3 |
| RPOA_RHOBA_1 | Q7UIC5 | 5.14 | 36760.77 |
| RPOA_RHOCS_1 | B6IRT0 | 5.01 | 37068.6 |
| RPOA_RHOFT_1 | Q21QP9 | 5.89 | 36090.29 |
| RPOA_RHOJR_1 | Q0S3E7 | 4.62 | 37913.65 |
| RPOA_RHORT_1 | Q2RQY4 | 4.79 | 37453.01 |
| RPOA_RHOS1_1 | A3PGN5 | 4.71 | 37095.27 |
| RPOA_RICAE_1 | C3PP83 | 5.17 | 38292.19 |
| RPOA_RICAH_1 | A8GPC6 | 5.48 | 38266.25 |
| RPOA_RICB8_1 | A8GVD7 | 5.38 | 38336.05 |
| RPOA_RICCK_1 | A8EZJ2 | 5.24 | 38080.07 |
| RPOA_RICCN_1 | Q92GZ0 | 5.17 | 38306.17 |
| RPOA_RICFE_1 | Q4UMQ5 | 5.23 | 38288.16 |
| RPOA_RICM5_1 | A8F2C3 | 5.22 | 38216.14 |
| RPOA_RICPR_1 | Q9ZCS9 | 5.22 | 38360.23 |
| RPOA_RICPU_1 | C4K2F5 | 5.24 | 38287.22 |
| RPOA_RICRO_1 | B0BUN6 | 5.24 | 38261.2 |
| RPOA_RICTY_1 | Q68WA2 | 5.3 | 38329.16 |
| RPOA_ROSCS_1 | A7NR36 | 4.95 | 35525.58 |
| RPOA_ROSDO_1 | Q16AB8 | 4.6 | 37086.03 |
| RPOA_ROSS1_1 | A5USG2 | 5.08 | 35489.58 |
| RPOA_RUBXD_1 | Q1AU56 | 4.71 | 34577.02 |
| RPOA_RUEPO_1 | Q5LW32 | 4.58 | 37147.14 |
| RPOA_RUEST_1 | Q1GK04 | 4.62 | 37229.31 |
| RPOA_SACEN_1 | A4FPJ2 | 4.57 | 38692.62 |
| RPOA_CHLT3_1 | B3QYF0 | 4.89 | 36974.96 |
| RPOA_ENT38_1 | A4WFA3 | 4.97 | 36465.7 |
| RPOA_CHLTR_1 | P0CE08 | 5.34 | 41824.31 |
| RPOA_BACSK_1 | Q5WLN5 | 4.72 | 34950.55 |
| RPOA_CHRSD_1 | Q1R0F0 | 4.68 | 37021.89 |
| RPOA_JANMA_1 | A6T3H8 | 5.41 | 35565.79 |
| RPOA_BACTN_1 | Q8A4A2 | 4.87 | 37448.84 |
| RPOA_CYTH3_1 | Q11QD8 | 5.09 | 37188.89 |
| RPOA_BART1_1 | A9IVZ5 | 4.65 | 37693.91 |
| RPOA_MESFL_1 | Q6F1W7 | 5.16 | 34705.86 |
| RPOA_BAUCH_1 | Q1LTB3 | 5.35 | 36605.19 |
| RPOA_DEIGD_1 | Q1IX99 | 4.88 | 36195.28 |
| RPOA_LACP3_1 | Q035A9 | 5.01 | 34758.67 |
| RPOA_CLOP1_1 | Q0TMS5 | 4.84 | 35176.35 |
| RPOA_BURM9_1 | A2S7K2 | 5.76 | 35684.97 |
| RPOA_DESAG_1 | Q30Z68 | 5.01 | 38992.36 |
| RPOA_LACSS_1 | Q38UT7 | 4.87 | 34845.78 |
| RPOA_COREF_1 | Q8FS33 | 4.44 | 36784.43 |
| RPOA_FUSNN_1 | Q8RE44 | 4.69 | 36577.2 |
| RPOA_GEOSL_1 | Q749B3 | 5.26 | 37726.14 |
| RPOA_CALS4_1 | Q8R7Y2 | 5.47 | 35151.5 |
| RPOA_LEPBA_1 | B0SA20 | 4.86 | 36599.62 |
| RPOA_DESPS_1 | Q6AP43 | 4.61 | 38723.78 |
| RPOA_DESVV_1 | A1VE90 | 5.06 | 38922.18 |
| RPOA_RUTMC_1 | A1AVM5 | 4.56 | 35692.56 |
| RPOA_SACD2_1 | Q21M33 | 4.75 | 36299.18 |
| RPOA_HYPNA_1 | Q0BYD8 | 4.82 | 37565.89 |
| RPOA_MAGMM_1 | A0L5Z8 | 4.63 | 37983.97 |
| RPOA_CHRVO_1 | Q7NQH7 | 5.24 | 35971.18 |
| RPOA_BACVZ_1 | A7Z0R5 | 4.79 | 34813.48 |
| RPOA_CLAMS_1 | B0RB72 | 4.71 | 36067.6 |
| RPOA_KORVE_1 | Q1IS93 | 5.13 | 39991.17 |
| RPOA_LACBA_1 | Q03PY3 | 4.82 | 34967.75 |
| RPOA_METC4_1 | B7L0S5 | 4.94 | 37226.57 |
| RPOA_LACGA_1 | Q046A0 | 4.71 | 34825.66 |
| RPOA_BORA1_1 | Q2L238 | 5.52 | 36291.69 |
| RPOA_CLONN_1 | A0PXX5 | 4.74 | 34947.92 |
| RPOA_DESAH_1 | C0Q9U7 | 4.81 | 37890.31 |
| RPOA_BREBN_1 | C0ZIK7 | 4.71 | 34788.48 |
| RPOA_GEOMG_1 | Q39XX9 | 5.26 | 37873.18 |
| RPOA_DESMR_1 | C4XLK3 | 5.06 | 39210.64 |
| RPOA_CALS8_1 | A4XLQ2 | 5.41 | 36267.76 |
| RPOA_LEPIC_1 | Q72NI8 | 4.86 | 36686.64 |
| RPOA_LEUMM_1 | Q03ZM0 | 4.59 | 34239.71 |
| RPOA_HAES1_1 | Q0I137 | 4.98 | 36597.79 |
| RPOA_HAHCH_1 | Q2S937 | 4.8 | 37134.15 |
| RPOA_HALHL_1 | A1WV97 | 4.76 | 36549.3 |
| RPOA_MYXXD_1 | Q1D748 | 5.42 | 37565.91 |
| RPOA_AROAE_1 | Q5P307 | 5.24 | 35630.82 |
| RPOA_AZOC5_1 | A8IAN5 | 4.93 | 37819.33 |
| RPOA_BRUO2_1 | A5VQY2 | 4.85 | 37273.49 |
| RPOA_BUCAI_1 | P57566 | 5.05 | 36855.31 |
| RPOA_CHLCH_1 | Q3APK0 | 4.99 | 37099.33 |
| RPOA_ARTS2_1 | A0JZ49 | 4.68 | 36169.91 |
| RPOA_CHLFF_1 | Q252X4 | 5.33 | 41989.53 |
| RPOA_CHLL2_1 | B3EGW3 | 5.06 | 37266.4 |
| RPOA_CHLL7_1 | Q3B6D6 | 4.91 | 37248.26 |
| RPOA_BACFN_1 | Q5L8D6 | 4.88 | 37444.94 |
| RPOA_BACHK_1 | Q6HPN2 | 4.88 | 34935.82 |
| RPOA_CHLTE_1 | Q8KAJ8 | 5.03 | 36936.07 |
| RPOA_CLOAB_1 | Q97EK6 | 4.94 | 35415.56 |
| RPOA_LACAC_1 | Q5FM65 | 4.85 | 34956.8 |
| RPOA_DECAR_1 | Q47J77 | 5.35 | 35383.79 |
| RPOA_BEII9_1 | B2IF96 | 5.01 | 37411.89 |
| RPOA_METFK_1 | Q1H4L2 | 4.95 | 36101.25 |
| RPOA_BIFAA_1 | A1A095 | 4.58 | 36232.9 |
| RPOA_METPB_1 | B1Z768 | 4.93 | 37186.52 |
| RPOA_CHLP8_1 | B3QR98 | 5.04 | 36859.95 |
| RPOA_LISW6_1 | A0ALU2 | 4.8 | 34905.71 |
| RPOA_HYDS0_1 | B4U772 | 4.87 | 35698.98 |
| RPOA_BACLD_1 | Q65P79 | 4.78 | 34831.54 |
| RPOA_CITK8_1 | A8AQJ0 | 4.97 | 36511.72 |
| RPOA_KLEP7_1 | A6TEU8 | 4.97 | 36465.7 |
| RPOA_BARQU_1 | Q6FZE6 | 4.7 | 37650.97 |
| RPOA_CLOB8_1 | A6LPU0 | 4.95 | 35123.35 |
| RPOA_LACH4_1 | A8YXN0 | 4.77 | 34988.82 |
| RPOA_BIFLO_1 | Q8G3Z3 | 4.58 | 36153.8 |
| RPOA_LACLA_1 | Q9CDY3 | 4.86 | 34194.07 |
| RPOA_BORBU_1 | O51455 | 5.35 | 38553.84 |
| RPOA_BORPD_1 | A9IHR6 | 5.6 | 36264.62 |
| RPOA_CORDI_1 | Q6NJ63 | 4.47 | 36973.69 |
| RPOA_GEOKA_1 | Q5L3R2 | 4.79 | 34822.69 |
| RPOA_BURVG_1 | A4JAR6 | 5.76 | 35696.98 |
| RPOA_CALBD_1 | B9MKF3 | 5.41 | 36323.87 |
| RPOA_GEOTN_1 | A4IJL4 | 4.8 | 34850.7 |
| RPOA_GLUDA_1 | A9H3I9 | 4.89 | 37406.92 |
| RPOA_DICNV_1 | A5EX94 | 4.6 | 35584.37 |
| RPOA_CARRP_1 | Q05FK5 | 9.76 | 37758.68 |
| RPOA_HELHP_1 | Q7VGB9 | 4.93 | 37308.78 |
| RPOA_HERA2_1 | A9B437 | 4.87 | 36736.96 |
| RPOA_PSYWF_1 | A5WCL4 | 4.71 | 37345.54 |
| RPOA_RALPJ_1 | B2UEJ3 | 5.52 | 35540.82 |
| RPOA_RALSO_1 | Q8XV38 | 5.52 | 35531.83 |
| RPOA_EHRCR_1 | Q2GH33 | 5.09 | 41289.22 |
| RPOA_RENSM_1 | A9WSR1 | 4.66 | 36139.85 |
| RPOA_AQUAE_1 | O66483 | 5.15 | 35768.24 |
| RPOA_BRUC2_1 | A9M5M5 | 4.9 | 37399.67 |
| RPOA_AZOSB_1 | A1KB01 | 5.41 | 35732.09 |
| RPOA_BACAN_1 | Q81VQ4 | 4.88 | 34935.82 |
| RPOA_CHLPM_1 | A4SCT6 | 4.91 | 37078.15 |
| RPOA_BRUSU_1 | Q8G094 | 4.9 | 37371.62 |
| RPOA_BARHE_1 | Q9FDC7 | 4.85 | 37672.11 |
| RPOA_BDEBA_1 | Q6MJ36 | 5.18 | 38553.08 |
| RPOA_METCA_1 | Q605D7 | 5.52 | 35828.03 |
| RPOA_BEUC1_1 | C5C0G1 | 4.62 | 36564.32 |
| RPOA_METNO_1 | B8IT35 | 5.06 | 37315.77 |
| RPOA_BLOFL_1 | Q7VQC3 | 5.19 | 37157.76 |
| RPOA_CLOCE_1 | B8I808 | 4.77 | 34940.8 |
| RPOA_METSB_1 | B8EIT5 | 5.11 | 37166.56 |
| RPOA_DESAD_1 | C6C1B1 | 4.98 | 39267 |
| RPOA_CLOTH_1 | A3DJK1 | 5.01 | 35039.18 |
| RPOA_BRADU_1 | Q89JA7 | 4.9 | 37944.34 |
| RPOA_DESAP_1 | B1I1B3 | 5 | 35370.63 |
| RPOA_DESDA_1 | B8IYL7 | 5.23 | 38883.44 |
| RPOA_BURTA_1 | Q2SU53 | 5.76 | 35654.94 |
| RPOA_CAMFF_1 | A0RM36 | 4.81 | 37336.67 |
| RPOA_CAMHC_1 | A7HZX1 | 4.92 | 37924.42 |
| RPOA_GLUOX_1 | Q5FU07 | 4.87 | 37289.87 |
| RPOA_GRABC_1 | Q0BUM6 | 4.83 | 37511.9 |
| RPOA_CUPMC_1 | Q1LI63 | 5.49 | 35600.85 |
| RPOA_BACCN_1 | A7GK47 | 4.84 | 34947.83 |
| RPOA_IDILO_1 | Q5QXV8 | 4.59 | 36250.06 |
| RPOA_MACCJ_1 | B9E9L7 | 4.71 | 35059.93 |
| RPOA_MAGSA_1 | Q2W2L3 | 4.9 | 37453.02 |
| RPOA_JANSC_1 | Q28US8 | 4.64 | 37150.17 |
| RPOA_BACV8_1 | A6KYH0 | 4.88 | 37533.02 |
| RPOA_BURM1_1 | A9ADL9 | 5.63 | 35669.95 |
| RPOA_DELAS_1 | A9BRX5 | 5.96 | 36294.49 |
| RPOA_BORAP_1 | Q0SN05 | 5.19 | 38647.88 |
| RPOA_BORBR_1 | P0A4E6 | 5.6 | 36158.54 |
| RPOA_CLOTE_1 | Q890R0 | 4.88 | 35343.74 |
| RPOA_LARHH_1 | C1DAU3 | 5.12 | 36074.39 |
| RPOA_COLP3_1 | Q488Y8 | 4.69 | 36152.24 |
| RNA Polymerase subunit beta | Accession number | pI | Molecular Weight |
| RPOB_ECOLI_1 | P0A8V2 | 5.14 | 150632.4 |
| RPOB_MYCTU_1 | P9WGY9 | 4.92 | 129865 |
| RPOB_MYCS2_1 | P60281 | 4.88 | 128531.3 |
| RPOB_RICPR_1 | O52271 | 5.87 | 154582.9 |
| RPOB_LISMO_1 | Q9RLT9 | 5.05 | 132604 |
| RPOB_THET8_1 | Q8RQE9 | 5.71 | 125264.5 |
| RPOB_STAA8_1 | P47768 | 4.91 | 133219.5 |
| RPOB_THEAQ_1 | Q9KWU7 | 5.73 | 124758 |
| RPOB_RICTY_1 | P77941 | 5.91 | 154647 |
| RPOB_SALTY_1 | P06173 | 5.14 | 150601.1 |
| RPOB_NEIMB_1 | Q59622 | 5.34 | 155709.5 |
| RPOB_LEGPN_1 | O86094 | 5.4 | 152752.1 |
| RPOB2_NOCFA_1 | Q5YPE0 | 4.98 | 128606.9 |
| RPOB_SHEAM_1 | A1S211 | 5.25 | 150175.8 |
| RPOB_SHEPC_1 | A4YBZ0 | 5.25 | 150213 |
| RPOB_SHESA_1 | A0KRL7 | 5.25 | 150274 |
| RPOB_SHEWM_1 | B1KMZ0 | 5.15 | 149895.4 |
| RPOB_SHIBS_1 | Q31U10 | 5.14 | 150632.4 |
| RPOB_SINMW_1 | A6U851 | 4.98 | 153338.5 |
| RPOB1_ENTFC_1 | Q8GCR6 | 4.92 | 135021.5 |
| RPOB_ACHLI_1 | A9NEL7 | 5.71 | 141438.9 |
| RPOB_ACIAC_1 | A1TVT0 | 5.76 | 153099.3 |
| RPOB_ACIBT_1 | A3M1G3 | 5.22 | 151323.9 |
| RPOB_ACISJ_1 | A1WCM9 | 5.73 | 153288.5 |
| RPOB_RUEST_1 | Q1GK49 | 4.93 | 153832.7 |
| RPOB_SACEN_1 | A4FPP3 | 4.89 | 128683.4 |
| RPOB_SALA4_1 | B5F0W7 | 5.14 | 150571.1 |
| RPOB_SALEP_1 | B5QYD8 | 5.14 | 150571.1 |
| RPOB_SALHS_1 | B4TCS4 | 5.14 | 150571.1 |
| RPOB_SALPA_1 | Q5PK93 | 5.14 | 150571.1 |
| RPOB_SHEVI_1 | Q9KW14 | 5.28 | 150103.8 |
| RPOBC_HELPH_1 | Q1CS68 | 6.69 | 323862.5 |
| RPOB_ACIF2_1 | B7J460 | 5.42 | 151761.9 |
| RPOB_ROSS1_1 | A5USR7 | 5.45 | 137726.9 |
| RPOB_RUBXD_1 | Q1AU22 | 5.11 | 127102.7 |
| RPOB_SALG2_1 | B5RFK1 | 5.15 | 150511.1 |
| RPOB_SALNS_1 | B4T0Y9 | 5.14 | 150571.1 |
| RPOB_SERP5_1 | A8G8E7 | 5.17 | 150339.1 |
| RPOB_SHEB2_1 | B8EBL2 | 5.29 | 150019.9 |
| RPOB_SHIFL_1 | P0A8V5 | 5.14 | 150632.4 |
| RPOB_SODGM_1 | Q2NWR6 | 5.13 | 150366 |
| RPOBC_HELAH_1 | Q17VN6 | 6.6 | 323895.7 |
| RPOBC_WOLPM_1 | Q73IW9 | 6.02 | 317782.4 |
| RPOB_SHEDO_1 | Q12SW6 | 5.2 | 150123 |
| RPOB_SHEFN_1 | Q089R1 | 5.21 | 149935.7 |
| RPOB_SHELP_1 | A3Q975 | 5.14 | 149948.5 |
| RPOB_SHESH_1 | A8G1F5 | 5.15 | 149877.2 |
| RPOB_SHISS_1 | Q3YUZ7 | 5.14 | 150632.4 |
| RPOB_SINFN_1 | C3MAX2 | 5.01 | 153441.6 |
| RPOBC_WOLSU_1 | Q7MA56 | 6.38 | 321723.6 |
| RPOB_ACIAD_1 | Q6FF90 | 5.21 | 151484.1 |
| RPOB_ACICJ_1 | A5FZX1 | 5.31 | 155922.6 |
| RPOB_SACD2_1 | Q21M93 | 5.22 | 151592.3 |
| RPOB_SALAR_1 | A9MHF1 | 5.14 | 150571.1 |
| RPOB_SHEON_1 | Q8EK74 | 5.27 | 150295.1 |
| RPOB_SHEPA_1 | A8GYW9 | 5.2 | 149714.5 |
| RPOB_SHIDS_1 | Q32AF9 | 5.15 | 150646.4 |
| RPOB_RUEPO_1 | Q5LMQ5 | 5.03 | 153272.5 |
| RPOB_RUTMC_1 | A1AX75 | 6.65 | 151531.2 |
| RPOB_SALCH_1 | Q57H69 | 5.14 | 150587.2 |
| RPOB_SALDC_1 | B5FQJ9 | 5.14 | 150571.1 |
| RPOB_SALSV_1 | B4TQJ5 | 5.14 | 150571.1 |
| RPOB_SALTI_1 | Q8Z320 | 5.14 | 150631.2 |
| RPOB_SHEPW_1 | B8CNC5 | 5.12 | 149664.2 |
| RPOBC_HELHP_1 | Q7VJ82 | 7.05 | 324320.5 |
| RPOBC_WOLTR_1 | Q5GRY9 | 6.21 | 318102.2 |
| RPOB_ACIC1_1 | A0LRL3 | 5.12 | 129702 |
| RPOB_ACIET_1 | B9MH47 | 5.73 | 153301.6 |
| RPOB_BACCZ_1 | Q63H98 | 4.99 | 131893.5 |
| RPOB_ERYLH_1 | Q2N5Q7 | 5.03 | 154475.9 |
| RPOB_PSEP1_1 | A5VXP0 | 5.56 | 150992.6 |
| RPOB_LISW6_1 | A0AF63 | 5.05 | 132589.9 |
| RPOB_AERHH_1 | A0KQA5 | 5.26 | 150227 |
| RPOB_MYXXD_1 | Q1D7U3 | 5.56 | 157532.1 |
| RPOB_CLAM3_1 | A5CUC7 | 5.02 | 128598.6 |
| RPOB_JANSC_1 | Q28UX7 | 4.96 | 154187.2 |
| RPOB_AGRRK_1 | B9JDS1 | 4.98 | 153605 |
| RPOB_CLOB6_1 | C3KVQ9 | 4.85 | 140102.7 |
| RPOB_AGRVS_1 | B9JVM8 | 5.02 | 153824.1 |
| RPOB_FRATH_1 | Q2A1M7 | 5.47 | 151335.7 |
| RPOB_MESFL_1 | Q6F0L7 | 5.05 | 142776.9 |
| RPOB_ALISL_1 | B6ENR3 | 5.12 | 150134.6 |
| RPOB_BIFAA_1 | A1A317 | 4.9 | 131477.9 |
| RPOB_LACLA_1 | Q9CEN6 | 5.01 | 133153.6 |
| RPOB_POLSJ_1 | Q123G3 | 5.68 | 152528.8 |
| RPOB_METRJ_1 | B1LY43 | 5.18 | 153094.5 |
| RPOB_NOVAD_1 | Q2GCD7 | 5.06 | 154132.8 |
| RPOB_CAMLR_1 | B9KFG7 | 6.32 | 155941.4 |
| RPOB_BUCBP_1 | Q89B20 | 8.87 | 151550 |
| RPOB_ACTP2_1 | A3N325 | 5.18 | 149710.1 |
| RPOB_DEHMC_1 | Q3ZX01 | 5.18 | 141526 |
| RPOB_MYCUA_1 | A0PM24 | 4.99 | 129489.5 |
| RPOB_BURCC_1 | B1JU14 | 5.46 | 153279.7 |
| RPOB_AERS4_1 | A4SHU9 | 5.27 | 150252.1 |
| RPOB_LYSSC_1 | B1HMZ6 | 4.96 | 133879.5 |
| RPOB_FRAAA_1 | Q0RRT0 | 4.98 | 126703.4 |
| RPOB_BURM7_1 | A3MRU5 | 5.46 | 153150.5 |
| RPOB_CLOB8_1 | A6LPQ4 | 4.83 | 138830.4 |
| RPOB_BURP0_1 | A3P0C5 | 5.46 | 153150.5 |
| RPOB_LACGA_1 | Q046D2 | 5.4 | 136060.1 |
| RPOB_CLOP1_1 | Q0TMN8 | 4.85 | 138550.1 |
| RPOB_CALS4_1 | Q8R7U6 | 4.79 | 138644.2 |
| RPOB_METI4_1 | B3E163 | 6.04 | 144000.4 |
| RPOB_GEOBB_1 | B5EFP3 | 5.47 | 152912.8 |
| RPOB_CAMJE_1 | Q46124 | 6.34 | 155915.6 |
| RPOB_ALKOO_1 | A8MLD2 | 4.8 | 139082.4 |
| RPOB_LACRD_1 | A5VLL4 | 5.01 | 135051 |
| RPOB_COREF_1 | Q8FS97 | 4.9 | 129483.3 |
| RPOB_BORBR_1 | Q7WRD9 | 5.5 | 153122.9 |
| RPOB_GEOTN_1 | A4IJI1 | 5.05 | 133412.4 |
| RPOB_RENSM_1 | A9WSY0 | 4.9 | 129264 |
| RPOB_PROM1_1 | A2C4N2 | 5.53 | 122570.2 |
| RPOB_CAUVN_1 | B8GZW7 | 5.25 | 150890.1 |
| RPOB_CELJU_1 | B3PK30 | 5.39 | 151555.4 |
| RPOB_COXBR_1 | A9NAL4 | 5.81 | 153570.3 |
| RPOB_CHLAA_1 | A9WH12 | 5.57 | 137772 |
| RPOB_LEPBL_1 | Q054E2 | 5.6 | 137683.3 |
| RPOB_CUPNJ_1 | Q46WD4 | 5.57 | 152559.5 |
| RPOB_BRUA1_1 | B2S687 | 5.03 | 153668.8 |
| RPOB_CHLCV_1 | Q822J1 | 5.54 | 140261.1 |
| RPOB_PSEA6_1 | Q15YB1 | 5.09 | 149786.3 |
| RPOB_LEUMM_1 | Q03V60 | 4.96 | 134288.5 |
| RPOB_PARPJ_1 | B2T759 | 5.48 | 153057.3 |
| RPOB_BRUO2_1 | A5VR15 | 5.03 | 153668.8 |
| RPOB_STRU0_1 | B9DSZ5 | 4.94 | 132664.7 |
| RPOB_RHOS4_1 | Q3J5T0 | 5.14 | 153818 |
| RPOB_SYNSC_1 | Q3AHX5 | 5.36 | 122559 |
| RPOB_SYNY3_1 | P77965 | 5.17 | 123364.6 |
| RPOB_XANOM_1 | Q2NZX8 | 5.45 | 154272.2 |
| RPOB_ANAMM_1 | Q5PBG4 | 5.44 | 154522.1 |
| RPOB_STRP3_1 | P0DF30 | 5 | 132854.9 |
| RPOB_THEM4_1 | A6LKB8 | 5.93 | 132745.5 |
| RPOB_AYWBP_1 | Q2NJ15 | 6.08 | 143614.7 |
| RPOB_THEP3_1 | B0KCJ2 | 4.77 | 138915.7 |
| RPOB_YERPG_1 | A9R0H8 | 5.16 | 150399.2 |
| RPOB_CHRSD_1 | Q1R0I2 | 5.15 | 151639.1 |
| RPOB_DEIGD_1 | Q1J0P6 | 5.2 | 127383.9 |
| RPOB_DELAS_1 | A9BR98 | 5.7 | 152887.1 |
| RPOB_JANMA_1 | A6T3L3 | 5.63 | 152387.4 |
| RPOB_MAGMM_1 | A0L5W6 | 5.38 | 152023.2 |
| RPOB_MAGSA_1 | Q2W2I1 | 5.49 | 154339.2 |
| RPOB_KLEP3_1 | B5XYF5 | 5.17 | 150502.1 |
| RPOB_DESHY_1 | Q250P0 | 4.91 | 125187.8 |
| RPOB_PHOLL_1 | Q7N9A4 | 5.16 | 150606.3 |
| RPOB_MARMS_1 | A6W399 | 5.34 | 151755.7 |
| RPOB_PHYAS_1 | B1VAM6 | 6.52 | 140223.1 |
| RPOB_LACDA_1 | Q1GBM5 | 4.93 | 135843.6 |
| RPOB_PSYCK_1 | Q1Q8P9 | 5.1 | 152577.2 |
| RPOB_NITHX_1 | Q1QN44 | 5.62 | 154219 |
| RPOB_FUSNN_1 | Q8RHI6 | 5.03 | 133125.7 |
| RPOB_CLOTE_1 | Q890N4 | 4.84 | 138903.5 |
| RPOB_COLP3_1 | Q47UV9 | 5.41 | 149617.7 |
| RPOB_RALPJ_1 | B2UEN6 | 5.66 | 152264.4 |
| RPOB_LACS1_1 | Q1WVA5 | 5.07 | 134609.3 |
| RPOB_GEOSM_1 | C6E4R4 | 5.46 | 152899.8 |
| RPOB_CORJK_1 | Q4JT32 | 4.74 | 129103.5 |
| RPOB_GEOUR_1 | A5GAY1 | 5.66 | 153157.7 |
| RPOB_OENOB_1 | Q04E85 | 4.95 | 133224.7 |
| RPOB_RHIL3_1 | Q1MIE9 | 5 | 153395.5 |
| RPOB_BREBN_1 | C0ZIH0 | 5.26 | 131794 |
| RPOB_LEPIC_1 | Q72UA8 | 5.6 | 137767.3 |
| RPOB_MYCHJ_1 | Q4A969 | 6.04 | 137714.5 |
| RPOB_LEUCK_1 | B1MVW8 | 4.92 | 134454.6 |
| RPOB_HAEPS_1 | B8F741 | 5.17 | 149536.8 |
| RPOB_PARUW_1 | Q6MDM1 | 5.48 | 140972 |
| RPOB_BRUSI_1 | B0CH41 | 5.03 | 153668.8 |
| RPOB_PSEE4_1 | Q1IFX3 | 5.57 | 150754.3 |
| RPOB_TROWH_1 | P59643 | 5.18 | 127924.1 |
| RPOB_STRT2_1 | Q5M2F5 | 5.02 | 133260.6 |
| RPOB_SULDN_1 | Q30TP7 | 5.9 | 156404.6 |
| RPOB_VIBCH_1 | Q9KV30 | 5.14 | 149454.9 |
| RPOC_BORBU_1 | O51349 | 8.18 | 154658.2 |
| RPOB_RICRS_1 | A8GQW4 | 6.07 | 154257.8 |
| RPOC_DESVH_1 | Q727C6 | 6.67 | 154813.2 |
| RPOB_STRA5_1 | Q8E239 | 4.94 | 133011 |
| RPOB_ANAPZ_1 | Q2GJ68 | 5.31 | 154522.9 |
| RPOB_STRGC_1 | A8AZI3 | 5.02 | 132684.8 |
| RPOB_BACAN_1 | Q81VT8 | 4.99 | 131891.5 |
| RPOB_TOLAT_1 | C4LBV2 | 5.18 | 149813.5 |
| RPOB_CHLTE_1 | Q8KG15 | 5.31 | 145469.1 |
| RPOB_BURA4_1 | B1YRC2 | 5.48 | 153254.7 |
| RPOB_PELUB_1 | Q4FLL2 | 5.46 | 152477.9 |
| RPOB_PSESM_1 | Q889X8 | 5.56 | 151044.7 |
| RPOB_DESAG_1 | Q30X05 | 5.52 | 154036.5 |
| RPOB_MARHV_1 | A1TYJ0 | 5.22 | 151419.2 |
| RPOB_NEOSE_1 | Q93MK9 | 5.49 | 151550.6 |
| RPOB_LACBA_1 | Q03PU9 | 4.97 | 134300.4 |
| RPOB_BAUCH_1 | Q1LSX7 | 6.03 | 151516.5 |
| RPOB_NITEC_1 | Q0AF53 | 5.87 | 151907.9 |
| RPOB_PSYWF_1 | A5WH35 | 5.03 | 152908.3 |
| RPOB_ALKEH_1 | Q0ABI2 | 5.16 | 150618.9 |
| RPOB_GEOKA_1 | Q5L405 | 5.05 | 133489.6 |
| RPOB_CORDI_1 | P60280 | 4.84 | 129791.4 |
| RPOB_PORCN_1 | Q9F3X8 | 5.32 | 142623.3 |
| RPOB_PORG3_1 | B2RL45 | 5.58 | 142381.1 |
| RPOB_OCEIH_1 | Q8ETY8 | 4.92 | 132269.4 |
| RPOB_CORK4_1 | C4LL71 | 4.87 | 129067.5 |
| RPOB_CHESB_1 | Q11HB3 | 5.02 | 154227.5 |
| RPOB_VESOH_1 | A5CW25 | 7.63 | 152158 |
| RPOB_SYNAS_1 | Q2LQ87 | 5.37 | 153161.1 |
| RPOB_RICAH_1 | A8GMA7 | 6.14 | 154163.7 |
| RPOB_RICBR_1 | Q1RHD0 | 6.03 | 153845 |
| RPOB_ANADF_1 | A7HCH7 | 5.53 | 158370 |
| RPOB_TREPA_1 | O83269 | 5.74 | 132446.8 |
| RPOB_DECAR_1 | Q47JB0 | 5.62 | 158061.4 |
| RPOB_BACFR_1 | Q64NJ7 | 5.31 | 142453.5 |
| RPOB_ACTSZ_1 | A6VKC5 | 5.2 | 149313.8 |
| RPOB_BACHK_1 | Q6HPR6 | 4.99 | 131893.5 |
| RPOB_MYCVP_1 | A1T4J2 | 4.86 | 128489.3 |
| RPOB_EXISA_1 | C4KZQ4 | 4.97 | 131840.4 |
| RPOB_FLAJ1_1 | A5FIJ3 | 5.33 | 142684.4 |
| RPOB_CLOAB_1 | Q97EG9 | 4.93 | 139248.9 |
| RPOB_NEIG2_1 | B4RQW2 | 5.37 | 155718.6 |
| RPOB_DESDA_1 | B8J1A8 | 5.46 | 154129.9 |
| RPOB_DESMR_1 | C4XIN9 | 5.54 | 152835 |
| RPOB_NEORS_1 | Q93MK8 | 5.56 | 152511.9 |
| RPOB_BARQU_1 | Q9KJM5 | 5.1 | 154830.5 |
| RPOB_PHYMT_1 | B3QZH0 | 8.52 | 144953.7 |
| RPOB_METCA_1 | Q60A06 | 5.46 | 151759 |
| RPOB_CAMFF_1 | A0RQI5 | 5.87 | 156298.6 |
| RPOB_NITSB_1 | A6Q1M3 | 5.82 | 157341.1 |
| RPOB_CLOTH_1 | A3DIZ4 | 4.76 | 139955.6 |
| RPOB_ALKMQ_1 | A6TWJ0 | 4.82 | 139728.8 |
| RPOB_NOCSJ_1 | A1SEK1 | 4.91 | 129879 |
| RPOB_GEOLS_1 | B3E7S8 | 5.67 | 167393.7 |
| RPOB_RALSO_1 | Q8XUZ8 | 5.59 | 152428.3 |
| RPOB_RHIEC_1 | Q2K9M4 | 5.02 | 153419.6 |
| RPOB_MYCGI_1 | A4T1P4 | 4.87 | 129230.1 |
| RPOB_CHLL7_1 | Q3B1H7 | 5.33 | 145415.7 |
| RPOB_MYCLB_1 | B8ZSC7 | 4.96 | 129728 |
| RPOB_RHOBA_1 | Q7URW6 | 5.23 | 138006.8 |
| RPOB_MYCMO_1 | Q6KI09 | 7.95 | 132457.3 |
| RPOB_MYCMS_1 | Q6MRX6 | 4.97 | 144828.5 |
| RPOB_HAMD5_1 | C4K4F1 | 5.37 | 150446.5 |
| RPOB_PASMU_1 | Q9CK91 | 5.2 | 149567 |
| RPOB_PSEFS_1 | C3K2Y3 | 5.66 | 151017.7 |
| RPOB_RHOOB_1 | C1AYV9 | 4.91 | 128199 |
| RPOB_PSEMY_1 | A4XZ97 | 5.47 | 150752 |
| RPOB_MYCPU_1 | Q98Q23 | 8.38 | 136663.4 |
| RPOB_RHOPS_1 | Q134R8 | 5.48 | 153491.2 |
| RPOB_TERTT_1 | C5BQ39 | 5.17 | 151312.7 |
| RPOC_RICCN_1 | Q9RH40 | 8.03 | 153244.6 |
| RPOB_ZYMMO_1 | Q5NPK5 | 5.13 | 156539.7 |
| RPOB_PEDPA_1 | Q03EA9 | 4.96 | 134619.9 |
| RPOB_MACCJ_1 | B9E8Q5 | 4.94 | 132348.1 |
| RPOB_BURL3_1 | Q39KH5 | 5.46 | 153356.8 |
| RPOB_FRASN_1 | A8LC64 | 4.99 | 126613.3 |
| RPOB_BARBK_1 | A1USC8 | 5.19 | 154846.6 |
| RPOB_KOSOT_1 | C5CGD9 | 5.89 | 131840.4 |
| RPOB_DESPS_1 | Q6AP78 | 5.28 | 151635.2 |
| RPOB_BURTA_1 | Q2SU19 | 5.48 | 153149.6 |
| RPOB_CLOK1_1 | B9DYA1 | 4.95 | 139107.5 |
| RPOB_BDEBA_1 | Q6MJ09 | 5.37 | 157078.1 |
| RPOB_NITEU_1 | Q82T75 | 5.85 | 151795.7 |
| RPOB_BIFLD_1 | B3DTE2 | 4.88 | 131643.8 |
| RPOB_NITMU_1 | Q2YB05 | 5.72 | 150941.1 |
| RPOB_BORA1_1 | Q2L2M3 | 5.49 | 153499.7 |
| RPOB_CARHZ_1 | Q3A9Q7 | 5.06 | 127899.5 |
| RPOB_LACSS_1 | Q38UQ2 | 4.92 | 133762 |
| RPOB_CORU7_1 | B1VES1 | 4.73 | 129574.1 |
| RPOB_BORHD_1 | B2S092 | 5.65 | 129995 |
| RPOB_BRADU_1 | Q89J74 | 5.59 | 153276.2 |
| RPOB_LEPBP_1 | B0SSI4 | 5.86 | 137912.9 |
| RPOB_HAEIN_1 | P43738 | 5.19 | 149784 |
| RPOB_PARP8_1 | B2JIH4 | 5.51 | 153039.4 |
| RPOB_BRUME_1 | Q8YHP8 | 5.03 | 153668.8 |
| RPOB_CHLPN_1 | Q9Z9A0 | 5.57 | 140109.9 |
| RPOB_RHOE4_1 | C0ZVQ6 | 4.9 | 128340.3 |
| RPOB_PECAS_1 | Q6DAN0 | 5.23 | 150614.4 |
| RPOB_HERAR_1 | A4G9U5 | 5.62 | 152432.5 |
| RPOB_MYCS5_1 | Q4A5S7 | 6.45 | 135227.6 |
| RPOB_STRSY_1 | A4VSK2 | 5.05 | 132870.1 |
| RPOB_UREP2_1 | B1AIH5 | 5.3 | 162099.2 |
| RPOB_UREU1_1 | B5ZAZ3 | 5.3 | 162186.4 |
| RPOB_SULMW_1 | A8Z5T3 | 9.66 | 153022 |
| RPOB_VIBVU_1 | Q8DD20 | 5.11 | 149619.1 |
| RPOB_RICFE_1 | Q4UKD4 | 6.05 | 154331.7 |
| RPOB_XANAC_1 | Q8PNT0 | 5.46 | 154198.2 |
| RPOB_XANCB_1 | B0RU89 | 5.35 | 154854.9 |
| RPOB_RICM5_1 | A8F0P7 | 6.11 | 154292.9 |
| RPOB_ROSDO_1 | Q160X7 | 4.97 | 153395.3 |
| RPOB_KINRD_1 | A6W5T0 | 4.91 | 129224.1 |
| RPOB_BACVZ_1 | A7Z0M9 | 4.94 | 133820.4 |
| RPOB_PSEU5_1 | A4VHM3 | 5.56 | 150970.6 |
| RPOB_LACCB_1 | B3WAM8 | 5.25 | 133700.2 |
| RPOB_METC4_1 | B7KN45 | 5.2 | 153042.4 |
| RPOB_PSYIN_1 | A1T065 | 5.15 | 150448.3 |
| RPOB_CALS8_1 | A4XI30 | 4.96 | 138333.1 |
| RPOB_GEODF_1 | B9M6V2 | 5.68 | 153061.3 |
| RPOB_NITWN_1 | Q3SSY0 | 5.54 | 154232.2 |
| RPOB_GEOMG_1 | Q39Y13 | 5.52 | 152878.8 |
| RPOB_CORGL_1 | Q8NT26 | 4.86 | 128785.3 |
| RPOB_CARRP_1 | Q05FH8 | 9.78 | 149254.7 |
| RPOB_MYCBP_1 | A1KGE7 | 4.92 | 129865 |
| RPOB_MYCCT_1 | Q2ST48 | 5.02 | 144569.3 |
| RPOB_GLUOX_1 | Q5FTX7 | 5.19 | 155016.2 |
| RPOB_BORPA_1 | Q7W2G9 | 5.5 | 153082.9 |
| RPOB_ONYPE_1 | Q6YQW3 | 6.17 | 143378.4 |
| RPOB_LEIXX_1 | Q6ACX5 | 4.9 | 128136 |
| RPOB_BORRA_1 | B5RRJ7 | 5.74 | 130087 |
| RPOB_MYCGE_1 | P47583 | 6.35 | 156330.6 |
| RPOB_GRAFK_1 | A0M3Y9 | 5.4 | 142739.4 |
| RPOB_CHLAB_1 | Q5L5I3 | 5.62 | 140335.3 |
| RPOB_CUPTR_1 | B3R7T6 | 5.54 | 152631.5 |
| RPOB_VIBPA_1 | Q87KQ4 | 5.1 | 149552.9 |
| RPOB_SYNFM_1 | A0LII4 | 5.48 | 153980.5 |
| RPOB_STRAW_1 | Q82DQ5 | 4.85 | 128296.9 |
| RPOB_AQUPY_1 | Q9X6Y1 | 6.65 | 167301.5 |
| RPOB_YERE8_1 | A1JII0 | 5.11 | 150459.2 |
| RPOB_CHLTB_1 | B0BBU6 | 5.63 | 140057.7 |
| RPOB_HYDCU_1 | Q31IY9 | 5.16 | 150207.9 |
| RPOB_BACLD_1 | Q65PB5 | 4.94 | 133692.2 |
| RPOB_DEIRA_1 | Q9RVV9 | 5.21 | 128735.3 |
| RPOB_DESAA_1 | B8FEU1 | 5.5 | 152604.9 |
| RPOB_FLAPJ_1 | A6GYU0 | 5.44 | 142492.3 |
| RPOB_BACTN_1 | Q8A469 | 5.29 | 142487.6 |
| RPOB_MANSM_1 | Q65W41 | 5.19 | 149638.2 |
| RPOB_BACWK_1 | A9VP69 | 5 | 131782.4 |
| RPOB_DESOH_1 | A8ZV51 | 6.35 | 152065.5 |
| RPOB_ALCBS_1 | Q0VSM2 | 5.05 | 154611.2 |
| RPOB_ALIF1_1 | Q5E238 | 5.07 | 149982.4 |
| RPOB_CLONN_1 | A0PXT8 | 4.84 | 139720.4 |
| RPOB_DICNV_1 | A5EX70 | 5.42 | 152468 |
| RPOB_METFK_1 | Q1H4P4 | 5.34 | 155365.1 |
| RPOB_NITOC_1 | Q3J8Q7 | 5.35 | 151135.5 |
| RPOB_POLNS_1 | B1XSP3 | 5.51 | 152450.4 |
| RPOB_BLOFL_1 | Q7VRP7 | 6.15 | 151236.7 |
| RPOB_METS4_1 | B0UHX6 | 5.26 | 152952.4 |
| RPOB_BORBP_1 | Q661M9 | 5.73 | 129675.4 |
| RPOB_LAWIP_1 | Q1MPW8 | 5.42 | 153862.8 |
| RPOB_ORITB_1 | A5CC93 | 5.67 | 153662.3 |
| RPOB_BORT9_1 | A1QZH7 | 5.62 | 130066 |
| RPOB_CHLAD_1 | B8G4U9 | 5.67 | 137745 |
| RPOB_PSE14_1 | Q48D29 | 5.56 | 151003.7 |
| RPOB_CHLFF_1 | Q255E6 | 5.51 | 140346.1 |
| RPOB_PARDP_1 | A1B015 | 5.04 | 153614.4 |
| RPOB_BRUC2_1 | A9M5R0 | 5.03 | 153682.8 |
| RPOB_CHLMU_1 | P56869 | 5.56 | 139965.5 |
| RPOB_MYCMM_1 | B2HSJ3 | 4.98 | 129547.5 |
| RPOB_RHOCS_1 | B6IRP6 | 5.35 | 156348.3 |
| RPOB_CYTH3_1 | Q11QA5 | 5.62 | 145289.5 |
| RPOB_RHOJR_1 | Q0SFB3 | 4.91 | 128316.2 |
| RPOB_PECCP_1 | C6DHR5 | 5.18 | 150668.4 |
| RPOB_ENT38_1 | A4W5A7 | 5.12 | 150473.2 |
| RPOB_PSELT_1 | A8F4G0 | 5.96 | 131439 |
| RPOB_SORC5_1 | A9GRB1 | 5.39 | 155045.9 |
| RPOB_RICAE_1 | C3PMH7 | 6.07 | 154370.9 |
| RPOB_VIBCB_1 | A7MXF1 | 5.12 | 149654 |
| RPOB_SYMTH_1 | Q67JT3 | 4.75 | 139531.8 |
| RPOB_RICCK_1 | A8EXK8 | 6.04 | 154182.6 |
| RPOB_XANP2_1 | A7IKQ0 | 5.21 | 151769.2 |
| RPOB_THESQ_1 | B1L935 | 5.24 | 143190.6 |
| RPOB_BACP2_1 | A8F976 | 4.94 | 133963.7 |
| RPOB_BACSK_1 | Q5WLS0 | 5.03 | 132501.2 |
| RPOB_PETMO_1 | A9BF33 | 5.45 | 134955.8 |
| RPOB_BURM1_1 | A9ADI5 | 5.48 | 153215.6 |
| RPOB_BACV8_1 | A6KYK3 | 5.34 | 142484.7 |
| RPOB_FRAP2_1 | B0TX10 | 5.57 | 151453.8 |
| RPOB_PHEZH_1 | B4R8K5 | 5.12 | 151282.2 |
| RPOB_MARMM_1 | Q0ANP3 | 5.21 | 150305.7 |
| RPOB_PHOPR_1 | Q6LLW2 | 5.25 | 149774.2 |
| RPOB_DESRM_1 | A4J103 | 4.93 | 129390.4 |
| RPOB_DINSH_1 | A8LM40 | 4.93 | 153055.7 |
| RPOB_METNO_1 | B8IS78 | 5.2 | 152881 |
| RPOB_METPP_1 | A2SLG5 | 5.55 | 152766.8 |
| RPOB_LACPL_1 | Q88XZ3 | 4.97 | 134501.9 |
| RPOB_MOOTA_1 | Q2RFN9 | 4.86 | 127976.5 |
| RPOB_GEOSL_1 | Q748Y6 | 5.5 | 152507.6 |
| RPOB_MYCA1_1 | A0QL49 | 4.93 | 129759.8 |
| RPOB_OCHA4_1 | A6X0A9 | 4.99 | 153536.5 |
| RPOB_GLOVI_1 | Q7NIA0 | 5.41 | 124478.5 |
| RPOB_MYCGA_1 | P47715 | 5.69 | 156053 |
| RPOB_BRASB_1 | A5ELN7 | 5.6 | 153408.4 |
| RPOB_RHILO_1 | Q98N66 | 5.23 | 153901.5 |
| RPOB_PSEA7_1 | A6UZI1 | 5.61 | 150866.5 |
| RPOB_HAES1_1 | Q0I5B7 | 5.27 | 149894.4 |
| RPOB_EHRCR_1 | Q8KWX2 | 5.21 | 155604.8 |
| RPOB_LISIN_1 | Q92F22 | 5.04 | 132605.9 |
| RPOB_MYCPE_1 | Q8EWX1 | 6.41 | 154732.6 |
| RPOB_HELMI_1 | B0TC48 | 4.98 | 129582.1 |
| RPOB_HERA2_1 | A9B6J3 | 5.39 | 135538.7 |
| RPOB_TRIEI_1 | Q110H1 | 5.5 | 123703.5 |
| RPOB_MYCSS_1 | Q1BDF0 | 4.9 | 129092.9 |
| RPOB_STRSV_1 | A3CKD3 | 5.05 | 132610.8 |
| RPOB_VEREI_1 | A1WK55 | 5.96 | 153029.6 |
| RPOB_SULNB_1 | A6Q6I2 | 5.5 | 156769.7 |
| RPOB_STACT_1 | B9DKV0 | 4.88 | 133212.2 |
| RPOB_STAS1_1 | Q49V52 | 4.84 | 132904 |
| RPOB_THEFY_1 | Q47LI5 | 5.08 | 128814.1 |
| RPOB_THEMA_1 | P29398 | 5.26 | 143138.4 |
| RPOB_CAUSK_1 | B0SUP7 | 5.18 | 151127.3 |
| RPOB_BORDL_1 | B5RLU9 | 5.74 | 130073 |
| RPOB_BORPD_1 | A9IJ25 | 5.43 | 153390.4 |
| RPOB_BORPE_1 | Q7W0R9 | 5.5 | 153153 |
| RPOB_CROS8_1 | A7MQQ9 | 5.14 | 150471.2 |
| RPOB_GRABC_1 | Q0BUQ6 | 5.31 | 154582.9 |
| RPOB_PROMH_1 | B4EYU9 | 5.11 | 150279.8 |
| RPOB_CUPMC_1 | Q1LI20 | 5.57 | 152496.3 |
| RPOB_CHLCH_1 | Q3ATP5 | 5.46 | 145666.2 |
| RPOB_CUTAK_1 | Q6A6K6 | 4.97 | 128430.2 |
| RPOB_RHIME_1 | Q92QH7 | 4.98 | 153517.7 |
| RPOB_EDWI9_1 | C5BHE3 | 5.12 | 150610.4 |
| RPOB_CHLPD_1 | A1BD23 | 5.28 | 147001.7 |
| RPOB_EHRRG_1 | Q5FFD9 | 5.23 | 155947.5 |
| RPOB_MYCPA_1 | Q73SE4 | 4.93 | 129759.8 |
| RPOB_MYCPN_1 | P78013 | 6.06 | 155621.6 |
| RPOB_PSEHT_1 | Q3ILP9 | 5.17 | 149686 |
| RPOB_ENTFA_1 | Q82Z40 | 4.92 | 135187.9 |
| RPOB_SOLUE_1 | Q01VB1 | 6.34 | 160437.1 |
| RPOB_SPHAL_1 | Q1GT23 | 4.98 | 154114.8 |
| RPOB_SPICI_1 | P47767 | 5.08 | 146533.9 |
| RPOB_RHORT_1 | Q2RQV3 | 5.18 | 154911.5 |
| RPOB_SYNE7_1 | Q31N17 | 5.21 | 123276.6 |
| RPOB_STAES_1 | Q8CQ84 | 4.91 | 133039.1 |
| RPOB_ANADE_1 | Q2II86 | 5.51 | 159067.8 |
| RPOB_ANAVT_1 | Q3M5D0 | 5.36 | 124942.8 |
| RPOB_ERWT9_1 | B2VG96 | 5.15 | 150532.1 |
| RPOB_ESCF3_1 | B7LUL5 | 5.14 | 150630.4 |
| RPOB_HYPNA_1 | Q0BYA7 | 5.14 | 153360.3 |
| RPOB_PELPD_1 | A1ALT4 | 5.52 | 167643.8 |
| RPOB_CITK8_1 | A8AKT9 | 5.14 | 150557 |
| RPOB_FERNB_1 | A7HNY2 | 6.3 | 133663.6 |
| RPOB_PEPD6_1 | Q18CF1 | 4.76 | 139322.7 |
| RPOB_DESAD_1 | C6C179 | 5.24 | 153014.7 |
| RPOB_AMYMS_1 | Q9L637 | 4.98 | 129354.1 |
| RPOB_AZOC5_1 | A8HTY8 | 5.3 | 153144.7 |
| RPOB_AZOVD_1 | C1DKK5 | 5.35 | 150932.3 |
| RPOB_THIDA_1 | Q3SLQ6 | 5.87 | 152058.4 |
| RPOB_WIGBR_1 | Q8D233 | 7.29 | 151550.3 |
| RPOB_SYNWW_1 | Q0AUH2 | 5.23 | 124192.5 |
| RPOB_XYLFA_1 | Q9PA86 | 5.63 | 154782.1 |
| RPOB_YERP3_1 | A7FNI3 | 5.16 | 150389.1 |
| RPOB_STRMU_1 | Q8DS46 | 5.04 | 132716.9 |
| RPOB_STRE4_1 | C0M7C8 | 4.97 | 132952 |
| RPOB_STRMK_1 | B2FQ38 | 5.36 | 154731.7 |
| RPOB_ARCB4_1 | A8EVZ4 | 5.8 | 155153.4 |
| RPOB_ARTS2_1 | A0JZ93 | 4.9 | 128726.4 |
| RPOB_TREDE_1 | Q73JJ7 | 5.8 | 131848.6 |
| RPOB_HISS2_1 | B0URZ6 | 5.27 | 149963.5 |
| RPOB_BACHD_1 | Q9Z9M2 | 5.04 | 132638.4 |
| RPOB_PELCD_1 | Q3A6Q4 | 5.3 | 152263.6 |
| RPOB_CHRVO_1 | Q7NQE6 | 5.47 | 155556.1 |
| RPOB_IDILO_1 | Q5QWA5 | 5.12 | 150266.6 |
| RPOB_PELTS_1 | A5D5I2 | 4.94 | 137444 |
| RPOB_AGRFC_1 | Q8UE08 | 4.99 | 153644.8 |
| RPOB_DESAH_1 | C0Q9X9 | 5.99 | 156023.6 |
| RPOB_FRACC_1 | Q2JFI5 | 5.09 | 126456.2 |
| RPOB_KORVE_1 | Q1IHH4 | 6.06 | 167265.2 |
| RPOB_BARHE_1 | Q9KJG4 | 5.08 | 154853.3 |
| RPOB_LACAC_1 | Q5FM97 | 5.31 | 135822.3 |
| RPOB_BART1_1 | A9ISG1 | 5.1 | 155048.8 |
| RPOB_BURVG_1 | A4JAN2 | 5.46 | 153373.8 |
| RPOB_PSYA2_1 | Q4FQH3 | 5.11 | 152757.5 |
| RPOB_CALBD_1 | B9MQG5 | 4.98 | 138420.4 |
| RPOB_LACH4_1 | A8YXJ8 | 5.5 | 135705.4 |
| RPOB_LACJO_1 | Q74L95 | 5.36 | 135813.7 |
| RPOB_POLAQ_1 | A4SUV4 | 5.54 | 152502.4 |
| RPOB_POLNA_1 | A1VTF8 | 5.81 | 152543 |
| RPOB_METPB_1 | B1ZGS0 | 5.2 | 152750 |
| RPOB_LACP3_1 | Q034X0 | 5.21 | 133701.2 |
| RPOB_BLOPB_1 | Q492B9 | 6.26 | 151222.3 |
| RPOB_NOSS1_1 | P22703 | 5.43 | 126524.6 |
| RPOB_BORAP_1 | Q0SNB8 | 5.73 | 129868.6 |
| RPOB_HAEDU_1 | Q7VKL7 | 5.2 | 149853.4 |
| RPOB_PAEAT_1 | A1R8V4 | 4.88 | 128955.7 |
| RPOB_PARD8_1 | A6LE81 | 5.45 | 142995.1 |
| RPOB_PARL1_1 | A7HWQ4 | 5.17 | 151815.9 |
| RPOB_EHRCJ_1 | Q3YST5 | 5.25 | 155494.8 |
| RPOB_HAHCH_1 | Q2S905 | 5.21 | 151520.3 |
| RPOB_HALHL_1 | A1WVC9 | 5.05 | 153691.5 |
| RPOB_PARXL_1 | Q13TG2 | 5.48 | 153056.4 |
| RPOB_RHOFT_1 | Q21SF7 | 5.68 | 152755.1 |
| RPOB_SPHWW_1 | A5VBZ8 | 5.07 | 160893.5 |
| RPOB_RICPU_1 | C4K1N9 | 6 | 154301.8 |
| RPOB_AROAE_1 | Q5P339 | 5.39 | 152838.7 |
| RPOB_AZOSB_1 | A1KB34 | 5.41 | 152990.2 |
| RPOB_THEAB_1 | B7ICR3 | 5.8 | 132195.6 |
| RPOB_AQUAE_1 | O67762 | 6.35 | 167387.6 |
| RPOB_THISH_1 | B8GV65 | 5.45 | 151563.9 |
| RPOB_STAHJ_1 | Q4L3K3 | 4.89 | 133268.5 |
| RPOB_THEP1_1 | A5IJW3 | 5.22 | 143205.5 |
| RPOB_THEPX_1 | B0K5G8 | 4.78 | 138832.6 |
| RNA Polymerase subunit beta' | Accession number | pI | Molecular Weight |
| RPOC_ECOLI_1 | P0A8T7 | 6.67 | 155160.3 |
| RPOC_MYCTU_1 | P9WGY7 | 5.86 | 146769.3 |
| RPOC_BACSU_1 | P37871 | 8.79 | 134252.1 |
| RPOC_THET8_1 | Q8RQE8 | 6.05 | 170756.5 |
| RPOC_MYCS2_1 | A0QS66 | 6.05 | 146513.3 |
| RPOC_STAA8_1 | Q2G0N5 | 6.53 | 135408.5 |
| RPOC_MYCGE_1 | P47582 | 9.34 | 145705.5 |
| RPOC_SALTY_1 | P0A2R4 | 6.43 | 155234.2 |
| RPOC2_PROMA_1 | Q7VA30 | 5.16 | 149736.6 |
| RPOC_RHOS1_1 | A3PGJ0 | 5.84 | 157344.5 |
| RPOBC_HELPH_1 | Q1CS68 | 6.69 | 323862.5 |
| RPOBC_HELAH_1 | Q17VN6 | 6.6 | 323895.7 |
| RPOBC_WOLPM_1 | Q73IW9 | 6.02 | 317782.4 |
| RPOBC_WOLSU_1 | Q7MA56 | 6.38 | 321723.6 |
| RPOBC_HELHP_1 | Q7VJ82 | 7.05 | 324320.5 |
| RPOBC_WOLTR_1 | Q5GRY9 | 6.21 | 318102.2 |
| RPOC_ALIF1_1 | Q5E239 | 5.66 | 155111.1 |
| RPOC_ALKOO_1 | A8MLD3 | 7.33 | 132049.4 |
| RPOC_FRATT_1 | Q5NID1 | 5.97 | 157417.1 |
| RPOC_BORHD_1 | B2S091 | 7.29 | 154598.8 |
| RPOC_DEIGD_1 | Q1J0P7 | 5.5 | 170602.5 |
| RPOC2_SYNPX_1 | Q7U8K2 | 5.08 | 148443.4 |
| RPOC_LEPCP_1 | B1Y7H2 | 7.31 | 155479.4 |
| RPOC_GEOBB_1 | B5EFP4 | 6.29 | 153878.1 |
| RPOC_DESVV_1 | A1VAJ5 | 6.67 | 154813.2 |
| RPOC_DICNV_1 | A5EX69 | 6.47 | 155425.5 |
| RPOC2_TRIEI_1 | Q110H3 | 4.77 | 155547.8 |
| RPOC_MYXXD_1 | Q1D7U2 | 7.54 | 156313.8 |
| RPOC_PSECP_1 | B8HD15 | 5.79 | 143389.6 |
| RPOC_SHIFL_1 | P0A8T9 | 6.67 | 155160.3 |
| RPOC_STRSY_1 | A4VSK3 | 6.76 | 135579.2 |
| RPOC_HAES1_1 | Q0I5B8 | 5.85 | 157931.4 |
| RPOC_NEIMB_1 | Q9K1J1 | 6.86 | 153762 |
| RPOC_PSEMY_1 | A4XZ96 | 6.7 | 154682.1 |
| RPOC_HERA2_1 | A9B6J1 | 5.69 | 172905.1 |
| RPOC_BACCN_1 | A7GK13 | 8.86 | 134741.3 |
| RPOC_XANP2_1 | A7IKQ1 | 8.48 | 154869.4 |
| RPOC_BRUC2_1 | A9M5Q8 | 6.42 | 155631.5 |
| RPOC_LISW6_1 | A0AF64 | 8.42 | 134845.2 |
| RPOC_SERP5_1 | A8G8E8 | 6.76 | 155223.7 |
| RPOC_BURCH_1 | A0K3L8 | 6.56 | 156129.5 |
| RPOC_ACIET_1 | B9MH46 | 6.49 | 154551 |
| RPOC_EHRCR_1 | Q2GFP4 | 6.7 | 157690.1 |
| RPOC_YERPS_1 | Q66FQ1 | 6.73 | 154904.4 |
| RPOC_NOCSJ_1 | A1SEK2 | 6.38 | 143494.5 |
| RPOC_BURM7_1 | A3MRU6 | 6.51 | 155946.3 |
| RPOC_CORA7_1 | C3PKN2 | 5.51 | 148010.8 |
| RPOC_STAHJ_1 | Q4L3K4 | 6.14 | 135006.8 |
| RPOC_RHIEC_1 | Q2K9M3 | 6.41 | 155289.6 |
| RPOC_STRA5_1 | Q8E238 | 6.17 | 135471 |
| RPOC_THEM4_1 | A6LKB7 | 6.19 | 185604.7 |
| RPOC_CALS4_1 | Q8R7U7 | 9 | 133279.6 |
| RPOC_STRMU_1 | Q8DS47 | 6.13 | 134977 |
| RPOC_ORITI_1 | B3CV54 | 8.72 | 154836.6 |
| RPOC_THESQ_1 | B1L934 | 6.15 | 190605 |
| RPOC_CAMLR_1 | B9KFG8 | 8.43 | 168830.8 |
| RPOC_BIFAA_1 | A1A316 | 5.33 | 148152.5 |
| RPOC_TREPA_1 | O83270 | 7.91 | 159790.1 |
| RPOC_VIBCH_1 | Q9KV29 | 6.48 | 155022.7 |
| RPOC_XANC5_1 | Q3BWZ0 | 7.82 | 155108 |
| RPOC_ALKMQ_1 | A6TWI9 | 8.42 | 130477.6 |
| RPOC_CHESB_1 | Q11HB4 | 6.65 | 155031.7 |
| RPOC_BORBU_1 | O51349 | 8.18 | 154658.2 |
| RPOC_BORDL_1 | B5RLU8 | 8.19 | 154569.9 |
| RPOC_RHOPA_1 | Q6N4S0 | 8.12 | 156056.2 |
| RPOC_CHLFF_1 | Q255E5 | 7.87 | 155021.2 |
| RPOC_PELTS_1 | A5D5I3 | 8.61 | 130826.8 |
| RPOC_ANASK_1 | B4UDT1 | 7.28 | 154659 |
| RPOC_MYCPA_1 | Q73SE3 | 5.87 | 147015.7 |
| RPOC_GEOKA_1 | Q5L404 | 9.13 | 134880.7 |
| RPOC_CHLPD_1 | A1BD24 | 7.78 | 166390.5 |
| RPOC_GEOSM_1 | C6E4R3 | 6.29 | 153915.2 |
| RPOC_RICCK_1 | A8EXK9 | 7.71 | 154039.6 |
| RPOC_PORG3_1 | B2RL44 | 6.84 | 160243.6 |
| RPOC_CHLTB_1 | B0BBU5 | 7.16 | 154839.7 |
| RPOC_RICM5_1 | A8F0P8 | 8.03 | 153461 |
| RPOC_RICRO_1 | B0BWB0 | 8.25 | 153274.8 |
| RPOC_PSEHT_1 | Q3ILP8 | 6.05 | 154568.2 |
| RPOC_XANOM_1 | Q2NZX9 | 7.57 | 155232.2 |
| RPOC_SORC5_1 | A9GRB4 | 7.35 | 158828.1 |
| RPOC_IDILO_1 | Q5QWA4 | 5.86 | 156412.3 |
| RPOC_CLOBM_1 | B1KSN2 | 8.22 | 131579.2 |
| RPOC_CLOK5_1 | A5N4P0 | 8.85 | 131468.7 |
| RPOC_NITWN_1 | Q3SSX9 | 8.22 | 155812.1 |
| RPOC_AERHH_1 | A0KQA4 | 6.37 | 158230.7 |
| RPOC_BACTN_1 | Q8A470 | 6.69 | 158439.3 |
| RPOC_MYCA5_1 | B3PM77 | 7.05 | 165333 |
| RPOC_LACDA_1 | Q1GBM4 | 6.06 | 136287.9 |
| RPOC_MYCBT_1 | C1AL01 | 5.82 | 146682.2 |
| RPOC_FRACC_1 | Q2JFI4 | 7.55 | 143900.5 |
| RPOC_TOLAT_1 | C4LBV1 | 6.96 | 157857.6 |
| RPOC_AKKM8_1 | B2UQY1 | 6.31 | 155528.1 |
| RPOC_BORBR_1 | Q7WRD8 | 6.4 | 156240.5 |
| RPOC_CHLAA_1 | A9WH11 | 5.61 | 169343.9 |
| RPOC_CYTH3_1 | Q11QA6 | 7.57 | 160129.3 |
| RPOC_CHLAD_1 | B8G4U8 | 5.68 | 168948.5 |
| RPOC_LEGPH_1 | Q5ZYP9 | 6.39 | 155653.7 |
| RPOC_DEIRA_1 | Q9RVW0 | 5.21 | 171361 |
| RPOC_LEPBL_1 | Q054E1 | 6.72 | 157533.9 |
| RPOC_PETMO_1 | A9BF32 | 5.69 | 187026.7 |
| RPOC_PHOPR_1 | Q6LLW3 | 6 | 155155.1 |
| RPOC_MYCPU_1 | Q98Q24 | 8.47 | 160697.3 |
| RPOC_BORT9_1 | A1QZH6 | 6.79 | 154542.6 |
| RPOC_ROSCS_1 | A7NJM0 | 5.6 | 169133.5 |
| RPOC_AZOSB_1 | A1KB33 | 6.1 | 155014.1 |
| RPOC_RUEST_1 | Q1GK48 | 5.59 | 156712.6 |
| RPOC_PSEP1_1 | A5VXP1 | 6.8 | 154770.2 |
| RPOC_HISS2_1 | B0URZ7 | 5.85 | 157917.3 |
| RPOC_SULMW_1 | A8Z5T2 | 9.04 | 167038.8 |
| RPOC_MARHV_1 | A1TYJ1 | 5.72 | 155265.9 |
| RPOC_YERPG_1 | A9R0H9 | 6.73 | 154904.4 |
| RPOC_METI4_1 | B3E164 | 8.75 | 155875.9 |
| RPOC_BURM1_1 | A9ADI6 | 6.52 | 156177.6 |
| RPOC_SHEDO_1 | Q12SW5 | 6.08 | 155541.7 |
| RPOC_LACBA_1 | Q03PV0 | 5.98 | 135677.4 |
| RPOC_THENN_1 | B9KBJ4 | 6.48 | 191027.4 |
| RPOC_COXBU_1 | Q83ET0 | 7.62 | 157103.5 |
| RPOC_CALS8_1 | A4XI31 | 8.6 | 130684.4 |
| RPOC_LACLA_1 | Q9CEN7 | 6.22 | 134731 |
| RPOC_STRP6_1 | Q5XE96 | 6.6 | 135340.7 |
| RPOC_THEYD_1 | B5YFV7 | 8.37 | 153474.9 |
| RPOC_STRPN_1 | Q97NQ8 | 6.6 | 136988.6 |
| RPOC_UREP2_1 | B1AIH6 | 8.82 | 147970.1 |
| RPOC_SHESM_1 | Q0HNU3 | 6.03 | 155469.6 |
| RPOC_ALKEH_1 | Q0ABI1 | 5.74 | 157013.4 |
| RPOC_CELJU_1 | B3PK31 | 6.92 | 155405.9 |
| RPOC_LAWIP_1 | Q1MPW9 | 6.67 | 155136.5 |
| RPOC_PELPB_1 | B4SG10 | 7.56 | 167165.6 |
| RPOC_ANAMM_1 | Q5PBG3 | 6.63 | 156717.8 |
| RPOC_LEIXX_1 | Q6ACX6 | 6.13 | 142373.5 |
| RPOC_DESAG_1 | Q30X04 | 6.54 | 154096.3 |
| RPOC_AQUAE_1 | O67763 | 6.32 | 178501 |
| RPOC_MYCPE_1 | Q8EWX0 | 8.83 | 145305.8 |
| RPOC_MYCSK_1 | A1UBJ4 | 6.07 | 146870.7 |
| RPOC_RICCN_1 | Q9RH40 | 8.03 | 153244.6 |
| RPOC_SHIBS_1 | Q31U09 | 6.67 | 155160.3 |
| RPOC_DINSH_1 | A8LM41 | 5.44 | 157123.2 |
| RPOC_ROSS1_1 | A5USR6 | 5.48 | 169331 |
| RPOC_PSEF5_1 | Q4K527 | 6.75 | 154542.9 |
| RPOC_SODGM_1 | Q2NWR5 | 6.39 | 155490.6 |
| RPOC_BRUA1_1 | B2S686 | 6.48 | 155640.6 |
| RPOC_STRT2_1 | Q5M2F6 | 6.13 | 135329.8 |
| RPOC_SALAI_1 | A8M538 | 7.14 | 144165 |
| RPOC_LISMF_1 | Q724E9 | 8.42 | 134755.2 |
| RPOC_BACCZ_1 | Q63H97 | 8.74 | 134393.8 |
| RPOC_BRUO2_1 | A5VR14 | 6.48 | 155600.5 |
| RPOC_MAGSA_1 | Q2W2I2 | 6.58 | 165697.2 |
| RPOC_NITHX_1 | Q1QN43 | 8.77 | 155671.2 |
| RPOC_BUCAI_1 | P57145 | 9.06 | 156944.3 |
| RPOC_EHRRG_1 | Q5FFD8 | 6.6 | 157170.6 |
| RPOC_ACTP7_1 | B3GYV5 | 6.09 | 157588 |
| RPOC_THEAQ_1 | Q9KWU6 | 6.12 | 170943.6 |
| RPOC_ENTFA_1 | Q82Z41 | 6.48 | 136815.5 |
| RPOC_COREF_1 | Q8FS96 | 5.59 | 147460.4 |
| RPOC_CORGB_1 | A4QBG3 | 5.51 | 147309.1 |
| RPOC_SHEPA_1 | A8GYX0 | 6.04 | 155186.2 |
| RPOC_AMOA5_1 | B3ETY9 | 8.23 | 160564.7 |
| RPOC_LEPBA_1 | B0SAG0 | 5.76 | 160647.1 |
| RPOC_BORPD_1 | A9IJ22 | 6.59 | 155939.1 |
| RPOC_GEODF_1 | B9M6V1 | 6.95 | 153201.8 |
| RPOC_AQUPY_1 | Q9X6Y2 | 6.39 | 178431.9 |
| RPOC_GEOMG_1 | Q39Y12 | 6.16 | 155028.6 |
| RPOC2_SYNY3_1 | P73334 | 4.72 | 144776.9 |
| RPOC_ALCBS_1 | Q0VSM1 | 5.94 | 155524.6 |
| RPOC_ANADE_1 | Q2II85 | 7.08 | 154551.8 |
| RPOC_PELPD_1 | A1ALT5 | 6.4 | 156570.2 |
| RPOC_BORPA_1 | Q7W2G8 | 6.4 | 156240.5 |
| RPOC_MYCMO_1 | Q6KI08 | 8.91 | 161754.4 |
| RPOC_BORPE_1 | Q7W0R8 | 6.4 | 156240.5 |
| RPOC_CHLPM_1 | A4SGK5 | 7.18 | 165024.1 |
| RPOC_GLUDA_1 | A9H3S0 | 8.28 | 155467.3 |
| RPOC_BRASB_1 | A5ELN6 | 8.54 | 155774.1 |
| RPOC_GRABC_1 | Q0BUQ5 | 7.78 | 154354.6 |
| RPOC_PSEA7_1 | A6UZI2 | 6.65 | 154369.4 |
| RPOC_AZOC5_1 | A8HTZ1 | 7.8 | 154729 |
| RPOC_HAEDU_1 | Q7VKL8 | 6.09 | 157316.9 |
| RPOC_CITK8_1 | A8AKT8 | 6.48 | 155211.2 |
| RPOC_ROSDO_1 | Q160X8 | 5.67 | 156724.9 |
| RPOC_CLAMS_1 | B0RB26 | 6.15 | 143323.6 |
| RPOC_RUEPO_1 | Q5LMQ6 | 5.53 | 156805.7 |
| RPOC_HALHL_1 | A1WVC8 | 5.54 | 157763.7 |
| RPOC_SPHAL_1 | Q1GT24 | 5.91 | 158095 |
| RPOC_SPHWW_1 | A5VBZ7 | 5.71 | 157224.2 |
| RPOC_HYDS0_1 | B4U738 | 8.28 | 176239.8 |
| RPOC_SALPB_1 | A9N0J5 | 6.43 | 155186.2 |
| RPOC_XYLFM_1 | B0U5X6 | 7.53 | 156391.1 |
| RPOC_SALTI_1 | P0A2R5 | 6.43 | 155234.2 |
| RPOC_SYNFM_1 | A0LII5 | 7.55 | 150843.3 |
| RPOC_METRJ_1 | B1LY42 | 8.09 | 154555.3 |
| RPOC_NOVAD_1 | Q2GCD8 | 5.77 | 157647.9 |
| RPOC_SHEFN_1 | Q089R0 | 6.12 | 155570.6 |
| RPOC_CORDI_1 | Q6NJF6 | 5.55 | 147984.1 |
| RPOC_MOOTA_1 | Q2RFP0 | 8.39 | 129845.7 |
| RPOC_BACVZ_1 | A7Z0N0 | 8.69 | 134167.8 |
| RPOC_RHOE4_1 | C0ZVQ7 | 6.2 | 146305.2 |
| RPOC_CAMJE_1 | Q9PI30 | 8.57 | 168822.9 |
| RPOC_PARP8_1 | B2JIH3 | 6.39 | 156478.6 |
| RPOC_BLOFL_1 | Q7VRP8 | 8.18 | 158450 |
| RPOC_BORBP_1 | Q661N0 | 8.42 | 154422 |
| RPOC_TROWT_1 | Q820D6 | 7.26 | 142577.9 |
| RPOC_SHEPC_1 | A4YBY9 | 6.06 | 155298.5 |
| RPOC_PELCD_1 | Q3A6Q3 | 5.85 | 155016.6 |
| RPOC_DESOH_1 | A8ZV52 | 8.44 | 160380.9 |
| RPOC_PEPD6_1 | Q18CF3 | 8.1 | 129729.5 |
| RPOC_MYCPN_1 | P75271 | 9.29 | 144895.5 |
| RPOC_MYCS5_1 | Q4A5S8 | 6.2 | 169481.3 |
| RPOC_RICBR_1 | Q1RHD1 | 8.25 | 152026.4 |
| RPOC_SOLUE_1 | Q01VB2 | 6.09 | 156896.1 |
| RPOC_HELMI_1 | B0TC49 | 8.41 | 132243.3 |
| RPOC_BRUSU_1 | Q8G070 | 6.42 | 155601.5 |
| RPOC_ACIAC_1 | A1TVS9 | 6.59 | 154758 |
| RPOC_SALTO_1 | A4XBQ5 | 7.14 | 144029.9 |
| RPOC_ACIBY_1 | B0VDH9 | 6.9 | 155050.8 |
| RPOC_SHEB2_1 | B8EBL1 | 6.15 | 155315.5 |
| RPOC_ERWT9_1 | B2VG97 | 6.81 | 155466.9 |
| RPOC_BURP1_1 | Q3JMQ4 | 6.43 | 155992.3 |
| RPOC_STRCO_1 | Q8CJT1 | 6.44 | 144604.4 |
| RPOC_FLAPJ_1 | A6GYT9 | 7.94 | 160605.9 |
| RPOC2_ANAVT_1 | Q3M5C8 | 4.8 | 147515.5 |
| RPOC_VIBCB_1 | A7MXF0 | 5.95 | 155025.4 |
| RPOC_CUPNH_1 | Q0K607 | 6.51 | 156035.7 |
| RPOC_CUTAK_1 | Q6A6K7 | 6.44 | 143444.1 |
| RPOC2_SYNE7_1 | Q31N15 | 4.88 | 143838.3 |
| RPOC_BORRA_1 | B5RRJ6 | 8.19 | 154567.9 |
| RPOC_DESRM_1 | A4J104 | 7.28 | 133190.5 |
| RPOC_POLNS_1 | B1XSP4 | 6.87 | 156449.6 |
| RPOC_GEOUR_1 | A5GAY2 | 7.77 | 153055.7 |
| RPOC_MYCUA_1 | A0PM25 | 5.85 | 146815.4 |
| RPOC_CHRVO_1 | Q7NQE7 | 7.3 | 155292.9 |
| RPOC_ACIAD_1 | Q6FF89 | 7 | 155194.9 |
| RPOC_PSEU2_1 | Q4ZMN8 | 6.78 | 154780.1 |
| RPOC_MESFL_1 | Q6F0L8 | 8.37 | 139622.4 |
| RPOC_JANSC_1 | Q28UX6 | 5.33 | 155204.9 |
| RPOC_KORVE_1 | Q1IHH5 | 6.3 | 156561.4 |
| RPOC_EXIS2_1 | B1YGU3 | 8.64 | 134063.2 |
| RPOC_MYCA9_1 | B1MH61 | 6.29 | 146542.3 |
| RPOC_THEMA_1 | P36252 | 6.15 | 190520.9 |
| RPOC_STRE4_1 | C0M7R5 | 6.71 | 135332 |
| RPOC_CROS8_1 | A7MQQ8 | 6.49 | 155003.1 |
| RPOC_BARBK_1 | A1USC9 | 6.81 | 156008.9 |
| RPOC_LACP3_1 | Q034X1 | 5.98 | 136463.1 |
| RPOC_LACPL_1 | Q88XZ2 | 6.63 | 135318.4 |
| RPOC_PARUW_1 | Q6MDM0 | 7.31 | 155296.8 |
| RPOC_UNCTG_1 | B1GZ76 | 8.97 | 179214.4 |
| RPOC_CUPMC_1 | Q1LI21 | 6.61 | 155726.4 |
| RPOC_CUPTR_1 | B3R7T5 | 6.51 | 155961.7 |
| RPOC_MYCMM_1 | B2HSJ4 | 5.94 | 146762.4 |
| RPOC_DESAP_1 | B1I1M9 | 8.53 | 129281.1 |
| RPOC2_NOSP7_1 | B2J1A0 | 4.76 | 146860.9 |
| RPOC_CHLL2_1 | B3EER3 | 8.07 | 166732.2 |
| RPOC_PHOLL_1 | Q7N9A3 | 6.42 | 154763.7 |
| RPOC_LEUCK_1 | B1MVW7 | 5.72 | 135411.5 |
| RPOC_CHLT3_1 | B3QYL5 | 8.1 | 167904.5 |
| RPOC_CHLTE_1 | Q8KG14 | 6.6 | 166281.6 |
| RPOC_PSEA6_1 | Q15YB0 | 5.66 | 154817.3 |
| RPOC_AYWBP_1 | Q2NJ16 | 8.91 | 153982.1 |
| RPOC_HAEI8_1 | Q4QN34 | 5.73 | 157216.5 |
| RPOC_SINMW_1 | A6U852 | 6.35 | 155334.8 |
| RPOC_RUTMC_1 | A1AX74 | 6.47 | 155261.1 |
| RPOC_STRU0_1 | B9DSZ6 | 6.2 | 135349.7 |
| RPOC_BACHK_1 | Q6HPR5 | 8.74 | 134420.8 |
| RPOC_METEP_1 | A9W8N9 | 7.55 | 154860.2 |
| RPOC_CLOPE_1 | P0C2E8 | 6.39 | 131607.9 |
| RPOC_PSYA2_1 | Q4FQH4 | 5.91 | 156049.5 |
| RPOC_BURCM_1 | Q0BJ53 | 6.45 | 156248.6 |
| RPOC_ENT38_1 | A4W5A8 | 6.44 | 155094.2 |
| RPOC_AERS4_1 | A4SHV0 | 6.22 | 158198.7 |
| RPOC_FINM2_1 | B0S2E5 | 5.85 | 135073.9 |
| RPOC_OCHA4_1 | A6X0B0 | 6.56 | 155653.5 |
| RPOC_OENOB_1 | Q04E86 | 6.17 | 136990 |
| RPOC_STRGG_1 | B1W441 | 7.27 | 144471.5 |
| RPOC_CAMFF_1 | A0RQI4 | 8.25 | 168010.8 |
| RPOC_PAEAT_1 | A1R8V3 | 5.78 | 143505.6 |
| RPOC_PARDP_1 | A1B017 | 5.57 | 155462.1 |
| RPOC_BEII9_1 | B2IK56 | 6.85 | 154440.5 |
| RPOC2_ACAM1_1 | B0C385 | 4.83 | 145388.1 |
| RPOC_SHEWM_1 | B1KMY9 | 5.83 | 155289.1 |
| RPOC2_MICAN_1 | B0JGK8 | 4.66 | 146829.9 |
| RPOC_MYCMS_1 | Q6MRX5 | 8.37 | 141359 |
| RPOC_LEPIN_1 | Q8F0S3 | 6.55 | 157624 |
| RPOC_BRADU_1 | Q89J75 | 8.64 | 155426.7 |
| RPOC_LISIN_1 | P77879 | 8.51 | 134755.2 |
| RPOC_BRUMB_1 | C0RJK9 | 6.42 | 155557.4 |
| RPOC_MANSM_1 | Q65W40 | 5.76 | 157562 |
| RPOC_BACHD_1 | Q9Z9M1 | 8.39 | 134927.5 |
| RPOC_SYNAS_1 | Q2LQ86 | 8.21 | 153996.6 |
| RPOC_ACICJ_1 | A5FZX0 | 7.96 | 153790 |
| RPOC_PSEU5_1 | A4VHM4 | 6.4 | 154986.4 |
| RPOC_BACSK_1 | Q5WLR9 | 8.69 | 134496.8 |
| RPOC_COLP3_1 | Q47UW0 | 6.02 | 155319.8 |
| RPOC_SHEHH_1 | B0TM18 | 6.04 | 155254.3 |
| RPOC_FLAJ1_1 | A5FIJ4 | 7.52 | 160128.3 |
| RPOC_LACF3_1 | B2GDX6 | 6.52 | 135576.7 |
| RPOC_FRASN_1 | A8LC63 | 7.57 | 144118.7 |
| RPOC_MYCGI_1 | A4T1P3 | 6.1 | 147031.9 |
| RPOC_MYCH7_1 | Q4A7B0 | 9.24 | 159395.7 |
| RPOC_MYCLB_1 | B8ZSC6 | 6.21 | 146895.7 |
| RPOC_LACP7_1 | A9KJL3 | 5.7 | 140846.4 |
| RPOC_PEDPA_1 | Q03EB0 | 6.03 | 135034.2 |
| RPOC_CHLAB_1 | Q5L5I4 | 7.35 | 154963.1 |
| RPOC_DEHMC_1 | Q3ZX00 | 7.05 | 144316.7 |
| RPOC2_CYAA5_1 | B1WZT6 | 4.77 | 143093.4 |
| RPOC_DESPS_1 | Q6AP77 | 8.35 | 149797.2 |
| RPOC_RICAH_1 | A8GMA8 | 8.33 | 153256.7 |
| RPOC_HAHCH_1 | Q2S906 | 6.62 | 154517.8 |
| RPOC_SACD2_1 | Q21M92 | 6.35 | 155649.8 |
| RPOC_SULNB_1 | A6Q6I3 | 5.98 | 166492.4 |
| RPOC_KINRD_1 | A6W5T1 | 6.21 | 144099.4 |
| RPOC_METPB_1 | B1ZGS1 | 7.78 | 154748.2 |
| RPOC_BURTA_1 | Q2SU20 | 6.52 | 156126.4 |
| RPOC_THEPX_1 | B0K5G9 | 8.86 | 133185.2 |
| RPOC_BIFLD_1 | B3DTE1 | 5.45 | 149265 |
| RPOC_DECAR_1 | Q47JA9 | 6.37 | 155039.3 |
| RPOC_CHLCH_1 | Q3ATP4 | 6.93 | 167739.4 |
| RPOC_FUSNN_1 | Q8RHI7 | 8.89 | 148103.2 |
| RPOC_DELAS_1 | A9BR99 | 6.57 | 154709 |
| RPOC_CHLL7_1 | Q3B1H8 | 6.77 | 166870.1 |
| RPOC_RHORT_1 | Q2RQV4 | 6.83 | 155038 |
| RPOC_ARCB4_1 | A8EVZ3 | 6.73 | 168145.1 |
| RPOC_GLUOX_1 | Q5FTX8 | 6.78 | 159526.6 |
| RPOC_RICPR_1 | Q9ZE20 | 8.04 | 153381.9 |
| RPOC_GRAFK_1 | A0M3Y8 | 7.5 | 160104.1 |
| RPOC_RICTY_1 | Q68XM7 | 7.71 | 153402.8 |
| RPOC_NEIG2_1 | B4RQW3 | 6.86 | 153796.1 |
| RPOC_BACAN_1 | P77819 | 8.74 | 134448.9 |
| RPOC_NITSB_1 | A6Q1M4 | 8.03 | 168273.5 |
| RPOC_CLONN_1 | A0PXT9 | 7.26 | 132511.7 |
| RPOC_NOCFA_1 | Q5YPE1 | 6.02 | 146682.3 |
| RPOC_SYNWW_1 | Q0AUH3 | 5.85 | 132179.6 |
| RPOC_ELUMP_1 | B2KEN0 | 9 | 152804.8 |
| RPOC_METS4_1 | B0UHX5 | 8.13 | 154658.1 |
| RPOC_OCEIH_1 | Q8ETY7 | 6.12 | 134618.4 |
| RPOC_THEP1_1 | A5IJW2 | 6.17 | 190694 |
| RPOC_RHOCS_1 | B6IRP7 | 6.68 | 159414.1 |
| RPOC_MYCGA_1 | P47716 | 9.07 | 145058.9 |
| RPOC_BARHE_1 | Q6G3X4 | 6.72 | 155586.4 |
| RPOC_PARL1_1 | A7HWQ5 | 5.98 | 154203.4 |
| RPOC_BDEBA_1 | Q6MJ10 | 8.31 | 151749.7 |
| RPOC_PARPJ_1 | B2T758 | 6.39 | 156378.6 |
| RPOC_BORA1_1 | Q2L2L3 | 6.47 | 156470.9 |
| RPOC_POLSJ_1 | Q123G4 | 7.08 | 154857.5 |
| RPOC_LEUMM_1 | P94892 | 5.61 | 135788.7 |
| RPOC2_GLOVI_1 | Q7NDF7 | 5 | 137588.1 |
| RPOC_NEOSM_1 | Q2GD91 | 8.65 | 152089.6 |
| RPOC_SALCH_1 | Q57H68 | 6.43 | 155234.2 |
| RPOC_BACFN_1 | Q5L898 | 6.83 | 158624.6 |
| RPOC_JANMA_1 | A6T3L2 | 7.51 | 156121.9 |
| RPOC_EHRCJ_1 | Q3YST4 | 7.01 | 157617.1 |
| RPOC_PSYCK_1 | Q1Q8Q0 | 5.98 | 155161.4 |
| RPOC_PSYWF_1 | A5WH34 | 5.83 | 155676.1 |
| RPOC_THEFY_1 | Q47LI6 | 8.02 | 145295.4 |
| RPOC_STAS1_1 | Q49V53 | 6.24 | 135249.2 |
| RPOC_MYCA1_1 | A0QL48 | 5.87 | 147027.7 |
| RPOC_CORJK_1 | Q4JT33 | 5.88 | 146329.4 |
| RPOC_STRAW_1 | Q82DQ4 | 6.76 | 145044 |
| RPOC_FRAP2_1 | B0TX11 | 5.99 | 157278.7 |
| RPOC_LACGA_1 | Q046D1 | 6 | 136371 |
| RPOC_RHOOB_1 | C1AYV8 | 6 | 146389 |
| RPOC_CARHZ_1 | Q3A9Q8 | 8.66 | 129252.7 |
| RPOC_LACS1_1 | Q1WVA4 | 6.24 | 136647.4 |
| RPOC_PECAS_1 | Q6DAM9 | 6.74 | 155419.9 |
| RPOC_UREU1_1 | B5ZAZ4 | 8.71 | 147789.9 |
| RPOC_SHEPW_1 | B8CNC6 | 5.87 | 155072 |
| RPOC_WIGBR_1 | Q8D232 | 9.25 | 157285 |
| RPOC_BACV8_1 | A6KYK2 | 6.7 | 158838.9 |
| RPOC_RHIL3_1 | Q1MIE8 | 6.55 | 155492.1 |
| RPOC_CORU7_1 | B1VES2 | 5.96 | 146471.6 |
| RPOC_SULDN_1 | Q30TP6 | 8.09 | 166265.5 |
| RPOC_SYMTH_1 | Q67JT4 | 7.14 | 132940.9 |
| RPOC_METPP_1 | A2SLG4 | 7.99 | 155395.5 |
| RPOC_PSYIN_1 | A1T064 | 6.26 | 155103.5 |
| RPOC_RALSO_1 | Q8XUZ9 | 6.69 | 155076.7 |
| RPOC_AGRFC_1 | Q8UE09 | 6.41 | 155516 |
| RPOC_LACCB_1 | B3WAM7 | 5.98 | 136451.1 |
| RPOC_RHOBA_1 | Q7URW4 | 6.39 | 159165.5 |
| RPOC_STRGC_1 | A8AZI2 | 6.7 | 135510.4 |
| RPOC_BEUC1_1 | C5C0K2 | 5.88 | 143368.5 |
| RPOC_PASMU_1 | Q9CK92 | 5.97 | 157186.8 |
| RPOC_TREDE_1 | Q73JJ8 | 7.11 | 160036.5 |
| RPOC_VEREI_1 | A1WK56 | 6.91 | 155017.6 |
| RPOC_DESHY_1 | Q250N9 | 8.41 | 130062.9 |
| RPOC2_NOSS1_1 | P22705 | 4.81 | 147582.6 |
| RPOC_CHLP8_1 | B3QQS1 | 6.99 | 166471.5 |
| RPOC_GEOLS_1 | B3E7S9 | 6.15 | 154169.5 |
| RPOC_AROAE_1 | Q5P338 | 6.61 | 155259.6 |
| RPOC_PROA2_1 | B4S498 | 6.43 | 166784.5 |
| RPOC_MYCVP_1 | A1T4J3 | 6.1 | 147143.9 |
| RPOC_PSEE4_1 | Q1IFX2 | 6.8 | 154788.2 |
| RPOC_SHISS_1 | Q3YUZ6 | 6.67 | 155160.3 |
| RPOC_AZOPC_1 | B6YPZ6 | 8.06 | 158028.3 |
| RPOC_PSELT_1 | A8F4G1 | 6.04 | 184460.6 |
| RPOC_CLOAB_1 | Q97EH0 | 8.1 | 131810 |
| RPOC_HYDCU_1 | Q31IY8 | 5.72 | 156163.1 |
| RPOC_MAGMM_1 | A0L5W7 | 7.13 | 157052 |
| RPOC_MARMS_1 | A6W398 | 6.04 | 154131.1 |
| RPOC_BACLD_1 | Q65PB4 | 8.4 | 134395 |
| RPOC_METNO_1 | B8IS79 | 7.98 | 154748.2 |
| RPOC_STAEQ_1 | Q5HRK9 | 5.89 | 135262.8 |
| RPOC_FRAAA_1 | Q0RRS9 | 7.81 | 144063.6 |
| RPOC_ONYPE_1 | Q6YQW2 | 8.62 | 153591.3 |
| RPOC_OPITP_1 | B1ZPB7 | 7.16 | 152789.1 |
| RPOC_THEP3_1 | B0KCJ3 | 8.84 | 133278.3 |
| RPOC_CAUVC_1 | Q9AAU1 | 6.4 | 154250.4 |
| RPOC_PARXL_1 | Q13TG3 | 6.39 | 156317.5 |
| RPOC_THIDA_1 | Q3SLQ5 | 6.67 | 153693.1 |
| RPOC_VARPS_1 | C5CKF4 | 6.82 | 154824.1 |
| RPOC_VIBVU_1 | Q8DD19 | 5.93 | 154662 |
| RPOC_CHLCV_1 | Q822J2 | 7.53 | 155059.2 |
| RPOC_PELUB_1 | Q4FLL3 | 8.81 | 154508.4 |
| RPOC_GEOTN_1 | A4IJI2 | 8.95 | 134807.4 |
| RPOC_PSE14_1 | Q48D30 | 6.78 | 154752.1 |
| RPOC_CHRSD_1 | Q1R0I1 | 5.6 | 155015.3 |
| RPOC_SHIDS_1 | Q32AG0 | 7.08 | 155188.4 |
| RPOC_NATTJ_1 | B2A4D2 | 6.72 | 136096.1 |
| RPOC_STRSV_1 | A3CKD4 | 6.6 | 135766.5 |
| RPOC_RUBXD_1 | Q1AU23 | 5.18 | 146243.2 |
| RPOC_SACEN_1 | A4FPP2 | 6.1 | 144955.3 |
| RPOC_ACHLI_1 | A9NEL8 | 7.37 | 155620.4 |
| RPOC_NITEU_1 | Q82T76 | 6.83 | 155851.4 |
| RPOC_MARMM_1 | Q0ANP4 | 5.36 | 154405.2 |
| RPOC_YERE8_1 | A1JII1 | 6.61 | 154952.4 |
| RPOC_SULSY_1 | B2V7L9 | 6.17 | 177600 |
| RPOC_METCA_1 | Q60A05 | 6.26 | 155233.4 |
| RPOC_ACIC1_1 | A0LRL4 | 6.77 | 145722.2 |
| RPOC_SHEAM_1 | A1S212 | 6.11 | 155271.1 |
| RPOC_ACISJ_1 | A1WCM8 | 6.59 | 154586.1 |
| RPOC_ZYMMO_1 | Q5NPK4 | 6.31 | 154075.4 |
| RPOC_SHELP_1 | A3Q976 | 5.89 | 155353.9 |
| RPOC_CORK4_1 | C4LL70 | 5.8 | 148388.2 |
| RPOC_RHIME_1 | Q92QH6 | 6.35 | 155347.8 |
| RPOC_BACWK_1 | A9VP70 | 8.87 | 134416.9 |
| RPOC_STRMK_1 | B2FQ39 | 6.69 | 155286.1 |
| RPOC_LACJO_1 | Q74L94 | 5.8 | 136440 |
| RPOC_CARRP_1 | Q05FH9 | 9.79 | 150183.4 |
| RPOC_BART1_1 | A9ISG4 | 6.87 | 155816.7 |
| RPOC_LACRJ_1 | B2G8Y5 | 7.06 | 135424.5 |
| RPOC_LACSS_1 | Q38UQ3 | 6.06 | 135927.9 |
| RPOC_BLOPB_1 | Q492C0 | 7.92 | 158292.5 |
| RPOC_BORAP_1 | Q0SNB9 | 8.36 | 154471.9 |
| RPOC_VIBPA_1 | Q87KQ5 | 5.81 | 154919 |
| RPOC_RHILO_1 | Q98N65 | 6.65 | 155100.7 |
| RPOC_BURVG_1 | A4JAN3 | 6.54 | 156188.6 |
| RPOC_MYCCT_1 | Q2ST47 | 8 | 141317.8 |
| RPOC_VESOH_1 | A5CW24 | 6.41 | 154881.6 |
| RPOC_MYCAP_1 | A5IZ52 | 6.47 | 167185.6 |
| RPOC_ANAPZ_1 | Q2GJ69 | 6.97 | 156243.4 |
| RPOC_POLNA_1 | A1VTF7 | 6.92 | 154619.2 |
| RPOC_ARTS2_1 | A0JZ92 | 5.94 | 143598.9 |
| RPOC_RICFE_1 | Q4UKD5 | 7.71 | 153404.9 |
| RPOC_HERAR_1 | A4G9U4 | 7.01 | 156152.9 |
| RPOC_CLOB8_1 | A6LPQ5 | 6.36 | 131775 |
| RPOC_SALRD_1 | Q2S1Q6 | 4.87 | 161773.3 |
| RPOC_NITMU_1 | Q2YB04 | 8.32 | 156214.1 |
| RPOC_BACP2_1 | A8F977 | 8.68 | 134173.8 |
| RPOC_KOCRD_1 | B2GIK0 | 5.64 | 143320 |
| RPOC_BURL3_1 | Q39KH4 | 6.47 | 156262.6 |
| RPOC_THEAB_1 | B7ICR2 | 5.72 | 184820.2 |
| RPOC_ACTSZ_1 | A6VKC4 | 5.73 | 157774.1 |
| RPOC_RENSM_1 | A9WSX9 | 6.16 | 143133.5 |
| RPOC_OLICO_1 | B6JES1 | 8.47 | 155616.4 |
| RPOC_RHOJR_1 | Q0SFB4 | 5.95 | 146371 |
| RPOC_PARD8_1 | A6LE80 | 6.64 | 159783.8 |
| RPOC_BIFA0_1 | B8DWJ5 | 5.23 | 149447.8 |
| RPOC_XANAC_1 | Q8PNS9 | 7.82 | 155136.1 |
| RPOC_SHEVI_1 | Q9KW13 | 6.53 | 155750.9 |
| RPOC_CHLMU_1 | Q9PK79 | 6.89 | 154898.7 |
| RPOC_POLAQ_1 | A4SUV5 | 7.04 | 156199.4 |
| RPOC_CHLPN_1 | Q9Z999 | 6.8 | 154901.1 |
| RPOC_SALAR_1 | A9MHE9 | 6.41 | 155198.2 |
| RPOC_LYSSC_1 | B1HMZ5 | 6.93 | 137293.8 |
| RPOC_HYPNA_1 | Q0BYA8 | 7.91 | 154543.9 |
| RPOC_NITEC_1 | Q0AF54 | 6.62 | 155337.7 |
| RPOC_NITOC_1 | Q3J8Q8 | 6.13 | 155582.9 |
| RPOC_METFK_1 | Q1H4P3 | 6.72 | 155515.9 |
| RPOC_KLEP7_1 | A6TGP1 | 6.46 | 155262.3 |
| RPOC_CLOTE_1 | Q890N5 | 7.51 | 126984.3 |
| RPOC_CLOTH_1 | A3DIZ5 | 8.8 | 130155.5 |
| RPOC_MICLC_1 | C5CC70 | 5.56 | 142417 |
| RPOC_LACAC_1 | Q5FM96 | 6.24 | 135745.3 |
| RPOC_ERYLH_1 | Q2N5Q5 | 5.72 | 159737.5 |
| RPOC_SHEON_1 | Q8EK73 | 5.97 | 155519.6 |
| RPOC_FERNB_1 | A7HNY1 | 6 | 186749.9 |
| RPOC_RHOFT_1 | Q21SF8 | 6.95 | 154592.9 |
| RPOC_LACH4_1 | A8YXJ9 | 6.2 | 135725.3 |
| RPOC_BARQU_1 | Q6FZM0 | 6.66 | 155572.4 |
| RPOC_CAUSK_1 | B0SUP8 | 6.51 | 154040.1 |
| RPOC_BAUCH_1 | Q1LSX6 | 6.7 | 156952.7 |
| RPOC_SHESH_1 | A8G1F4 | 6.19 | 155364.4 |
| RNA Polymerase subunit omega | Accession number | pI | Molecular Weight |
| RPOZ_ECOLI_1 | P0A800 | 4.87 | 10105.38 |
| RPOZ_THEAQ_1 | Q9EVV4 | 5.5 | 11494.23 |
| RPOZ_MYCTU_1 | P9WGY5 | 4.33 | 11842.22 |
| RPOZ_MYCS2_1 | A0QWT1 | 4.41 | 11534.83 |
| RPOZ_BRADU_1 | Q9RH70 | 4.12 | 14353.86 |
| RPOZ_STRCO_1 | Q9KXS1 | 4.66 | 9709 |
| RPOZ_STRKA_1 | Q8GAU3 | 4.66 | 9723.03 |
| RPOZ_STRP1_1 | P68840 | 5.53 | 11705.38 |
| RPOZ_FRACC_1 | Q2J841 | 4.66 | 10078.38 |
| RPOZ_AGRFC_1 | Q8UGK8 | 4.07 | 14296.62 |
| RPOZ_DEIRA_1 | Q9RRJ6 | 9.68 | 11399.92 |
| RPOZ_STRR6_1 | P66731 | 6.2 | 11792.43 |
| RPOZ_HELPY_1 | P60325 | 9.45 | 8504.88 |
| RPOZ_CORGL_1 | Q8NQ43 | 4.83 | 10520.94 |
| RPOZ_BACSU_1 | O35011 | 5.6 | 7753.9 |
| RPOZ_STRA5_1 | P66729 | 6.76 | 11621.32 |
| RPOZ_ACIBC_1 | B2HZW5 | 5.06 | 10444.8 |
| RPOZ_BURTA_1 | Q2SY71 | 9.74 | 7427.76 |
| RPOZ_SHIB3_1 | B2TTX9 | 4.87 | 10236.57 |
| RPOZ_SODGM_1 | Q2NQS8 | 4.85 | 10214.55 |
| RPOZ_ALIF1_1 | Q5E8P6 | 4.99 | 10102.35 |
| RPOZ_VIBVU_1 | Q8DDV5 | 5.03 | 10057.35 |
| RPOZ_ALIFM_1 | B5FFD6 | 4.99 | 10102.35 |
| RPOZ_VIBVY_1 | Q7MPX0 | 5.03 | 10057.35 |
| RPOZ_ALISL_1 | B6EPJ3 | 4.97 | 10082.3 |
| RPOZ_WIGBR_1 | Q8D2E9 | 6.2 | 7121.26 |
| RPOZ_LACS1_1 | Q1WUB4 | 4.74 | 7183.18 |
| RPOZ_ALKMQ_1 | A6TRX1 | 7.96 | 7569.65 |
| RPOZ_PSEMY_1 | A4Y0M0 | 4.26 | 9733.96 |
| RPOZ_SULNB_1 | A6QB34 | 5.33 | 7494.62 |
| RPOZ_SALA4_1 | B5EY10 | 4.87 | 10236.57 |
| RPOZ_BORPD_1 | A9ILJ1 | 9.85 | 7428.62 |
| RPOZ_SYNJA_1 | Q2JXT8 | 5.4 | 9335.58 |
| RPOZ_PSESM_1 | Q88BE3 | 4.38 | 9710.01 |
| RPOZ_CLOAB_1 | Q97IC9 | 4.92 | 7927.12 |
| RPOZ_MAGSA_1 | Q2W519 | 3.99 | 13944.35 |
| RPOZ_HELMI_1 | B0TGS4 | 10 | 7474.88 |
| RPOZ_YERPN_1 | Q1CCZ2 | 4.99 | 10164.49 |
| RPOZ_CLOBB_1 | B2THR8 | 4.97 | 7425.54 |
| RPOZ_BACC4_1 | B7H6K7 | 5.26 | 7688.87 |
| RPOZ_RHOCS_1 | B6IN07 | 4.01 | 15317.81 |
| RPOZ_STRM5_1 | B4STZ5 | 4.46 | 11146.48 |
| RPOZ_RHOJR_1 | Q0S0L6 | 4.81 | 10874.23 |
| RPOZ_KLEP7_1 | A6TFP8 | 4.87 | 10236.57 |
| RPOZ_TRIEI_1 | Q10YY8 | 6.28 | 9554.76 |
| RPOZ_COREF_1 | Q8FT46 | 4.67 | 10588.05 |
| RPOZ_CROS8_1 | A7MQB3 | 4.87 | 10236.57 |
| RPOZ_SHESW_1 | A1RPI1 | 4.84 | 10124.56 |
| RPOZ_KOSOT_1 | C5CGE8 | 10.06 | 9092.95 |
| RPOZ_MYCLE_1 | Q9CCQ6 | 4.51 | 11974.44 |
| RPOZ_PROM5_1 | A2BYF5 | 6.06 | 8970.01 |
| RPOZ_PSEA7_1 | A6VEE4 | 4.2 | 9774.92 |
| RPOZ_STRS7_1 | C0MH28 | 6.17 | 11760.53 |
| RPOZ_BIFAA_1 | A1A0T8 | 4.21 | 10354.51 |
| RPOZ_ACTPJ_1 | B0BSZ8 | 4.66 | 10403.78 |
| RPOZ_CAMC1_1 | A7ZEY3 | 6.21 | 7599.87 |
| RPOZ_DEIGD_1 | Q1J1S9 | 9.98 | 11350.01 |
| RPOZ_CAMJ8_1 | A8FMX9 | 6.26 | 8317.57 |
| RPOZ_DESAA_1 | B8F9J5 | 6.56 | 7832.13 |
| RPOZ_LACLM_1 | A2RN34 | 6.77 | 13432.51 |
| RPOZ_FRATT_1 | Q5NGX3 | 4.83 | 8183.37 |
| RPOZ_VIBTL_1 | B7VHI0 | 4.99 | 10011.28 |
| RPOZ_ROSDO_1 | Q16AJ3 | 4.34 | 13063.5 |
| RPOZ_DICNV_1 | A5EXW8 | 6.26 | 8539.78 |
| RPOZ_CHESB_1 | Q11JT3 | 4.21 | 14612.17 |
| RPOZ_STAAM_1 | P66725 | 5.76 | 8150.25 |
| RPOZ_PSEPK_1 | Q88C82 | 4.4 | 9745.93 |
| RPOZ_CHRVO_1 | Q7NRL2 | 10 | 7373.49 |
| RPOZ_XANOR_1 | Q5H3R9 | 4.57 | 11098.54 |
| RPOZ_HAEIE_1 | A5UBD9 | 4.76 | 9888.35 |
| RPOZ_LYSSC_1 | B1HQE0 | 5.63 | 8562.67 |
| RPOZ_RALSO_1 | Q8XXG0 | 9.65 | 7387.69 |
| RPOZ_YERPB_1 | B2JYM2 | 4.87 | 10165.48 |
| RPOZ_CLOB8_1 | A6LSJ4 | 4.81 | 8092.31 |
| RPOZ_RHIEC_1 | Q2KAE9 | 4.1 | 14696.21 |
| RPOZ_THEAB_1 | B7IFG6 | 9.19 | 8765.34 |
| RPOZ_PEDPA_1 | Q03FY6 | 4.5 | 7786.85 |
| RPOZ_BURCH_1 | A0K5H6 | 9.74 | 7427.76 |
| RPOZ_RHOBA_1 | Q7UP93 | 4.34 | 10121.56 |
| RPOZ_SHEB5_1 | A3CZH4 | 4.84 | 10140.56 |
| RPOZ_PEPD6_1 | Q182S6 | 5.41 | 10197.52 |
| RPOZ_THENN_1 | B9K7W8 | 7.89 | 9335.13 |
| RPOZ_PERMH_1 | C0QR95 | 9.57 | 7692.99 |
| RPOZ_RHOFT_1 | Q21TP6 | 9.69 | 7417.89 |
| RPOZ_CLONN_1 | A0Q119 | 5.18 | 8153.27 |
| RPOZ_CORJK_1 | Q4JVH5 | 4.51 | 10423.74 |
| RPOZ_SHEVD_1 | Q7WZE4 | 4.54 | 10327.7 |
| RPOZ_FRASN_1 | A8LE17 | 4.51 | 10170.51 |
| RPOZ_MYCVP_1 | A1T8H5 | 4.51 | 11348.72 |
| RPOZ_VIBCH_1 | Q9KNM3 | 4.99 | 10054.37 |
| RPOZ_RICPR_1 | Q9ZCX4 | 4.67 | 14896.89 |
| RPOZ_AGRVS_1 | B9JUN3 | 4.1 | 14674.08 |
| RPOZ_DESDA_1 | B8J412 | 4.89 | 8798.97 |
| RPOZ_CARHZ_1 | Q3AC15 | 10.17 | 7721.97 |
| RPOZ_PSEE4_1 | Q1I2S9 | 4.4 | 9728.94 |
| RPOZ_GEOMG_1 | Q39T73 | 9.93 | 7665.06 |
| RPOZ_RUEST_1 | Q1GDG8 | 4.28 | 13026.46 |
| RPOZ_GEOSL_1 | Q74AW2 | 10.42 | 7804.18 |
| RPOZ_NITWN_1 | Q3SRA8 | 4.13 | 14400.93 |
| RPOZ_ANAMM_1 | Q5PB40 | 7.71 | 13923.57 |
| RPOZ_LISMC_1 | C1KWC5 | 7.96 | 7714.98 |
| RPOZ_SALNS_1 | B4SXF1 | 4.87 | 10236.57 |
| RPOZ_HAES1_1 | Q0I5L9 | 5.37 | 10360.76 |
| RPOZ_AZOVD_1 | C1DI45 | 4.33 | 9702.95 |
| RPOZ_PARPJ_1 | B2T6B2 | 9.74 | 7427.76 |
| RPOZ_SALTY_1 | P0A803 | 4.87 | 10236.57 |
| RPOZ_STRAW_1 | Q827Q2 | 4.66 | 9735.04 |
| RPOZ_RHILW_1 | B5ZW67 | 4.1 | 14724.26 |
| RPOZ_PELPD_1 | A1ARN6 | 10.28 | 8073.58 |
| RPOZ_PELTS_1 | A5D1C3 | 9.99 | 7603.89 |
| RPOZ_THEMA_1 | Q9X214 | 9.13 | 9350.16 |
| RPOZ_HERAR_1 | A4G6Y1 | 9.69 | 7497.91 |
| RPOZ_METPP_1 | A2SJD1 | 10.01 | 7440.82 |
| RPOZ_THEP3_1 | B0KA14 | 8.76 | 7872.23 |
| RPOZ_BURM9_1 | A2S9I5 | 9.74 | 7399.71 |
| RPOZ_HYPNA_1 | Q0C3U4 | 4.69 | 13035.68 |
| RPOZ_EHRCJ_1 | Q3YSH8 | 9.18 | 15431.37 |
| RPOZ_ERWT9_1 | B2VL59 | 4.99 | 10204.53 |
| RPOZ_SHEON_1 | Q8EJU7 | 4.84 | 10124.56 |
| RPOZ_SHEPA_1 | A8H9F1 | 4.62 | 10312.68 |
| RPOZ_RHOS5_1 | A4WVQ1 | 4.41 | 13221.79 |
| RPOZ_CYAP7_1 | B7KF62 | 5.6 | 8935.09 |
| RPOZ_RICAE_1 | C3PP13 | 4.67 | 14767.72 |
| RPOZ_MYXXD_1 | Q1D2S6 | 9.89 | 8663.13 |
| RPOZ_BARHE_1 | Q6G465 | 4.33 | 14503.96 |
| RPOZ_LACJO_1 | Q74IM7 | 8.05 | 7872.04 |
| RPOZ_RICCN_1 | Q92H89 | 4.67 | 14767.72 |
| RPOZ_SPHAL_1 | Q1GSQ7 | 4.76 | 12316.77 |
| RPOZ_NITEU_1 | Q82SQ2 | 6.19 | 7573.83 |
| RPOZ_BIFLO_1 | Q8G3H3 | 4.23 | 10366.49 |
| RPOZ_NITHX_1 | Q1QL49 | 4.19 | 14486.04 |
| RPOZ_CAUVC_1 | P58066 | 4.64 | 13271.85 |
| RPOZ_GEOSM_1 | C6E2G6 | 10.22 | 7540.99 |
| RPOZ_BORPA_1 | Q7W6B1 | 9.85 | 7448.69 |
| RPOZ_GLOVI_1 | Q7NK60 | 4.73 | 8524.58 |
| RPOZ_GLUDA_1 | A9HKY5 | 4.43 | 14534.22 |
| RPOZ_SALCH_1 | Q57I91 | 4.87 | 10236.57 |
| RPOZ_NOVAD_1 | Q2GC33 | 4.43 | 12370.85 |
| RPOZ_ANASK_1 | B4UD71 | 11.18 | 9775.53 |
| RPOZ_CITK8_1 | A8ARN6 | 4.87 | 10236.57 |
| RPOZ_OENOB_1 | Q04EL5 | 5.6 | 12218.05 |
| RPOZ_PSYA2_1 | Q4FQY6 | 4.96 | 9443.72 |
| RPOZ_STAHJ_1 | Q4L5R2 | 5.72 | 7641.74 |
| RPOZ_STAS1_1 | Q49WZ2 | 6.21 | 7837.11 |
| RPOZ_BRUO2_1 | A5VPI8 | 4.22 | 14514.07 |
| RPOZ_CUPTR_1 | B3R3N5 | 9.65 | 7438.79 |
| RPOZ_NEIMB_1 | P66724 | 9.18 | 7499.51 |
| RPOZ_RICRS_1 | A8GSU9 | 4.75 | 14756.74 |
| RPOZ_CAMLR_1 | B9KFW0 | 7.93 | 8157.5 |
| RPOZ_LEPCP_1 | B1Y0M2 | 10.28 | 7315.7 |
| RPOZ_BORPE_1 | Q7VXZ4 | 9.85 | 7476.75 |
| RPOZ_SALDC_1 | B5FM73 | 4.87 | 10236.57 |
| RPOZ_LISW6_1 | A0AJT1 | 8.93 | 7597.86 |
| RPOZ_CLAMS_1 | B0REV6 | 4.41 | 9769.07 |
| RPOZ_PSYWF_1 | A5WCZ0 | 5.69 | 9286.6 |
| RPOZ_YERE8_1 | A1JHV7 | 4.87 | 10165.48 |
| RPOZ_SYNWW_1 | Q0AXK9 | 6.25 | 7971.09 |
| RPOZ_THEP1_1 | A5ILC9 | 9.36 | 9301.15 |
| RPOZ_HYDCU_1 | Q31DP2 | 4.53 | 8379.5 |
| RPOZ_MICAN_1 | B0JNU3 | 6.23 | 8871.25 |
| RPOZ_BURP0_1 | A3NY34 | 9.74 | 7399.71 |
| RPOZ_SHEFN_1 | Q088T8 | 4.97 | 10087.58 |
| RPOZ_CLOP1_1 | Q0TPK7 | 4.65 | 7971.05 |
| RPOZ_TREPA_1 | O83699 | 4.33 | 7349.44 |
| RPOZ_ACICJ_1 | A5FVX2 | 4.17 | 14776.35 |
| RPOZ_BACHK_1 | Q6HEU5 | 5.26 | 7751.98 |
| RPOZ_LACAC_1 | Q5FJH3 | 5.5 | 8088.2 |
| RPOZ_BACSK_1 | Q5WFK4 | 7.96 | 8094.38 |
| RPOZ_DEIDV_1 | C1CZ29 | 10.09 | 11410.02 |
| RPOZ_CAMC5_1 | A7GXE4 | 6.22 | 7632.92 |
| RPOZ_LACH4_1 | A8YVW4 | 5.49 | 8074.18 |
| RPOZ_NEIG2_1 | B4RN20 | 9.18 | 7499.51 |
| RPOZ_BDEBA_1 | Q6MMQ7 | 9.25 | 8796.2 |
| RPOZ_BIFA0_1 | B8DTW7 | 4.27 | 10367.6 |
| RPOZ_NEOSM_1 | Q2GF15 | 4.56 | 14458.79 |
| RPOZ_STRT2_1 | Q5M3I5 | 7.94 | 11775.67 |
| RPOZ_GEOKA_1 | Q5L0S7 | 6.25 | 7463.82 |
| RPOZ_RUBXD_1 | Q1AVZ4 | 5.22 | 11557.97 |
| RPOZ_GEOLS_1 | B3E4R5 | 10.1 | 7631.04 |
| RPOZ_STRU0_1 | B9DV47 | 7.93 | 11751.56 |
| RPOZ_LACSS_1 | Q38XT9 | 4.89 | 9401.82 |
| RPOZ_LEGPA_1 | Q5X3P3 | 4.7 | 7671.76 |
| RPOZ_XANAC_1 | P66732 | 4.5 | 11142.55 |
| RPOZ_DICTD_1 | B8E0X9 | 4.85 | 7708.01 |
| RPOZ_SYNAS_1 | Q2LQ83 | 5.36 | 10324.81 |
| RPOZ_NOSP7_1 | B2IUW7 | 6.59 | 9199.53 |
| RPOZ_SALG2_1 | B5RG73 | 4.87 | 10236.57 |
| RPOZ_MAGMM_1 | A0L486 | 4.12 | 13371.81 |
| RPOZ_HELAH_1 | Q17Y32 | 9.45 | 8504.88 |
| RPOZ_SALTO_1 | A4X624 | 4.64 | 9531.86 |
| RPOZ_HELHP_1 | Q7VHY0 | 5.15 | 8473.81 |
| RPOZ_RHILO_1 | Q985B3 | 4.13 | 14638.07 |
| RPOZ_METRJ_1 | B1LTE4 | 4.25 | 14882.56 |
| RPOZ_PHOPR_1 | Q6LVP6 | 5.29 | 10579.86 |
| RPOZ_THESQ_1 | B1LAZ3 | 9.13 | 9350.16 |
| RPOZ_CLOK1_1 | B9E1D8 | 8.01 | 8065.39 |
| RPOZ_MYCBT_1 | C1AN35 | 4.33 | 11842.22 |
| RPOZ_VEREI_1 | A1WQL7 | 10.28 | 7428.81 |
| RPOZ_VESOH_1 | A5CX12 | 4.54 | 12165.89 |
| RPOZ_COXBU_1 | Q83EL6 | 4.73 | 10759.11 |
| RPOZ_CUPNH_1 | Q0KD22 | 9.65 | 7438.79 |
| RPOZ_BACCN_1 | A7GRK0 | 5.04 | 7806 |
| RPOZ_MYCPA_1 | Q741G7 | 4.43 | 11695.09 |
| RPOZ_LACDA_1 | Q1G9H9 | 4.66 | 7834.75 |
| RPOZ_DECAR_1 | Q479A9 | 10.17 | 7361.64 |
| RPOZ_RICAH_1 | A8GP45 | 4.56 | 14811.74 |
| RPOZ_BART1_1 | A9IRL2 | 4.33 | 14754.21 |
| RPOZ_AGRRK_1 | B9JC68 | 4.11 | 14624.04 |
| RPOZ_STRSV_1 | A3CPX6 | 5.83 | 11948.67 |
| RPOZ_PROMH_1 | B4EZA3 | 5.03 | 10163.57 |
| RPOZ_SHIDS_1 | Q329K7 | 4.87 | 10236.57 |
| RPOZ_DELAS_1 | A9BTA2 | 9.74 | 7475.82 |
| RPOZ_SHIF8_1 | Q0SYH7 | 4.87 | 10236.57 |
| RPOZ_ALCBS_1 | Q0VT95 | 4.57 | 9532.72 |
| RPOZ_GEOBB_1 | B5E9N7 | 10.22 | 7524.99 |
| RPOZ_GEODF_1 | B9M902 | 10.43 | 7773.21 |
| RPOZ_NITEC_1 | Q0AD35 | 10.16 | 7539.91 |
| RPOZ_CAUSK_1 | B0T3H0 | 4.57 | 13302.85 |
| RPOZ_DESVH_1 | Q725M7 | 5.14 | 8851.25 |
| RPOZ_PSEFS_1 | C3K482 | 4.31 | 9827.06 |
| RPOZ_BORA1_1 | Q2L066 | 9.85 | 7448.69 |
| RPOZ_SACEN_1 | A4FBI2 | 4.72 | 10503.83 |
| RPOZ_GEOUR_1 | A5G6A4 | 10.43 | 7727.18 |
| RPOZ_CHRSD_1 | Q1QSI0 | 5.07 | 9389.63 |
| RPOZ_XANCP_1 | P66733 | 4.5 | 11142.55 |
| RPOZ_OCHA4_1 | A6X296 | 4.17 | 14681.2 |
| RPOZ_PARXL_1 | Q13V50 | 9.74 | 7427.76 |
| RPOZ_MARMM_1 | Q0APB9 | 4.45 | 13706.36 |
| RPOZ_SYNY3_1 | P74352 | 5.32 | 8736.77 |
| RPOZ_BRUSU_1 | Q8G1Q6 | 4.22 | 14542.12 |
| RPOZ_BURL3_1 | Q39IL1 | 9.74 | 7427.76 |
| RPOZ_BURM1_1 | A9AEQ4 | 9.74 | 7441.79 |
| RPOZ_POLAQ_1 | A4SXS9 | 9.61 | 7477.74 |
| RPOZ_POLNA_1 | A1VKF1 | 10.12 | 7418.82 |
| RPOZ_RHOPA_1 | Q6N6C5 | 4.12 | 14367.94 |
| RPOZ_EHRRW_1 | Q5HBN0 | 6.9 | 15007.99 |
| RPOZ_THEYD_1 | B5YJ35 | 5.64 | 15558.91 |
| RPOZ_KINRD_1 | A6WCB6 | 4.51 | 9532.8 |
| RPOZ_RHORT_1 | Q2RT88 | 3.87 | 14917.21 |
| RPOZ_TROW8_1 | Q820D7 | 4.69 | 9373.49 |
| RPOZ_ACIF5_1 | B5EN39 | 4.93 | 9676.88 |
| RPOZ_RICPU_1 | C4K1J3 | 4.67 | 14757.68 |
| RPOZ_ACIAC_1 | A1TT47 | 9.74 | 7468.83 |
| RPOZ_ESCF3_1 | B7LVL1 | 4.87 | 10236.57 |
| RPOE_SALTI_1 | P0A2F1 | 5.38 | 21711.74 |
| RPOZ_BACP2_1 | A8FD34 | 4.81 | 7747.75 |
| RPOZ_LACCB_1 | B3WEX2 | 5.39 | 9204.62 |
| RPOZ_CALS4_1 | Q8R9S7 | 8.73 | 7961.37 |
| RPOZ_BACWK_1 | A9VTA8 | 4.73 | 7815.1 |
| RPOZ_DESAD_1 | C6BYN6 | 8.82 | 8178.48 |
| RPOZ_DESAG_1 | Q316U8 | 6.27 | 8231.56 |
| RPOZ_VIBPA_1 | Q87TB0 | 5.01 | 10022.31 |
| RPOZ_DESAH_1 | C0QKM9 | 10.12 | 7596 |
| RPOZ_LACRD_1 | A5VKQ8 | 4.63 | 7921.97 |
| RPOZ_DESHY_1 | Q24TX5 | 9 | 7744.99 |
| RPOZ_DESMR_1 | C4XRE2 | 5.33 | 8362.75 |
| RPOZ_WOLPP_1 | B3CMP5 | 5.02 | 14808.55 |
| RPOZ_WOLSU_1 | Q7MSQ0 | 8.06 | 8085.5 |
| RPOZ_SACD2_1 | Q21EC8 | 4.21 | 9969.14 |
| RPOZ_GEOSW_1 | C5D8R2 | 5.76 | 7441.67 |
| RPOZ_NOCSJ_1 | A1SJF7 | 4.27 | 11740.09 |
| RPOZ_OCEIH_1 | Q8ER27 | 9.45 | 7322.53 |
| RPOZ_AQUAE_1 | O66570 | 8.01 | 8801.1 |
| RPOZ_XYLFA_1 | Q9PD77 | 4.6 | 11235.73 |
| RPOZ_MANSM_1 | Q65RR6 | 4.97 | 10015.44 |
| RPOZ_HALHL_1 | A1WVN4 | 4.11 | 12751.88 |
| RPOZ_SALSV_1 | B4U000 | 4.87 | 10236.57 |
| RPOZ_HAMD5_1 | C4K473 | 6.73 | 10225.72 |
| RPOZ_SERP5_1 | A8GLH0 | 4.85 | 10202.49 |
| RPOZ_HISS2_1 | B0URZ0 | 5.37 | 10360.76 |
| RPOZ_IDILO_1 | Q5QYI0 | 4.76 | 10036.31 |
| RPOZ_MOOTA_1 | Q2RK30 | 10 | 7411.8 |
| RPOZ_ALKOO_1 | A8MH81 | 4.61 | 8037.19 |
| RPOZ_RUTMC_1 | A1AW62 | 4.9 | 8812.04 |
| RPOZ_WOLTR_1 | Q5GSP9 | 4.33 | 16145.47 |
| RPOZ_DICT6_1 | B5YF48 | 4.82 | 7664.9 |
| RPOZ_NOCFA_1 | Q5YTM8 | 4.61 | 10084.34 |
| RPOZ_SALEP_1 | B5QTY1 | 4.87 | 10236.57 |
| RPOZ_XANP2_1 | A7IM18 | 4.17 | 14179.65 |
| RPOZ_OLICO_1 | B6JGF6 | 4.13 | 14333.91 |
| RPOZ_SALPK_1 | B5BI24 | 4.87 | 10236.57 |
| RPOZ_TERTT_1 | C5BLF6 | 4.38 | 10355.64 |
| RPOZ_PELCD_1 | Q3A522 | 9.93 | 7736.16 |
| RPOZ_THEFY_1 | Q47R16 | 4.41 | 9596.93 |
| RPOZ_ZYMMO_1 | Q5NLR6 | 4.56 | 13530.08 |
| RPOZ_PHOLL_1 | Q7N9P3 | 5.03 | 10155.5 |
| RPOZ_RHOOB_1 | C1B4J5 | 4.81 | 10902.28 |
| RPOZ_THET2_1 | Q72ID6 | 5.23 | 11517.32 |
| RPOZ_SHEHH_1 | B0TPU5 | 4.62 | 10170.53 |
| RPOZ_SHELP_1 | A3QIR6 | 4.62 | 10200.55 |
| RPOZ_VARPS_1 | C5CYY0 | 9.89 | 7415.85 |
| RPOZ_CORU7_1 | B1VDN5 | 4.42 | 10352.67 |
| RPOZ_SHESH_1 | A8FQ25 | 4.62 | 10312.68 |
| RPOZ_CUPMC_1 | Q1LQ33 | 9.65 | 7438.79 |
| RPOZ_BACLD_1 | Q65JS9 | 6.56 | 7553.67 |
| RPOZ_LACBA_1 | Q03RS0 | 4.55 | 7760.91 |
| RPOZ_FRAAA_1 | Q0RF85 | 4.66 | 10106.43 |
| RPOZ_ACTSZ_1 | A6VN44 | 5.04 | 9914.27 |
| RPOZ_BACVZ_1 | A7Z4I9 | 5.25 | 7709.74 |
| RPOZ_AERHH_1 | A0KR23 | 4.57 | 10015.27 |
| RPOZ_BARQU_1 | Q6G088 | 4.24 | 14677.2 |
| RPOZ_SHISS_1 | Q3YW12 | 4.87 | 10236.57 |
| RPOZ_BRASB_1 | A5EKM2 | 4.12 | 14309.85 |
| RPOZ_ANAVT_1 | Q3MBJ9 | 6.59 | 9145.47 |
| RPOZ_AROAE_1 | Q5P3L9 | 9.74 | 7878.19 |
| RPOZ_PASMU_1 | Q9CMB2 | 5.66 | 10116.55 |
| RPOZ_PECCP_1 | C6DJC5 | 4.85 | 10268.56 |
| RPOZ_SHEAM_1 | A1S270 | 5.18 | 10041.46 |
| RPOZ_PETMO_1 | A9BEX2 | 9.52 | 8150.6 |
| RPOZ_METS4_1 | B0UN75 | 4.2 | 15002.61 |
| RPOZ_CLOCE_1 | B8I2Z7 | 9.13 | 7620.88 |
| RPOZ_JANMA_1 | A6SXM0 | 9.89 | 7481.85 |
| RPOZ_TOLAT_1 | C4L810 | 4.51 | 10541.81 |
| RPOZ_CLOTE_1 | P60332 | 4.76 | 8002.19 |
| RPOZ_SHEPC_1 | A4YBK6 | 4.84 | 10124.56 |
| RPOZ_CORDI_1 | P60333 | 4.51 | 10076.3 |
| RPOZ_ACIC1_1 | A0LUF4 | 4.64 | 9473.95 |
| RPOZ_EXIS2_1 | B1YIQ5 | 9.85 | 7444.66 |
| RPOZ_LACF3_1 | B2GD58 | 4.81 | 10704.1 |
| RPOZ_LACGA_1 | Q044H2 | 9.35 | 7871.1 |
| RPOZ_NAUPA_1 | B9L8H3 | 8.04 | 7601.98 |
| RPOZ_STRSY_1 | A4VTF1 | 6.19 | 11791.54 |
| RPOZ_RUEPO_1 | Q5LNK0 | 4.25 | 13076.51 |
| RPOZ_ALTMD_1 | B4S2X2 | 4.43 | 9861.01 |
| RPOZ_ANADE_1 | Q2IFJ5 | 11.18 | 9775.53 |
| RPOZ_SYNE7_1 | Q31MH9 | 4.86 | 8777.99 |
| RPOZ_BRUA1_1 | B2SAC9 | 4.22 | 14514.07 |
| RPOZ_STACT_1 | B9DPL6 | 5.64 | 8516.75 |
| RPOZ_BRUMB_1 | C0RHZ3 | 4.22 | 14514.07 |
| RPOZ_METEP_1 | A9VYZ2 | 4.27 | 14989.67 |
| RPOZ_THEM4_1 | A6LJ74 | 7.93 | 8750.24 |
| RPOZ_THEPX_1 | B0K295 | 7.82 | 7872.19 |
| RPOZ_EHRCR_1 | Q2GG39 | 9.05 | 15382.28 |
| RPOZ_SHEDO_1 | Q12IL7 | 4.65 | 10062.44 |
| RPOZ_ENT38_1 | A4W500 | 4.87 | 10211.52 |
| RPOZ_THIDA_1 | Q3SLI5 | 9.61 | 7438.62 |
| RPOZ_ERYLH_1 | Q2N7Q3 | 4.42 | 12441.9 |
| RPOZ_ACAM1_1 | B0C118 | 8.16 | 8850.1 |
| RPOZ_BACHD_1 | Q9K9Y3 | 5.77 | 7819.97 |
| RPOZ_LACP3_1 | Q038H0 | 5.39 | 9204.62 |
| RPOZ_BEUC1_1 | C5C692 | 4.48 | 9812.09 |
| RPOZ_RICTY_1 | Q68WF8 | 4.59 | 14769.64 |
| RPOZ_LARHH_1 | C1DC35 | 10 | 7863.1 |
| RPOZ_DINSH_1 | A8LLD2 | 4.3 | 13153.67 |
| RPOZ_LISIN_1 | Q92AI2 | 7.97 | 7597.81 |
| RPOZ_NOSS1_1 | Q8YNB9 | 6.59 | 9129.48 |
| RPOZ_ANAPZ_1 | Q2GKJ2 | 6.9 | 14639.18 |
| RPOZ_BREBN_1 | C0ZG01 | 6.58 | 7866.07 |
| RPOZ_PSEU5_1 | A4VGR5 | 4.2 | 9808.98 |
| RPOZ_PSYCK_1 | Q1Q972 | 4.96 | 9443.72 |
| RPOZ_PARL1_1 | A7HX41 | 4.23 | 15477.14 |
| RPOZ_MARMS_1 | A6W3K1 | 5.33 | 8290.48 |
| RPOZ_RHOE4_1 | C0ZZD2 | 4.58 | 10684.97 |
| RPOZ_METSB_1 | B8EK21 | 4.18 | 14246.84 |
| RPOZ_POLNS_1 | B1XUH5 | 9.61 | 7477.74 |
| RPOZ_SHEPW_1 | B8CHM9 | 4.74 | 10170.57 |
| RPOZ_FRAP2_1 | B0TZ05 | 4.78 | 8095.2 |
| RPOZ_RICB8_1 | A8GW64 | 4.58 | 14772.5 |
| RPOZ_RICCK_1 | A8EYA7 | 4.58 | 14732.59 |
| RPOZ_PSEA6_1 | Q15Z09 | 5.04 | 9861.04 |
| RPOZ_SINMW_1 | A6U7A2 | 4.19 | 14932.51 |
| RPOZ_SPHWW_1 | A5VC58 | 4.49 | 12312.71 |
| RPOZ_ALKEH_1 | Q0A5V4 | 4.32 | 11484.61 |
| RPOZ_NITMU_1 | Q2YD11 | 9.18 | 7348.67 |
| RPOZ_PSEHT_1 | Q3IJH7 | 4.61 | 9774.06 |
| RPOZ_PARDP_1 | A1B1V8 | 4.37 | 13389.06 |
| RPOZ_PSYIN_1 | A1T0P2 | 4.95 | 10094.47 |
| RPOZ_BRUC2_1 | A9MA26 | 4.22 | 14542.12 |
| RPOZ_BACAC_1 | C3L757 | 5.26 | 7751.98 |
| RPOZ_PECAS_1 | Q6DB61 | 4.83 | 10254.53 |
| RPOZ_RHIME_1 | Q92R52 | 4.19 | 14902.49 |
| RPOZ_METPB_1 | B1ZJQ8 | 4.3 | 14999.66 |
| RPOZ_EDWI9_1 | C5B9B3 | 4.89 | 10252.57 |
| RPOZ_ENTFA_1 | Q82ZD6 | 5.36 | 11540.97 |
| RPOZ_AERS4_1 | A4SH73 | 4.57 | 10015.27 |
| RPOZ_BARBK_1 | A1US30 | 4.28 | 14788.27 |
| RPOZ_AGARV_1 | C4Z9X7 | 4.65 | 9443.73 |
| RPOZ_BLOPB_1 | Q491W4 | 9.22 | 7157.36 |
| RPOZ_NITSB_1 | A6Q312 | 6.28 | 7882.25 |
| RPOZ_BORBR_1 | Q7WI79 | 9.85 | 7448.69 |
| RPOZ_SALAI_1 | A8LY23 | 4.64 | 9545.88 |
| RPOZ_SYNFM_1 | A0LJ43 | 4.62 | 13510.41 |
| RPOZ_HAEPS_1 | B8F5Q2 | 4.71 | 10457.87 |
| RPOZ_AZOSB_1 | A1KCL2 | 9.51 | 7829.1 |
| RPOZ_HAHCH_1 | Q2S8R3 | 4.59 | 7904.94 |
| RPOZ_ACIAD_1 | Q6F7G9 | 4.87 | 10542.8 |
| RPOZ_ACIET_1 | B9MEK5 | 10.01 | 7387.76 |
| RPOZ_CUTAK_1 | Q6A8H5 | 4.2 | 12483.97 |
| RPOZ_FINM2_1 | B0S136 | 9.56 | 7485.82 |
| RPOZ_PSE14_1 | Q48Q16 | 4.38 | 9710.01 |
| RPOZ_LACP7_1 | A9KPG3 | 4.54 | 9147.22 |
| RPOZ_LACPL_1 | Q88WL6 | 4.58 | 7742.89 |
| RPOZ_NITOC_1 | Q3JBT2 | 4.81 | 9502.73 |
| RPOZ_SYMTH_1 | Q67PR8 | 9.52 | 7703.06 |
| RPOZ_LEIXX_1 | Q6AF81 | 4.5 | 9528.77 |
| RPOZ_SALAR_1 | A9MKM3 | 4.87 | 10236.57 |
| RPOZ_SALHS_1 | B4T9Z3 | 4.87 | 10236.57 |
| RPOZ_AZOC5_1 | A8I0F9 | 4.06 | 14054.53 |
| RPOZ_RALPJ_1 | B2U8D3 | 9.65 | 7403.69 |
| RPOZ_MARHV_1 | A1TYB4 | 4.52 | 7885.98 |
| RPOZ_METFK_1 | Q1GXB8 | 9.51 | 7590.84 |
| RPOZ_METNO_1 | B8IT21 | 4.22 | 14863.45 |
| RPOZ_STRMU_1 | Q8DVK5 | 5.51 | 11731.44 |
| RPOZ_JANSC_1 | Q28V32 | 4.3 | 13258.71 |
| RPOZ_MYCA1_1 | A0QI28 | 4.43 | 11679.05 |
| RPOZ_POLSJ_1 | Q12DV8 | 9.8 | 7421.81 |
| RPOZ_THISH_1 | B8GQN1 | 4.96 | 9706.97 |
| RPOZ_ACISJ_1 | A1W4L3 | 10.01 | 7387.76 |
| RPOZ_BURVG_1 | A4JCC5 | 9.74 | 7427.76 |
| RPOZ_SHEWM_1 | B1KLV2 | 4.56 | 10327.7 |
| RPOZ_BAUCH_1 | Q1LTY2 | 9.51 | 7265.45 |
| RPOZ_BEII9_1 | B2IIA1 | 4.14 | 14339.88 |
| RPOZ_HAEDU_1 | Q7VKI2 | 4.69 | 11417.84 |
| RPOZ_PARP8_1 | B2JEI3 | 9.74 | 7427.76 |
| RPOZ_METCA_1 | Q606J4 | 5.36 | 10711.29 |
| RPOZ_PELUB_1 | Q4FLD7 | 4.15 | 19777.58 |
| RPOZ_STRGG_1 | B1W468 | 4.66 | 9753.05 |
| Sigma-24 | Accession number | pI | Molecular Weight |
| RPOE_ECOLI_1 | P0AGB6 | 5.38 | 21695.74 |
| RPOE_RHOS4_1 | Q3IYV6 | 6.53 | 20703.49 |
| RPOE_SALTY_1 | P0A2F0 | 5.38 | 21711.74 |
| RPOE_HAEIN_1 | P44790 | 4.86 | 21791.62 |
| RPOE_SHIFL_1 | P0AGB9 | 5.38 | 21695.74 |
| Sigma-28 | Accession number | pI | Molecular Weight |
| FLIA_ECOLI_1 | P0AEM6 | 5.2 | 27521.11 |
| RP28_BACTK_1 | P62181 | 6.06 | 24987.85 |
| RP28_BACCR_1 | P62180 | 6.06 | 24987.85 |
| RP28_BACAN_1 | P62182 | 6.06 | 24987.85 |
| FLIA_YEREN_1 | P52621 | 5.3 | 26579.98 |
| FLIA_PSEAE_1 | P29248 | 5.7 | 27522.09 |
| FLIA_SALTY_1 | P0A2E8 | 5.12 | 27473.04 |
| Sigma-32 | Accession number | pI | Molecular Weight |
| RPOH_ECOLI_1 | P0AGB3 | 5.64 | 32468.8 |
| RPOH_CAUVC_1 | P0CAW9 | 6.08 | 33528.36 |
| RPOH_VIBCH_1 | P50511 | 5.71 | 32650.1 |
| RPOH_PSEAE_1 | P42378 | 6.03 | 32580.88 |
| RPOH_ZYMMO_1 | P50512 | 5.88 | 34434.51 |
| RPOH_SERMA_1 | P50510 | 5.54 | 32671.08 |
| RPOH_ENTCL_1 | P50508 | 5.45 | 32654.12 |
| RPOH_RHIRD_1 | P50507 | 6.17 | 34418.29 |
| RPOH_PROMI_1 | P50509 | 5.24 | 32559.87 |
| RPOH_BUCAI_1 | O05385 | 9.76 | 32936.84 |
| RPOH_HAEIN_1 | P44404 | 5.34 | 32033.33 |
| RPOH_VIBVU_1 | Q8DD54 | 5.88 | 32325.8 |
| RPOH_CITFR_1 | P11539 | 5.8 | 32572.97 |
| Sigma-38 | Accession number | pI | Molecular Weight |
| RPOS_ECOLI_1 | P13445 | 4.87 | 37971.86 |
| RPOS_PSEAE_1 | P45684 | 5.26 | 38235.38 |
| RPOS_SALTU_1 | F5ZTT9 | 4.85 | 37932.74 |
| RPOS_VIBCH_1 | O51804 | 4.65 | 38534.39 |
| RPOS_YEREN_1 | P47765 | 4.75 | 37967.69 |
| RPOS_SALTI_1 | P0A2E6 | 4.85 | 37932.74 |
| RPOS_SHIFL_1 | P35540 | 4.91 | 37971.9 |
| RPOS_COXBU_1 | Q9KI19 | 9.78 | 40470.85 |
| RPOS_SALDU_1 | P0A2E7 | 4.85 | 37932.74 |
| Sigma-54 | Accession number | pI | Molecular Weight |
| RP54_PSEPK_1 | P0A171 | 4.7 | 56215.2 |
| RP54_ECOLI_1 | P24255 | 4.63 | 53989.78 |
| RP54_SALTY_1 | P26979 | 4.63 | 53987.85 |
| RP54_PSEAE_1 | P49988 | 4.7 | 56054.22 |
| RP54_RHOCB_1 | D5ANH9 | 5.73 | 46384.81 |
| RP55_BRADU_1 | P30333 | 4.78 | 58831.85 |
| RP54_KLEOX_1 | P06223 | 4.6 | 53926.82 |
| RP54_RHIME_1 | P17263 | 4.94 | 56678.96 |
| RP54_ACIGI_1 | P33983 | 5.06 | 54901.49 |
| RP54_CUPNH_1 | P28615 | 5.16 | 54770.07 |
| RP54_ACIFR_1 | P24695 | 4.88 | 52927.77 |
| RP54_AZOVI_1 | P08623 | 4.73 | 56917.07 |
| RP54_CAUVC_1 | Q03408 | 5.01 | 54456.56 |
| RP54_SINFN_1 | P22881 | 4.95 | 57195.52 |
| RP54_SHEVD_1 | Q9S0L2 | 4.79 | 55359.76 |
| RP54_BACSU_1 | P24219 | 7.72 | 49700.68 |
| RP54_CLOK5_1 | P38944 | 5.14 | 53028.61 |
| RP54_XANEU_1 | P77998 | 5.09 | 52217.04 |
| RP54_HELPJ_1 | Q9ZLC8 | 5.92 | 48246.58 |
| RP54_RHOSH_1 | Q01194 | 5.18 | 47870.64 |
| RP54_AZOC5_1 | P33984 | 4.84 | 55887.68 |
| RP54_RHIET_1 | P49989 | 4.61 | 57477.56 |
| RP54_TREPA_1 | O83149 | 9.54 | 54279.8 |
| RP54_VIBAN_1 | O08429 | 4.56 | 54641.6 |
| Sigma-70 | Accession number | pI | Molecular Weight |
| RPOD_ECOLI_1 | P00579 | 4.68 | 70263.28 |
| RPOD_RHIEC_1 | Q2K619 | 5 | 77201.27 |
| RPOD_RHOCA_1 | P0CZ15 | 4.83 | 75935.16 |
| RPOD_AGRFC_1 | P33452 | 4.98 | 77399.44 |
| RPOD_COXBU_1 | Q83BB6 | 9.13 | 79553.23 |
| RPOD_PSEPU_1 | P52327 | 5.08 | 69725.99 |
| RPOD_RICFE_1 | Q4UJT1 | 5.36 | 72958.43 |
| RPOD_CAUVC_1 | P52324 | 4.92 | 72675.2 |
| RPOD_XYLFA_1 | Q9PDM9 | 4.94 | 69907.18 |
| RPSD_BACSU_1 | P10726 | 5.33 | 29467.86 |
| RPOD_HELPY_1 | P55993 | 7.94 | 77737.37 |
| RPOD_LEPIN_1 | P59117 | 9.09 | 67873.32 |
| RPOD_RICBR_1 | Q1RKH7 | 5.24 | 73733.82 |
| RPOD_SALTI_1 | P0A2E4 | 4.7 | 70530.63 |
| RPOD_TREPA_1 | O83506 | 7.63 | 70902.4 |
| RPOD_MYXXA_1 | P17531 | 5.27 | 80398.69 |
| RPOD_NEIGO_1 | P52325 | 4.92 | 73679.27 |
| RPOD_PSEAE_1 | P26480 | 4.9 | 69643.74 |
| RPOD_PSEPH_1 | P52326 | 5.01 | 69436.9 |
| RPOD_RHIME_1 | Q59753 | 5.01 | 77174.29 |
| RPOD_RICCN_1 | Q92FZ8 | 5.35 | 72831.26 |
| RPOD_RICPR_1 | P33451 | 5.39 | 73080.39 |
| RPOD_BUCBP_1 | Q89B10 | 5.98 | 70624.63 |
| RPOD_SALTY_1 | P0A2E3 | 4.7 | 70530.63 |
| RPOD_SHEVD_1 | O24744 | 4.99 | 70030.68 |
| RPOD_HAEIN_1 | P43766 | 4.69 | 72084.92 |
| RPOD_RICTY_1 | Q68VQ5 | 5.42 | 73113.37 |
| RPOD_XANAC_1 | Q8PG33 | 4.88 | 70084.37 |
| RPOD_BORBU_1 | P52323 | 5.15 | 73643.06 |
| RPOD_XANCP_1 | Q8P4H2 | 4.89 | 69947.13 |
| Rho Factor | Accession number | pI | Molecular Weight |
| RHO_ECOLI_1 | P0AG30 | 6.75 | 47004.21 |
| RHO_MYCTU_1 | P9WHF3 | 5.56 | 65133.48 |
| RHO_SALTY_1 | P0A295 | 6.75 | 46993.18 |
| RHO_STRLI_1 | P52157 | 9.05 | 76544.17 |
| RHO_MICLU_1 | P52154 | 6.17 | 75030.63 |
| RHO_THEMA_1 | P38527 | 5.7 | 48301.94 |
| RHO_PSEAE_1 | Q9HTV1 | 6.4 | 47069.3 |
| RHO_BUCAI_1 | P57652 | 7.77 | 46888.26 |
| RHO_SHIFL_1 | P0AG33 | 6.75 | 47004.21 |
| RHO_SALTI_1 | P0A296 | 6.75 | 46993.18 |
| RHO_PSEFC_1 | P52155 | 7.05 | 46954.31 |
| RHO_RICTY_1 | Q68WL0 | 5.9 | 51333.77 |
| RHO_BORBU_1 | P33561 | 8.21 | 57625.26 |
| RHO_MYCBO_1 | P66029 | 5.56 | 65133.48 |
| RHO_FIBSS_1 | C9RLJ9 | 5.64 | 78252.25 |
| RHO_BACSU_1 | Q03222 | 6.51 | 48628.95 |
| RHO_HELPY_1 | P56466 | 6.5 | 49546.65 |
| RHO_AQUAE_1 | O67031 | 9.24 | 50091.53 |
| RHO_RHOS4_1 | P52156 | 5.37 | 47238.06 |
| RHO_DEIRA_1 | P52153 | 5.94 | 47131.19 |
| RHO_HAEIN_1 | P44619 | 6.16 | 46978.07 |
| RHO_ALLVD_1 | P52152 | 8.4 | 46553.7 |
| RHO_CHRVO_1 | Q7NXP1 | 6.31 | 47002.9 |
| RHO_STRRD_1 | D2B129 | 8.63 | 42569.34 |
| RHO_RHOBA_1 | Q7UGV0 | 5.98 | 57864.92 |
| RHO_AKKM8_1 | B2UR37 | 5.4 | 52945.44 |
| RHO_FUSNN_1 | Q8RG42 | 5.27 | 46169.02 |
| RHO_MYCLE_1 | P45835 | 5.35 | 66102.72 |
| RHO_RICFE_1 | Q4ULF7 | 6.21 | 51268.7 |
| RHO1_EHRCR_1 | P0CH92 | 6.69 | 46864.43 |
| RHO_SULMS_1 | C7LJY3 | 9.95 | 43128.16 |
| RHO_TREPA_1 | O83281 | 6.47 | 58265.76 |
| RHO_RICBR_1 | Q1RIJ6 | 6.21 | 50416.83 |
| RHO_GEMAT_1 | C1A5H8 | 10.18 | 81379.15 |
| RHO_NEIGO_1 | Q06447 | 6.01 | 47360.38 |
| RHO_RICCN_1 | Q92HL2 | 7.02 | 51277.84 |
| RHO_STRM9_1 | D1AWS1 | 6.04 | 46939.34 |
| RHO_XYLFA_1 | Q9PA21 | 7.86 | 46115.15 |
| RHO_RICPR_1 | Q9ZD24 | 6.2 | 51239.81 |
| NusA | Accession number | pI | Molecular Weight |
| NUSA_ECOLI_1 | P0AFF6 | 4.53 | 54870.92 |
| NUSA_MYCTU_1 | P9WIV3 | 6.42 | 37641.6 |
| NUSA_SALTY_1 | P37430 | 4.5 | 55425.7 |
| NUSA_SHIFL_1 | P0AFF9 | 4.53 | 54870.92 |
| NUSA_BUCAI_1 | P57459 | 5.36 | 55995.41 |
| NUSA_COXBU_1 | Q83BS0 | 4.59 | 56274.88 |
| NUSA_BORBU_1 | O51740 | 4.8 | 54816.74 |
| NUSA_RICPR_1 | Q9ZCZ7 | 5.06 | 56883.09 |
| NUSA_RICCN_1 | Q92HF4 | 4.96 | 56522.68 |
| NUSA_HAEIN_1 | P43915 | 4.55 | 55262.67 |
| NUSA_BACSU_1 | P32727 | 4.78 | 41725.26 |
| NUSA_HELPY_1 | P55977 | 5.64 | 44649.76 |
| NUSA_THET8_1 | P48514 | 5.74 | 43938.94 |
| NUSA_MYCLE_1 | Q9Z5J1 | 6.12 | 37735.64 |
| NUSA_MYCGE_1 | P47387 | 5.59 | 59751.45 |
| NUSA_MYCPN_1 | P75591 | 5.87 | 60272.1 |
| NUSA_MYCBO_1 | P0A5M3 | 6.42 | 37641.6 |
| NusG | Accession number | pI | Molecular Weight |
| NUSG_ECOLI_1 | P0AFG0 | 6.33 | 20400.32 |
| NUSG_MYCTU_1 | P9WIU9 | 4.7 | 25446.59 |
| NUSG_STRVG_1 | P27309 | 4.06 | 32591.82 |
| NUSG_THEMA_1 | P29397 | 9.03 | 40327.05 |
| NUSG_AQUAE_1 | O67757 | 6.87 | 27998.67 |
| NUSG_BACSU_1 | Q06795 | 5.43 | 20126.14 |
| NUSG_STRP6_1 | Q5XE43 | 4.54 | 20385.2 |
| NUSG_SALTY_1 | P0AA02 | 6.33 | 20414.35 |
| NUSG_YERPE_1 | Q8ZAP0 | 5.99 | 20383.27 |
| NUSG_VIBCH_1 | Q9KV35 | 5.55 | 20519.39 |
| NUSG_SALTI_1 | P0AA03 | 6.33 | 20414.35 |
| NUSG_SHIFL_1 | P0AFG2 | 6.33 | 20400.32 |
| NUSG_PSEAE_1 | Q9HWC4 | 5.75 | 20162.15 |
| NUSG_THET8_1 | P35872 | 5.42 | 20448.6 |
| NUSG_DEIRA_1 | Q9RSS6 | 4.9 | 20966.88 |
| NUSG_SYNY3_1 | P36265 | 5.92 | 23415.82 |
| NUSG_STAA8_1 | Q2G0P2 | 5.1 | 20663.69 |
| NUSG_HAEIN_1 | P43916 | 5.54 | 21381.2 |
| NUSG_VIBPA_1 | Q87KP9 | 5.56 | 20577.43 |
| NUSG_BUCAI_1 | P57151 | 9.39 | 20960.14 |
| NUSG_VIBVU_1 | Q8DD25 | 5.56 | 20641.48 |
| NUSG_BORBU_1 | O51355 | 7.82 | 21215.69 |
| NUSG_CAMJE_1 | Q9PI36 | 6 | 20195.27 |
| NUSG_CHLTR_1 | O84322 | 5.23 | 20745.83 |
| NUSG_LACLA_1 | Q9CDV7 | 4.55 | 21110.02 |
| NUSG_MYCGE_1 | P47300 | 5.65 | 36322.77 |
| NUSG_PASMU_1 | Q9CK84 | 6.03 | 21113.9 |
| NUSG_STAEQ_1 | Q5HRL6 | 5.09 | 20672.75 |
| NUSG_TREPA_1 | O83264 | 9.13 | 20928.3 |
| NUSG_CHLPN_1 | Q9Z9A5 | 5.03 | 20791.85 |
| NUSG_XYLFA_1 | Q9PA81 | 5.99 | 21208.24 |
| NUSG_HELPY_1 | P55976 | 6.98 | 20261.3 |
| NUSG_MYCBO_1 | P65590 | 4.7 | 25446.59 |
| NUSG_MYCLE_1 | Q9CBK0 | 4.8 | 24346.54 |
| NUSG_MYCPN_1 | P75049 | 5.21 | 36107.5 |
| NUSG_NEIMB_1 | P65592 | 6.03 | 20550.4 |
| NUSG_RICCN_1 | Q92J91 | 8.91 | 21685.03 |
| NUSG_BACHD_1 | Q9KGE7 | 5.42 | 20466.56 |
| NUSG_RICBR_1 | Q1RHC5 | 7.81 | 21501.79 |
| NUSG_RICFE_1 | Q4UKC9 | 8.6 | 21811.16 |
| NUSG_RICPR_1 | P50056 | 9.43 | 21601.2 |
| NUSG_RICTY_1 | Q68XN3 | 9.22 | 21720.23 |
| NUSG_STACT_1 | P36264 | 5.19 | 20650.71 |
| NUSG_STRCO_1 | P36266 | 4 | 32844.63 |
| NUSG_CHLMU_1 | Q9PK75 | 5.23 | 20717.77 |
| NUSG_STRGB_1 | P52852 | 4.03 | 33843.94 |
| NUSG_STRGR_1 | P36260 | 4.11 | 31809.83 |
